# Supplementary material for: Xpert Ultra stool testing to diagnose tuberculosis in children in Ethiopia and Indonesia: a model-based cost-effectiveness analysis
Source: BMJ Open. 2022 Jul 1;12(7):e058388. doi: 10.1136/bmjopen-2021-058388 (PMC9252203; doi:10.1136/bmjopen-2021-058388)
Supplement: Supplementary data [file bmjopen-2021-058388supp002.pdf]

Xpert Ultra stool testing to diagnose tuberculosis in children in Ethiopia and Indonesia: a model-based cost-effectiveness analysis.

Appendix 2a: Model parameter estimation

Model structure and description 2

Description of parameters from review 4

Spontaneous sputum expectoration (parameter a) 4

Fraction of TB bacteriologically positive under ideal conditions (parameter Fbc) 6

Prevalence of true TB among presumptive TB 8

Accuracy of clinical assessment in bacteriologically negative TB 13

Accuracy of bacteriological tests 14

Level of initial care-seeking 15

Summary of model parameters from review and distributions 16

Summary of other parameters 16

Parameters in common between countries from previous work 16

Parameters specific to Indonesia 17

Parameters specific to Ethiopia 17

Other parameters based on assumption 17

Description approach to expert opinion 18

References 20

Model structure and description

The model is implemented as a decision tree that matches our understanding of patient pathways of care. The structure of the tree is shown in Figure A1 along with the names of the probabilities of going down each path, and the names of the costs associated with each node (underneath node names; 0 if no costs are applied). Quantities such as probabilities or costs can depend on 'attributes' of patients entering; here, this means true TB status (yes or no) and child age group (0-4 or 5-14 years). The model calculates mean values of various quantities over the tree for a large number (10 thousand) of sampled input parameters and cohort characteristics (i.e., make up by attribute) to generate a probabilistic sensitivity analysis that is used for the generation of results. The quantities calculated over the tree are: the number of deaths; the cost to healthcare providers; the number of referrals; the number of assessments performed; the number of bacteriological assessments performed; the number of anti-TB treatments; the number of bacteriologically-confirmed anti-TB treatments; the number of anti-TB treatments initiated at PHC level; the number of anti-TB treatments initiated among bacteriologically-confirmed TB cases; a validation variable that should always total 1. The model was implemented in R using the HEdtree package.

All fundamental input parameters are treated as random variables with specified distributions to represent uncertainty. Labelled parameters on Figure A1 may depend in specified ways on these underlying fundamental input parameters. Most parameters appearing as labels in Figure A1 directly correspond to fundamental input parameters, and are named as such in parameter tables. However, there are three classes of exception: 1) parameters describing treatment and non-treatment outcomes; 2) parameters on early stages of the care cascade relating to bacteriological testing; 3) parameters describing the prevalence of attributes in the patient cohort, which are not shown on Figure A1.

The approach to outcomes (class 1 above) are based on previously published work[1] and are recapped below (see Table A9), along with some additional modelling details. This document focuses on the review work to inform new input parameters, many of which are related to parameters in classes 2 and 3 above.

Briefly, we assume that parameters *a* are determined by the ability of children in each age category to spontaneously expectorate sputum, i.e., an attempt to collect a spontaneously expectorate sputum is always made at PHC or hospital. Parameters *b* are based on data on the diagnostic accuracy of stool-approaches, but assume that only a fraction of all children in each age group (*Fbc*) are bacteriologically-confirmable under ideal circumstances. Since diagnostic accuracy is typically reported with respect to confirmed cases, we assume test sensitivity only applied to a fraction of *Fbc* of patients. Parameters *f, d,* and *h* for clinical assessments at PHC or hospital are assumed to be the same, and are informed by data we found to inform the diagnostic accuracy for clinical diagnosis of TB in each age group (i.e., these are sensitivity for true TB, and one minus specificity for true not TB). Importantly, we assume that under the intervention, a bacteriologically negative test is always followed up with clinical assessment (i.e., this assessment, which will be made in any case, is able to override a false-negative test result with unchanged sensitivity).

Mafirakureva N, *et al. BMJ Open* 2022; 12:e058388. doi: 10.1136/bmjopen-2021-058388

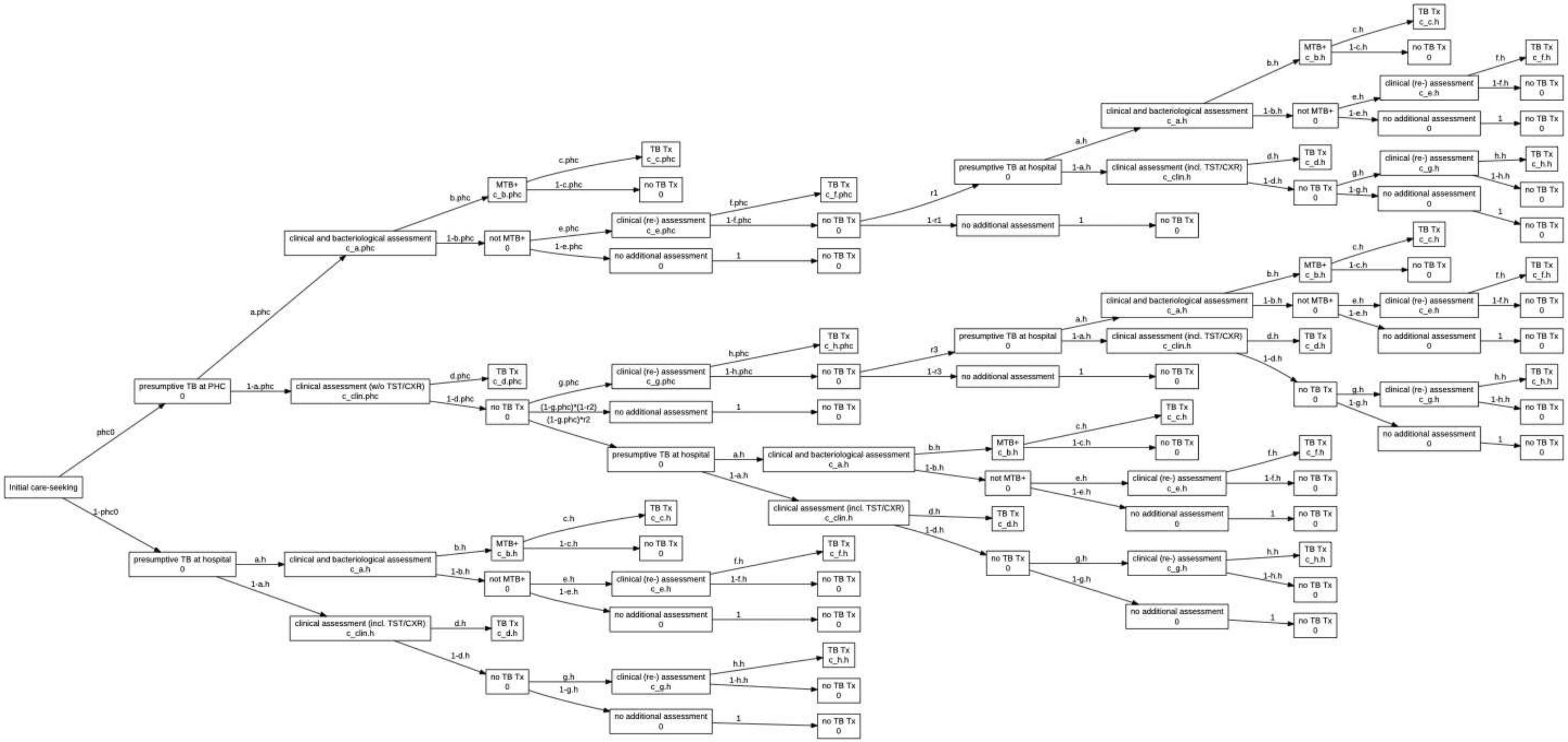

Figure A1 Diagram of modelled decision tree including the edge labels (names of probabilities for each path) and node cost names (underneath node names)

3

Description of parameters from review

The model was informed with parameters obtained from ongoing studies using the SOS stool method where available, a systematic literature search (see Appendix 1), and expert opinion for those parameters for which no published data was identified. This Appendix provides an overview of the original data and the summation methods used to quantify a parameter for each of the parameters used in the model.

Spontaneous sputum possible

For this parameter, we collected data from our own and published studies on the proportion of children that submitted a spontaneously expectorated sputum sample for diagnostic workup. We included those studies that accepted spontaneously expectorated sputum from all children that could produce such a sample and included other respiratory specimens (i.e., nasogastric (lavage) aspirates, nasopharyngeal aspirates, or induced sputum) for those children unable to spontaneously expectorate, reporting the number of specimens by type received per age group. Only two studies included in our comprehensive review of original peer-reviewed papers (Appendix 1) met these criteria[2, 3].

Table A1 Proportion of children who submitted spontaneously expectorated sputum, by age group.

Reference	Setting	Age group	Number of children	Proportion spontaneous sputum (95% CI if provided)
Kaswandani, Tiemersma et al, unpublished	in- and outpatients with symptoms or signs of presumptive TB in 10 secondary and tertiary care hospitals on Java, Indonesia	0-4 years	222	1.80% (0.67% - 4.72%)
		5-10 years	82	13.41% (7.54% - 22.73%)
Bates et al[2]	in-patients with primary or secondary admission diagnosis of TB at pediatric and child health department of Lusaka University hospital in Zambia	0-4 years	663	2.30%
		5-9 years	124	45.20%
		10-14 years	138	50.00%
Hanrahan et al[3]	outpatients with presumptive TB at 1 primary care clinic in Johannesburg, South Africa	2 months - 4 years	202	3.90%
		5-9 years	17	58.80%

Bates et al[2] collected sputum samples from all children who could expectorate while gastric lavage aspirates were obtained from children unable to expectorate. Hanrahan et al[3] collected a spontaneous sputum sample whenever possible. Sputum collection was guided and overseen by a dedicated paediatrician. If the child was unable to expectorate, one nasopharyngeal aspirate and one induced sputum sample were obtained by a nurse. We also collected relevant data in a study in Indonesia on the diagnostic accuracy of the SOS stool method with Xpert. Sample collection was overseen as per routine procedures in the facilities, but was usually done by a nurse. Collection of a

Mafirakureva N, et al. BMJ Open 2022; 12:e058388. doi: 10.1136/bmjopen-2021-058388

spontaneously expectorated sputum or an alternative specimen (either sputum induction (generally for children of 2 years and older) or nasogastric aspiration (for younger children)) was conducted as per nurse's judgement.

Table A1 summarizes the data extracted while Figure A2 plots the same data with 95% confidence intervals, using binomial confidence intervals only where counts were provided.
The proportions from the Indonesian studies were lower than those reported from the two published studies, especially for the older children, but may in fact be closer to the reality on the ground, as in the Indonesian study, no special efforts were undertaken to obtain spontaneous sputum from all children.

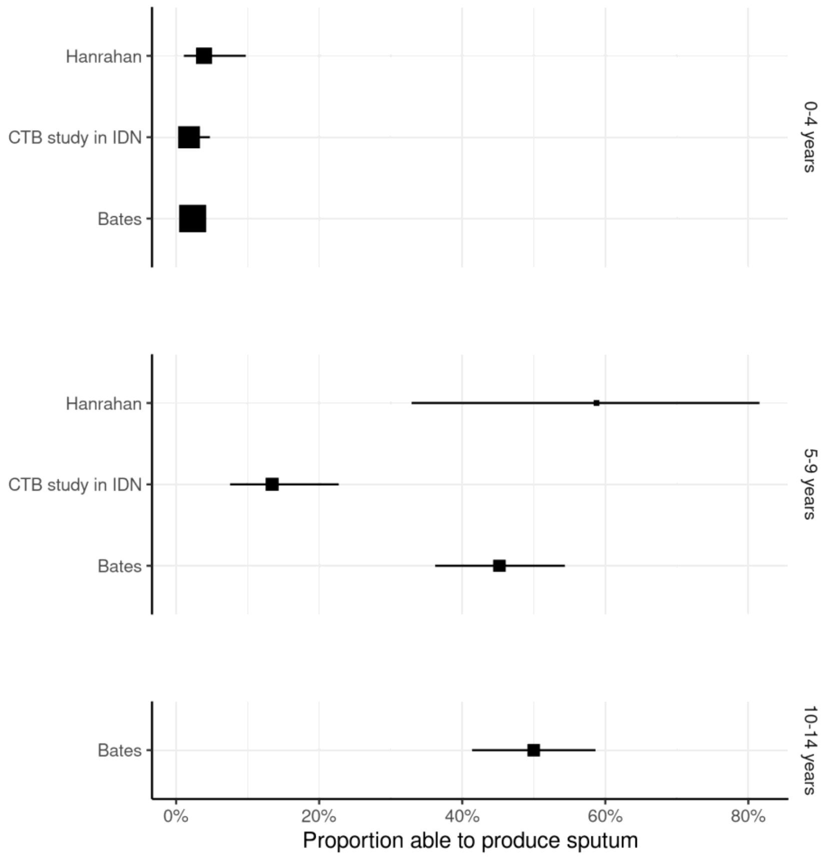

Figure A2. Proportion of children able to spontaneously expectorate sputum by age group with 95% uncertainty intervals.
Names refer to first authors of papers (also described in Table A1 and Appendix 1, Table A2). CTB: Challenge TB project; IDN: Indonesia.

The summary estimates from a random effects meta-analysis after pooling of the results were 2.4% (95% prediction interval (PI): 1.6 - 3.6%) for the rate of spontaneous sputum expectoration among children aged 0-4 years, and 38.9% (95% prediction interval [PI]: 0.098 - 78.8%) among children aged 5 years and above. Note that the 95%CI in this case was much narrower (21.7% - 59.4%).

Fraction of children bacteriologically confirmable

TB in children, especially children under 5 years of age, is often of paucibacillary nature and often no *M. tuberculosis* bacilli can be detected. Most evidence on the sensitivity of diagnostic tests is reported against a gold standard based on bacteriological confirmation; often sensitivity is very poor among children with bacteriologically negative TB. For our model, it was therefore important to understand what the maximum fraction of children for whom TB could be bacteriologically confirmed if an array of diagnostic tests were used. For this parameter, F_{bc}, we included studies that tested multiple different specimens of the same child, using sensitive diagnostics such as Mycobacteria Growth Indicator Tube (MGIT) culture and GeneXpert (Ultra). Four studies meeting these criteria were identified (Table A2). Figure A3 provides the point estimates with 95% uncertainty intervals.

It should be noted that all these four studies were conducted in Cape Town, South Africa, in only 4 different hospitals (Red Cross War Memorial Children's Hospital[4-6], New Somerset Hospital[4-6], Tygerberg Hospital[7] and Karl Bremer Hospital[7]), and included hospitalized children only. Restriction of the study populations to admitted (i.e., most ill) children may introduce bias to higher proportions of confirmable TB, as a positive correlation between bacterial load and seriousness of the illness is expected. For example, in another study including children with minimal TB (defined as non-severe, symptomatic, smear-negative TB), the disease was bacteriologically confirmed on a respiratory sample in only 14.16% of the cases.[8] All children had submitted at least 2 specimens of gastric lavage, gastric washing or sputum, which were tested by culture (MGIT and Lowenstein-Jensen medium), Xpert MTB/Rif and Xpert Ultra.

Mafirakureva N, *et al. BMJ Open* 2022; 12:e058388. doi: 10.1136/bmjopen-2021-058388

Table A2 Fraction of TB that was bacteriologically confirmed from studies applying sensitive diagnostics to multiple specimens.

Reference	Setting	Type and number of specimens taken	Type of diagnostic tests conducted	Number of children enrolled	Number of children treated for TB	Bacteriological confirmation	Fraction with bacteriological confirmation of TB
Nicol et al[4]	children aged <15 years admitted with presumptive pulmonary TB (incl. at least cough of >2weeks plus another sign or symptom) to 2 hospitals in Cape Town, South Africa	2 IS taken at least 4h apart; n=385 with 2 IS, n=67 with one IS specimen	Fluorescent smear microscopy and Xpert MTB/Rif on concentrated sample, MGIT culture	452	n=216: 69/70 definite TB , 147/216 possible TB (incl. 6 with Xpert MTB+ results)	n=76: 70 culture-positive, 6 Xpert positive, culture-negative	34.72%
Walters et al[9]	children aged <13 years presenting to 2 hospitals in Cape Town, South Africa with presumptive intrathoracic TB	sputum (5 years or older)/NGA (<5 years) + IS + NPA), stool (max 7 samples). All respiratory samples tested on smear + MGIT and partly on GX, stool GX	respiratory samples: fluorescent smear microscopy and Xpert MTB/Rif on concentrated sample, MGIT culture if collected by study team. Smear and culture if collected by hospital staff. Stool samples: Xpert and culture (the latter only until half-way the study)	379	n=170: 73 with bacteriologically confirmed TB, 69 with unconfirmed TB, 28 with unlikely TB	n=73: 71 culture-or Xpert positive on respiratory sample, 1 Xpert-positive on stool sample and 1 culture-positive on stool sample	42.94%
Zar et al (2012)[5]	Children aged <15 years with presumptive TB hospitalized in Cape Town, South Africa, because of severe pneumonia, need for oxygen/intravenous therapy, or social conditions precluding home care	2 NPA (taken at least 4h apart) and 2 IS (taken at least 30 min after NPA, and taken at least 4h apart); n=396 with 2 paired IS and NPA, n=139 with 1 paired IS and NPA	Fluorescent smear microscopy and Xpert MTB/Rif on concentrated sample, MGIT culture	535	n=283: 87 with definite TB, 194 with possible TB	n=98: 87 culture-positive and 11 Xpert NPA/IS positive and culture-negative (of whom 9 were treated)	33.92%
Zar et al (2019)[6]	Children aged <15 years, hospitalized for suspected TB in Cape Town, South Africa	2 NPA (taken at least 4h apart) and 2 IS (taken at least 30 min after NPA, and taken at least 4h apart); n=130 with 2 paired IS and NPA, n=65 with 1 paired IS and NPA	Fluorescent smear microscopy and Xpert Ultra (for 2 NPA and 1 IS) on concentrated sample, MGIT culture	195	n=144: 40 with confirmed TB, 104 with unconfirmed TB (not sure though if all were treated)	n=48: 40 culture-positive, between 5 and 9 Xpert Ultra NPA/IS positive*, culture-negative	31.25 - 34.03%*

* The exact number of Xpert-positive, culture-negative cases does not become clear from the paper: there were between 5 (IS and NPA results completely overlap) and 9 (no overlap between IS and NPA results) of such cases.

Mafirakureva N, *et al. BMJ Open* 2022; 12:e058388. doi: 10.1136/bmjopen-2021-058388

The summary estimate from a random effects meta-analysis was 38.0% (95% prediction interval [PI]: 33.1 - 43.1%).

For the above studies, most children were under 5 years of age. The fraction of children with TB in whom it is possible to obtain bacteriological confirmation is thought to be higher for the 5-14 years age group, but we did not find any suitable data to directly inform this. For the 5-14 year old age group, we therefore divided the proportion of children aged 5-14 reported from South African enhanced surveillance data in du Preez et al.[10] by the spontaneous sputum fraction for this age group from above. This assumes that the fraction of children bacteriologically confirmed in routine practice is the product of the fraction who can spontaneously expectorate sputum, and the fraction who would be bacteriologically confirmed with enhanced sample collection and multiple testing (F_{bc}). This yielded an estimate of 58.0% (50.5 - 65.8%) for F_{bc} in this age group.

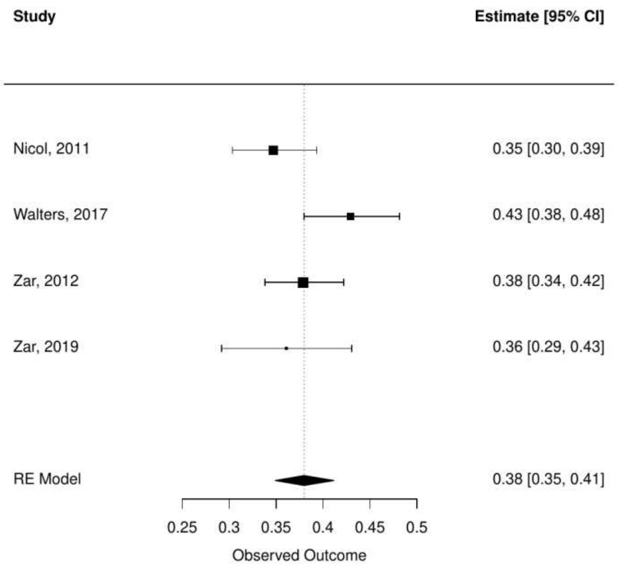

Figure A3. Meta-analytic results for the fraction of children with bacteriologically positive TB under ideal circumstances.

Prevalence of true TB in presumptive

For this parameter, from our systematic review (Appendix 1), we selected studies that reported the number of children with presumptive TB and the number of children diagnosed with TB (by method) during the study period.

We restricted to studies that reported using case definitions based on one of the Graham consensus definitions,[11, 12] or the NIH definition, including confirmed/probable TB as TB or the number starting treatment if this was stated (see Table A3). Where age categories reported were not exactly 0-4 years, 5-14 years or 0-14 years, we approximated the age category reported by the studies by its closest match, aggregating over counts if necessary. We performed a random-effects meta-analysis for each age grouping (see Figure A4), finding a summary estimate of 45% (95% prediction interval [PI]: 7.7 - 89%). There was high heterogeneity and wide prediction intervals, with no clear difference between the 0-4 year and 5-14 year age group. We therefore based our parametrization on the pooled 0-14 year analysis, using the midpoint and prediction interval to inform a beta distribution.

Table A3 Studies reporting TB prevalence among presumptive TB patients using NIH or Graham case-definitions.

Author	Year	Design	Setting	Clinical diagnosis	Specimens and tests	Age group	Included	Diagnosed with TB	Type of diagnosis
Bacha[13]	2017	Retrospective descriptive	children evaluated for presumptive TB and/or referred for TB treatment in 1 regional referral hospital in Southern Highlands Zone of Tanzania serving a child population of 3.2 mln children	as per internationally proposed criteria (see Graham et al. 2015)	SSM and Xpert on sputum or IS if unable to expectorate. Culture only if there was a 2nd sample (89.4% of children)	0-14 years	455	120	21 confirmed, 99 probable, 37 possible TB
Elhassan[14]	2016	Cross-sectional	Children with presumptive TB presenting to 5 TB centers in Khartoum state, Sudan	Confirmed TB: cough>2wks AND culture+, Probable: cough>2wk AND CXR abnormal AND HH contact; Possible: Cough>2wk AND HH contact AND TST+	SSM (ZN and auramine fluorescence), IS6110 PCR and LJ culture on sputum (if 7+y) or NGA (if<7y)	0-18y (0-15y as per Methods but 0-18y per Tables & Figures)	197	125	32 confirmed, 56 probable, 37 possible
								32	LJ culture confirmed
						<=6y	86	47	3 confirmed, 29 probable, 15 possible
								3	LJ culture confirmed
						7-12 y	63	40	10 confirmed, 17 probable, 13 possible
								10	LJ culture confirmed
						13-18y	48	38	19 confirmed, 10 probable, 9 possible
								19	LJ culture confirmed
Giang[15]	2015	Cross-sectional	HIV negative children presenting with presumptive TB at a sub-national TB referral hospital in Ho Chi Minh City, Vietnam	at least 1 symptom suggestive of TB plus a positive culture or smear, or plus CXR suggesting TB, positive response to TB therapy, documented close contact with TB patient, or positive TST	concentrated SSM and Xpert, MGIT culture on an average of 2 samples (NS)	0-14 years	150	131	38 confirmed, 60 probable, 33 possible
							150	38	culture or smear-positive (confirmed) PTB
							150	46	Xpert-positive (among confirmed, probable and possible cases only)
							150	39	culture-positive (among confirmed, probable and possible cases only)
Hanrahan [3]	2019	cross-sectional	children with presumptive TB incl. symptomatic child HH contacts of adult TB patients regardless of symptom duration presenting at a high-volume, primary health-care clinic which provides outpatient care for a densely populated urban and peri-urban impoverished community of about	Per Graham, 2015. Confirmed TB: microbiologically positive by SSM, culture or Xpert on any sample. Unconfirmed TB: no microbiological confirmation but >=2 of CXR suggesting TB, positive response to TB treatment, TB contact history, or TST+.	concentrated FM, Xpert, and MGIT culture on 1 spontaneous sputum sample or 1 NPA+1 IS if unable to produce sputum; 1 stool	60 days to ≤10 years	119	105	4 confirmed, 101 unconfirmed TB
								4	confirmed TB

Author	Year	Design	Setting	Clinical diagnosis	Specimens and tests	Age group	Included	Diagnosed with TB	Type of diagnosis
			200,000–300,000 (18% children <10y)						
Moussa[16]	2016	Cross-sectional	children with clinical signs of PTB presenting at 1 tertiary care hospital in Cairo, Egypt	at least 1 symptom suggestive of TB plus "microbiological confirmation", or plus CXR suggesting TB, positive response to TB therapy, documented close contact with TB patient, or immunological evidence of MTB infection	SSM, LJ culture on 2 (induced) sputum samples, Xpert MTB/Rif on 2 stool samples	1-15 years	115	107	36 confirmed, 61 probable, 10 possible
							115	36	confirmed PTB
						1-5 years	41	38	10 confirmed, 25 probable, 3 possible
						6-15 years	74	69	26 confirmed, 36 probable, 7 possible
						1-5 years	41	10	confirmed PTB
						6-15 years	74	26	confirmed PTB
Myo[17]	2018	Cross-sectional	Children with suspected PTB presenting at tertiary care pediatric hospital in Mandalay, Myanmar	revised NIH classification: culture or Xpert positive, or at least 2 of symptoms/ signs suggesting TB, CXR consistent with TB, TB exposure or immunological evidence of MTB, or a positive response to TB treatment	concentrated SSM, direct Xpert MTB/Rif and LJ culture on 1 GLA	1 month-12 years	231	121	38 confirmed, 83 unconfirmed
							231	38	culture- or Xpert-positive (confirmed) PTB
Nicol[18]	2013	Cross-sectional	Children presenting with presumptive TB at 1 primary healthcare clinic and 1 tertiary care hospital in Cape Town, South Africa	culture-positive or any other started on TB treatment, or not started on TB treatment but with persistent TB suggestive symptoms and signs at 3-month follow-up	concentrated Xpert and MGIT on 2 IS and Xpert testing of 2 aliquots from 1 stool	0-14 years	115	65	17 definite, 48 possible
							115	17	culture-positive (definite) PTB
Nicol[19]	2019	Cross-sectional	Children presenting with presumptive TB at 1 tertiary care hospital in Cape Town, South Africa	culture-positive or any other started on TB treatment, or not started on TB treatment but with persistent TB suggestive symptoms and signs at 3-month follow-up	Xpert and MGIT on 2 IS (2 oral swabs with quantitative PCR, not incl. in diagnosis)	0-14 years	165	121	40 confirmed, 81 unconfirmed
							165	40	culture-positive (confirmed) PTB
Reither[20]	2014	cross-sectional	Children presenting with presumptive TB at 2 research sites in Tanzania and 1 hospital in Kampala, Uganda	symptoms suggestive of TB and AFB+ smear or abnormal CXR suggestive for TB, or CXR not clearly suggesting TB but no alternative Dx and complete resolution of	concentrated SSM, Xpert, MGIT and LJ culture on sputum/IS (1-5 samples per child)	2 months-15 years	451	147	37 confirmed, 48 highly probable, 62 probable PTB
							451	37	culture-positive (confirmed) PTB
						2 months-5 years	211	74	16 confirmed, 26 highly probable, 32 probable PTB
							211	16	culture-positive (confirmed) PTB

Author	Year	Design	Setting	Clinical diagnosis	Specimens and tests	Age group	Included	Diagnosed with TB	Type of diagnosis
				symptoms/signs on TB treatment		6-10 years	133	39	10 confirmed, 13 highly probable, 16 probable PTB
							133	10	culture-positive (confirmed) PTB
						11-15 years	106	34	11 confirmed, 9 highly probable, 14 probable PTB
							106	11	culture-positive (confirmed) PTB
Sabi[21]	2016	cross-sectional	Children presenting with presumptive TB in 1 zonal hospital in NW Tanzania serving a population of 13 mln. 91% of children admitted to hospital	using 4 different published clinical score charts incl. TST and CXR resutls; but for analysis using Graham et al 2012	FM, Xpert, LJ culture on IS	2 months-12 years	192	40	10 confirmed, 10 probable, 20 possible PTB
							192	10	culture positive (confirmed) PTB
Sorsa[22]	2020	Retrospective document review (historical cross-sectional before- after study)	Children presenting with presumptive TB at Asella Teaching and Referral hospital serving a population of approx. 4 mln in South-Central Ethiopia; Jan 2014-Dec 2017 with Xpert as intervention installed Jan 2016	Confirmed: >=1 TB symptom (cough>=2 wk, contact with TB patient, fever, weight loss, failure to gain weight) and microbiologically confirmed by SSM/Xpert; Probable: >=2 of TB contact history, clinical feature suggesting TB, TST+, CXR abnormal suggesting TB	not specified, but likely direct SSM or Xpert on a sputum sample, not clear if NGA was also done.	<15 y	775	453	142 confirmed, 311 probable
								142	confirmed (SSM/Xpert)
							404 ('before'-period)	254	54 confirmed, 200 probable
								54	confirmed (SSM)
							371 ('after'-period)	199	88 confirmed, 111 probable
								88	confirmed (Xpert)
Walters[9]	2018	prospective cohort	Children (12.5% HIV+) with suspected PTB at two public referral hospitals offering general and specialized pediatric care (Rahima Moosa M&C hospital Johannesburg and Desmond Tutu TB center serving 2 hospitals in Cape Town)	per Graham, 2015	SSM, Xpert and MGIT culture on 1-2 respiratory specimens (1 spontaneous sputum or IS, + 1 GA in subset of children aged <5)	no range provided; median 15.5 month, IQR, 10.9–24.3 months	148	42	treated for TB
								63	3 confirmed, 60 unconfirmed
								3	confirmed TB (culture or Xpert+)
								2	Culture+
Walters , van der Zalm[7]	2017	prospective	Children with suspected intrathoracic TB presenting to Tygerberg Hospital and Karl Bremer Hospital in Cape Town, South Africa, Apr 2012-Aug 2015	per Graham, 2015	2x(Sputum/GA + IS + NPA), stool (max 7 samples). All respiratory samples tested on FM + MGIT and partly on GX, stool GX	<13 y; med: 15.9 months IQR 9.2-29.3	379	258	73 confirmed, 185 unconfirmed TB
								73	confirmed TB (71 detected on culture/Xpert non-stool samples, 1 on stool culture, 1 on stool Xpert)
								71	confirmed TB (on non-stool samples)
								170	TB treatment initiated (reference standard)
Zar[23]	2013	prospective					384	197	30 definite, 167 possible TB

Author	Year	Design	Setting	Clinical diagnosis	Specimens and tests	Age group	Included	Diagnosed with TB	Type of diagnosis
			Children presenting with suspected pulmonary TB at 1 primary care clinic in Khayelitsha, Cape Town, South Africa, Aug 2010-Jul 2012	definite TB: culture+; Possible TB: receiving TB treatment + all whose symptoms/ signs at FU did not resolve if not receiving TB treatment	concentrated FM, concentrated Xpert, MGIT culture on IS+NPA: 80% 2 paired IS+NPA; 20% 1 paired IS+NPA	<15 y; median 38.3 m (IQR:21.2-56.5)		30	definite TB
								180	started on TB treatment

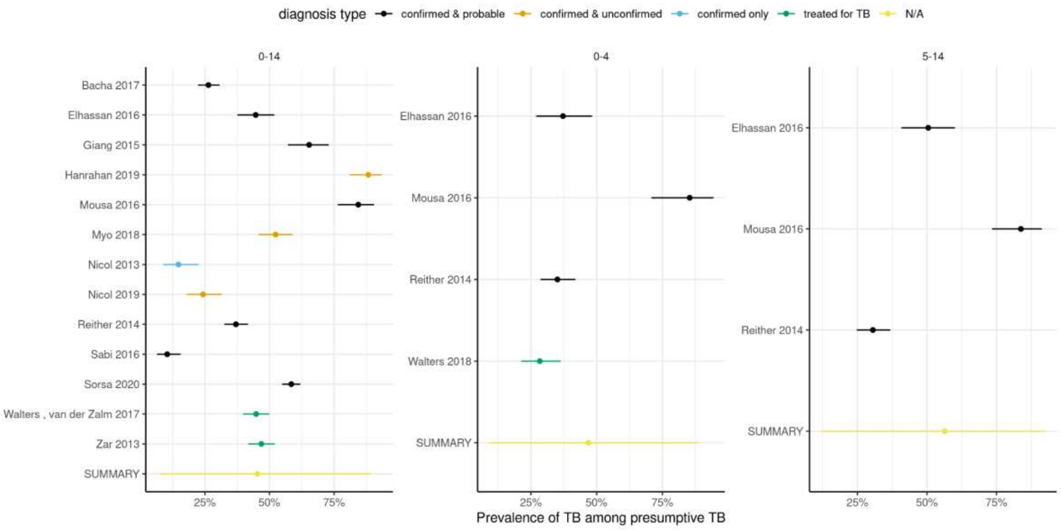

Figure A4 Forest plots for age groups 0-14 years, 0-4 years and 5-14 years of the prevalence of TB among presumptive TB

Accuracy of clinical assessment in bacteriologically negative TB

Sensitivity and specificity of clinical diagnosis

Assessing the diagnostic accuracy of clinical diagnosis for TB in children under routine care is challenging given the absence of a gold standard. Pearce et al.[24] systematically reviewed the accuracy of score-based approaches to diagnosing TB in children, and found one study (van Beekhuizen[25]) which can be interpreted as giving a sensitivity assessment of 62%, and specificity of 95%. The more recent cohort study by Marais et al[26] suggested a sensitivity of 62.6% and specificity of 89.8% among 428 children aged ≤13 years investigated for TB in South Africa. Restricting to children under 3 or those living with HIV, sensitivity was lower: sensitivity 51.8% (specificity 92.5%) for HIV-negative children under 3 years of age; sensitivity of 56.2% (specificity 61.8%) for children living with HIV.

The study of Beneri et al.[27] compared two case definitions within a trial context. Excluding bacteriologically confirmed TB, and counting NIH-unlikely TB as negative gives the cross-tabulation in Table A4.

Table A4 Aggregated data from Beneri et al.[27]

Classification for bacteriologically negative children		National Institute of Health (NIH)	
		TB+	TB-
P1041	TB+	93	1
	TB-	44	7

Evaluating the stricter trial (P1041) against the NIH case definition yields a sensitivity of 67.9% and a specificity of 87.5%.

A recent paper by Gunasekera et al.[28] developed an optimized scoring approach to TB diagnosis in children in South Africa, and reported a sensitivity of 71.5% when restricting the tool to inputs only from clinical evaluation (i.e. excluding Xpert MTB/Rif and chest X-ray).

We also considered the WHO estimated case-detection ratio for TB in each country and age group (typically 40-50% in relevant contexts). This approach is problematic because these estimates have large uncertainty, and also because CDR factors in children who did not present for care and who were diagnosed but not reported. It therefore is likely a lower bound for the sensitivity of TB detection algorithms in each country.

Given these data, and the likelihood that trials and optimized diagnostic scores may overestimate accuracy, we opted to use the estimates from Marais et al.[26] We used the estimate for HIV-negative children under 3 for all children under 5, and the overall estimate for children aged 5-14 years (see Table A5).

Table A5 Parameters used for accuracy of clinical diagnosis

**Name**	**Distribution**	**Description**	**Source**	**Mean (IQR)**
spec.clinu3	B(83.25,6.75)	Specificity of clinical dx < 5 years	Marais 2006[26]	0.928 (0.908 - 0.945)
sens.clinu3	B(46.62,43.38)	Sensitivity of clinical dx < 5 years	Marais 2006[26]	0.518 (0.482 - 0.554)
spec.clin	B(80.82,9.18)	Specificity of clinical dx 5-14 years	Marais 2006[26]	0.901 (0.878 - 0.921)
sens.clin	B(56.34,33.66)	Sensitivity of clinical dx 5-14 years	Marais 2006[26]	0.627 (0.592 - 0.661)

Accuracy of bacteriological tests

Sensitivity and specificity of Xpert and smear microscopy on sputum, and of Xpert on stool

For diagnostic tests other than stool, we used the accuracies quoted by the systematic review of Detjen et al.,[29] see Table A6.

Table A6 Diagnostic accuracies reported by Detjen et al.[29]

**Diagnostic**	**Sample**	**Reference**	**Sensitivity (95% CI & PI)**	**Specificity (95% CI & PI)**
Xpert	Expectorated/Induced Sputum	Culture	62% (51–73; 30–87)	98% (97–99; 90–100)
Xpert	Gastric lavage	Culture	66% (51–81; 33–91)	98% (96–99; 91–100)
Xpert	Expectorated /Induced Sputum	Clinical for C-ve	2% (1–3; 0–6)	100% (99–100; 99–100)
Microscopy	Expectorated	Culture	26% (14–39; 4–69)	100% (99–100; 94–100)

	/Induced Sputum			
Microscopy	Gastric lavage	Culture	22% (12–35; 6–51)	99% (97–100; 93–100)

For the accuracy of stool for diagnosing TB in children, we used the two systematic reviews and meta-analyses: MacLean et al.[30] and Mesman et al.[31] (Table A7).

Table A7 Parameters found by review on diagnostic test accuracy

**Name**	**Distribution**	**Description**	**Source**	**Mean (IQR)**
sens.stool	B(20.39,15.3)	Sensitivity of Xpert on stool in bac+ children	Mesman 2019[31]	0.571 (0.515 - 0.627)
spec.stool	B(326.97,6.67)	Specificity of stool in bac+ children	Mesman 2019[31]	0.981 (0.975 - 0.986)
sens.xpert	B(45.75,28.04)	Sensitivity for C+ of Xpert on sputum	Detjen 2015[29]	0.621 (0.582 - 0.659)
spec.xpert	B(736.91,15.03)	Specificity for C+ of Xpert on sputum	Detjen 2015[29]	0.980 (0.977 - 0.984)
sens.sm	B(12.03,34.26)	Sensitivity for C+ of SM on sputum	Detjen 2015[29]	0.257 (0.215 - 0.302)
spec.sm	B(759.66,3.81)	Specificity for C+ of SM on sputum	Detjen 2015[29]	0.995 (0.994 - 0.997)

Level of initial care-seeking

We found no data specific to paediatric TB to inform the proportion of children initially seeking care at primary healthcare level. We ultimately relied on estimates of initial care seeking for Ethiopia and Indonesia made in two TB patient pathway analysis (PPA) papers, namely Fekadu et al.[32] and Surya et al.[33] We included care sought in the private and public sectors, mapping primary care level to the levels L0 and L1 defined in the papers. The former suggested 89.6% of children initially seek care at primary level in Ethiopia; the latter that 92.8% of children initially seek care at primary level in Indonesia. In the absence of data to inform uncertainty, and given the quality of this evidence for our question, we assumed the 95% uncertainty interval was at +/- 10% points around the central estimate.

Summary of model parameters from review and distributions

Table A8 Parameters informed by analyses above

Name	Distribution	Description	Source	Mean (IQR)
spont.sput.u5	B(21.572,877.28)	Spontaneous sputum possible (0-4)	see methods	0.024 (0.020 - 0.027)
spont.sput.o5	B(2.59,4.07)	Spontaneous sputum possible (5-14)	see methods	0.377 (0.254 - 0.512)
Fbc.u5	B(137.19,223.84)	Fraction of children bacteriologically confirmable <5	see methods	0.380 (0.363 - 0.397)
Fbc.o5	B(97.76,45.12)	Fraction of children bacteriologically confirmable 5-14	see methods	0.684 (0.659 - 0.711)
p_truetb	B(3.10,1.85)	Prevalence of true TB in presumptive	see methods	0.625 (0.484 - 0.783)
spec.clinu3	B(83.25,6.75)	Specificity of clinical dx	Marais 2006[26]	0.928 (0.908 - 0.945)
sens.clinu3	B(46.62,43.38)	Sensitivity of clinical dx	Marais 2006[26]	0.518 (0.482 - 0.554)
spec.clin	B(80.82,9.18)	Specificity of clinical dx	Marais 2006[26]	0.901 (0.878 - 0.921)
sens.clin	B(56.34,33.66)	Sensitivity of clinical dx	Marais 2006[26]	0.627 (0.592 - 0.661)
phc0_e	B(31.18,3.62)	Proportion of first care-seeking at PHC for Ethiopia	Fekadu 2017[32]	0.896 (0.777 - 0.973)
phc0_i	B(22.89,1.78)	Proportion of first care-seeking at PHC for Indonesia	Surya 2017[33]	0.928 (0.801 - 0.992)

Summary of other parameters

Parameters in common between countries from previous work

Most the of the CFR parameters are based on Jenkins et al.[1]

Table A9 Parameters informed by the literature review

Name	Distribution	Description	Source	Mean (IQR)
cfrontxY	LN(-3.96,0.64)	CFR children <5 on TB treatment	Jenkins et al 2017[1]	0.019 (0.012 - 0.029)
cfrontxO	LN(-4.82,0.48)	CFR children 5-14 on TB treatment	Jenkins et al 2017[1]	0.008 (0.006 - 0.011)
cfrnotxY	LN(-0.83,0.08)	CFR children <5 without TB treatment	Jenkins et al 2017[1]	0.436 (0.413 - 0.460)
cfrnotxO	LN(-1.90,0.12)	CFR children 5-14 without TB treatment	Jenkins et al 2017[1]	0.149 (0.137 - 0.162)

Parameters specific to Ethiopia

r2 in particular was adjusted upwards after consultation to reflect a low confidence with child TB diagnosis and management at primary level.

Table A10 Parameters specific to Ethiopia not included above

Name	Distribution	Description	Source	Mean (IQR)
fracu5	B(7.504,12.47)	Fraction of presumptive TB under 5	Based on fraction of WHO TB < 5	0.371 (0.300 - 0.447)
r1	B(1,15)	Referral PHC -> H after clinical re-assessment following bac-	Expert opinion	0.045 (0.019 - 0.088)
r2	B(8,2)	Referral PHC -> H after initial clinical assessment w/o bac	Expert opinion	0.800 (0.728 - 0.899)
g.phc	B(1,15)	Clincial re-assessment, PHC	Expert opinion	0.045 (0.019 - 0.088)

Parameters specific to Indonesia

r1 in particular was adjusted upwards after consultation to reflect a low confidence in bacteriologic testing for child TB.

Table A11 Parameters specific to Indonesia not included above

Name	Distribution	Description	Source	Mean (IQR)
fracu5	B(69.37,65.49)	Fraction of presumptive TB under 5*	based on fraction of WHO TB < 5	0.514 (0.485 - 0.543)
r1	B(2,8)	Referral PHC -> H after clinical re-assessment following bac-	Expert opinion	0.200 (0.107 - 0.272)
r2	B(5,5)	Referral PHC -> H after initial clinical assessment w/o bac	Expert opinion	0.500(0.391 - 0.607)
g.phc	B(1,15)	Clincial re-assessment, PHC	Expert opinion	0.045 (0.019 - 0.088)

* based on the proportion of TB cases under 5 among all child TB cases (<15y)

Other parameters based on assumption

Note: many of these parameters could potentially be made country-specific, but currently are not.

Table A12 Parameters without direct evidence based on assumptions

NAME	DISTRIBUTION	DESCRIPTION	SOURCE	Mean (IQR)
c.phc	B(95,5)	Proportion of bacteriologically confirmed children initiating anti-TB treatment, PHC	assumption	0.953 (0.937 - 0.966)
c.h	B(95,5)	Proportion of bacteriologically confirmed children initiating anti-TB treatment, H	assumption	0.953 (0.937 - 0.966)
e.phc	B(1,15)	Clinical re-assessment after bac-, PHC	assumption	0.045 (0.019 - 0.088)
e.h	B(1,15)	Clinical re-assessment after bac-, H	assumption	0.045 (0.019 - 0.088)
g.h	B(1,15)	Clinical re-assessment, H	assumption	0.045 (0.019 - 0.088)

Mafirakureva N, *et al. BMJ Open* 2022; 12:e058388. doi: 10.1136/bmjopen-2021-058388

r3	B(5,5)	Referral PHC -> H after clinical re-assessment w/o bac	assumption	0.500(0.391 - 0.607)

Mafirakureva N, *et al. BMJ Open* 2022; 12:e058388. doi: 10.1136/bmjopen-2021-058388

Description approach to expert opinion

For parameters for which no data was found in published literature, our best estimate based on the practical experience from the authors working in Ethiopia (AB and MG) and Indonesia (NK and RT) was included. NK and RT are experienced pediatricians working in large tertiary care settings in Indonesia. Both are active in the TB section of the Indonesian Association of Pediatricians, of which currently is the chairperson. RT has ample research experience in the field of diagnosing childhood TB in primary, secondary and tertiary care settings. AB and MG are both working for the local KCNV office. AB is a pediatrician with experience in the clinical, research and programmatic settings. He is a member of the Ethiopian Pediatric Association. MG is an senior M&E advisor with up-to-date practical experience in rural and urban sites involved in childhood TB projects run by KNCV.

Data was needed to inform parameters on the proportion of children with bacteriologically confirmed TB started on treatment (*c.phc* and *c.h*), the proportion clinically reassessed after initial bacteriological exclusion of TB (*e.phc* and *e.h*), the proportion of children clinically reassessed after short broad-course of antibiotics (*g.phc* and *g.h*), and about referrals from the primary to higher (hospital) levels (*r1*, *r2* and *r3*).

A data collection tool was distributed to the experts, in which per parameter, their best guess and the minimum and maximum value they considered reasonable could be filled (Table). Then, several online sessions were organized. The first session served to explain the data collection tool. The second session served to discuss the completed tool and solve differences in opinion between the experts where needed. A third session was organized to present the model output using the experts' best estimates. In this session, the set of parameters was further adapted to come to model outputs that seemed reasonable for the country.

Mafirakureva N, *et al. BMJ Open* 2022; 12:e058388. doi: 10.1136/bmjopen-2021-058388

References

1. Jenkins, H.E., et al., *Mortality in children diagnosed with tuberculosis: a systematic review and meta-analysis.* The Lancet. Infectious diseases, 2017. **17**(3): p. 285-295.
2. Bates, M., et al., *Assessment of the Xpert MTB/RIF assay for diagnosis of tuberculosis with gastric lavage aspirates in children in sub-Saharan Africa: a prospective descriptive study.* The Lancet Infectious Diseases, 2013. **13**(1): p. 36-42.
3. Hanrahan, C.F., et al., *Diagnostic strategies for childhood tuberculosis in the context of primary care in a high burden setting: the value of alternative sampling methods.* Paediatrics and International Child Health, 2018. **39**(2): p. 88-94.
4. Nicol, M.P., et al., *Accuracy of the Xpert MTB/RIF test for the diagnosis of pulmonary tuberculosis in children admitted to hospital in Cape Town, South Africa: a descriptive study.* The Lancet. Infectious diseases, 2011. **11**(11): p. 819-824.
5. Zar, H.J., et al., *Rapid molecular diagnosis of pulmonary tuberculosis in children using nasopharyngeal specimens.* Clinical infectious diseases : an official publication of the Infectious Diseases Society of America, 2012. **55**(8): p. 1088-1095.
6. Zar, H.J., et al., *Tuberculosis Diagnosis in Children Using Xpert Ultra on Different Respiratory Specimens.* American journal of respiratory and critical care medicine, 2019. **200**(12): p. 1531-1538.
7. Walters, E., et al., *Xpert MTB/RIF on Stool Is Useful for the Rapid Diagnosis of Tuberculosis in Young Children With Severe Pulmonary Disease.* The Pediatric infectious disease journal, 2017. **36**(9): p. 837-843.
8. Ssengooba, W., et al., *Accuracy of Xpert Ultra in Diagnosis of Pulmonary Tuberculosis among Children in Uganda: a Substudy from the SHINE Trial.* Journal of clinical microbiology, 2020. **58**(9): p. e00410-20.
9. Walters, E., et al., *Molecular Detection of Mycobacterium tuberculosis from Stools in Young Children by Use of a Novel Centrifugation-Free Processing Method.* Journal of clinical microbiology, 2018. **56**(9): p. e00781-18.
10. du Preez, K., et al., *The Impact of the Evolving Human Immunodeficiency Virus Response on the Epidemiology of Tuberculosis in South African Children and Adolescents.* Clinical Infectious Diseases, 2021.
11. Graham, S.M., et al., *Evaluation of tuberculosis diagnostics in children: 1. Proposed clinical case definitions for classification of intrathoracic tuberculosis disease. Consensus from an expert panel.* The Journal of infectious diseases, 2012. **205 Suppl 2**(Suppl 2): p. S199-S208.
12. Graham, S.M., et al., *Clinical Case Definitions for Classification of Intrathoracic Tuberculosis in Children: An Update.* Clinical infectious diseases : an official publication of the Infectious Diseases Society of America, 2015. **61Suppl 3**(Suppl 3): p. S179-S187.
13. Bacha, J.M., et al., *Why being an expert - despite xpert -remains crucial for children in high TB burden settings.* BMC infectious diseases, 2017. **17**(1): p. 123-123.
14. Elhassan, M.M., et al., *Challenges in diagnosing tuberculosis in children: a comparative study from Sudan.* International Journal of Infectious Diseases, 2016. **43**: p. 25-29.
15. Giang, D.C., et al., *Prospective evaluation of GeneXpert for the diagnosis of HIV- negative pediatric TB cases.* BMC infectious diseases, 2015. **15**: p. 70-70.
16. Moussa, H.S., F.S. Bayoumi, and A.M.A. Mohamed, *Gene Xpert for direct detection of Mycobacterium tuberculosis in stool specimens from children with presumptive pulmonary tuberculosis.* Annals of Clinical & Laboratory Science, 2016. **46**(2): p. 198-203.
17. Myo, K., et al., *Evaluation of Xpert® MTB/RIF assay as a diagnostic test for pulmonary tuberculosis in children in Myanmar.* The International Journal of Tuberculosis and Lung Disease, 2018. **22**(9): p. 1051-1055.
18. Nicol, M.P., et al., *Xpert MTB/RIF testing of stool samples for the diagnosis of pulmonary tuberculosis in children.* Clinical infectious diseases : an official publication of the Infectious Diseases Society of America, 2013. **57**(3): p. e18-e21.
19. Nicol, M.P., et al., *Microbiological diagnosis of pulmonary tuberculosis in children by oral swab polymerase chain reaction.* Scientific reports, 2019. **9**(1): p. 10789-10789.

20. Reither, K., et al., *Xpert MTB/RIF assay for diagnosis of pulmonary tuberculosis in children: A prospective, multi-centre evaluation.* Journal of Infection, 2015. **70**(4): p. 392-399.
21. Sabi, I., et al., *Pulmonary TB bacteriologically confirmed by induced sputum among children at Bugando Medical Centre, Tanzania.* The International Journal of Tuberculosis and Lung Disease, 2016. **20**(2): p. 228-234.
22. Sorsa, A., et al., *Use of Xpert Contributes to Accurate Diagnosis, Timely Initiation, and Rational Use of Anti-TB Treatment Among Childhood Tuberculosis Cases in South Central Ethiopia.* Pediatric health, medicine and therapeutics, 2020. **11**: p. 153-160.
23. Zar, H.J., et al., *Rapid diagnosis of pulmonary tuberculosis in African children in a primary care setting by use of Xpert MTB/RIF on respiratory specimens: a prospective study.* The Lancet Global Health, 2013. **1**(2): p. e97-e104.
24. Pearce, E.C., et al., *A systematic review of clinical diagnostic systems used in the diagnosis of tuberculosis in children.* AIDS research and treatment, 2012. **2012**: p. 401896-401896.
25. van Beekhuizen, H.J., *Tuberculosis Score Chart in Children in Aitape, Papua New Guinea.* Tropical Doctor, 1998. **28**(3): p. 155-160.
26. Marais, B.J., et al., *A Refined Symptom-Based Approach to Diagnose Pulmonary Tuberculosis in Children.* Pediatrics, 2006. **118**(5): p. e1350-e1359.
27. Beneri, C.A., et al., *Understanding NIH clinical case definitions for pediatric intrathoracic TB by applying them to a clinical trial.* The international journal of tuberculosis and lung disease : the official journal of the International Union against Tuberculosis and Lung Disease, 2016. **20**(1): p. 93-100.
28. Gunasekera, K.S., et al., *Development of a Treatment-decision Algorithm for Human Immunodeficiency Virus–uninfected Children Evaluated for Pulmonary Tuberculosis.* Clinical Infectious Diseases, 2021.
29. Detjen, A.K., et al., *Xpert MTB/RIF assay for the diagnosis of pulmonary tuberculosis in children: a systematic review and meta-analysis.* The Lancet. Respiratory medicine, 2015. **3**(6): p. 451-461.
30. MacLean, E., et al., *Diagnostic Accuracy of Stool Xpert MTB/RIF for Detection of Pulmonary Tuberculosis in Children: a Systematic Review and Meta-analysis.* Journal of clinical microbiology, 2019. **57**(6): p. e02057-18.
31. Mesman, A.W., et al., *Diagnostic accuracy of molecular detection of Mycobacterium tuberculosis in pediatric stool samples: A systematic review and meta-analysis.* Tuberculosis (Edinburgh, Scotland), 2019. **119**: p. 101878-101878.
32. Fekadu, L., et al., *Increasing Access to Tuberculosis Services in Ethiopia: Findings From a Patient-Pathway Analysis.* The Journal of infectious diseases, 2017. **216**(suppl_7): p. S696-S701.
33. Surya, A., et al., *Quality Tuberculosis Care in Indonesia: Using Patient Pathway Analysis to Optimize Public-Private Collaboration.* The Journal of infectious diseases, 2017. **216**(suppl_7): p. S724-S732.
