# Supplementary material for: Xpert Ultra stool testing to diagnose tuberculosis in children in Ethiopia and Indonesia: a model-based cost-effectiveness analysis
Source: BMJ Open. 2022 Jul 1;12(7):e058388. doi: 10.1136/bmjopen-2021-058388 (PMC9252203; doi:10.1136/bmjopen-2021-058388)
Supplement: Supplementary data [file bmjopen-2021-058388supp001.pdf]

Xpert Ultra stool testing to diagnose tuberculosis in children in Ethiopia and Indonesia: a model-based cost-effectiveness analysis.

APPENDIX 1: Literature search

To inform the model parameters presented in Appendix 2a, we extracted data from systematic reviews and from papers identified through an extensive targeted systematic literature search. This information was supplemented with information from papers identified from the authors' personal databases where relevant.

Data collection from published peer-reviewed systematic-reviews

We identified relevant systematic reviews and meta-analyses on TB in children in PubMed (www.pubmed.ncbi.nlm.nih.gov). Search details are provided in Box A1.1.

Box A1. Search strategy for systematic reviews

Searched in Pubmed for "systematic review meta-analysis tuberculosis children", which is interpreted by the search engine as:

(("systematic review"[Publication Type] OR "systematic reviews as topic"[MeSH Terms]) OR "systematic review"[All Fields]) AND (("meta-analysis"[Publication Type] OR "meta-analysis as topic"[MeSH Terms]) OR "meta-analysis"[All Fields]) AND (((("tuberculosi"[All Fields] OR "tuberculosis"[MeSH Terms]) OR "tuberculosis"[All Fields]) OR "tuberculoses"[All Fields]) OR "tuberculosis s"[All Fields]) AND (((((("child"[MeSH Terms] OR "child"[All Fields]) OR "children"[All Fields]) OR "child s"[All Fields]) OR "children s"[All Fields]) OR "childrens"[All Fields]) OR "childs"[All Fields])

Search date: 19 June 2020.

Of the 150 systematic reviews identified (of which one was a duplicate paper), 23 were judged relevant for full-text review (Figure A1.1). Of the 22 papers reviewed in full-text, four papers contained information about the accuracy of relevant microbiological tests for TB (1-4). However, one of these did not present meta-analytical estimates of the sensitivity and specificity of the test (Xpert Ultra, in this paper) for children (4). Two other papers presented data on the same subject and included roughly the same original work (2, 3), while for one of these, the pooled estimates presented were difficult to interpret as no comparison against culture or Xpert only was included (3). Thus, two papers provided relevant data for extraction (Figure 1): Detjen et al. (1) reported meta-analytic estimates of the sensitivity and specificity of sputum smear microscopy and Xpert on sputum, gastric lavage and nasopharyngeal aspirates (here summarized as 'respiratory samples') against culture of a respiratory sample. Mesman and colleagues (3) presented meta-analytic estimates of the sensitivity and specificity of Xpert stool testing against different reference standards (culture or Xpert on a respiratory sample, bacteriologically confirmed TB, and clinically diagnosed unconfirmed TB).

Mafirakureva N, *et al. BMJ Open* 2022; 12:e058388. doi: 10.1136/bmjopen-2021-058388

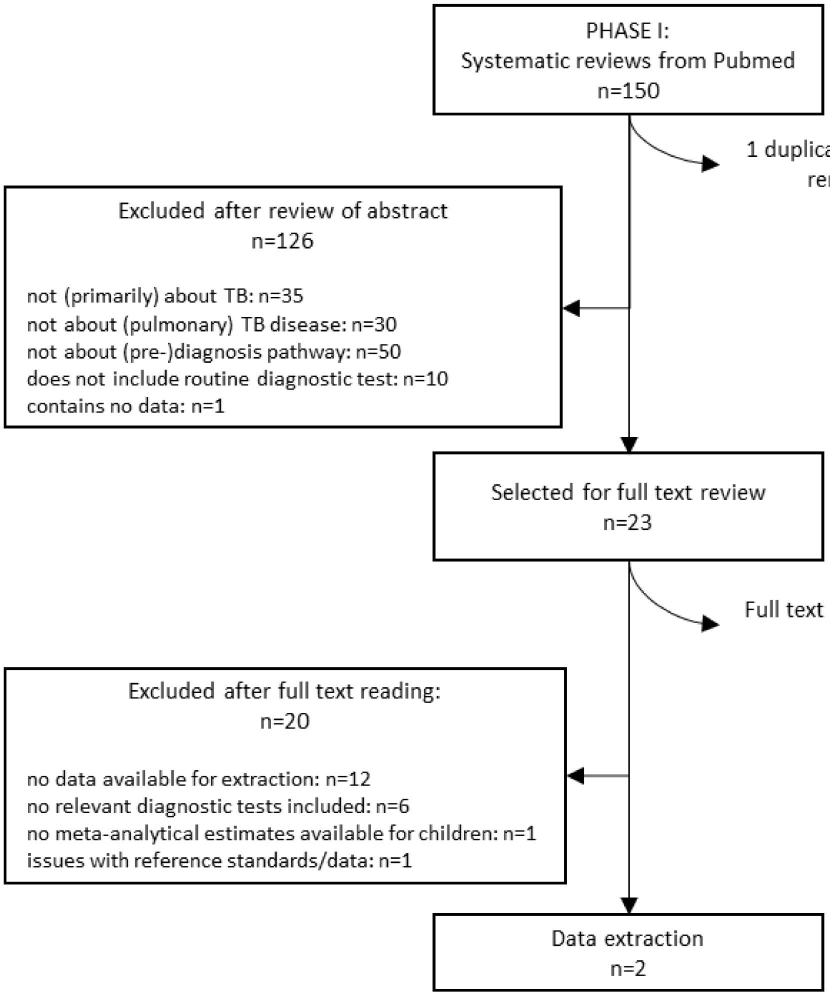

Figure A1. Prisma flow diagram for a search including published peer-reviewed systematic reviews and meta-analysis summarizing literature on TB in children.

Data collection from published peer-reviewed original papers

Next, we conducted a literature search to identify studies about TB diagnostic testing in infants and children, including health care seeking and health care cascade, with a focus on Ethiopia and Indonesia. A systematic search strategy was developed with assistance of an academic librarian from the University of Sheffield. The search strategy used a combination of free-text and thesaurus searching (where available) as outlined in Table A1. Papers with terms for Bacillus Calmette Guérin (BCG), latent tuberculosis, interferon-gamma release assay (IGRA) and tuberculin skin test in titles were excluded from the search as they are relevant to TB infection, but not to active pulmonary TB and were therefore deemed to retrieve irrelevant results. Case reports were excluded as these do not provide generalizable data. The searches were limited to English Language and Human studies published from 2010 - present where databases allowed, except for searches specifically addressing Indonesia/Ethiopia to which no such limits were applied (Table A1). Searches were conducted between 19 and 26 October 2020. Further details of the search strategy are provided in Box A1.2.

Mafirakureva N, *et al. BMJ Open* 2022; 12:e058388. doi: 10.1136/bmjopen-2021-058388

Table A1. Overview of search terms used for searching peer-reviewed original papers.

**Exploded MeSH/lookup term**	**Occurring in title**	**Occurring in title or abstract**[1]
The following were combined using 'AND':		
Tuberculosis or Diagnosis	tuberculosis or TB	
Child or Infant		child or infan[2]
Sputum or Feces		(sputum or stool or f?eces) and (test or sample or specimen)
		test* or diagnos* or screen*
Indonesia or Ethiopia or Developing Countries		Indonesia or Ethiopia or Africa or Asia or West Indies or specific countries[3]
The following were combined with the previous using 'NOT':		
case reports[4]		case report
	bacilli Calmette-Guerin or BCG	
	latent tuberculosis or LTBI or Interferon Gamma Release Assay or IGRA or tuberculin skin test or TST	

[1] In the Cochrane library, key word searches were also included here for all terms, except for the regions and countries; in Medline, this was done only for the terms *child* and *infan*; [2] In Cochrane and Science Citation Index via Web of Science, these terms were replaced with infant*; [3] Specific countries included: Angola, Bangladesh, Benin, Bolivia, Burkina Faso, Burkina Fasso, Burundi, Cambodia, Central African Republic, Chad, Congo, Cote d'Ivoire, Ivory Coast, Djibouti, Egypt, Eritrea, Gambia, Ghana, Guatemala, Guinea, India, Kenya, Korea, Lao PDR, Lesotho, Liberia, Madagascar, Malawi, Mali, Mauritania, Mozambique, Myanmar, Myanma, Nepal, Niger, Nigeria, Philippines, Philipines, Phillipines, Phillippines, Rwanda, Ruanda, Sao Tome, Senegal, Sri Lanka, Somalia, Sudan, Swaziland, Tanzania, Timor-Leste, Togo, Uganda, Vietnam, Viet Nam, Zambia, Zimbabwe; searches for other countries than Ethiopia and Indonesia were limited to English language and humans and *yr="2010 -Current"*; [4] Only included if this lookup term existed for the system (see box 2 for specifications).

In total, 2,974 unique titles were available for title screening, from which 770 were selected for abstract screening. Subsequently, we selected 260 papers for full-text review, of which, after review, data were extracted from 73 (Figure A1.2). The extracted information from these 73 papers was reviewed by the modeling team for its usefulness and applicability to inform the model. Finally, the extracted data for 21 papers was judged to be directly relevant to inform the model. Table A2 provides an overview of all 73 papers for which data was extracted, and specifies which papers were used to inform the model.

Mafirakureva N, *et al. BMJ Open* 2022; 12:e058388. doi: 10.1136/bmjopen-2021-058388

Box A2. Details of search strategy for peer-reviewed original publications

- Developed in MEDLINE
- studies about TB diagnostic testing in infants and children
- two sets of search results:
  - Indonesia or Ethiopia
  - other countries in Africa or Asia (adapting the Cochrane LMIC filter (https://epoc.cochrane.org/lmic-filters).
- Searches were further specified following examination of 100 references in pilot search, excluding terms for BCG, latent tuberculosis, IGRA and tuberculin skin test appearing in titles
- Case reports were excluded where possible

**Database**	**Date Searched**	**Number of References Retrieved (including duplicates)**	**Total N retrieved (including duplicates)**
Ovid MEDLINE(R)<br>1946 to Oct Week 3 2020	23/10/20	Indonesia & Ethiopia = 180<br>Other countries = 1,198	**Indonesia & Ethiopia = 537**<br><br>**Other countries = 4,348**
Ovid MEDLINE(R) and Epub Ahead of Print, In-Process & Other Non-Indexed Citations and Daily<br>2016 to Oct 22, 2020*	23/10/20	Indonesia & Ethiopia = 97<br>Other countries = 790	
Ovid Embase<br>1974 to Oct 23, 2020**	26/10/20	Indonesia & Ethiopia = 237<br>Other countries = 1,783	
Cochrane Database of Systematic Reviews<br>Issue 10 of 12, Oct 2020	19/10/20	Indonesia & Ethiopia = 2<br>Other countries = 11 (plus one protocol)	
Cochrane Central Register of Controlled Trials<br>Issue 10 of 12, Oct 2020	19/10/20	Indonesia & Ethiopia = 4<br>Other countries = 108	
Science Citation Index via Web of Science<br>1900-present	26/10/20<br>19/10/20	Indonesia & Ethiopia = 17<br>Other countries = 457	
Conference Proceedings Citation Index-Science (CPCI-S) via Web of Science<br>1990-present	26/10/20	Indonesia & Ethiopia = 1<br>Other countries = 2	

** English Language and Human limits removed as do not work correctly in MEDLINE In-Process; ** case report(s) not a publication type in Embase*

Mafirakureva N, *et al. BMJ Open* 2022; 12:e058388. doi: 10.1136/bmjopen-2021-058388

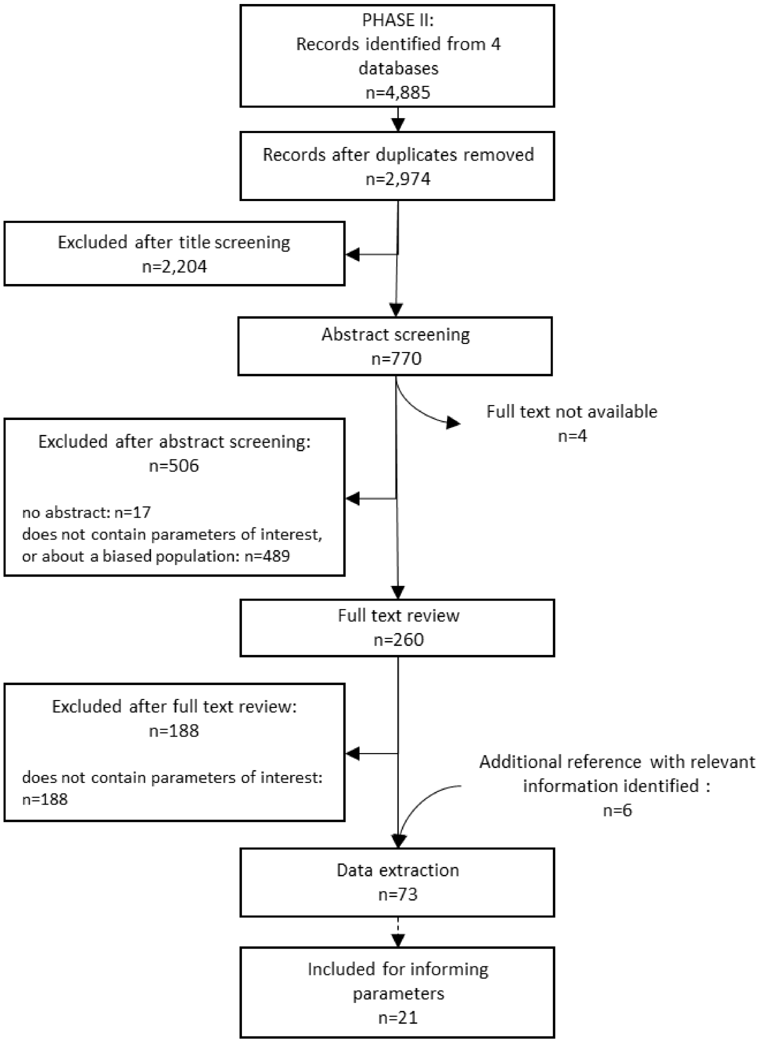

Figure A2. Prisma flow diagram for systematic literature review on TB diagnostic testing in infants and children.

Mafirakureva N, *et al. BMJ Open* 2022; 12:e058388. doi: 10.1136/bmjopen-2021-058388

Table A2. Overview of papers from which data was extracted in the comprehensive literature review of phase 2 (see Figure 2 for details).

Reference (first author and year of publication)	Country	Child population included (N)	Age range	Health care level (no. centers)	Data extracted	Model parameters informed	Comment (e.g., reason for not being considered for informing model parameters)
Andriyoko, 2019 (5)	Indonesia	lab-based, stool plus sputum/NGA submitted for TB diagnosis (36)	0-15y	level 3 (1)	% of presumptive TB cases with confirmed TB		unclear how study population was composed (laboratory study)
Ardizzoni, 2015 (6)	multiple	register review of samples with Xpert results (1,278)	0-14y	NA	% of presumptive TB cases with confirmed TB		Indonesia nor Ethiopia included; data shown on all ages but data extracted for children and (induced) sputum/NGA only
Assefa, 2015 (7)	Ethiopia	household contacts of SS+TB patients (230)	0-5y	level 1 (27)	% of child population seeking care		biased population (semi-active case finding)
Atwebembeire, 2016 (8)	Uganda	lab-based, string test and induced sputum samples (88)	NS	level 3 (1)	% of samples MTB-positive		No information on final TB diagnosis
Bacha, 2017 (9)	Tanzania	presumptive TB or referred for TB treatment (455)	0-14y	level 2/3 (1)	% of presumptive TB cases with confirmed, probable and possible TB	*p_truetb*	
Banada, 2016 (10)	South Africa	consecutive confirmed (20) and probable (20) TB cases	0-14y	level1 (NS) and 2 (NS)	% of samples MTB-positive, by type of sample		Only includes diagnosed TB patients
Bates, 2013 (11)	Zambia	primary or secondary admission diagnosis of TB (930)	0-15y	level 3 (1)	% of presumptive TB cases submitting respiratory specimen for TB diagnosis, by type of specimen and age group<br>% of children with true TB	*spont.sput*	
van Beekhuizen, 1998* (12)	Papua New Guinea	admitted for malnutrition, recurrent pneumonia, or signs/symptoms of TB (301)	0-16y	level 1 (1)	Sensitivity and specificity of clinical diagnosis		Evaluated the sensitivity and specificity of a TB score chart instead of pediatrician's diagnosis
Beneri, 2016 (13)	South Africa	presumptive TB in RCT on TPT for HIV-exposed and -infected (219)	<5y	NA	% of presumptive TB cases with confirmed, probable and possible TB; Sensitivity and specificity of clinical diagnosis		considered for informing *sens.clin* and *spec.clin*, but not a representative population (semi-active case finding)
Berggren-Palme, 2004 (14)	Ethiopia	clinically diagnosed with TB (355)	0-14y	level 3 (1)	% of TB cases submitting spontaneously expectorated sputum for TB diagnosis		only diagnosed TB patients included
Binua, 2019 (15)	Philippines	presumptive TB (incl. EPTB) (112)	4-18y	level 3 (1)	% of presumptive TB cases with definite (smear-positive) TB		abstract only, no detailed information; EPTB included

Mafirakureva N, *et al. BMJ Open* 2022; 12:e058388. doi: 10.1136/bmjopen-2021-058388

Reference (first author and year of publication)	Country	Child population included (N)	Age range	Health care level (no. centers)	Data extracted	Model parameters informed	Comment (e.g., reason for not being considered for informing model parameters)
Bojang, 2016 (16)	The Gambia	presumptive TB (24)	0-14y	level 1 (NS) & research unit (1)	% of presumptive TB cases with definite (Xpert) TB		No information on final TB diagnosis
Brent, 2017 (17)	Kenya	presumptive TB (1,442)	0-14y	level 2 (2)	% of presumptive TB cases with confirmed, highly probable and possible TB		did not use standard clinical case definitions, EPTB included which cannot be separated from pulmonary TB
Bunyasi, 2015 (18)	South Africa	investigated for incident TB in vaccine trial (active & passive FU) (1,020)	<4y	NA	% of presumptive TB cases with confirmed TB		No information on final TB diagnosis; non-representative population
Chipinduro, 2017 (19)	Zimbabwe	presumptive TB (221)	5-16y	level 1 (8)	% of presumptive TB cases submitting induced sputum for TB diagnosis		no data on % spontaneously expectorating sputum
Chisti, 2013 (20)	multiple	acute pneumonia with SAM and/or HIV infection (747)	<5y	NS (NS)	% of children with acute pneumonia being diagnosed with confirmed TB		population not representative for children with presumptive TB
Das, 2019 (21)	India	presumptive TB, partly admitted (171)	0-14y	level 3 (1)	% of presumptive TB cases with confirmed (smear/Xpert-positive) TB		no data on % clinically diagnosed with pulmonary TB
Dayal, 2020 (22)	India	diagnosed with probable TB (114)	0-13y	level 3 (1)	% of samples MTB-positive, by type of sample		Only includes diagnosed TB patients
Elhassan, 2016 (23)	Sudan	presumptive TB (197)	0-13y	level 1 (5)	% of presumptive TB cases with confirmed, probable and possible TB	*p_truetb*	
Eliso, 2015 (24)	Ethiopia	cough of any duration (43)	6-15y	level 1 (4)	% of presumptive TB cases with definite (smear-positive) TB		only smear-positive TB included
Fekadu, 2017* (25)	Ethiopia	NA	NA	level 1 (NA)	% of children seeking care at level 0/1 health facilities first	*phc0_e*	this study used data from multiple sources to estimate TB care cascade
Garcia-Basteiro, 2015 (26)	Mozambique	presumptive TB (766)	0-2y	NA (Research Center) (1)	% of presumptive TB cases with definite and probable TB		possible TB not presented in the paper, limited age bands
Giang, 2015 (27)	Vietnam	presumptive TB (150)	0-14y	level 3 (1)	% of presumptive TB cases with confirmed, probable and possible TB	*p_truetb*	
Gous, 2015 (28)	South Africa	presumptive TB (484)	0-14y	level 2 (1)	% of samples MTB-positive, by method		No information on final TB diagnosis
Hanrahan, 2019 (29)	South Africa	presumptive TB (119)	2m-10y	level 1 (1, high-volume)	% of presumptive TB cases submitting respiratory specimen for TB diagnosis, by type of specimen and age group	*spont.sput, p_truetb*	

Mafirakureva N, *et al. BMJ Open* 2022; 12:e058388. doi: 10.1136/bmjopen-2021-058388

Reference (first author and year of publication)	Country	Child population included (N)	Age range	Health care level (no. centers)	Data extracted	Model parameters informed	Comment (e.g., reason for not being considered for informing model parameters)
Kabir, 2018 (30)	Bangladesh	clinically diagnosed with TB (102)	0-14y	level 3 (1)	% of samples MTB-positive, by method		No information on final TB diagnosis
Kabir, 2020 (31)	Bangladesh	presumptive TB (448)	0-14y	level 3 (1)	% of presumptive TB cases submitting induced sputum and/or stool for TB diagnosis		no data on % spontaneously expectorating sputum
Kalra, 2020 (32)	India	presumptive TB (94,415)	0-14y	level 3 (1)	% of presumptive TB cases submitting any specimen for TB diagnosis, by type of specimen		no data on % spontaneously expectorating sputum
Kalu, 2013 (33)	Nigeria	presumptive TB (263)	3m-14y	level 3 (1)	% of presumptive TB cases with confirmed (culture-positive and/or smear-positive) TB		no data on % clinically diagnosed with pulmonary TB
Lopez-Varela, 2015** (34)	Mozambique	presumptive TB (789)	0-2y	NS (Research Center) (1)	% of presumptive TB cases with definite, probable and possible TB		limited age bands
Marais, 2006* (35)	South Africa	cough>2 weeks without response to oral antibiotics course (428)	0-12y	level 1 (5)	Sensitivity and specificity of clinical diagnosis	*sens.clin, spec.clin*	
Moussa, 2016 (36)	Egypt	presumptive TB (115)	0-15y	level 3 (1)	% of presumptive TB cases with confirmed, probable and possible TB	*p_truetb*	
Mukherjee, 2013 (37)	India	clinically diagnosed intrathoracic TB (403)	6m-15y	level 3 (2)	% of bacteriologically confirmed TB		only includes diagnosed TB patients
Mulenga, 2011 (38)	South Africa	two cohorts investigated for incident TB in two vaccine trials (active & passive FU) (1,445+740)	0-2y?	NA	% of child population seeking care<br><br>Sensitivity and specificity of clinical diagnosis		mixture of PHC and hospital care seeking, limited age bands;<br>contains % with different combinations of symptoms and signs of TB, but no data for parameter of interest
Mulenga, 2015 (39)	South Africa	investigated for incident TB in vaccine trial (active FU) (1,017)	0-2y	NA	Sensitivity and specificity of clinical diagnosis		contains % with different combinations of symptoms and signs of TB, but no data for parameter of interest
Munoz-Sellart, 2009 (40)	Ethiopia	diagnosed with TB (231)	0-14y	level 1 (7) and 2 (1)	% of smear-positive TB		only includes diagnosed TB patients
Mwangwa, 2017 (41)	Uganda	diagnosed with TB in HIV RCT (42)	0-15y	level 1 (32 communities)	% of bacteriologically confirmed TB cases started on treatment		likely higher than SOC
Myo, 2018 (42)	Myanmar	presumptive TB (231)	1m-12y	level 3 (1)	% of presumptive TB cases with confirmed and unconfirmed TB<br>% of bacteriologically confirmed TB cases started on treatment	*p_truetb*	 <br>likely higher than SOC

Mafirakureva N, *et al. BMJ Open* 2022; 12:e058388. doi: 10.1136/bmjopen-2021-058388

Reference (first author and year of publication)	Country	Child population included (N)	Age range	Health care level (no. centers)	Data extracted	Model parameters informed	Comment (e.g., reason for not being considered for informing model parameters)
Nansumba, 2016 (43)	Uganda	presumptive TB (137)	3-14y	level 3 (1)	% of presumptive TB cases submitting respiratory specimen for TB diagnosis;<br>% of presumptive TB cases with confirmed (culture-positive) TB;<br>% of bacteriologically confirmed TB cases started on treatment		no data on % spontaneously expectorating sputum;<br>no data on clinically diagnosed TB;<br>likely higher than SOC
Negash, 2020 (44)	Ethiopia	lab-based, any sputum received for TB diagnosis (414)	4-14y	level 1 (4 hospitals, 34 HCs)	% of presumptive TB cases with Xpert-positive TB		no data on clinically diagnosed TB
Nhu, 2013 (45)	Vietnam	presumptive TB (73)	0-15y	level 3 (1)	% of bacteriologically confirmed TB		only includes diagnosed TB patients
Nicol, 2011 (46)	South Africa	admitted for presumptive TB (452)	0-15y	level 3 (2)	% of bacteriologically confirmed TB;<br>% of bacteriologically confirmed TB cases started on treatment	*Fbc*	only includes in-patients in level-3 hospital<br>likely higher than SOC
Nicol, 2013 (47)	South Africa	presumptive TB (115)	0-14y	level 1 (1) and level 3 (1)	% of presumptive TB cases with confirmed, probable and possible TB	*p_truetb*	none of the children was diagnosed with possible TB
Nicol, 2019 (48)	South Africa	presumptive TB (165)	0-14y	level 3 (1)	% of presumptive TB cases with confirmed and unconfirmed TB;<br>% of bacteriologically confirmed TB cases started on treatment	*p_truetb*	likely higher than SOC
Nissen, 2012 (49)	Tanzania	presumptive TB (195)	0-14y	level 1/2 (1)	% returning for clinical re-evaluation after initial exclusion of TB		likely higher than SOC as asked to return by the study team
Oliwa, 2019 (50)	Kenya	admitted for presumptive TB (23,741)	0-15y	level 2 (13)	% of presumptive TB cases that gets TST, chest X-ray, and bacteriology offered		not regarded sufficiently representative for Ethiopia and Indonesia
Orikiriza, 2018 (51)	Uganda	presumptive TB, partly admitted (392)	1m-14y	level 2/3 (1)	% of presumptive TB cases with confirmed TB, % started on TB treatment		case definitions provided in Methods section were not used to present results
Pearce, 2012* (52)	NA	NA	NA	NA	Sensitivity and specificity of clinical diagnosis		review, no original data; only one study identified providing a sensitivity score
Ramos, 2013 (53)	Ethiopia	retrospective review of sputum reports (875)	0-14y	level 2 (1)	% of presumptive TB cases with smear-positive TB		only smear-positive TB included
Ramos, 2019 (54)	Ethiopia	diagnosed with smear-positive TB (862)	0-14y	level 2 (1)	% of TB patients diagnosed with smear-positive TB		only diagnosed TB patients included

Mafirakureva N, *et al. BMJ Open* 2022; 12:e058388. doi: 10.1136/bmjopen-2021-058388

Reference (first author and year of publication)	Country	Child population included (N)	Age range	Health care level (no. centers)	Data extracted	Model parameters informed	Comment (e.g., reason for not being considered for informing model parameters)
Raizada, 2014 (55)	India	presumptive pulmonary TB (4,600)	0-14y	network of level 1 HF (±400), microscopy centers (99), and sub-district TB units (18)	% of presumptive TB cases with bacteriologically confirmed (by SSM/ Xpert) TB		no data on clinically diagnosed TB
Raizada, 2015 (56)	India	presumptive pulmonary TB (517)	0-14y	as Razaida, 2014 (55)	% of presumptive TB cases with bacteriologically confirmed (by SSM/ Xpert) TB		no data on clinically diagnosed TB
Raizada, 2018a (57)	India	lab-based study, presumptive TB (3,045)	0-14y	central Xpert labs (4) receiving samples from all levels (public & private) in 4 big cities	% of bacteriologically confirmed TB cases started on treatment		also EPTB included, no differentiation by type of TB possible
Raizada, 2018b (58)	India	lab-based study, presumptive TB (465)	<2y	as Razaida, 2018a (57)	% of bacteriologically confirmed TB cases started on treatment		also EPTB included, no differentiation by type of TB possible
Reither, 2015 (59)	Uganda, Tanzania	presumptive TB (451)	2m-15y	NA (Research Center) (2), level 2 (1)	% of presumptive TB cases with confirmed, highly probable and probable TB	*p_truetb*	
Sabi, 2016 (60)	Tanzania	presumptive TB (192)	2m-12y	level 2 (1)	% of presumptive TB cases submitting respiratory specimen for TB diagnosis;<br>% of presumptive TB cases with confirmed, probable and possible TB	*p_truetb*	no data on % spontaneously expectorating sputum
Sabi, 2018 (61)	Tanzania	presumptive TB (277)	6m-16y	NS (Research Center) (2)	% of presumptive TB cases with confirmed, highly probable and probable TB		Focus of report on stored sputum samples tested with Xpert Ultra. More relevant data presented in Reither, 2015
Sekadde, 2013 (62)	Uganda	presumptive TB (235)	2m-12y	level 3 (1)	% of presumptive TB cases submitting induced sputum for TB diagnosis;<br>% of presumptive TB cases with confirmed TB		no data on % spontaneously expectorating sputum;<br>no data on clinically diagnosed TB
Sharma, 2020 (63)	India	non-expectorating with strong clinical suspicion of TB (210)	6m-12y	level 3 (1)	% of presumptive TB cases with bacteriologically confirmed TB		non-expectorating children only; no data on clinically diagnosed TB

Mafirakureva N, *et al. BMJ Open* 2022; 12:e058388. doi: 10.1136/bmjopen-2021-058388

Reference (first author and year of publication)	Country	Child population included (N)	Age range	Health care level (no. centers)	Data extracted	Model parameters informed	Comment (e.g., reason for not being considered for informing model parameters)
Shata, 1996 (64)	Malawi	presumptive TB (29)	3-15y	level 3 (1)	% of presumptive TB cases submitting induced sputum for TB diagnosis		no data on % spontaneously expectorating sputum
Singh, 2016 (65)	India	presumptive TB (50)	0-14y	NS (3)	% of presumptive TB cases with confirmed (SSM) and probable TB		no internationally accepted definition used for the clinical definition of TB
Sorsa, 2020 (66)	Ethiopia	presumptive TB (775)	0-14y	level 3 (1)	% of presumptive TB cases with confirmed (SSM/Xpert) and probable TB	*p_truetb*	
Ssengooba, 2020 (67)	Uganda	diagnosed with "minimal TB" participating in clinical trial (353)	0-15y	NS	% of samples MTB-positive, by type of sample		only diagnosed TB patients included
Surya, 2017* (68)	Indonesia	NA	NA	level 1 (NA)	% of children seeking care at level 0/1 health facilities first	*phc0_i*	this study used data from multiple sources to estimate TB care cascade
Swaminathan, 2008 (69)	India	presumptive TB (2,652)	6m-12y	level 3 (3)	% of presumptive TB cases with bacteriologically confirmed TB		no data on clinically diagnosed TB
Walters, 2017a (70)	South Africa	presumptive intrathoracic TB (188)	0-12y	level 3 (2)	% of presumptive TB cases with confirmed and unconfirmed TB		population is the same as presented in Walters, vd Zalm, 2017
Walters, 2017b (71)	South Africa	presumptive intrathoracic TB (379)	0-12y	level 3 (2)	% TB bacteriologically positive under ideal conditions<br>% of presumptive TB cases with confirmed and unconfirmed TB	*Fbc,*<br>*p_truetb*	
Walters, 2018 (72)	South Africa	presumptive TB (148)	0-15y	level 3 (2)	% of presumptive TB cases submitting stool for TB diagnosis;<br>% of presumptive TB cases with confirmed and unconfirmed TB<br>% started on TB treatment after clinical re-evaluation	*p_truetb*	no data on % spontaneously expectorating sputum<br>likely higher than SOC
Yadav, 2020 (73)	India	presumptive TB (155)	0-15y	level 3 (1)	% of presumptive TB cases submitting respiratory specimen for TB diagnosis, by type of specimen		not clear if spontaneous expectoration was attempted in all children
Zar, 2005 (74)	South Africa	admitted for presumptive TB (250)	1m-5y	level 3 (2)	% of presumptive TB cases with confirmed (SSM/culture) TB		no data on clinically diagnosed TB
Zar, 2012 (75)	South Africa	admitted for different severe conditions with presumptive TB (535)	0-14y	level 3 (2)	% TB bacteriologically positive under ideal conditions<br>% of presumptive TB cases with definite (Xpert/culture) and possible TB	*Fbc*	non-representative population (hospitalized with severe conditions)

Mafirakureva N, *et al. BMJ Open* 2022; 12:e058388. doi: 10.1136/bmjopen-2021-058388

**Reference (first author and year of publication)**	**Country**	**Child population included (N)**	**Age range**	**Health care level (no. centers)**	**Data extracted**	**Model parameters informed**	**Comment (e.g., reason for not being considered for informing model parameters)**
Zar, 2013 (76)	South Africa	presumptive TB (384)	0-14y	level 1 (1, high-volume)	% of presumptive TB cases with definite (Xpert/culture) and possible TB<br>% started on TB treatment after clinical re-evaluation	*p_truetb*	likely higher than SOC
Zar, 2019 (77)	South Africa	admitted for presumptive TB (195)	0-14y	level 3 (1)	% TB bacteriologically positive under ideal conditions<br>% of presumptive TB cases with confirmed and unconfirmed TB	*Fbc*	non-representative population (hospitalized with severe conditions)

Abbreviations used in table: EPTB: extrapulmonary tuberculosis, Fu: follow up, HC: health center, HF: healthcare facility, m: months, MTB: *Mycobacterium tuberculosis*, NA: not applicable, NS: not specified, RCT: randomized controlled trial, SOC: standard of care, SSM: sputum smear microscopy, TB: tuberculosis, y: years; * Identified from authors' personal databases, not through the systematic literature review; ** Identified as paper containing the source data of one of the papers identified in the systematic literature review (26).

Mafirakureva N, *et al. BMJ Open* 2022; 12:e058388. doi: 10.1136/bmjopen-2021-058388

References

1. Detjen AK, DiNardo AR, Leyden J, Steingart KR, Menzies D, Schiller I, Dendukuri N, Mandalakas AM. 2015. Xpert MTB/RIF assay for the diagnosis of pulmonary tuberculosis in children: a systematic review and meta-analysis. Lancet Respir Med 3:451-61.
2. MacLean E, Sulis G, Denkinger CM, Johnston JC, Pai M, Ahmad Khan F. 2019. Diagnostic Accuracy of Stool Xpert MTB/RIF for Detection of Pulmonary Tuberculosis in Children: a Systematic Review and Meta-analysis. J Clin Microbiol 57.
3. Mesman AW, Rodriguez C, Ager E, Coit J, Trevisi L, Franke MF. 2019. Diagnostic accuracy of molecular detection of Mycobacterium tuberculosis in pediatric stool samples: A systematic review and meta-analysis. Tuberculosis (Edinb) 119:101878.
4. Zhang M, Xue M, He JQ. 2020. Diagnostic accuracy of the new Xpert MTB/RIF Ultra for tuberculosis disease: A preliminary systematic review and meta-analysis. Int J Infect Dis 90:35-45.
5. Andriyoko B, Janiar H, Kusumadewi R, Klinkenberg E, de Haas P, Tiemersma E. 2019. Simple stool processing method for the diagnosis of pulmonary tuberculosis using GeneXpert MTB/RIF. European Respiratory Journal 53:03.
6. Ardizzoni E, Fajardo E, Saranchuk P, Casenghi M, Page AL, Varaine F, Kosack CS, Hepple P. 2015. Implementing the Xpert R MTB/RIF Diagnostic Test for Tuberculosis and Rifampicin Resistance: Outcomes and Lessons Learned in 18 Countries. PLoS ONE [Electronic Resource] 10:e0144656.
7. Assefa D, Klinkenberg E, Yosef G. 2015. Cross Sectional Study Evaluating Routine Contact Investigation in Addis Ababa, Ethiopia: A Missed Opportunity to Prevent Tuberculosis in Children. PLoS ONE [Electronic Resource] 10:e0129135.
8. Atwebembeire J, Orikiriza P, Bonnet M, Atwine D, Katawera V, Nansumba M, Nyehangane D, Bazira J, Mwanga-Amumpaire J, Byarugaba F, Boum Y. 2016. Xpert(R) MTB/RIF for detection of Mycobacterium tuberculosis from frozen string and induced sputum sediments. International Journal of Tuberculosis & Lung Disease 20:1113-7.
9. Bacha JM, Ngo K, Clowes P, Draper HR, Ntinginya EN, DiNardo A, Mangu C, Sabi I, Mtafya B, Mandalakas AM. 2017. Why being an expert - despite xpert -remains crucial for children in high TB burden settings. BMC Infectious Diseases 17:123.
10. Banada PP, Naidoo U, Deshpande S, Karim F, Flynn JL, O'Malley M, Jones M, Nanassy O, Jeena P, Alland D. 2016. A Novel Sample Processing Method for Rapid Detection of Tuberculosis in the Stool of Pediatric Patients Using the Xpert MTB/RIF Assay. PLoS ONE [Electronic Resource] 11:e0151980.
11. Bates M, O'Grady J, Maeurer M, Tembo J, Chilukutu L, Chabala C, Kasonde R, Mulota P, Mzyece J, Chomba M, Mukonda L, Mumba M, Kapata N, Rachow A, Clowes P, Hoelscher M, Mwaba P, Zumla A. 2013. Assessment of the Xpert MTB/RIF assay for diagnosis of tuberculosis with gastric lavage aspirates in children in sub-Saharan Africa: a prospective descriptive study. The Lancet Infectious Diseases 13:36-42.
12. van Beekhuizen HJ. 1998. Tuberculosis score chart in children in Aitape, Papua New Guinea. Trop Doct 28:155-60.
13. Beneri CA, Aaron L, Kim S, Jean-Philippe P, Madhi S, Violari A, Cotton MF, Mitchell C, Nachman S. 2016. Understanding NIH clinical case definitions for pediatric intrathoracic TB by applying them to a clinical trial. International Journal of Tuberculosis and Lung Disease 20:93-100.
14. Berggren Palme I, Gudetta B, Bruchfeld J, Eriksson M, Giesecke J. 2004. Detection of Mycobacterium tuberculosis in gastric aspirate and sputum collected from Ethiopian HIV-positive and HIV-negative children in a mixed in- and outpatient setting. Acta Paediatrica 93:311-5.
15. Binua F, Tuazon A. 2019. The yield of AFB smear and tb culture in the diagnosis of childhood tb using sputum induction with n-acetylcysteine: a randomized controlled trial. Pediatric pulmonology 54:S79-.
16. Bojang AL, Mendy FS, Tientcheu LD, Otu J, Antonio M, Kampmann B, Agbla S, Sutherland JS. 2016. Comparison of TB-LAMP, GeneXpert MTB/RIF and culture for diagnosis of pulmonary tuberculosis in The Gambia. Journal of Infection 72:332-7.
17. Brent AJ, Mugo D, Musyimi R, Mutiso A, Morpeth SC, Levin M, Scott JAG. 2017. Bacteriological diagnosis of childhood TB: a prospective observational study. Scientific Reports 7:11808.
18. Bunyasi EW, Tameris M, Geldenhuys H, Schmidt BM, Luabeya AK, Mulenga H, Scriba TJ, Hanekom WA, Mahomed H, McShane H, Hatherill M. 2015. Evaluation of Xpert R MTB/RIF Assay in Induced Sputum and Gastric Lavage Samples from Young Children with Suspected Tuberculosis from the MVA85A TB Vaccine Trial. PLoS ONE [Electronic Resource] 10:e0141623.

Mafirakureva N, *et al. BMJ Open* 2022; 12:e058388. doi: 10.1136/bmjopen-2021-058388

19. Chipinduro M, Mateveke K, Makamure B, Ferrand RA, Gomo E. 2017. Stool Xpert^R MTB/RIF test for the diagnosis of childhood pulmonary tuberculosis at primary clinics in Zimbabwe. International Journal of Tuberculosis & Lung Disease 21:161-166.
20. Chisti MJ, Ahmed T, Pietroni MA, Faruque AS, Ashraf H, Bardhan PK, Hossain I, Das SK, Salam MA. 2013. Pulmonary tuberculosis in severely-malnourished or HIV-infected children with pneumonia: a review. Journal of Health, Population & Nutrition 31:308-13.
21. Das A, Anupurba S, Mishra OP, Banerjee T, Tripathi R. 2019. Evaluation of Xpert MTB/RIF Assay for Diagnosis of Tuberculosis in Children. Journal of Tropical Pediatrics 65:14-20.
22. Dayal R, Yadav A, Agarwal D, Kumar M, Kamal R, Singh D, Bhatnagar S. 2020. Comparison of Diagnostic Yield of Tuberculosis Loop-Mediated Isothermal Amplification Assay With Cartridge-Based Nucleic Acid Amplification Test, Acid-Fast Bacilli Microscopy, and Mycobacteria Growth Indicator Tube Culture in Children With Pulmonary Tuberculosis. Journal of the Pediatric Infectious Diseases Societ 10:10.
23. Elhassan MM, Elmekki MA, Osman AL, Hamid ME. 2016. Challenges in diagnosing tuberculosis in children: a comparative study from Sudan. International Journal of Infectious Diseases 43:25-29.
24. Eliso E, Medhin G, Belay M. 2015. Prevalence of smear positive pulmonary tuberculosis among outpatients presenting with cough of any duration in Shashogo Woreda, Southern Ethiopia. BMC Public Health 15:112.
25. Fekadu L, Hanson C, Osberg M, Makayova J, Mingkwan P, Chin D. 2017. Increasing Access to Tuberculosis Services in Ethiopia: Findings From a Patient-Pathway Analysis. J Infect Dis 216:S696-S701.
26. Garcia-Basteiro AL, Lopez-Varela E, Augusto OJ, Gondo K, Munoz J, Sacarlal J, Marais B, Alonso PL, Ribo JL. 2015. Radiological findings in young children investigated for tuberculosis in Mozambique. PLoS ONE [Electronic Resource] 10:e0127323.
27. Giang do C, Duong TN, Ha DT, Nhan HT, Wolbers M, Nhu NT, Heemskerk D, Quang ND, Phuong DT, Hang PT, Loc TH, Lan NT, Dung NH, Farrar J, Caws M. 2015. Prospective evaluation of GeneXpert for the diagnosis of HIV- negative pediatric TB cases. BMC Infectious Diseases 15:70.
28. Gous N, Scott LE, Khan S, Reubenson G, Coovadia A, Stevens W. 2015. Diagnosing childhood pulmonary tuberculosis using a single sputum specimen on Xpert MTB/RIF at point of care. South African Medical Journal Suid-Afrikaanse Tydskrif Vir Geneeskunde 105:1044-8.
29. Hanrahan CF, Dansey H, Mutunga L, France H, Omar SV, Ismail N, Bassett J, Van Rie A. 2019. Diagnostic strategies for childhood tuberculosis in the context of primary care in a high burden setting: the value of alternative sampling methods. Paediatrics & international Child Health 39:88-94.
30. Kabir S, Uddin MKM, Chisti MJ, Fannana T, Haque ME, Uddin MR, Banu S, Ahmed T. 2018. Role of PCR method using IS6110 primer in detecting Mycobacterium tuberculosis among the clinically diagnosed childhood tuberculosis patients at an urban hospital in Dhaka, Bangladesh. International Journal of Infectious Diseases 68:108-114.
31. Kabir S, Rahman SMM, Ahmed S, Islam MS, Banu RS, Shewade HD, Thekkur P, Anwar S, Banu NA, Nasrin R, Uddin MKM, Choudhury S, Ahmed S, Paul KK, Khatun R, Chisti MJ, Banu S. 2020. Xpert Ultra assay on stool to diagnose pulmonary tuberculosis in children. Clinical Infectious Diseases 18:18.
32. Kalra A, Parija D, Raizada N, Sachdeva KS, Rao R, Swaminathan S, Khanna A, Chopra KK, Hanif M, Singh V, Umadevi KR, Sheladia KN, Rao R, Vasundhara N, S A, A RN, Azeem A, Chhajlani V, Khurana J, Das NJ, Choudhury B, Nair SA, Mall S, Sen R, Chadha SS, Denkinger CM, Boehme C, Sarin S. 2020. Upfront Xpert MTB/RIF for diagnosis of pediatric TB-Does it work? Experience from India. PLoS ONE [Electronic Resource] 15:e0236057.
33. Kalu EI, Ojide CK, Ugochukwu NV. 2013. Gastric aspirate smear microscopy as a diagnostic tool for childhood pulmonary tuberculosis. Annals of Tropical Medicine and Public Health 6:608-613.
34. Lopez-Varela E, Augusto OJ, Gondo K, Garcia-Basteiro AL, Fraile O, Ira T, Ribo Aristizabal JL, Bulo H, Munoz Gutierrez J, Aponte J, Macete E, Sacarlal J, Alonso PL. 2015. Incidence of Tuberculosis Among Young Children in Rural Mozambique. Pediatric Infectious Disease Journal 34:686-92.
35. Marais BJ, Gie RP, Hesseling AC, Schaaf HS, Lombard C, Enarson DA, Beyers N. 2006. A refined symptom-based approach to diagnose pulmonary tuberculosis in children. Pediatrics 118:E1350-E1359.
36. Moussa H, Bayoumi FS, Mohamed AM. 2016. Gene Xpert for Direct Detection of Mycobacterium Tuberculosis in Stool Specimens from Children with Presumptive Pulmonary Tuberculosis. Annals of Clinical & Laboratory Science 46:198-203.

Mafirakureva N, *et al. BMJ Open* 2022; 12:e058388. doi: 10.1136/bmjopen-2021-058388

37. Mukherjee A, Singh S, Lodha R, Singh V, Hesseling AC, Grewal HM, Kabra SK, Delhi Pediatric TBSG. 2013. Ambulatory gastric lavages provide better yields of Mycobacterium tuberculosis than induced sputum in children with intrathoracic tuberculosis. Pediatric Infectious Disease Journal 32:1313-7.
38. Mulenga H, Moyo S, Workman L, Hawkridge T, Verver S, Tameris M, Geldenhuys H, Hanekom W, Mahomed H, Hussey G, Hatherill M. 2011. Phenotypic variability in childhood TB: implications for diagnostic endpoints in tuberculosis vaccine trials. Vaccine 29:4316-21.
39. Mulenga H, Tameris MD, Luabeya KKA, Geldenhuys H, Scriba TJ, Hussey GD, Mahomed H, Landry BS, Hanekom WA, McShane H, Hatherill M. 2015. The role of clinical symptoms in the diagnosis of intrathoracic tuberculosis in young children. Pediatric Infectious Disease Journal 34:1157-1162.
40. Munoz-Sellart M, Yassin MA, Tumato M, Merid Y, Cuevas LE. 2009. Treatment outcome in children with tuberculosis in southern Ethiopia. Scandinavian Journal of Infectious Diseases 41:450-5.
41. Mwangwa F, Chamie G, Kwarisiima D, Ayieko J, Owaraganise A, Ruel TD, Plenty A, Tram KH, Clark TD, Cohen CR, Bukusi EA, Petersen M, Kamya MR, Charlebois ED, Havlir DV, Marquez C. 2017. Gaps in the Child Tuberculosis Care Cascade in 32 Rural Communities in Uganda and Kenya. Journal of Clinical Tuberculosis and Other Mycobacterial Diseases 9:24-29.
42. Myo K, Zaw M, Swe TL, Kyaw YY, Thwin T, Myo TT, Aye KO, Myint AA. 2018. Evaluation of Xpert MTB/RIF assay as a diagnostic test for pulmonary tuberculosis in children in Myanmar. International Journal of Tuberculosis and Lung Disease 22:1051-1055.
43. Nansumba M, Kumbakumba E, Orikiriza P, Muller Y, Nackers F, Debeaudrap P, Boum Y, 2nd, Bonnet M. 2016. Detection Yield and Tolerability of String Test for Diagnosis of Childhood Intrathoracic Tuberculosis. Pediatric Infectious Disease Journal 35:146-51.
44. Negash H, Legese H, Adhanom G, Mardu F, Tesfay K, Gebremeskel SG, Berhe B. 2020. Six years trend analysis of tuberculosis in Northwestern Tigrai, Ethiopia; 2019: A retrospective study. Infection and Drug Resistance 13:643-649.
45. Nhu NT, Ha DT, Anh ND, Thu DD, Duong TN, Quang ND, Lan NT, Quyet TV, Tuyen NT, Ha VT, Giang DC, Dung NH, Wolbers M, Farrar J, Caws M. 2013. Evaluation of Xpert MTB/RIF and MODS assay for the diagnosis of pediatric tuberculosis. BMC Infectious Diseases 13:31.
46. Nicol MP, Zar HJ. 2011. New specimens and laboratory diagnostics for childhood pulmonary TB: progress and prospects. Paediatric Respiratory Reviews 12:16-21.
47. Nicol MP, Spiers K, Workman L, Isaacs W, Munro J, Black F, Zemanay W, Zar HJ. 2013. Xpert MTB/RIF testing of stool samples for the diagnosis of pulmonary tuberculosis in children. Clinical Infectious Diseases 57:e18-21.
48. Nicol MP, Wood RC, Workman L, Prins M, Whitman C, Ghebrekristos Y, Mbhele S, Olson A, Jones-Engel LE, Zar HJ, Cangelosi GA. 2019. Microbiological diagnosis of pulmonary tuberculosis in children by oral swab polymerase chain reaction. Scientific Reports 9:10789.
49. Nissen TN, Rose MV, Kimaro G, Bygbjerg IC, Mfinanga SG, Ravn P. 2012. Challenges of loss to follow-up in tuberculosis research. PLoS ONE [Electronic Resource] 7:e40183.
50. Oliwa JN, Gathara D, Ogero M, van Hensbroek MB, English M, Van't Hoog A, Clinical Information N. 2019. Diagnostic practices and estimated burden of tuberculosis among children admitted to 13 government hospitals in Kenya: An analysis of two years' routine clinical data. PLoS ONE [Electronic Resource] 14:e0221145.
51. Orikiriza P, Nansumba M, Nyehangane D, Bastard M, Mugisha IT, Nansera D, Mwanga-Amumpaire J, Boum Y, 2nd, Kumbakumba E, Bonnet M. 2018. Xpert MTB/RIF diagnosis of childhood tuberculosis from sputum and stool samples in a high TB-HIV-prevalent setting. European Journal of Clinical Microbiology & Infectious Diseases 37:1465-1473.
52. Pearce EC, Woodward JF, Nyandiko WM, Vreeman RC, Ayaya SO. 2012. A systematic review of clinical diagnostic systems used in the diagnosis of tuberculosis in children. Aids Research and Treatment:401896.
53. Ramos JM, Perez-Butragueno M, Tisiano G, Yohannes T, Reyes F, Gorgolas M. 2013. Evaluation of Ziehl-Neelsen smear for diagnosis of pulmonary tuberculosis in childhood in a rural hospital in Ethiopia. International Journal of Mycobacteriology 2:171-3.
54. Ramos JM, Perez-Butragueno M, Tesfamariam A, Reyes F, Tiziano G, Endirays J, Balcha S, Elala T, Biru D, Comeche B, Gorgolas M. 2019. Comparing tuberculosis in children aged under 5 versus 5 to 14 years old in a rural hospital in southern Ethiopia: an 18-year retrospective cross-sectional study. BMC Public Health 19:856.
55. Raizada N, Sachdeva KS, Nair SA, Kulsange S, Gupta RS, Thakur R, Parmar M, Gray C, Ramachandran R, Vadera B, Ekka S, Dhawan S, Babre A, Ghedia M, Alavadi U, Dewan P, Khetrapal M, Khanna A,

Mafirakureva N, *et al. BMJ Open* 2022; 12:e058388. doi: 10.1136/bmjopen-2021-058388

Boehme C, Paramsivan CN. 2014. Enhancing TB case detection: experience in offering upfront Xpert MTB/RIF testing to pediatric presumptive TB and DR TB cases for early rapid diagnosis of drug sensitive and drug resistant TB. PLoS ONE [Electronic Resource] 9:e105346.

56. Raizada N, Sachdeva KS, Swaminathan S, Kulsange S, Khaparde SD, Nair SA, Khanna A, Chopra KK, Hanif M, Sethi GR, Umadevi KR, Keshav Chander G, Saha B, Shah A, Parmar M, Ghediya M, Jaju J, Boehme C, Paramasivan CN. 2015. Piloting Upfront Xpert MTB/RIF Testing on Various Specimens under Programmatic Conditions for Diagnosis of TB & DR-TB in Paediatric Population. PLoS ONE [Electronic Resource] 10:e0140375.
57. Raizada N, Khaparde SD, Salhotra VS, Rao R, Kalra A, Swaminathan S, Khanna A, Chopra KK, Hanif M, Singh V, Umadevi KR, Nair SA, Huddart S, Prakash CHS, Mall S, Singh P, Saha BK, Denkinger CM, Boehme C, Sarin S. 2018. Accelerating access to quality TB care for pediatric TB cases through better diagnostic strategy in four major cities of India. PLoS ONE [Electronic Resource] 13:e0193194.
58. Raizada N, Khaparde SD, Rao R, Kalra A, Sarin S, Salhotra VS, Swaminathan S, Khanna A, Chopra KK, Hanif M, Singh V, Umadevi KR, Nair SA, Huddart S, Tripathi R, Surya Prakash CH, Saha BK, Denkinger CM, Boehme C. 2018. Upfront Xpert MTB/RIF testing on various specimen types for presumptive infant TB cases for early and appropriate treatment initiation. PLoS ONE [Electronic Resource] 13:e0202085.
59. Reither K, Manyama C, Clowes P, Rachow A, Mapamba D, Steiner A, Ross A, Mfinanga E, Sasamalo M, Nsubuga M, Aloi F, Cirillo D, Jugheli L, Lwilla F. 2015. Xpert MTB/RIF assay for diagnosis of pulmonary tuberculosis in children: a prospective, multi-centre evaluation. Journal of Infection 70:392-9.
60. Sabi I, Kabyemera R, Mshana SE, Kidenya BR, Kasanga G, Gerwing-Adima LE, Meremo A, Clowes P, Rachow A, Peck RN. 2016. Pulmonary TB bacteriologically confirmed by induced sputum among children at Bugando Medical Centre, Tanzania. International Journal of Tuberculosis & Lung Disease 20:228-34.
61. Sabi I, Rachow A, Mapamba D, Clowes P, Ntinginya NE, Sasamalo M, Kamwela L, Haraka F, Hoelscher M, Paris DH, Saathoff E, Reither K. 2018. Xpert MTB/RIF Ultra assay for the diagnosis of pulmonary tuberculosis in children: a multicentre comparative accuracy study. Journal of Infection 77:321-327.
62. Sekadde MP, Wobudeya E, Joloba ML, Ssengooba W, Kisembo H, Bakeera-Kitaka S, Musoke P. 2013. Evaluation of the Xpert MTB/RIF test for the diagnosis of childhood pulmonary tuberculosis in Uganda: a cross-sectional diagnostic study. BMC Infectious Diseases 13:133.
63. Sharma S, Sarin R, Sahu G, Shukla G. 2020. Demographic profile, clinical and microbiological predictors of mortality amongst admitted pediatric TB patients in a tertiary referral tuberculosis hospital. Indian Journal of Tuberculosis 67:312-319.
64. Shata AMA, Coulter JBS, Parry CM, Chingani G, Broadhead RL, Hart CA. 1996. Sputum induction for the diagnosis of tuberculosis. Archives of Disease in Childhood 74:535-537.
65. Singh M, Sethi GR, Mantan M, Khanna A, Hanif M. 2016. Xpert MTB/RIF assay for the diagnosis of pulmonary tuberculosis in children. International Journal of Tuberculosis and Lung Disease 20:839-843.
66. Sorsa A, Jerene D, Negash S, Habtamu A. 2020. Use of Xpert Contributes to Accurate Diagnosis, Timely Initiation, and Rational Use of Anti-TB Treatment Among Childhood Tuberculosis Cases in South Central Ethiopia. Pediatric Health Medicine & Therapeutics 11:153-160.
67. Ssengooba W, Iragena JD, Nakiyingi L, Mujumbi S, Wobudeya E, Mboizi R, Boulware D, Meya DB, Choo L, Crook AM, Lebeau K, Joloba M, Demers AM, Cresswell FV, Gibb DM. 2020. Accuracy of Xpert Ultra in Diagnosis of Pulmonary Tuberculosis among Children in Uganda: a Substudy from the SHINE Trial. Journal of Clinical Microbiology 58:24.
68. Surya A, Setyaningsih B, Suryani Nasution H, Gita Parwati C, Yuzwar YE, Osberg M, Hanson CL, Hymoff A, Mingkwan P, Makayova J, Gebhard A, Waworuntu W. 2017. Quality Tuberculosis Care in Indonesia: Using Patient Pathway Analysis to Optimize Public-Private Collaboration. J Infect Dis 216:S724-S732.
69. Swaminathan S, Datta M, Radhamani MP, Mathew S, Reetha AM, Rajajee S, Mathew R, Radkhakrishnan A, Raghu MB. 2008. A profile of bacteriologically confirmed pulmonary tuberculosis in children. Indian Pediatrics 45:743-747.
70. Walters E, Demers AM, van der Zalm MM, Whitelaw A, Palmer M, Bosch C, Draper HR, Gie RP, Hesseling AC. 2017. Stool Culture for Diagnosis of Pulmonary Tuberculosis in Children. Journal of Clinical Microbiology 55:3355-3365.
71. Walters E, van der Zalm MM, Palmer M, Bosch C, Demers AM, Draper H, Goussard P, Schaaf HS, Friedrich SO, Whitelaw A, Warren R, Gie RP, Hesseling AC. 2017. Xpert MTB/RIF on Stool Is Useful for the Rapid Diagnosis of Tuberculosis in Young Children With Severe Pulmonary Disease. Pediatric Infectious Disease Journal 36:837-843.

Mafirakureva N, *et al. BMJ Open* 2022; 12:e058388. doi: 10.1136/bmjopen-2021-058388

72. Walters E, Scott L, Nabeta P, Demers AM, Reubenson G, Bosch C, David A, van der Zalm M, Havumaki J, Palmer M, Hesseling AC, Ncayiyana J, Stevens W, Alland D, Denkinger C, Banada P. 2018. Molecular Detection of Mycobacterium tuberculosis from Stools in Young Children by Use of a Novel Centrifugation-Free Processing Method. Journal of Clinical Microbiology 56:09.
73. Yadav R, Vaidya P, Mathew JL, Singh S, Khaneja R, Agarwal P, Singh M, Sethi S. 2020. Diagnostic accuracy of Xpert MTB/RIF Ultra for detection of Mycobacterium tuberculosis in children: a prospective cohort study. Letters in Applied Microbiology 08:08.
74. Zar HJ, Honslo D, Apolles P, Swingler G, Hussey G. 2005. Induced sputum versus gastric lavage for microbiological confirmation of pulmonary tuberculosis in infants and young children: a prospective study. Lancet 365:130-134.
75. Zar HJ, Workman L, Isaacs W, Munro J, Black F, Eley B, Allen V, Boehme CC, Zemanay W, Nicol MP. 2012. Rapid molecular diagnosis of pulmonary tuberculosis in children using nasopharyngeal specimens. Clinical Infectious Diseases 55:1088-95.
76. Zar HJ, Workman L, Isaacs W, Dheda K, Zemanay W, Nicol MP. 2013. Rapid diagnosis of pulmonary tuberculosis in African children in a primary care setting by use of Xpert MTB/RIF on respiratory specimens: a prospective study. The Lancet Global Health 1:e97-e104.
77. Zar HJ, Workman LJ, Prins M, Bateman LJ, Mbhele SP, Whitman CB, Denkinger CM, Nicol MP. 2019. Tuberculosis Diagnosis in Children Using Xpert Ultra on Different Respiratory Specimens. American Journal of Respiratory & Critical Care Medicine 200:1531-1538.
