# Supplementary material for: Xpert Ultra stool testing to diagnose tuberculosis in children in Ethiopia and Indonesia: a model-based cost-effectiveness analysis
Source: BMJ Open. 2022 Jul 1;12(7):e058388. doi: 10.1136/bmjopen-2021-058388 (PMC9252203; doi:10.1136/bmjopen-2021-058388)
Supplement: Supplementary data [file bmjopen-2021-058388supp003.pdf]

Xpert Ultra stool testing to diagnose tuberculosis in children in Ethiopia and Indonesia: a model-based cost-effectiveness analysis.

Appendix 2b: Overview of cost parameters

Contents

General costing assumptions 3
Identifying resources used 3
Measuring resource utilization 3
Valuation of resources 4
Clinical assessment 4
TB assessment at health centre 4
TB assessment at hospital 5
TST 6
CXR 6
Sample collection 6
Bacteriological assessment 6
Sputum smear microscopy (SSM) examination 6
GeneXpert test 6
Clinical (re-) assessment 7
TB reassessment at health centre 7
TB reassessment at hospital 7
TB treatment 7
TB treatment medications 7
TB treatment follow-up at health centre 7
TB treatment follow-up at hospital 7
Laboratory monitoring 8
Comparison with other cost estimates 8
Costs tables 9
Table A1 Costs for Ethiopia & Indonesia 9
Table A2 Comparison of estimated unit costs for diagnostic visit, sputum collection, smear microscopy examination and XpertMTB/RIF test with recently published Better estimates of the costs of TB control (Value TB) project[13]. 10
References 11

Mafirakureva N, *et al. BMJ Open* 2022; 12:e058388. doi: 10.1136/bmjopen-2021-058388

General costing assumptions

Identifying resources used

A careful review of the diagnostic and treatment algorithm for childhood TB for Ethiopia (ETH)[1] and Indonesia (IDN)[2] was performed to identify resource use and costs associated with the diagnosis and treatment for childhood TB in the current standard of care (SOC) and under the intervention of using stool with Xpert for the diagnosis of TB **(Figures 1-3)**. The main activities in both the SOC and the intervention included symptom-based screening for TB, clinical evaluation, sample collection, bacteriological examination, radiological examination, empiric antibiotic therapy to exclude other diseases, treatment initiation for diagnosed TB cases, TB treatment follow-up and TB treatment monitoring laboratory tests. The following cost components were identified; health facility visit (for TB screening, rescreening, diagnosis and treatment), sample collection (spontaneous sputum, stool), bacteriological examination (smear microscopy, GeneXpert), and medicines (anti-TB medicines, pyridoxine).

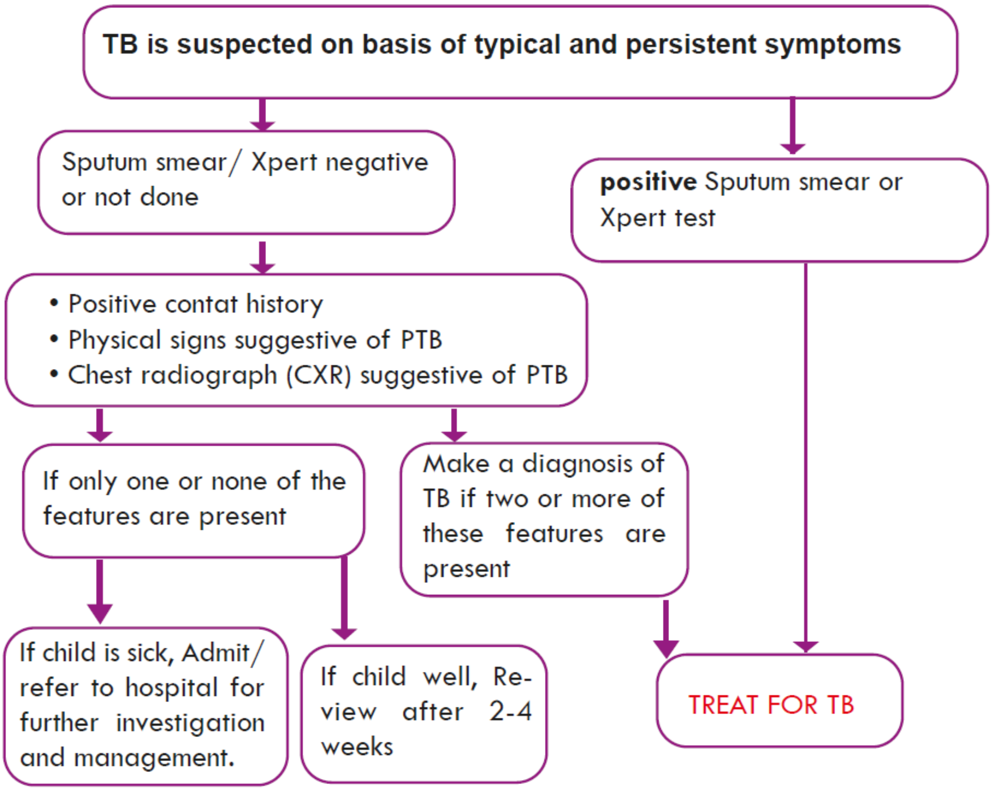

Figure 1. Algorithm for diagnosis of tuberculosis in HIV uninfected children in Ethiopia[1]

Measuring resource utilization

The quantities of each resource type consumed in the diagnostic and treatment algorithm for childhood TB was informed by the national guidelines for the management of childhood TB in each country (**Figures 1-3**). Local TB experts collaborating with KNCV on childhood TB related studies provided country-specific input on resource use.

Mafirakureva N, *et al. BMJ Open* 2022; 12:e058388. doi: 10.1136/bmjopen-2021-058388

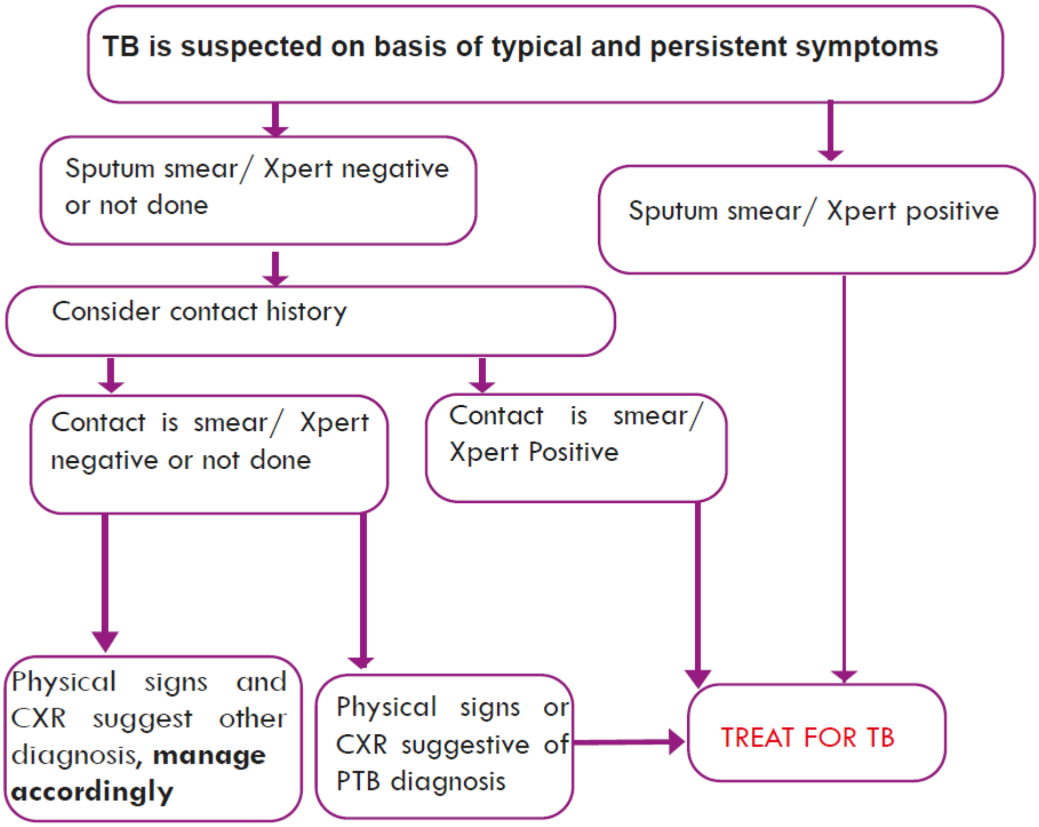

Figure 2. Algorithm for diagnosis of tuberculosis in HIV-infected children in Ethiopia[1]

Valuation of resources

The costs for each resource were estimated by attaching monetary values using relevant unit costs for each country. Summing up these costs (per patient) gives an estimate of the total cost. All costs were estimated from the healthcare provider's perspective and are reported in 2019 USD. Historical costs were adjusted for inflation to 2019 prices using relevant GDP deflators[3] and costs from other countries were transferred to Ethiopia and Indonesia by applying relevant purchasing power parity conversion factors[3]. Costs were assumed to accrue in the present, with no discounting applied. The following costs were estimated.

Clinical assessment

This cost comprises of the cost of clinical assessment to investigate children with presumptive TB and the cost associated with collecting the necessary samples for bacteriological evaluation.

TB assessment at health centre

We assumed the cost for the initial TB assessment at the primary health centre to be equivalent to the country-specific cost of two outpatient visits (range; 1-3 visits) to a health centre with no beds based on WHO-CHOICE estimates[4]. This equates to $10.22 (95% UI; 4.93-15.50) in ETH and $43.35 (95% UI; 19.11-67.59) in IDN.

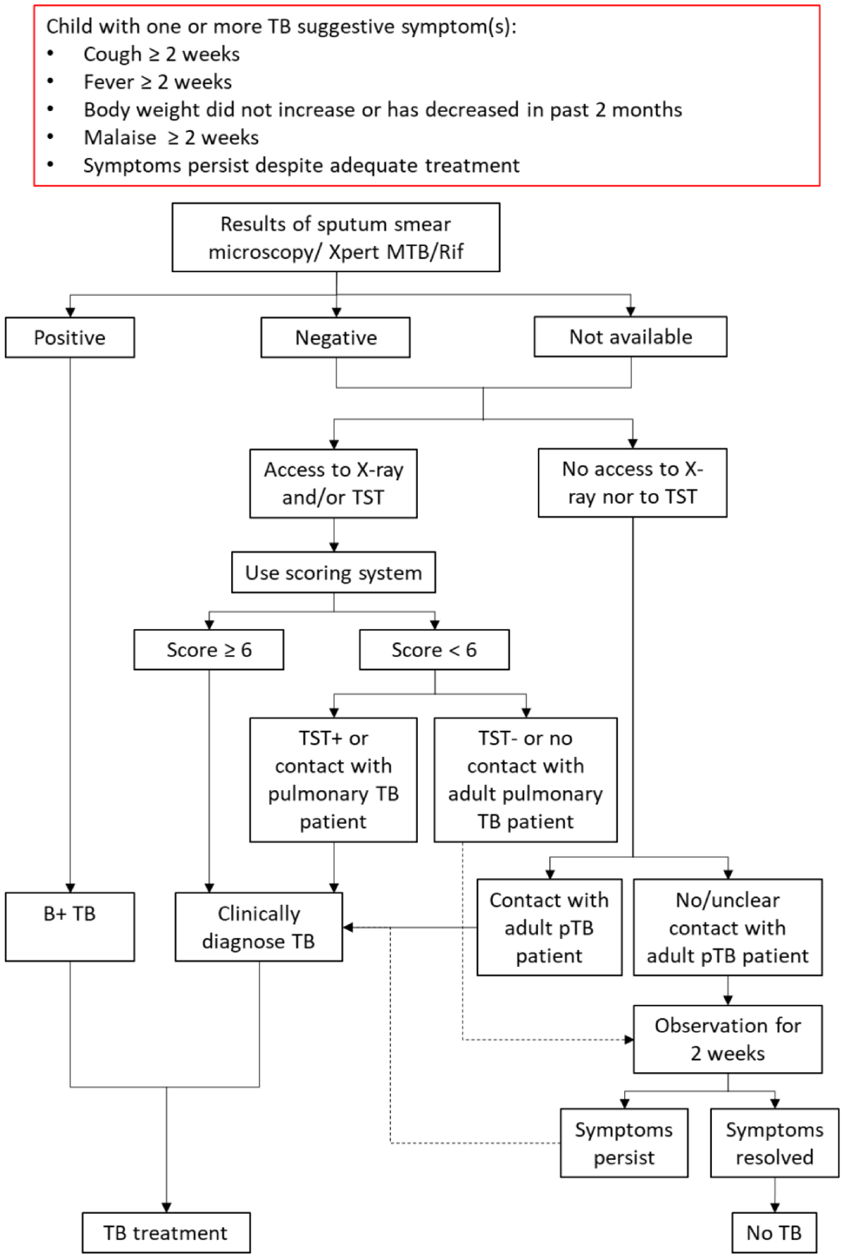

Figure 3. Algorithm for diagnosis of tuberculosis in children in Indonesia[2]

TB assessment at hospital

Similarly, we assumed the cost for the initial TB assessment at the hospital to be equivalent to the country-specific cost of two outpatient visits (range; 1-3 visits) to a primary hospital, defined as a ‘hospitals intended primarily for treating simple cases (e.g. “district hospital”)’ [4]. This results in an estimate of $14.37 (95% UI; 7.79-20.96) in ETH and $61.00 (95% UI; 30.76-91.23) in IDN. The costs of tuberculin skin test (TST) and chest X-ray were separately addressed (see below).

Mafirakureva N, *et al. BMJ Open* 2022; 12:e058388. doi: 10.1136/bmjopen-2021-058388

TST

The Indonesian NTP uses a scoring system for the diagnosis of TB if a bacteriological diagnosis cannot be made (Figure 1). The scoring system uses a combination of tuberculin skin test (TST) results, chest X-ray results (CXR), symptoms and history of contact with TB patients (Table 1). TST and CXR are often not available especially at the primary health centres, hence these costs are currently not modelled.

CXR

The unit cost for CXR for Ethiopia ($8.75) was based on the cost per radiograph reported by the Ethiopian NTP[5]. The unit cost applied for CXR for Indonesia ($11.52) was based on a previous MSH estimate[6]. This cost is applied to a proportion of children assessed at the hospital (80-90%) only since chest X-rays are not available at the primary health centres.

Sample collection

Availability of advanced sample collection procedures is generally limited in both countries, with sputum induction occurring only in big hospitals in Indonesia and nasogastric aspiration only available at big teaching hospitals in Ethiopia. We assumed only self-expectorated sample collection is available and applied the adjusted unit costs for collecting two samples for testing with smear microscopy ($4.64 in ETH and $3.48 in IDN) or one sample for testing with GeneXpert ($2.32 in ETH and $1.74 in IDN) per child based on a study done in adults in South Africa [7]. The unit costs applied for procedures for collecting a single stool sample ($1.67) in the intervention are based on estimates provided by the Paediatric Operational Sustainability Expertise Exchange group (POSEE group) [8]. The POSEE group developed a budgeting tool to assist national TB programs in estimating the costs related to the procurement of devices and consumables needed for sample collection in the paediatric population. These POSEE group cost estimates currently exclude staff, space, training, sample transportation and overheads costs.

Bacteriological assessment

The cost for bacteriological assessment for TB comprises of the costs of testing using either a sputum smear microscopy (SSM) examination or a single GeneXpert test, depending on the availability of the test at each level of care (primary health centre versus hospital) in Ethiopia and Indonesia. Bacteriological testing with the GeneXpert is not widely available in both countries and most testing centres use sputum smear microscopy while some centres refer samples to a GeneXpert testing facility. We therefore assumed sole use of smear microscopy in both countries in the standard of care where two samples are collected for testing in the base case.

Sputum smear microscopy (SSM) examination

The unit cost for SSM for Ethiopia was based on the microscopy cost per test ($1.50) reported by the Ethiopian NTP[5] resulting in an adjusted cost of $1.69. The unit cost for SSM in Indonesia ($3.77) was based on a previous MSH estimate[6].

GeneXpert test

The unit cost for the GeneXpert test was estimated based on country specific data available from the OneHealth Tool[9]. These data include staff times, staff salaries, the Xpert cartridge and consumables. The cost of the GeneXpert equipment was estimated based on the procurement cost of the Xpert MTB/RIF 4-module machine and its annual maintenance cost available from the Global Drug Facility[10]. Costs associated with unused GeneXpert

Mafirakureva N, *et al. BMJ Open* 2022; 12:e058388. doi: 10.1136/bmjopen-2021-058388

equipment capacity were estimated by accounting for the number of tests performed per day in relation to an assumed daily maximum capacity of 16 tests[11]. Overhead costs were estimated as 5% of the total direct costs based on recent studies showing overhead costs contributing 1-10% of total Xpert costs[11-13]. The estimated unit costs for the GeneXpert test are $ 26.04 (95% UI; 18.95-33.13) for ETH and $23.70 (95% UI; 16.59-30.81) for IDN.

Clinical (re-) assessment

This cost comprised of the cost of clinical re-assessment of children with significant clinical manifestations of TB following exclusion of TB during the initial assessment.

TB reassessment at health centre

We assumed the cost for TB re-assessment at the primary health centre to be equivalent to the country-specific cost of a single outpatient visit to a health centre with no beds[4]. This equated to $5.11 (95% UI; 2.86-7.35) in ETH and $21.68 (95% UI; 11.16-32.19) in IDN.

TB reassessment at hospital

Similarly, we assumed the cost for TB re-assessment at the hospital to be equivalent to the country-specific cost of a single outpatient visit to a primary hospital, defined as a 'hospitals intended primarily for treating simple cases (e.g. "district hospital")' [4]. This resulted in an estimate of $7.19 (95% UI; 4.51-9.87) in ETH and $30.50 (95% UI; 17.84-43.16) in IDN.

TB treatment

Treatment cost for bacteriologically confirmed TB comprises of the cost of anti-tuberculosis drugs including pyridoxine, the costs of follow-up visits (drug pickups or medical review) at the healthcare facilities and costs of laboratory monitoring.

TB treatment medications

The costs of anti-tuberculosis drugs and pyridoxine were estimated using weight band based dosing and applying unit costs available from the Global Drug Facility[14]. We assumed a treatment duration of 6 months. This resulted in the following costs: $11.38, $22.77, $34.15, and $45.54 for children in the weight bands 4-7kg, 8-11kg, 12-15kg and 16-24kg, respectively. The cost of pyridoxine for the duration of TB treatment was estimated to be $2.52[14].

TB treatment follow-up at health centre

We assume the cost for each TB treatment follow-up visit at the primary health centre to be equivalent to the country-specific cost of a single outpatient visit to a health centre with no beds (see above)[4]. This unit cost was multiplied by the number of follow-up visits dictated by the national TB treatment algorithm to estimate the total cost for TB treatment follow-up. Based on input from the local TB experts, we assumed 6 follow-up visits per child on TB treatment in IND and 72 follow-up visits per child on TB treatment in ETH where clinic-based directly observed therapy is used in children. This resulted in the TB treatment follow-up cost at the health centre of $367.76 (95% UI; 167.54-814.45) in ETH and $130.05 (95% UI; 55.51-307.92) in IDN.

TB treatment follow-up at hospital

Similarly, we assumed the cost for each TB treatment follow-up visit at the hospital to be equivalent to the country-specific cost of a single outpatient visit to a primary hospital,

Mafirakureva N, *et al. BMJ Open* 2022; 12:e058388. doi: 10.1136/bmjopen-2021-058388

defined as a 'hospitals intended primarily for treating simple cases (e.g. "district hospital")' [4]. This unit cost was multiplied by the number of follow-up visits to estimate the total cost for TB treatment follow-up. Based on input from the two local TB experts, we assumed 6 follow-up visits per child on TB treatment in IND and 72 follow-up visits per child on TB treatment in ETH where directly observed therapy is used in children. This resulted in the TB treatment follow-up cost of $517.48 (95% UI; 261.52-1033.08) in ETH and $183.00 (95% UI; 86.65-390.58) in IDN.

Laboratory monitoring

Laboratory monitoring is usually not done in children, unless they can spontaneously expectorate a sample. This is only the case for a certain proportion of the oldest age group, which is likely lower than the estimate that we use for the oldest age class (see Table A8 in Appendix 2a). Therefore, we currently do not include the cost of laboratory monitoring in our analysis.

Comparison with other cost estimates

We evaluated the accuracy of our unit cost estimates by comparing them to the recently published cost estimates for Ethiopia by the Better estimates of the costs of TB control (Value TB) project[15]. We compared the unit costs for a diagnostic visit, sputum sample collection, sputum smear microscopy examination, XpertMTB/RIF test and treatment monitoring visit. Although not exactly the same, our unit costs were quite comparable to the Value-TB cost estimates.

Costs tables

Table A1 Costs for Ethiopia & Indonesia

Cost parameter	Description	Ethiopia	Indonesia	References
c_a_phc<br>c_clin.phc	TB clinical assessment at health centre	10.22 (4.93 - 15.50)	43.35 (19.11 - 67.59)	[4]
c_e_phc<br>c_g.phc	TB clinical reassessment at health centre	5.11 (2.86 - 7.35)	21.68 (11.16 - 32.19)	[4]
c_b.phc.sess<br>c_b.h.sess	Self-expectorated sputum sample	2.32 (1.74 - 2.90)	1.74 (1.30 - 2.17)	[8]
c_b.phc.ss<br>c_b.h.ss	Stool sample	1.67 (1.25 - 2.09)	1.67 (1.25 - 2.09)	[8]
c_b.phc.ssm<br>c_b.h.ssm	Sputum smear microscopy	3.39 (1.94 - 4.83)	7.54 (5.96 - 9.12)	[5, 6]
c_b.phc.xpert<br>c_b.h.xpert	GeneXpert test	26.04 (18.95 - 33.13)	23.70 (16.59 - 30.81)	Estimated based on data from OneHealth Tool[9]
c_c.phc<br>c_d.phc<br>c_f.phc<br>c_h.phc	TB treatment at health centre	396.22 (220.27 - 572.18)	158.51 (81.18 - 235.85)	[4, 14]
c_a.h<br>c_clin.h	TB clinical assessment at hospital	14.37 (7.79 - 20.96)	61.00 (30.76 - 91.23)	[4]
c_e.h<br>c_g.h	TB clinical reassessment at hospital	7.19 (4.51 - 9.87)	30.50 (17.84 - 43.16)	[4]
c_c.h<br>c_f.h<br>c_d.h<br>c_h.h	TB treatment at hospital	396.22 (220.27 - 572.18)	158.51 (81.18 - 235.85)	[4, 14]

Mafirakureva N, *et al. BMJ Open* 2022; 12:e058388. doi: 10.1136/bmjopen-2021-058388

Table A2 Comparison of estimated unit costs for diagnostic visit, sputum collection, smear microscopy examination and XpertMTB/RIF test with recently published Better estimates of the costs of TB control (Value TB) project[13].

	**Country**	**Diagnostic visit**	**Sputum collection**	**Smear microscopy examination**	**XpertMTB/RIF test**
Health centre	ETH	5.84 (1.14-18.14)	4.64	1.69 (2.40-4.96)	26.47 (16.67-49.03)
	IND	9.91 (1.89-30.54)	4.22	3.77	27.07 (17.04-49.40)
	Value-TB ETH	3.33 (1.00-9.18)	3.28 (1-7.77)	4.53 (1.00-10.30)	20.83 (16-26.69)
Primary hospital	ETH	5.84 (1.61-26.39)	4.64	1.69 (2.40-4.96)	26.47 (16.67-49.03)
	IND	9.91 (2.75-18.76)	4.22	3.77	27.07 (17.04-49.40)
	Value-TB ETH	6.41 (1-14.66)	5.12 (3.00-6.91)	6.24 (4.00-8.43)	37.87 (19-57.78)

References

1. Ministry of Health Ethiopia., *Guidelines for Clinical and Programmatic Management of TB, DR-TB and Leprosy in Ethiopia, 7th edition*. 2020, Ministry of Health Ethiopia: Addis Ababa, Ethiopia.
2. Ministry of Health Republic of Indonesia., *National guideline for TB control*. 2016, Ministry of Health Republic of Indonesia: Jakarta, Indonesia.
3. World Bank. *World Bank national accounts data, and OECD National Accounts data files. GDP deflator*. 2021 14/05/2021]; Available from: https://data.worldbank.org/indicator/NY.GDP.DEFL.ZS.
4. Stenberg, K., et al., *Econometric estimation of WHO-CHOICE country-specific costs for inpatient and outpatient health service delivery.* Cost Eff Resour Alloc, 2018. **16**: p. 11.
5. Tesfaye, A., et al., *Modeling the patient and health system impacts of alternative xpert® MTB/RIF algorithms for the diagnosis of pulmonary tuberculosis in Addis Ababa, Ethiopia.* BMC infectious diseases, 2017. **17**(1): p. 318-318.
6. Jarrah Z, C.D., Hafidz F. , *The cost of scaling up TB services in Indonesia. TB CARE I–management sciences for health. Cambridge: Management Science for Health. .* 2013.
7. Peter, J.G., et al., *Comparison of two methods for acquisition of sputum samples for diagnosis of suspected tuberculosis in smear-negative or sputum-scarce people: a randomised controlled trial.* Lancet Respir Med, 2013. **1**(6): p. 471-8.
8. Paediatric Operational Sustainability Expertise Exchange group. *Paediatric Operational Sustainability Expertise Exchange group (POSEE group) budgeting tools* 2020 [cited 16/02/2021; Available from: https://www.dropbox.com/sh/rmzlqoot9m1muxe/AACOPh8SuS4WwMQD_vfN-oHCa?dl=0.
9. World Health Organization. *OneHealth Tool.* 2020; Available from: http://www.who.int/choice/onehealthtool/en/.
10. Stop TB Partnership | Global Drug Facility, *Global Drug Facility (GDF) Diagnostics catalogue August 2020.* 2021.
11. Sarin, S., et al., *Cost and operational impact of promoting upfront GeneXpert MTB/RIF test referrals for presumptive pediatric tuberculosis patients in India.* PLOS ONE, 2019. **14**(4): p. e0214675.
12. Sohn, H., et al., *Cost and affordability analysis of TB-LAMP and Xpert MTB/RIF assays as routine diagnostic tests in peripheral laboratories in Malawi and Vietnam.* J Glob Health Sci, 2019. **1**(1).
13. Pooran, A., et al., *Point of care Xpert MTB/RIF versus smear microscopy for tuberculosis diagnosis in southern African primary care clinics: a multicentre economic evaluation.* The Lancet. Global health, 2019. **7**(6): p. e798-e807.
14. Stop TB Partnership | Global Drug Facility, *Global Drug Facility (GDF) Medicines catalogue January 2021.* 2021.
15. Sweeney, S., et al., *Value TB Dataset: costs per intervention*. 2021, Harvard Dataverse.

Mafirakureva N, *et al. BMJ Open* 2022; 12:e058388. doi: 10.1136/bmjopen-2021-058388
