# Supplementary material for: Xpert Ultra stool testing to diagnose tuberculosis in children in Ethiopia and Indonesia: a model-based cost-effectiveness analysis
Source: BMJ Open. 2022 Jul 1;12(7):e058388. doi: 10.1136/bmjopen-2021-058388 (PMC9252203; doi:10.1136/bmjopen-2021-058388)
Supplement: Supplementary data [file bmjopen-2021-058388supp004.pdf]

Xpert Ultra stool testing to diagnose tuberculosis in children in Ethiopia and Indonesia: a model-based cost-effectiveness analysis.

Appendix 3: Supplementary results

Additional results for base case analysis 2
Age-specific results 2
Age 0-4 years 2
Age 5-14 years 4
Cost-effectiveness acceptability curves 6

Results for low prevalence sensitivity analysis 8
Age-specific results 8
All ages: 0-14 years 8
Age 0-4 years 10
Age 5-14 years 12

Results for Xpert baseline sensitivity analysis 14
Age-specific results 14
All ages: 0-14 years 14
Age 0-4 years 16
Age 5-14 years 18

Results for 0% discount rate sensitivity analysis 20
Age-specific results 20
All ages: 0-14 years 20
Age 0-4 years 22
Age 5-14 years 24

Results for 5% discount rate sensitivity analysis 26
Age-specific results 26
All ages: 0-14 years 26
Age 0-4 years 28
Age 5-14 years 30

Comparison of ICERs for sensitivity analyses 32

Mafirakureva N, *et al. BMJ Open* 2022; 12:e058388. doi: 10.1136/bmjopen-2021-058388

Additional results for base case analysis

Age-specific results

Age 0-4 years

Table A1 Ethiopia

**Quantity per 100 children with presumptive TB (unless stated):**	**Standard of care**	**Intervention**	**Difference**
children with true TB	45.5 (8.7 - 85.0)	45.5 (8.7 - 85.0)	0.0 (0.0 - 0.0)
assessments	208.1 (172.8 - 241.7)	252.9 (217.5 - 286.1)	44.7 (32.9 - 55.1)
bacteriological investigations	3.4 (2.1 - 4.9)	103.1 (87.6 - 112.5)	99.8 (84.3 - 109.1)
anti-TB treatments (ATT)	36.6 (15.3 - 59.7)	36.4 (15.2 - 59.3)	-0.2 (-2.8 - 2.7)
ATT initiated at PHC	68.3 (59.6 - 75.7)	81.0 (70.9 - 88.4)	12.7 (8.0 - 17.4)
percent of true-positive receiving ATT	66.2 (55.9 - 75.4)	66.8 (58.4 - 74.6)	0.7 (-3.4 - 5.1)
percent of ATT bacteriologically confirmed	0.6 (0.3 - 1.0)	31.4 (20.4 - 42.2)	30.8 (19.9 - 41.4)
percent of ATT false-positive	21.7 (2.4 - 65.8)	20.7 (2.4 - 63.4)	-1.0 (-4.2 - 1.9)
referrals, inc. self-referrals	40.1 (28.3 - 51.7)	14.7 (9.1 - 21.8)	-25.4 (-33.2 - -17.4)
deaths	7.5 (1.4 - 15.2)	7.3 (1.4 - 14.7)	-0.1 (-1.2 - 0.7)
life-years lost	205.9 (37.9 - 418.6)	202.4 (37.5 - 405.7)	-3.4 (-32.0 - 19.9)
cost	17934.1 (7124.0 - 35159.0)	17667.9 (7614.0 - 32685.5)	-266.2 (-13326.6 - 12081.4)

Mafirakureva N, *et al. BMJ Open* 2022; 12:e058388. doi: 10.1136/bmjopen-2021-058388

Table A2 Indonesia

Quantity per 100 children with presumptive TB (unless stated):	Standard of care	Intervention	Difference
children with true TB	45.5 (8.7 - 85.0)	45.5 (8.7 - 85.0)	0.0 (0.0 - 0.0)
assessments	209.4 (173.8 - 242.9)	255.2 (219.1 - 289.2)	45.9 (33.8 - 56.0)
bacteriological investigations	3.4 (2.1 - 5.0)	103.6 (88.2 - 113.0)	100.2 (84.9 - 109.5)
anti-TB treatments (ATT)	36.8 (15.4 - 60.4)	36.5 (15.2 - 59.6)	-0.3 (-3.0 - 2.4)
ATT initiated at PHC	70.1 (61.1 - 77.4)	83.6 (72.6 - 90.3)	13.4 (8.6 - 18.1)
percent of true-positive receiving ATT	66.6 (56.3 - 76.0)	67.0 (58.5 - 74.8)	0.4 (-3.7 - 4.8)
percent of ATT bacteriologically confirmed	0.6 (0.3 - 1.0)	31.4 (20.4 - 42.1)	30.8 (20.0 - 41.3)
percent of ATT false-positive	21.8 (2.4 - 65.8)	20.7 (2.4 - 63.7)	-1.1 (-4.3 - 1.9)
referrals, inc. self-referrals	41.5 (29.2 - 53.1)	15.3 (9.5 - 22.4)	-26.3 (-34.4 - -17.9)
deaths	7.4 (1.4 - 15.1)	7.3 (1.4 - 14.6)	-0.1 (-1.1 - 0.8)
life-years lost	209.8 (38.7 - 429.1)	207.8 (39.0 - 416.6)	-2.0 (-31.0 - 23.2)
cost	13672.3 (7286.9 - 22370.9)	14090.7 (8344.4 - 21727.0)	418.4 (-8064.8 - 9011.5)

Mafirakureva N, *et al. BMJ Open* 2022; 12:e058388. doi: 10.1136/bmjopen-2021-058388

Age 5-14 years

Table A3 Ethiopia

Quantity per 100 children with presumptive TB (unless stated):	Standard of care	Intervention	Difference
children with true TB	45.5 (8.7 - 85.0)	45.5 (8.7 - 85.0)	0.0 (0.0 - 0.0)
assessments	197.9 (169.6 - 225.2)	242.2 (200.8 - 281.7)	44.3 (24.8 - 62.7)
bacteriological investigations	47.1 (12.5 - 82.7)	101.9 (86.2 - 111.6)	54.8 (16.5 - 91.1)
anti-TB treatments (ATT)	29.6 (10.6 - 53.8)	42.6 (18.5 - 67.8)	13.0 (1.5 - 30.3)
ATT initiated at PHC	74.6 (63.8 - 84.3)	82.4 (71.8 - 90.2)	7.8 (2.1 - 12.9)
percent of true-positive receiving ATT	53.6 (31.9 - 72.9)	76.8 (69.4 - 83.1)	23.2 (5.1 - 45.4)
percent of ATT bacteriologically confirmed	14.5 (2.4 - 39.5)	33.7 (20.3 - 46.1)	19.2 (-5.9 - 37.4)
percent of ATT false-positive	21.6 (2.6 - 65.3)	22.4 (2.8 - 66.2)	0.8 (-2.5 - 6.2)
referrals, inc. self-referrals	23.1 (8.8 - 39.1)	13.3 (7.3 - 20.5)	-9.7 (-22.7 - 4.4)
deaths	3.4 (0.6 - 7.4)	1.9 (0.3 - 3.9)	-1.5 (-4.0 - -0.2)
life-years lost	93.7 (16.9 - 203.0)	52.5 (9.6 - 107.9)	-41.1 (-111.6 - -4.3)
cost	14407.8 (5303.7 - 29936.5)	20277.4 (8872.7 - 37127.0)	5869.6 (-6634.5 - 19361.5)

Table A4 Indonesia

**Quantity per 100 children with presumptive TB (unless stated):**	**Standard of care**	**Intervention**	**Difference**
children with true TB	45.5 (8.7 - 85.0)	45.5 (8.7 - 85.0)	0.0 (0.0 - 0.0)
assessments	198.8 (170.2 - 226.2)	244.3 (202.0 - 284.1)	45.5 (25.6 - 64.3)
bacteriological investigations	47.3 (12.7 - 83.3)	102.3 (86.7 - 112.2)	54.9 (16.6 - 91.6)
anti-TB treatments (ATT)	29.6 (10.5 - 54.2)	42.7 (18.5 - 68.1)	13.1 (1.4 - 30.8)
ATT initiated at PHC	77.2 (65.5 - 87.0)	85.1 (73.6 - 92.2)	7.9 (1.5 - 13.5)
percent of true-positive receiving ATT	53.6 (31.5 - 73.0)	76.9 (69.6 - 83.3)	23.3 (4.9 - 45.9)
percent of ATT bacteriologically confirmed	14.1 (2.3 - 38.3)	33.7 (20.2 - 46.1)	19.5 (-4.9 - 37.4)
percent of ATT false-positive	21.7 (2.6 - 65.3)	22.4 (2.8 - 66.3)	0.8 (-2.6 - 6.1)
referrals, inc. self-referrals	23.9 (9.1 - 40.3)	13.8 (7.5 - 21.2)	-10.1 (-23.7 - 4.3)
deaths	3.4 (0.6 - 7.4)	1.9 (0.4 - 3.9)	-1.5 (-4.1 - -0.1)
life-years lost	96.6 (17.4 - 210.3)	53.9 (10.0 - 111.1)	-42.7 (-115.7 - -4.3)
cost	11270.2 (6013.6 - 18958.7)	14987.1 (8815.0 - 23229.9)	3716.9 (-3812.4 - 10646.6)

Mafirakureva N, *et al. BMJ Open* 2022; 12:e058388. doi: 10.1136/bmjopen-2021-058388

Cost-effectiveness acceptability curves

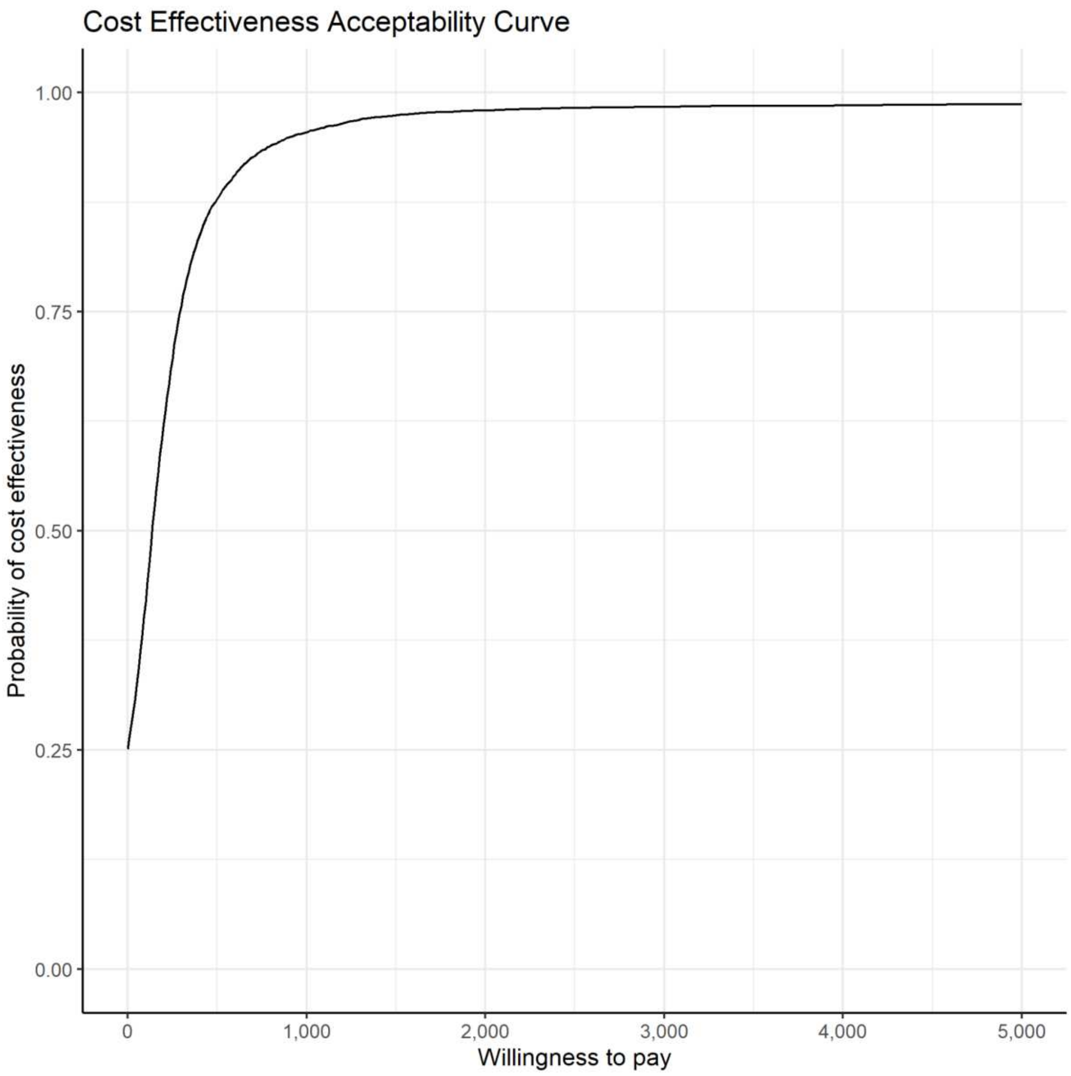

Figure A1 Ethiopia

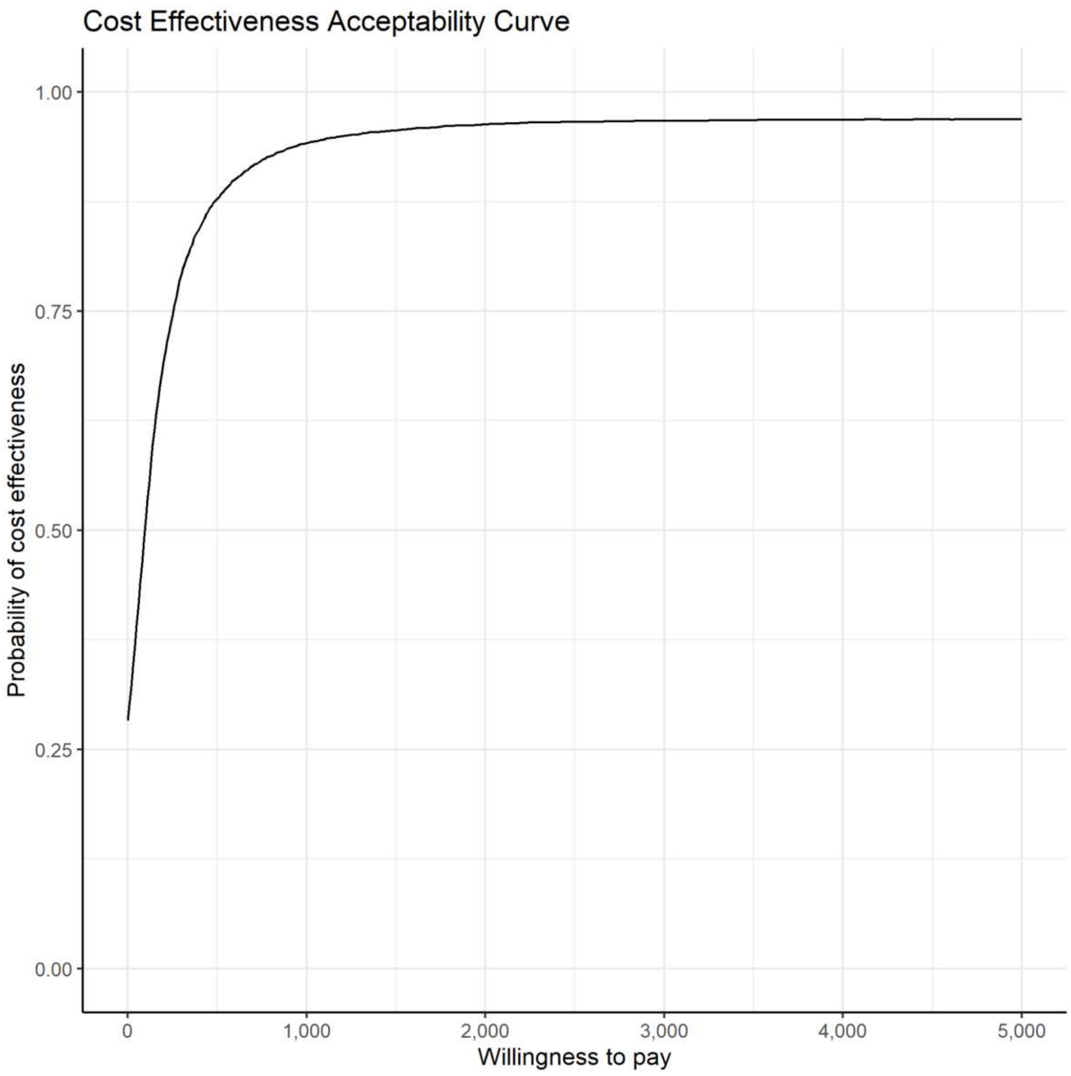

Figure A2 Indonesia

Results for low prevalence sensitivity analysis

Age-specific results

All ages: 0-14 years

Table A5 Ethiopia

Quantity per 100 children with presumptive TB (unless stated):	Standard of care	Intervention	Difference
children with true TB	22.8 (4.4 - 42.5)	22.8 (4.4 - 42.5)	0.0 (0.0 - 0.0)
assessments	224.4 (203.7 - 248.1)	260.5 (239.5 - 281.9)	36.0 (22.2 - 53.2)
bacteriological investigations	32.9 (9.7 - 60.1)	98.3 (85.7 - 110.0)	65.3 (36.2 - 91.3)
anti-TB treatments (ATT)	22.3 (11.0 - 35.6)	25.5 (13.5 - 38.0)	3.2 (-3.1 - 11.4)
ATT initiated at PHC	66.7 (56.8 - 76.9)	84.0 (72.4 - 93.3)	17.3 (8.7 - 26.2)
percent of true-positive receiving ATT	60.4 (44.3 - 74.6)	71.2 (64.4 - 77.6)	10.8 (-2.7 - 27.7)
percent of ATT bacteriologically confirmed	6.9 (1.5 - 17.2)	29.0 (17.8 - 39.9)	22.1 (9.7 - 34.0)
percent of ATT false-positive	41.2 (17.1 - 79.8)	39.7 (16.7 - 78.8)	-1.5 (-5.9 - 4.2)
referrals, inc. self-referrals	41.7 (22.6 - 60.9)	9.4 (2.0 - 21.6)	-32.2 (-52.2 - -12.2)
deaths	2.3 (0.4 - 4.8)	2.1 (0.4 - 4.4)	-0.2 (-1.1 - 0.3)
life-years lost	64.0 (11.7 - 131.4)	57.3 (10.4 - 120.7)	-6.7 (-30.5 - 9.3)
cost	11688.2 (5594.7 - 21367.9)	12666.3 (6357.3 - 21679.1)	978.1 (-7407.9 - 9219.3)

Table A6 Indonesia

Quantity per 100 children with presumptive TB (unless stated):	Standard of care	Intervention	Difference
children with true TB	22.8 (4.4 - 42.5)	22.8 (4.4 - 42.5)	0.0 (0.0 - 0.0)
assessments	221.3 (204.3 - 239.6)	271.1 (248.7 - 292.5)	49.8 (37.8 - 60.8)
bacteriological investigations	25.5 (8.1 - 43.9)	105.5 (90.4 - 113.9)	80.0 (56.7 - 100.3)
anti-TB treatments (ATT)	22.1 (11.2 - 34.2)	26.0 (14.1 - 38.5)	4.0 (-0.4 - 9.9)
ATT initiated at PHC	70.8 (61.5 - 78.1)	82.7 (71.8 - 89.5)	11.9 (7.2 - 16.4)
percent of true-positive receiving ATT	60.3 (48.2 - 71.4)	71.8 (65.9 - 77.3)	11.5 (1.8 - 23.1)
percent of ATT bacteriologically confirmed	5.0 (1.2 - 11.2)	28.7 (17.8 - 39.1)	23.6 (13.1 - 34.3)
percent of ATT false-positive	40.8 (16.7 - 79.7)	40.5 (17.4 - 79.3)	-0.3 (-4.1 - 5.0)
referrals, inc. self-referrals	38.2 (27.9 - 48.3)	17.4 (12.9 - 23.8)	-20.8 (-29.8 - -10.8)
deaths	2.7 (0.5 - 5.4)	2.3 (0.4 - 4.6)	-0.4 (-1.1 - 0.0)
life-years lost	77.4 (14.7 - 155.1)	66.5 (12.4 - 132.3)	-10.9 (-30.9 - 0.1)
cost	10894.9 (6314.8 - 17164.5)	12513.6 (7709.2 - 18868.1)	1618.7 (-4945.3 - 8121.1)

Mafirakureva N, *et al. BMJ Open* 2022; 12:e058388. doi: 10.1136/bmjopen-2021-058388

Age 0-4 years

Table A7 Ethiopia

Quantity per 100 children with presumptive TB (unless stated):	Standard of care	Intervention	Difference
children with true TB	22.8 (4.4 - 42.5)	22.8 (4.4 - 42.5)	0.0 (0.0 - 0.0)
assessments	235.5 (209.9 - 262.1)	264.5 (244.8 - 284.9)	29.0 (13.7 - 47.9)
bacteriological investigations	3.7 (2.3 - 5.5)	98.6 (86.2 - 110.5)	94.8 (82.5 - 106.6)
anti-TB treatments (ATT)	25.5 (12.3 - 39.3)	22.6 (11.0 - 34.7)	-3.0 (-6.9 - 0.4)
ATT initiated at PHC	62.6 (53.4 - 72.7)	83.6 (71.9 - 93.1)	21.0 (11.6 - 30.7)
percent of true-positive receiving ATT	69.3 (57.6 - 79.5)	64.8 (55.8 - 73.1)	-4.5 (-10.6 - 2.6)
percent of ATT bacteriologically confirmed	0.6 (0.3 - 1.0)	28.8 (17.8 - 39.7)	28.2 (17.4 - 38.9)
percent of ATT false-positive	40.7 (14.3 - 80.7)	37.8 (14.0 - 77.9)	-2.9 (-6.6 - 1.8)
referrals, inc. self-referrals	56.0 (35.1 - 73.4)	9.8 (2.1 - 22.3)	-46.2 (-64.8 - -25.7)
deaths	3.4 (0.6 - 7.2)	3.9 (0.7 - 7.8)	0.4 (-0.2 - 1.4)
life-years lost	94.9 (17.3 - 197.9)	106.6 (19.9 - 213.9)	11.7 (-6.3 - 37.5)
cost	13498.5 (6292.1 - 24528.2)	11443.3 (5638.1 - 19702.2)	-2055.2 (-11147.7 - 5839.4)

Mafirakureva N, *et al. BMJ Open* 2022; 12:e058388. doi: 10.1136/bmjopen-2021-058388

Table A8 Indonesia

Quantity per 100 children with presumptive TB (unless stated):	Standard of care	Intervention	Difference
children with true TB	22.8 (4.4 - 42.5)	22.8 (4.4 - 42.5)	0.0 (0.0 - 0.0)
assessments	228.3 (208.1 - 249.0)	274.5 (252.9 - 294.7)	46.2 (34.0 - 55.8)
bacteriological investigations	3.5 (2.2 - 5.2)	106.0 (90.9 - 114.3)	102.5 (87.3 - 110.8)
anti-TB treatments (ATT)	24.4 (11.7 - 37.5)	23.8 (11.8 - 36.2)	-0.6 (-3.0 - 1.4)
ATT initiated at PHC	67.8 (59.3 - 75.2)	82.1 (71.5 - 88.8)	14.2 (9.1 - 18.9)
percent of true-positive receiving ATT	66.6 (56.3 - 76.0)	67.0 (58.5 - 74.8)	0.4 (-3.7 - 4.8)
percent of ATT bacteriologically confirmed	0.5 (0.3 - 1.0)	28.7 (17.6 - 39.6)	28.1 (17.2 - 38.9)
percent of ATT false-positive	40.3 (14.1 - 80.3)	38.8 (14.6 - 78.5)	-1.5 (-4.8 - 3.1)
referrals, inc. self-referrals	47.5 (38.8 - 55.3)	17.9 (13.4 - 24.4)	-29.6 (-35.9 - -22.1)
deaths	3.7 (0.7 - 7.5)	3.7 (0.7 - 7.3)	-0.0 (-0.5 - 0.4)
life-years lost	104.9 (19.4 - 214.5)	103.9 (19.5 - 208.3)	-1.0 (-15.5 - 11.6)
cost	11896.1 (6406.6 - 19196.8)	12176.0 (7451.0 - 18467.1)	279.8 (-7399.6 - 7997.8)

Mafirakureva N, *et al. BMJ Open* 2022; 12:e058388. doi: 10.1136/bmjopen-2021-058388

Age 5-14 years

Table A9 Ethiopia

**Quantity per 100 children with presumptive TB (unless stated):**	**Standard of care**	**Intervention**	**Difference**
children with true TB	22.8 (4.4 - 42.5)	22.8 (4.4 - 42.5)	0.0 (0.0 - 0.0)
assessments	217.8 (198.9 - 241.4)	258.0 (236.2 - 280.4)	40.3 (25.0 - 58.7)
bacteriological investigations	50.4 (13.8 - 85.9)	98.1 (85.4 - 109.7)	47.6 (10.1 - 85.7)
anti-TB treatments (ATT)	20.3 (8.1 - 35.7)	27.2 (14.0 - 40.7)	6.9 (-2.2 - 18.6)
ATT initiated at PHC	70.2 (58.8 - 81.9)	84.2 (72.7 - 93.4)	14.1 (4.7 - 23.9)
percent of true-positive receiving ATT	55.1 (32.5 - 75.7)	75.1 (67.2 - 82.0)	20.0 (0.5 - 43.3)
percent of ATT bacteriologically confirmed	12.7 (2.1 - 35.4)	29.4 (16.9 - 41.7)	16.6 (-5.9 - 33.0)
percent of ATT false-positive	40.6 (15.3 - 80.4)	40.2 (16.0 - 79.7)	-0.4 (-5.3 - 6.7)
referrals, inc. self-referrals	33.1 (12.2 - 55.5)	9.2 (1.9 - 21.2)	-23.8 (-47.1 - -1.3)
deaths	1.6 (0.3 - 3.6)	1.0 (0.2 - 2.1)	-0.6 (-1.9 - -0.0)
life-years lost	45.5 (8.0 - 100.0)	27.7 (5.1 - 57.5)	-17.8 (-51.9 - -0.3)
cost	10603.2 (4477.3 - 20733.1)	13401.2 (6616.6 - 22999.5)	2798.0 (-6051.3 - 11513.5)

Table A10 Indonesia

Quantity per 100 children with presumptive TB (unless stated):	Standard of care	Intervention	Difference
children with true TB	22.8 (4.4 - 42.5)	22.8 (4.4 - 42.5)	0.0 (0.0 - 0.0)
assessments	213.8 (198.0 - 233.1)	267.4 (243.3 - 290.7)	53.6 (39.5 - 69.5)
bacteriological investigations	48.9 (13.3 - 84.6)	105.1 (89.9 - 113.6)	56.2 (18.0 - 93.5)
anti-TB treatments (ATT)	19.6 (7.9 - 34.4)	28.5 (15.0 - 42.2)	8.8 (0.5 - 20.4)
ATT initiated at PHC	75.1 (63.7 - 85.5)	83.2 (72.2 - 90.3)	8.1 (0.7 - 14.4)
percent of true-positive receiving ATT	53.6 (31.5 - 73.0)	76.9 (69.6 - 83.3)	23.3 (4.9 - 45.9)
percent of ATT bacteriologically confirmed	12.2 (1.9 - 34.3)	29.1 (16.6 - 41.3)	16.9 (-4.8 - 33.0)
percent of ATT false-positive	40.2 (15.1 - 79.9)	41.4 (16.8 - 80.4)	1.2 (-3.2 - 8.0)
referrals, inc. self-referrals	28.3 (11.5 - 44.1)	16.9 (12.3 - 23.3)	-11.4 (-26.4 - 5.6)
deaths	1.7 (0.3 - 3.7)	0.9 (0.2 - 2.0)	-0.8 (-2.0 - -0.1)
life-years lost	48.3 (8.7 - 105.1)	26.9 (5.0 - 55.6)	-21.4 (-57.9 - -2.1)
cost	9830.4 (5352.0 - 15979.3)	12871.7 (7911.6 - 19463.3)	3041.3 (-3390.0 - 8592.6)

Mafirakureva N, *et al. BMJ Open* 2022; 12:e058388. doi: 10.1136/bmjopen-2021-058388

Results for Xpert baseline sensitivity analysis

Age-specific results

All ages: 0-14 years

Table A11 Ethiopia

Quantity per 100 children with presumptive TB (unless stated):	Standard of care	Intervention	Difference
children with true TB	45.5 (8.7 - 85.0)	45.5 (8.7 - 85.0)	0.0 (0.0 - 0.0)
assessments	199.4 (168.3 - 230.2)	246.2 (207.3 - 283.3)	46.8 (33.2 - 59.9)
bacteriological investigations	30.7 (8.7 - 57.5)	102.3 (86.8 - 112.0)	71.7 (41.5 - 96.3)
anti-TB treatments (ATT)	34.3 (14.2 - 56.8)	40.3 (17.6 - 64.4)	6.0 (0.1 - 15.0)
ATT initiated at PHC	73.6 (63.9 - 81.8)	81.9 (71.6 - 89.5)	8.4 (3.2 - 13.2)
percent of true-positive receiving ATT	62.6 (51.4 - 72.7)	73.0 (66.7 - 78.8)	10.5 (1.6 - 22.0)
percent of ATT bacteriologically confirmed	14.0 (3.0 - 32.6)	32.8 (20.7 - 44.1)	18.8 (-0.2 - 34.6)
percent of ATT false-positive	21.3 (2.7 - 63.8)	21.9 (2.9 - 64.9)	0.6 (-2.4 - 5.1)
referrals, inc. self-referrals	29.5 (17.0 - 42.9)	13.8 (8.0 - 21.0)	-15.6 (-25.8 - -4.9)
deaths	4.6 (0.9 - 9.4)	3.9 (0.7 - 8.3)	-0.7 (-2.0 - 0.0)
life-years lost	127.7 (23.8 - 260.7)	108.7 (19.7 - 228.5)	-19.0 (-55.1 - 0.1)
cost	16678.2 (6843.1 - 32205.5)	19297.7 (8413.8 - 35444.7)	2619.5 (-9141.3 - 14513.1)

Mafirakureva N, *et al. BMJ Open* 2022; 12:e058388. doi: 10.1136/bmjopen-2021-058388

Table A12 Indonesia

Quantity per 100 children with presumptive TB (unless stated):	Standard of care	Intervention	Difference
children with true TB	45.5 (8.7 - 85.0)	45.5 (8.7 - 85.0)	0.0 (0.0 - 0.0)
assessments	202.3 (170.6 - 232.9)	249.9 (211.2 - 286.5)	47.7 (34.7 - 59.6)
bacteriological investigations	24.7 (7.8 - 43.2)	103.0 (87.5 - 112.6)	78.2 (54.5 - 98.4)
anti-TB treatments (ATT)	35.1 (14.8 - 57.5)	39.5 (17.1 - 63.3)	4.4 (-0.4 - 11.1)
ATT initiated at PHC	74.4 (64.7 - 81.7)	84.4 (73.2 - 91.2)	10.0 (5.4 - 14.6)
percent of true-positive receiving ATT	63.8 (54.3 - 72.8)	71.8 (65.9 - 77.3)	8.0 (0.6 - 16.6)
percent of ATT bacteriologically confirmed	10.7 (2.6 - 22.7)	32.5 (20.9 - 43.4)	21.7 (7.7 - 35.3)
percent of ATT false-positive	21.6 (2.7 - 64.4)	21.8 (2.9 - 64.6)	0.2 (-2.8 - 4.2)
referrals, inc. self-referrals	33.0 (21.5 - 45.5)	14.5 (8.6 - 21.7)	-18.4 (-27.6 - -9.5)
deaths	5.2 (1.0 - 10.3)	4.7 (0.9 - 9.3)	-0.5 (-1.6 - 0.1)
life-years lost	147.8 (28.1 - 294.2)	133.1 (24.7 - 264.6)	-14.7 (-45.7 - 3.5)
cost	12852.5 (7260.6 - 20698.7)	14525.7 (8603.6 - 22403.0)	1673.3 (-5630.9 - 8936.4)

Mafirakureva N, *et al. BMJ Open* 2022; 12:e058388. doi: 10.1136/bmjopen-2021-058388

Age 0-4 years

Table A13 Ethiopia

Quantity per 100 children with presumptive TB (unless stated):	Standard of care	Intervention	Difference
children with true TB	45.5 (8.7 - 85.0)	45.5 (8.7 - 85.0)	0.0 (0.0 - 0.0)
assessments	208.0 (172.6 - 241.6)	252.9 (217.5 - 286.1)	44.9 (33.1 - 55.2)
bacteriological investigations	3.4 (2.1 - 4.9)	103.1 (87.6 - 112.5)	99.8 (84.3 - 109.1)
anti-TB treatments (ATT)	36.7 (15.4 - 59.9)	36.4 (15.2 - 59.3)	-0.3 (-2.9 - 2.5)
ATT initiated at PHC	68.4 (59.7 - 75.8)	81.0 (70.9 - 88.4)	12.6 (7.9 - 17.2)
percent of true-positive receiving ATT	66.5 (56.2 - 75.7)	66.8 (58.4 - 74.6)	0.3 (-3.7 - 4.8)
percent of ATT bacteriologically confirmed	1.1 (0.5 - 1.7)	31.4 (20.4 - 42.2)	30.3 (19.7 - 41.0)
percent of ATT false-positive	21.7 (2.4 - 65.7)	20.7 (2.4 - 63.4)	-1.0 (-4.1 - 1.9)
referrals, inc. self-referrals	40.1 (28.3 - 51.7)	14.7 (9.1 - 21.8)	-25.4 (-33.2 - -17.4)
deaths	7.4 (1.4 - 15.1)	7.3 (1.4 - 14.7)	-0.1 (-1.1 - 0.8)
life-years lost	204.3 (37.6 - 416.1)	202.4 (37.5 - 405.7)	-1.8 (-29.6 - 21.8)
cost	18004.2 (7149.6 - 35253.8)	17667.9 (7614.0 - 32685.5)	-336.2 (-13382.6 - 11963.8)

Mafirakureva N, *et al. BMJ Open* 2022; 12:e058388. doi: 10.1136/bmjopen-2021-058388

Table A14 Indonesia

Quantity per 100 children with presumptive TB (unless stated):	Standard of care	Intervention	Difference
children with true TB	45.5 (8.7 - 85.0)	45.5 (8.7 - 85.0)	0.0 (0.0 - 0.0)
assessments	209.2 (173.5 - 242.9)	255.2 (219.1 - 289.2)	46.1 (34.0 - 56.2)
bacteriological investigations	3.4 (2.1 - 5.0)	103.6 (88.2 - 113.0)	100.2 (84.9 - 109.5)
anti-TB treatments (ATT)	37.0 (15.5 - 60.7)	36.5 (15.2 - 59.6)	-0.5 (-3.1 - 2.2)
ATT initiated at PHC	70.3 (61.3 - 77.5)	83.6 (72.6 - 90.3)	13.3 (8.4 - 17.9)
percent of true-positive receiving ATT	67.0 (56.6 - 76.3)	67.0 (58.5 - 74.8)	0.1 (-3.9 - 4.5)
percent of ATT bacteriologically confirmed	1.1 (0.5 - 1.7)	31.4 (20.4 - 42.1)	30.3 (19.6 - 40.9)
percent of ATT false-positive	21.8 (2.4 - 65.7)	20.7 (2.4 - 63.7)	-1.0 (-4.2 - 1.9)
referrals, inc. self-referrals	41.5 (29.2 - 53.1)	15.3 (9.5 - 22.4)	-26.3 (-34.4 - -17.9)
deaths	7.3 (1.4 - 15.0)	7.3 (1.4 - 14.6)	-0.0 (-1.0 - 0.9)
life-years lost	208.1 (38.4 - 426.3)	207.8 (39.0 - 416.6)	-0.3 (-28.4 - 25.5)
cost	13704.3 (7307.0 - 22423.2)	14090.7 (8344.4 - 21727.0)	386.4 (-8103.9 - 8967.9)

Age 5-14 years

Table A15 Ethiopia

Quantity per 100 children with presumptive TB (unless stated):	Standard of care	Intervention	Difference
children with true TB	45.5 (8.7 - 85.0)	45.5 (8.7 - 85.0)	0.0 (0.0 - 0.0)
assessments	194.2 (164.3 - 224.2)	242.2 (200.8 - 281.7)	48.0 (31.6 - 65.4)
bacteriological investigations	47.1 (12.5 - 82.7)	101.9 (86.2 - 111.6)	54.8 (16.5 - 91.1)
anti-TB treatments (ATT)	32.9 (12.5 - 56.8)	42.6 (18.5 - 67.8)	9.7 (0.9 - 22.5)
ATT initiated at PHC	77.2 (66.1 - 87.6)	82.4 (71.8 - 90.2)	5.2 (-2.2 - 11.8)
percent of true-positive receiving ATT	60.2 (44.6 - 74.9)	76.8 (69.4 - 83.1)	16.5 (3.3 - 32.6)
percent of ATT bacteriologically confirmed	23.5 (4.2 - 56.3)	33.7 (20.3 - 46.1)	10.2 (-22.8 - 33.6)
percent of ATT false-positive	20.7 (2.5 - 64.1)	22.4 (2.8 - 66.2)	1.7 (-1.9 - 8.3)
referrals, inc. self-referrals	23.0 (8.8 - 39.1)	13.3 (7.3 - 20.5)	-9.7 (-22.7 - 4.4)
deaths	3.0 (0.6 - 6.3)	1.9 (0.3 - 3.9)	-1.1 (-2.9 - -0.1)
life-years lost	81.8 (15.2 - 172.9)	52.5 (9.6 - 107.9)	-29.3 (-80.4 - -2.9)
cost	15884.6 (6196.1 - 31393.0)	20277.4 (8872.7 - 37127.0)	4392.8 (-7520.5 - 16511.0)

Table A16 Indonesia

Quantity per 100 children with presumptive TB (unless stated):	Standard of care	Intervention	Difference
children with true TB	45.5 (8.7 - 85.0)	45.5 (8.7 - 85.0)	0.0 (0.0 - 0.0)
assessments	194.9 (164.8 - 225.1)	244.3 (202.0 - 284.1)	49.4 (32.8 - 67.1)
bacteriological investigations	47.3 (12.7 - 83.3)	102.3 (86.7 - 112.2)	54.9 (16.6 - 91.6)
anti-TB treatments (ATT)	33.1 (12.6 - 57.0)	42.7 (18.5 - 68.1)	9.7 (0.8 - 22.5)
ATT initiated at PHC	79.6 (67.8 - 90.1)	85.1 (73.6 - 92.2)	5.5 (-2.4 - 12.6)
percent of true-positive receiving ATT	60.5 (44.7 - 75.1)	76.9 (69.6 - 83.3)	16.5 (3.1 - 32.6)
percent of ATT bacteriologically confirmed	23.5 (4.3 - 56.3)	33.7 (20.2 - 46.1)	10.2 (-22.6 - 33.6)
percent of ATT false-positive	20.8 (2.5 - 64.3)	22.4 (2.8 - 66.3)	1.7 (-1.9 - 8.3)
referrals, inc. self-referrals	23.8 (9.0 - 40.3)	13.8 (7.5 - 21.2)	-10.1 (-23.7 - 4.3)
deaths	2.9 (0.6 - 6.3)	1.9 (0.4 - 3.9)	-1.1 (-2.9 - -0.1)
life-years lost	83.9 (15.8 - 178.7)	53.9 (10.0 - 111.1)	-30.1 (-83.3 - -2.7)
cost	11945.9 (6523.9 - 19684.3)	14987.1 (8815.0 - 23229.9)	3041.3 (-4161.7 - 9508.2)

Mafirakureva N, *et al. BMJ Open* 2022; 12:e058388. doi: 10.1136/bmjopen-2021-058388

Results for 0% discount rate sensitivity analysis

Age-specific results

All ages: 0-14 years

Table A17 Ethiopia

Quantity per 100 children with presumptive TB (unless stated):	Standard of care	Intervention	Difference
children with true TB	45.5 (8.7 - 85.0)	45.5 (8.7 - 85.0)	0.0 (0.0 - 0.0)
assessments	201.8 (171.8 - 230.9)	246.2 (207.3 - 283.3)	44.4 (29.5 - 58.1)
bacteriological investigations	30.7 (8.7 - 57.5)	102.3 (86.8 - 112.0)	71.7 (41.5 - 96.3)
anti-TB treatments (ATT)	32.2 (13.2 - 54.5)	40.3 (17.6 - 64.4)	8.1 (0.6 - 20.3)
ATT initiated at PHC	71.8 (62.3 - 79.6)	81.9 (71.6 - 89.5)	10.1 (5.8 - 14.2)
percent of true-positive receiving ATT	58.3 (43.0 - 71.1)	73.0 (66.7 - 78.8)	14.7 (2.8 - 30.5)
percent of ATT bacteriologically confirmed	8.0 (1.7 - 19.8)	32.8 (20.7 - 44.1)	24.8 (10.6 - 37.8)
percent of ATT false-positive	21.9 (2.8 - 64.6)	21.9 (2.9 - 64.9)	0.0 (-3.0 - 4.0)
referrals, inc. self-referrals	29.5 (17.0 - 42.9)	13.8 (8.0 - 21.0)	-15.6 (-25.8 - -4.9)
deaths	4.9 (0.9 - 10.0)	3.9 (0.7 - 8.3)	-1.0 (-2.8 - -0.1)
life-years lost	339.1 (62.7 - 692.0)	271.6 (49.2 - 570.9)	-67.5 (-189.8 - -4.1)
cost	15729.4 (6368.3 - 31027.5)	19297.7 (8413.8 - 35444.7)	3568.3 (-8472.2 - 16311.6)

Table A18 Indonesia

**Quantity per 100 children with presumptive TB (unless stated):**	**Standard of care**	**Intervention**	**Difference**
children with true TB	45.5 (8.7 - 85.0)	45.5 (8.7 - 85.0)	0.0 (0.0 - 0.0)
assessments	204.2 (173.4 - 233.5)	249.9 (211.2 - 286.5)	45.7 (31.9 - 58.0)
bacteriological investigations	24.7 (7.8 - 43.2)	103.0 (87.5 - 112.6)	78.2 (54.5 - 98.4)
anti-TB treatments (ATT)	33.3 (14.1 - 55.3)	39.5 (17.1 - 63.3)	6.2 (0.1 - 15.2)
ATT initiated at PHC	73.0 (63.2 - 80.3)	84.4 (73.2 - 91.2)	11.3 (7.1 - 15.4)
percent of true-positive receiving ATT	60.3 (48.2 - 71.4)	71.8 (65.9 - 77.3)	11.5 (1.8 - 23.1)
percent of ATT bacteriologically confirmed	5.9 (1.4 - 12.9)	32.5 (20.9 - 43.4)	26.6 (14.9 - 38.2)
percent of ATT false-positive	22.0 (2.8 - 65.1)	21.8 (2.9 - 64.6)	-0.3 (-3.5 - 3.5)
referrals, inc. self-referrals	33.0 (21.5 - 45.5)	14.5 (8.6 - 21.7)	-18.4 (-27.6 - -9.6)
deaths	5.4 (1.0 - 10.9)	4.7 (0.9 - 9.3)	-0.8 (-2.2 - 0.0)
life-years lost	391.6 (74.2 - 784.5)	336.7 (62.6 - 669.4)	-55.0 (-156.1 - 0.6)
cost	12508.1 (7056.4 - 20279.0)	14525.7 (8603.6 - 22403.0)	2017.6 (-5421.3 - 9470.6)

Age 0-4 years

Table A19 Ethiopia

**Quantity per 100 children with presumptive TB (unless stated):**	**Standard of care**	**Intervention**	**Difference**
children with true TB	45.5 (8.7 - 85.0)	45.5 (8.7 - 85.0)	0.0 (0.0 - 0.0)
assessments	208.1 (172.8 - 241.7)	252.9 (217.5 - 286.1)	44.7 (32.9 - 55.1)
bacteriological investigations	3.4 (2.1 - 4.9)	103.1 (87.6 - 112.5)	99.8 (84.3 - 109.1)
anti-TB treatments (ATT)	36.6 (15.3 - 59.7)	36.4 (15.2 - 59.3)	-0.2 (-2.8 - 2.7)
ATT initiated at PHC	68.3 (59.6 - 75.7)	81.0 (70.9 - 88.4)	12.7 (8.0 - 17.4)
percent of true-positive receiving ATT	66.2 (55.9 - 75.4)	66.8 (58.4 - 74.6)	0.7 (-3.4 - 5.1)
percent of ATT bacteriologically confirmed	0.6 (0.3 - 1.0)	31.4 (20.4 - 42.2)	30.8 (19.9 - 41.4)
percent of ATT false-positive	21.7 (2.4 - 65.8)	20.7 (2.4 - 63.4)	-1.0 (-4.2 - 1.9)
referrals, inc. self-referrals	40.1 (28.3 - 51.7)	14.7 (9.1 - 21.8)	-25.4 (-33.2 - -17.4)
deaths	7.5 (1.4 - 15.2)	7.3 (1.4 - 14.7)	-0.1 (-1.2 - 0.7)
life-years lost	514.5 (94.8 - 1046.0)	505.9 (93.8 - 1013.6)	-8.6 (-79.9 - 49.8)
cost	17934.1 (7124.0 - 35159.0)	17667.9 (7614.0 - 32685.5)	-266.2 (-13326.6 - 12081.4)

Table A20 Indonesia

Quantity per 100 children with presumptive TB (unless stated):	Standard of care	Intervention	Difference
children with true TB	45.5 (8.7 - 85.0)	45.5 (8.7 - 85.0)	0.0 (0.0 - 0.0)
assessments	209.4 (173.8 - 242.9)	255.2 (219.1 - 289.2)	45.9 (33.8 - 56.0)
bacteriological investigations	3.4 (2.1 - 5.0)	103.6 (88.2 - 113.0)	100.2 (84.9 - 109.5)
anti-TB treatments (ATT)	36.8 (15.4 - 60.4)	36.5 (15.2 - 59.6)	-0.3 (-3.0 - 2.4)
ATT initiated at PHC	70.1 (61.1 - 77.4)	83.6 (72.6 - 90.3)	13.4 (8.6 - 18.1)
percent of true-positive receiving ATT	66.6 (56.3 - 76.0)	67.0 (58.5 - 74.8)	0.4 (-3.7 - 4.8)
percent of ATT bacteriologically confirmed	0.6 (0.3 - 1.0)	31.4 (20.4 - 42.1)	30.8 (20.0 - 41.3)
percent of ATT false-positive	21.8 (2.4 - 65.8)	20.7 (2.4 - 63.7)	-1.1 (-4.3 - 1.9)
referrals, inc. self-referrals	41.5 (29.2 - 53.1)	15.3 (9.5 - 22.4)	-26.3 (-34.4 - -17.9)
deaths	7.4 (1.4 - 15.1)	7.3 (1.4 - 14.6)	-0.1 (-1.1 - 0.8)
life-years lost	530.6 (97.9 - 1085.3)	525.6 (98.7 - 1053.7)	-5.0 (-78.3 - 58.6)
cost	13672.3 (7286.9 - 22370.9)	14090.7 (8344.4 - 21727.0)	418.4 (-8064.8 - 9011.5)

Mafirakureva N, *et al. BMJ Open* 2022; 12:e058388. doi: 10.1136/bmjopen-2021-058388

Age 5-14 years

Table A21 Ethiopia

**Quantity per 100 children with presumptive TB (unless stated):**	**Standard of care**	**Intervention**	**Difference**
children with true TB	45.5 (8.7 - 85.0)	45.5 (8.7 - 85.0)	0.0 (0.0 - 0.0)
assessments	197.9 (169.6 - 225.2)	242.2 (200.8 - 281.7)	44.3 (24.8 - 62.7)
bacteriological investigations	47.1 (12.5 - 82.7)	101.9 (86.2 - 111.6)	54.8 (16.5 - 91.1)
anti-TB treatments (ATT)	29.6 (10.6 - 53.8)	42.6 (18.5 - 67.8)	13.0 (1.5 - 30.3)
ATT initiated at PHC	74.6 (63.8 - 84.3)	82.4 (71.8 - 90.2)	7.8 (2.1 - 12.9)
percent of true-positive receiving ATT	53.6 (31.9 - 72.9)	76.8 (69.4 - 83.1)	23.2 (5.1 - 45.4)
percent of ATT bacteriologically confirmed	14.5 (2.4 - 39.5)	33.7 (20.3 - 46.1)	19.2 (-5.9 - 37.4)
percent of ATT false-positive	21.6 (2.6 - 65.3)	22.4 (2.8 - 66.2)	0.8 (-2.5 - 6.2)
referrals, inc. self-referrals	23.1 (8.8 - 39.1)	13.3 (7.3 - 20.5)	-9.7 (-22.7 - 4.4)
deaths	3.4 (0.6 - 7.4)	1.9 (0.3 - 3.9)	-1.5 (-4.0 - -0.2)
life-years lost	234.0 (42.2 - 507.3)	131.2 (24.1 - 269.5)	-102.8 (-278.9 - -10.6)
cost	14407.8 (5303.7 - 29936.5)	20277.4 (8872.7 - 37127.0)	5869.6 (-6634.5 - 19361.5)

Mafirakureva N, *et al. BMJ Open* 2022; 12:e058388. doi: 10.1136/bmjopen-2021-058388

Table A22 Indonesia

**Quantity per 100 children with presumptive TB (unless stated):**	**Standard of care**	**Intervention**	**Difference**
children with true TB	45.5 (8.7 - 85.0)	45.5 (8.7 - 85.0)	0.0 (0.0 - 0.0)
assessments	198.8 (170.2 - 226.2)	244.3 (202.0 - 284.1)	45.5 (25.6 - 64.3)
bacteriological investigations	47.3 (12.7 - 83.3)	102.3 (86.7 - 112.2)	54.9 (16.6 - 91.6)
anti-TB treatments (ATT)	29.6 (10.5 - 54.2)	42.7 (18.5 - 68.1)	13.1 (1.4 - 30.8)
ATT initiated at PHC	77.2 (65.5 - 87.0)	85.1 (73.6 - 92.2)	7.9 (1.5 - 13.5)
percent of true-positive receiving ATT	53.6 (31.5 - 73.0)	76.9 (69.6 - 83.3)	23.3 (4.9 - 45.9)
percent of ATT bacteriologically confirmed	14.1 (2.3 - 38.3)	33.7 (20.2 - 46.1)	19.5 (-4.9 - 37.4)
percent of ATT false-positive	21.7 (2.6 - 65.3)	22.4 (2.8 - 66.3)	0.8 (-2.6 - 6.1)
referrals, inc. self-referrals	23.9 (9.1 - 40.3)	13.8 (7.5 - 21.2)	-10.1 (-23.7 - 4.3)
deaths	3.4 (0.6 - 7.4)	1.9 (0.4 - 3.9)	-1.5 (-4.1 - -0.1)
life-years lost	244.3 (44.1 - 531.9)	136.3 (25.4 - 281.0)	-108.0 (-292.8 - -10.8)
cost	11270.2 (6013.6 - 18958.7)	14987.1 (8815.0 - 23229.9)	3716.9 (-3812.4 - 10646.6)

Mafirakureva N, *et al*. *BMJ Open* 2022; 12:e058388. doi: 10.1136/bmjopen-2021-058388

Results for 5% discount rate sensitivity analysis

Age-specific results

All ages: 0-14 years

Table A23 Ethiopia

Quantity per 100 children with presumptive TB (unless stated):	Standard of care	Intervention	Difference
children with true TB	45.5 (8.7 - 85.0)	45.5 (8.7 - 85.0)	0.0 (0.0 - 0.0)
assessments	201.8 (171.8 - 230.9)	246.2 (207.3 - 283.3)	44.4 (29.5 - 58.1)
bacteriological investigations	30.7 (8.7 - 57.5)	102.3 (86.8 - 112.0)	71.7 (41.5 - 96.3)
anti-TB treatments (ATT)	32.2 (13.2 - 54.5)	40.3 (17.6 - 64.4)	8.1 (0.6 - 20.3)
ATT initiated at PHC	71.8 (62.3 - 79.6)	81.9 (71.6 - 89.5)	10.1 (5.8 - 14.2)
percent of true-positive receiving ATT	58.3 (43.0 - 71.1)	73.0 (66.7 - 78.8)	14.7 (2.8 - 30.5)
percent of ATT bacteriologically confirmed	8.0 (1.7 - 19.8)	32.8 (20.7 - 44.1)	24.8 (10.6 - 37.8)
percent of ATT false-positive	21.9 (2.8 - 64.6)	21.9 (2.9 - 64.9)	0.0 (-3.0 - 4.0)
referrals, inc. self-referrals	29.5 (17.0 - 42.9)	13.8 (8.0 - 21.0)	-15.6 (-25.8 - -4.9)
deaths	4.9 (0.9 - 10.0)	3.9 (0.7 - 8.3)	-1.0 (-2.8 - -0.1)
life-years lost	89.0 (16.5 - 181.7)	71.3 (12.9 - 149.9)	-17.7 (-49.8 - -1.1)
cost	15729.4 (6368.3 - 31027.5)	19297.7 (8413.8 - 35444.7)	3568.3 (-8472.2 - 16311.6)

Table A24 Indonesia

**Quantity per 100 children with presumptive TB (unless stated):**	**Standard of care**	**Intervention**	**Difference**
children with true TB	45.5 (8.7 - 85.0)	45.5 (8.7 - 85.0)	0.0 (0.0 - 0.0)
assessments	204.2 (173.4 - 233.5)	249.9 (211.2 - 286.5)	45.7 (31.9 - 58.0)
bacteriological investigations	24.7 (7.8 - 43.2)	103.0 (87.5 - 112.6)	78.2 (54.5 - 98.4)
anti-TB treatments (ATT)	33.3 (14.1 - 55.3)	39.5 (17.1 - 63.3)	6.2 (0.1 - 15.2)
ATT initiated at PHC	73.0 (63.2 - 80.3)	84.4 (73.2 - 91.2)	11.3 (7.1 - 15.4)
percent of true-positive receiving ATT	60.3 (48.2 - 71.4)	71.8 (65.9 - 77.3)	11.5 (1.8 - 23.1)
percent of ATT bacteriologically confirmed	5.9 (1.4 - 12.9)	32.5 (20.9 - 43.4)	26.6 (14.9 - 38.2)
percent of ATT false-positive	22.0 (2.8 - 65.1)	21.8 (2.9 - 64.6)	-0.3 (-3.5 - 3.5)
referrals, inc. self-referrals	33.0 (21.5 - 45.5)	14.5 (8.6 - 21.7)	-18.4 (-27.6 - -9.6)
deaths	5.4 (1.0 - 10.9)	4.7 (0.9 - 9.3)	-0.8 (-2.2 - 0.0)
life-years lost	101.1 (19.2 - 202.4)	86.9 (16.2 - 172.7)	-14.2 (-40.3 - 0.2)
cost	12508.1 (7056.4 - 20279.0)	14525.7 (8603.6 - 22403.0)	2017.6 (-5421.3 - 9470.6)

Mafirakureva N, *et al. BMJ Open* 2022; 12:e058388. doi: 10.1136/bmjopen-2021-058388

Age 0-4 years

Table A25 Ethiopia

**Quantity per 100 children with presumptive TB (unless stated):**	**Standard of care**	**Intervention**	**Difference**
children with true TB	45.5 (8.7 - 85.0)	45.5 (8.7 - 85.0)	0.0 (0.0 - 0.0)
assessments	208.1 (172.8 - 241.7)	252.9 (217.5 - 286.1)	44.7 (32.9 - 55.1)
bacteriological investigations	3.4 (2.1 - 4.9)	103.1 (87.6 - 112.5)	99.8 (84.3 - 109.1)
anti-TB treatments (ATT)	36.6 (15.3 - 59.7)	36.4 (15.2 - 59.3)	-0.2 (-2.8 - 2.7)
ATT initiated at PHC	68.3 (59.6 - 75.7)	81.0 (70.9 - 88.4)	12.7 (8.0 - 17.4)
percent of true-positive receiving ATT	66.2 (55.9 - 75.4)	66.8 (58.4 - 74.6)	0.7 (-3.4 - 5.1)
percent of ATT bacteriologically confirmed	0.6 (0.3 - 1.0)	31.4 (20.4 - 42.2)	30.8 (19.9 - 41.4)
percent of ATT false-positive	21.7 (2.4 - 65.8)	20.7 (2.4 - 63.4)	-1.0 (-4.2 - 1.9)
referrals, inc. self-referrals	40.1 (28.3 - 51.7)	14.7 (9.1 - 21.8)	-25.4 (-33.2 - -17.4)
deaths	7.5 (1.4 - 15.2)	7.3 (1.4 - 14.7)	-0.1 (-1.2 - 0.7)
life-years lost	135.1 (24.9 - 274.6)	132.8 (24.6 - 266.1)	-2.3 (-21.0 - 13.1)
cost	17934.1 (7124.0 - 35159.0)	17667.9 (7614.0 - 32685.5)	-266.2 (-13326.6 - 12081.4)

Mafirakureva N, *et al. BMJ Open* 2022; 12:e058388. doi: 10.1136/bmjopen-2021-058388

Table A26 Indonesia

Quantity per 100 children with presumptive TB (unless stated):	Standard of care	Intervention	Difference
children with true TB	45.5 (8.7 - 85.0)	45.5 (8.7 - 85.0)	0.0 (0.0 - 0.0)
assessments	209.4 (173.8 - 242.9)	255.2 (219.1 - 289.2)	45.9 (33.8 - 56.0)
bacteriological investigations	3.4 (2.1 - 5.0)	103.6 (88.2 - 113.0)	100.2 (84.9 - 109.5)
anti-TB treatments (ATT)	36.8 (15.4 - 60.4)	36.5 (15.2 - 59.6)	-0.3 (-3.0 - 2.4)
ATT initiated at PHC	70.1 (61.1 - 77.4)	83.6 (72.6 - 90.3)	13.4 (8.6 - 18.1)
percent of true-positive receiving ATT	66.6 (56.3 - 76.0)	67.0 (58.5 - 74.8)	0.4 (-3.7 - 4.8)
percent of ATT bacteriologically confirmed	0.6 (0.3 - 1.0)	31.4 (20.4 - 42.1)	30.8 (20.0 - 41.3)
percent of ATT false-positive	21.8 (2.4 - 65.8)	20.7 (2.4 - 63.7)	-1.1 (-4.3 - 1.9)
referrals, inc. self-referrals	41.5 (29.2 - 53.1)	15.3 (9.5 - 22.4)	-26.3 (-34.4 - -17.9)
deaths	7.4 (1.4 - 15.1)	7.3 (1.4 - 14.6)	-0.1 (-1.1 - 0.8)
life-years lost	136.9 (25.3 - 280.0)	135.6 (25.5 - 271.9)	-1.3 (-20.2 - 15.1)
cost	13672.3 (7286.9 - 22370.9)	14090.7 (8344.4 - 21727.0)	418.4 (-8064.8 - 9011.5)

Age 5-14 years

Table A27 Ethiopia

Quantity per 100 children with presumptive TB (unless stated):	Standard of care	Intervention	Difference
children with true TB	45.5 (8.7 - 85.0)	45.5 (8.7 - 85.0)	0.0 (0.0 - 0.0)
assessments	197.9 (169.6 - 225.2)	242.2 (200.8 - 281.7)	44.3 (24.8 - 62.7)
bacteriological investigations	47.1 (12.5 - 82.7)	101.9 (86.2 - 111.6)	54.8 (16.5 - 91.1)
anti-TB treatments (ATT)	29.6 (10.6 - 53.8)	42.6 (18.5 - 67.8)	13.0 (1.5 - 30.3)
ATT initiated at PHC	74.6 (63.8 - 84.3)	82.4 (71.8 - 90.2)	7.8 (2.1 - 12.9)
percent of true-positive receiving ATT	53.6 (31.9 - 72.9)	76.8 (69.4 - 83.1)	23.2 (5.1 - 45.4)
percent of ATT bacteriologically confirmed	14.5 (2.4 - 39.5)	33.7 (20.3 - 46.1)	19.2 (-5.9 - 37.4)
percent of ATT false-positive	21.6 (2.6 - 65.3)	22.4 (2.8 - 66.2)	0.8 (-2.5 - 6.2)
referrals, inc. self-referrals	23.1 (8.8 - 39.1)	13.3 (7.3 - 20.5)	-9.7 (-22.7 - 4.4)
deaths	3.4 (0.6 - 7.4)	1.9 (0.3 - 3.9)	-1.5 (-4.0 - -0.2)
life-years lost	61.4 (11.1 - 133.2)	34.5 (6.3 - 70.8)	-27.0 (-73.2 - -2.8)
cost	14407.8 (5303.7 - 29936.5)	20277.4 (8872.7 - 37127.0)	5869.6 (-6634.5 - 19361.5)

Table A28 Indonesia

**Quantity per 100 children with presumptive TB (unless stated):**	**Standard of care**	**Intervention**	**Difference**
children with true TB	45.5 (8.7 - 85.0)	45.5 (8.7 - 85.0)	0.0 (0.0 - 0.0)
assessments	198.8 (170.2 - 226.2)	244.3 (202.0 - 284.1)	45.5 (25.6 - 64.3)
bacteriological investigations	47.3 (12.7 - 83.3)	102.3 (86.7 - 112.2)	54.9 (16.6 - 91.6)
anti-TB treatments (ATT)	29.6 (10.5 - 54.2)	42.7 (18.5 - 68.1)	13.1 (1.4 - 30.8)
ATT initiated at PHC	77.2 (65.5 - 87.0)	85.1 (73.6 - 92.2)	7.9 (1.5 - 13.5)
percent of true-positive receiving ATT	53.6 (31.5 - 73.0)	76.9 (69.6 - 83.3)	23.3 (4.9 - 45.9)
percent of ATT bacteriologically confirmed	14.1 (2.3 - 38.3)	33.7 (20.2 - 46.1)	19.5 (-4.9 - 37.4)
percent of ATT false-positive	21.7 (2.6 - 65.3)	22.4 (2.8 - 66.3)	0.8 (-2.6 - 6.1)
referrals, inc. self-referrals	23.9 (9.1 - 40.3)	13.8 (7.5 - 21.2)	-10.1 (-23.7 - 4.3)
deaths	3.4 (0.6 - 7.4)	1.9 (0.4 - 3.9)	-1.5 (-4.1 - -0.1)
life-years lost	63.0 (11.4 - 137.2)	35.2 (6.5 - 72.5)	-27.9 (-75.5 - -2.8)
cost	11270.2 (6013.6 - 18958.7)	14987.1 (8815.0 - 23229.9)	3716.9 (-3812.4 - 10646.6)

Mafirakureva N, *et al. BMJ Open* 2022; 12:e058388. doi: 10.1136/bmjopen-2021-058388

Comparison of ICERs for sensitivity analyses

Table A29 ICERs by sensitivity analysis for each country

scenario	Ethiopia	Indonesia
basecase	132.2	93.8
Xpert SOC	137.8	114.8
Low prevalence	178.1	150.4
0% discount rate	54.8	38.3
5% discount rate	199.3	142.2

Mafirakureva N, *et al. BMJ Open* 2022; 12:e058388. doi: 10.1136/bmjopen-2021-058388
