# Supplementary material for: Xpert Ultra stool testing to diagnose tuberculosis in children in Ethiopia and Indonesia: a model-based cost-effectiveness analysis
Source: BMJ Open. 2022 Jul 1;12(7):e058388. doi: 10.1136/bmjopen-2021-058388 (PMC9252203; doi:10.1136/bmjopen-2021-058388)
Supplement: Author's manuscript [file bmjopen-2021-058388.draft_revisions.pdf]

BMJ Open

BMJ Open is committed to open peer review. As part of this commitment we make the peer review history of every article we publish publicly available.

When an article is published we post the peer reviewers' comments and the authors' responses online. We also post the versions of the paper that were used during peer review. These are the versions that the peer review comments apply to.

The versions of the paper that follow are the versions that were submitted during the peer review process. They are not the versions of record or the final published versions. They should not be cited or distributed as the published version of this manuscript.

BMJ Open is an open access journal and the full, final, typeset and author-corrected version of record of the manuscript is available on our site with no access controls, subscription charges or pay-per-view fees (http://bmjopen.bmj.com).

If you have any questions on BMJ Open's open peer review process please email info.bmjopen@bmj.com

BMJ Open

Xpert on stool to diagnose tuberculosis in children is cost-effective in Ethiopia and Indonesia: a model-based cost-effectiveness analysis

Journal:	*BMJ Open*
Manuscript ID	bmjopen-2021-058388
Article Type:	Original research
Date Submitted by the Author:	19-Oct-2021

Complete List of Authors:	Mafirakureva, Nyashadzaishe; The University of Sheffield, ScHARR<br>Klinkenberg, Eveline; Connect TB; Amsterdam University Medical Center, Global Health and Amsterdam Institute for Global Health and Development<br>Spruijt, Ineke; KNCV Tuberculosis Foundation<br>Levy, Jens; KNCV Tuberculosis Foundation<br>Adewo, Debebe S. ; The University of Sheffield, ScHARR<br>de Haas, Petra; KNCV Tuberculosis Foundation<br>Kaswandani, Nastiti; University of Indonesia Faculty of Medicine, Pediatric Department, RSCM Hospital<br>Bedru, Ahmed; KNCV Tuberculosis Foundation<br>Triasih, Rina; Universitas Gadjah Mada Fakultas Kedokteran, Department of Paediatrics<br>Gebremichel, Melaku; KNCV Tuberculosis Foundation<br>Dodd, PJ; The University of Sheffield, ScHARR<br>Tiemersma, Everdina; KNCV Tuberculosis Foundation, Technical Division

Keywords:	Health economics < HEALTH SERVICES ADMINISTRATION & MANAGEMENT, Tuberculosis < INFECTIOUS DISEASES, EPIDEMIOLOGY, HEALTH ECONOMICS, INFECTIOUS DISEASES, Paediatric infectious disease & immunisation < PAEDIATRICS

SCHOLARONE™
Manuscripts

I, the Submitting Author has the right to grant and does grant on behalf of all authors of the Work (as defined in the below author licence), an exclusive licence and/or a non-exclusive licence for contributions from authors who are: i) UK Crown employees; ii) where BMJ has agreed a CC-BY licence shall apply, and/or iii) in accordance with the terms applicable for US Federal Government officers or employees acting as part of their official duties; on a worldwide, perpetual, irrevocable, royalty-free basis to BMJ Publishing Group Ltd ("BMJ") its licensees and where the relevant Journal is co-owned by BMJ to the co-owners of the Journal, to publish the Work in this journal and any other BMJ products and to exploit all rights, as set out in our licence.

The Submitting Author accepts and understands that any supply made under these terms is made by BMJ to the Submitting Author unless you are acting as an employee on behalf of your employer or a postgraduate student of an affiliated institution which is paying any applicable article publishing charge ("APC") for Open Access articles. Where the Submitting Author wishes to make the Work available on an Open Access basis (and intends to pay the relevant APC), the terms of reuse of such Open Access shall be governed by a Creative Commons licence – details of these licences and which Creative Commons licence will apply to this Work are set out in our licence referred to above.

Other than as permitted in any relevant BMJ Author's Self Archiving Policies, I confirm this Work has not been accepted for publication elsewhere, is not being considered for publication elsewhere and does not duplicate material already published. I confirm all authors consent to publication of this Work and authorise the granting of this licence.

For peer review only

Xpert on stool to diagnose tuberculosis in children is cost-effective in Ethiopia and Indonesia: a model-based cost-effectiveness analysis

Authors

Nyashadzaishe Mafirakureva: n.mafirakureva@sheffield.ac.uk[1]

Eveline Klinkenberg: eveline@connecttb.org[2,3]

Ineke Spruijt: ineke.spruijt@kncvtbc.org[4]

Jens Levy: jens.levy@kncvtbc.org[4]

Debebe S Adewo: d.shaweno@sheffield.ac.uk[1]

Petra de Haas: Petra.deHaas@kncvtbc.org[4]

Nastiti Kaswandani: nkaswandani@ikafkui.net[5]

Ahmed Bedru: ahmed.bedru@kncvtbc.org[6]

Rina Triasih: rina_triasih@yahoo.com[7]

Melaku Gebremichel: melaku.gebremichel@kncvtbc.org[6]

Peter J Dodd: p.j.dodd@sheffield.ac.uk[1]

Everdina W Tiemersma: edine.tiemersma@kncvtbc.org [4]

Affiliations

[1] School of Health and Related Research, University of Sheffield, Sheffield, UK

[2] Independent consultant, Connect TB, The Hague, The Netherlands

[3] Department of Global Health and Amsterdam Institute for Global Health and Development, Amsterdam University Medical Centers, Amsterdam, The Netherlands.

[4] KNCV Tuberculosis Foundation, Maanweg 174, The Hague, The Netherlands

[5] Paediatric Department, RSCM Hospital, Faculty of Medicine, University of Indonesia, Jakarta, Indonesia

[6] KNCV Tuberculosis Foundation, Addis Ababa, Ethiopia

[7] Department of Paediatrics, Faculty of Medicine, Universitas Gadjah Mada/Dr. Sardjito Hospital, Yogyakarta, Indonesia.

Corresponding author: Nyashadzaishe Mafirakureva: n.mafirakureva@sheffield.ac.uk

Word count: ~ 450+1321+628+1172 = 3571

For peer review only - http://bmjopen.bmj.com/site/about/guidelines.xhtml

Keywords: cost-effectiveness analysis, systematic review

Abstract

Objectives

The World Health Organization currently recommend stool on GeneXpert MTB/Rif (Xpert) for the diagnosis of paediatric tuberculosis (TB). The simple one-step (SOS) stool method enables processing for Xpert testing at the primary healthcare (PHC) level. We modelled the impact and cost-effectiveness of implementing the SOS stool method at PHC for diagnosis of paediatric TB in Ethiopia and Indonesia, compared to standard of care.

Setting

All children (age <15 years) presenting with presumptive TB presenting at primary healthcare or hospital level in Ethiopia and Indonesia.

Primary outcome

Cost-effectiveness estimated as incremental costs compared with incremental disability-adjusted life-years saved.

Methods

Decision tree modelling was used to represent pathways of patient care and referral. We based model parameters on ongoing studies and surveillance, systematic literature review, and expert opinion. We estimated costs using data available publicly and in-country experts. Health outcomes were based on modelled mortality and discounted life-years lost.

Results

The intervention increased the sensitivity of TB diagnosis by 19-25% in both countries leading to a 14-20% relative reduction in mortality. Under the intervention, less than half of children seeking care at PHC were referred (or self-referred) to higher levels of care; the number of children initiating anti-TB treatment (ATT) increased by 18-25%; and more children (85%) initiated ATT at PHC level. Costs increased under the intervention compared to a base-case using smear microscopy in the SOC resulting in incremental cost-effectiveness ratios of $132 and $94 per DALY averted in Ethiopia and Indonesia, respectively. At a cost-effectiveness threshold of 0.5xGDP, the projected probability of the intervention being cost-effective in Ethiopia and Indonesia was 87% and 96%, respectively. The intervention remained cost-effective under sensitivity analyses.

Conclusions

The addition of the SOS stool method to the national algorithms for diagnosing TB in children is likely to be cost-effective in both Ethiopia and Indonesia.

For peer review only

Strengths and limitations of this study

- We report the first impact and cost-effectiveness of adding the SOS method to the current national algorithms for diagnosing TB in children (including at lower health care level).
- The SOS method is projected to increase the sensitivity of TB diagnosis leading to a reduction in mortality and life-years lost among children with presumptive TB.
- The projected reduction in referral rates from PHC to higher levels of care and has potential to reduce patient costs (out of pocket expenditures).
- The increased healthcare costs from the addition of the SOS stool method to the national algorithms for diagnosing TB in children were projected to be offset by the health benefits (and potential cost savings) resulting in the intervention being cost-effective.
- Although this work focuses on Ethiopia and Indonesia, the results from these two very diverse settings are likely to have global relevance to countries with high TB burden.

For peer review only - http://bmjopen.bmj.com/site/about/guidelines.xhtml

Background

It is estimated that in 2018, around 1.1 million children below 15 years of age fell ill from tuberculosis (TB).[1] In the same year, 250,000 children died of TB, mostly because TB was not diagnosed or was diagnosed too late. It is estimated that 55% of TB cases are missed, particularly in the youngest age group.[2] TB in children presents with nonspecific signs and symptoms, and *M. tuberculosis* bacilli are usually not detected.[2] Partly, this is because the main specimen used for diagnosing pulmonary TB is sputum, which is challenging to obtain, especially from (young) children. Therefore, (semi-)invasive methods such as nasogastric aspiration and sputum induction are often required. These methods are painful and stressful for children and care givers and sometimes require hospitalization. Moreover, not all primary health care (PHC) facilities in TB endemic areas, where parents with children usually first seek care, have facilities to perform these procedures. Alternative, non-invasive specimens, such as stool, can be used for the diagnosis of TB in children using Xpert MTB/RIF (Xpert) technology.[3, 4]

Since January 2020, WHO recommends stool on Xpert as a primary diagnostic test for TB in children with signs and symptoms of pulmonary TB.[5] This recommendation has the potential to improve bacteriological confirmation of TB in children, and is increasingly being adopted by national TB programs, for example, Ethiopia[6]. However, to make the test fit for use at the PHC level, a simple, non-hazardous, and cheap method to process stool for Xpert testing was needed. Several centrifuge-free methods have been proposed,[7-10] but all need additional equipment and/or consumables which may not be (easily) available in peripheral lower-level public health laboratories. Therefore, we developed a simple one-step (SOS) stool processing method for Xpert testing. This method can be applied in any laboratory with an Xpert machine, as it does not require additional equipment or consumables than those delivered routinely with the Xpert cartridges.[11] Hence, this method has the potential to significantly impact the number of children receiving a bacteriological confirmation of TB, including rifampicin resistance profile. Consequently, more paediatric TB patients can be diagnosed at lower levels of the healthcare system, with a reduced time to diagnosis because no referrals are needed to higher levels of healthcare as well as reduced costs for both the health system and families.

However, evidence on the impact and cost effectiveness of the SOS Xpert stool processing method is needed to inform implementation and scale-up in the routine health care system. Therefore, we modelled the potential impact and cost-effectiveness of bringing this test to the lower healthcare level where children present first, focusing on Ethiopia and Indonesia. Specifically, we aimed to estimate the impact of implementing the Xpert stool test for the diagnosis of pulmonary TB among children at the PHC level on rates of bacteriological confirmation of TB and mortality among children, the costs to the healthcare system, and the incremental cost-effectiveness of the approach.

For peer review only - http://bmjopen.bmj.com/site/about/guidelines.xhtml

Methods

Conceptual approach

We developed a conceptual model of care pathways for children (age <15 years) with presumptive TB presenting at either PHC facilities or hospitals, referral (including self-referral) between these levels, and clinical and bacteriological assessment and re-assessment (see Figure 1). This description of stages in patient care was based on national guidelines and local knowledge, and were broad enough to capture pathways in both Ethiopia and Indonesia, and incorporate the standard of care (SOC) as well as the intervention.

We defined presumptive TB patients, following guidelines in both settings, as children with signs or symptoms suggestive of pulmonary TB (at least one) such as persistent cough, unexplained fever and/or night sweats, poor weight gain or weight loss, reduced playfulness or malaise, history of contact with a TB patient, or enlarged lymph nodes in the neck (only Ethiopia).

Under the SOC, national guidelines in both countries recommend the usage of GeneXpert[6, 12]. However, in both countries, sputum smear microscopy is allowed for diagnosis if the health center has no access to GeneXpert. Despite this recommendation, in both countries, most PHC units do not have access to a GeneXpert machine, and therefore use sputum smear microscopy for the diagnosis of paediatric TB. For diagnosis of paediatric TB, in the primary (base-case) analyses, we assumed that sputum-smear microscopy was the bacteriological test used at PHC in SOC in both Ethiopia and Indonesia. As sensitivity analyses, we considered alternate scenarios with Xpert used for bacteriological testing for sputum in SOC.

The intervention was modelled as implementing the simple stool Xpert testing method at PHC and hospital level. Thus, considering spontaneous sputum expectoration to be limiting in obtaining a test result under SOC, we conceptualized the intervention as increasing the fraction of children with a bacteriological test result at both the primary and higher healthcare level.

We assumed that children with a negative bacteriological test under the intervention would receive clinical assessments for TB. A clinical diagnosis can be made based on TB-suggestive signs or symptoms, X-ray results, and tuberculin skin test (Indonesia only) or contact history with a TB patient. Indonesian referral centres that do not have access to X-ray and/or tuberculosis TB skin tests use a score chart for clinical diagnosis.

Systematic review data on the sensitivity of stool-based diagnostics for identifying TB in children, indicate sensitivity of 50-60% in children with bacteriologically confirmed TB, but very poor sensitivity (2-6%) in clinically diagnosed TB.[3, 4] We therefore assumed that stool testing would only detect a proportion of those children who would be bacteriologically positive under ideal circumstances. The intervention was expected to reduce mortality through higher sensitivity for detecting TB, and also to reduce referrals and re-assessments.

For peer review only - http://bmjopen.bmj.com/site/about/guidelines.xhtml

Modelling approach

The pathway of care shown in Figure 1 was coded into a decision tree using the HEdtree package in R.[13, 14] Referral endpoints from PHC level were modelled by adding an identical hospital care pathway to follow the three paths for referral from PHC level. All care outcomes were extended to either death or survival. The probability of children following different pathways through the tree was assumed to depend on: true TB status and age (0-4 years or 5-14 years). Mortality risk from TB by age group and anti-TB treatment (ATT) status was modelled using a published approach,[15] using case-fatality ratios (CFRs) based on systematic review data.[16] We neglected mortality in children who were truly negative for TB. We did not model drug-resistant TB or HIV status.

All parameters in the model were treated as uncertain and following specified distributions. All results were based on applying the model to calculate mean outcomes from the tree for each of 10,000 samples from these parameter distributions.

Literature review and parameterization

To inform the parameters needed in the decision tree model (Figure 1, parameters noted as such in Table 1), we followed a three-step data collection process. Firstly, we reviewed data from ongoing studies in Ethiopia[11] and Indonesia (Kaswandani N, Tiemersma EW, Janiar H, et al. Xpert MTB/RIF testing on stools using simple pre-processing methods to diagnose childhood pulmonary tuberculosis in Indonesia. 2019). Secondly, we systematically searched peer-reviewed literature for parameters not available from country study data. In brief, initially, systematic reviews on TB in children were sought by a search of PubMed including the terms “systematic review”, “meta-analysis”, “tuberculosis” and “children” on 19 June 2020. Subsequently, we constructed pooled estimates from primary literature, published from 2010 – present, about TB diagnostic testing in infants and children, including health care seeking and health care cascade with a focus on Ethiopia and Indonesia. For this, a systematic search strategy was developed by an information specialist combining free-text and thesaurus searching. Except for searches specifically addressing Indonesia/Ethiopia, we excluded case reports, non-English and non-human studies, and papers with terms for BCG, latent tuberculosis, IGRA and tuberculin skin test in titles because of their relevance to TB infection, not active pulmonary TB. Searches were conducted between 19 and 26 October 2020. Finally, to inform remaining parameters, we sought opinion from TB experts from each country (authors AB and MG for Ethiopia and NK and RT for Indonesia) in an iterative process using a questionnaire, and remote workshops to explain the model and focus on parameters identified as influential by one-way sensitivity analysis. More details are provided in Appendix 1 (literature search) and Appendix 2a (Model parameter estimation).

Cost parameters and health economic approach

We collected costs (reported in 2019 USD) from the healthcare provider’s perspective and adjusted historical costs for inflation to 2019 prices using relevant GDP deflators.[17] We transferred costs from

For peer review only - http://bmjopen.bmj.com/site/about/guidelines.xhtml

other countries to Ethiopia and Indonesia by applying relevant purchasing power parity conversion factors.[18] Costs were assumed to accrue in the present, with no discounting applied.

We assumed the cost for the initial TB assessment at the PHC was equivalent to the country-specific cost of two outpatient visits (or a single outpatient visit for re-assessment) to a health centre (health centre with no beds from WHO-CHOICE estimates[19]). Similar assumptions were used for hospital assessment and re-assessment with the corresponding WHO-CHOICE cost estimates.[19] The cost of bacteriological investigation in the SOC includes the country specific unit cost of either sputum smear microscopy (SSM)[20, 21] or Xpert, depending on availability at each level of care, adding the unit costs for collecting two sputum samples for testing with SSM or one sample for testing with Xpert. The unit costs for Xpert were estimated based on country specific data available from the OneHealth Tool (see Appendix).[22] Country-specific unit costs for collecting sputum samples were not available and are based on a study done in adults in South Africa.[23] In the intervention, we applied the unit cost for collecting a single stool sample based on estimates provided by the Paediatric Operational Sustainability Expertise Exchange group.[24] Treatment cost for diagnosed TB comprises the cost of anti-tuberculosis drugs (including pyridoxine), from the Global Drug Facility,[25] the costs of follow-up visits (drug pickups or medical review) according to national TB treatment guidelines at the healthcare facilities based on WHO-CHOICE unit cost estimates, and the costs of laboratory monitoring in bacteriologically confirmed TB only (see Appendix 2b (Overview of cost parameters)).

We used a disability-adjusted life-year (DALY) framework, calculating the life-years saved with a discount rate of 3% based on United Nations Population Division country-specific life tables. A simple mean across ages included in the 0-4 and 5-14 year age groups was used, and decrements in health-related quality of life or subsequent survival were not modelled.

Metrics calculated

For every 100 children seeking care with presumptive TB in each country, we calculated the deaths, DALYs, costs, referrals, clinical assessments, bacteriological assessments, ATTs, percent of true TB receiving ATT, percent of those receiving ATT bacteriologically confirmed, percent of those receiving ATT initiated at PHC, percent of ATT that is false-positive, as well as the change in these quantities under the intervention. We report the incremental cost-effectiveness ratio (ICER). For each country, we produced plots of the cost-effectiveness plane, cost-effectiveness acceptability curve, and expected net benefit, and tornado plots illustrating the one-way sensitivity of outcomes to influential model parameters. We also undertook specific scenario analyses: 1) we considered a low TB prevalence scenario (half the base-case prevalence among presumptive TB patients); 2) we considered Xpert as the universally available bacteriological test instead of sputum-smear microscopy in SOC; 3) we considered discount rates of 0% and 5% for the life years. The results of these sensitivity analyses are included in Appendix 3 (Additional results).

Patient and Public Involvement

Study participants or the public were not involved in the design, or conduct, or reporting, or dissemination plans of our research.

For peer review only - http://bmjopen.bmj.com/site/about/guidelines.xhtml

Research ethics statement

Not applicable. This was a modelling study based on secondary data.

Results

Following our review of the literature, we developed the model parametrization shown in Table 1. The data sources and approach to synthesis for each parameter are described in detail respectively in Appendix 1 and Appendix 2a. Briefly, we used existing systematic reviews for the basis of parameters describing diagnostic test accuracy, our own pooled estimates of true TB prevalence among presumptive TB, the fraction of TB that is bacteriological confirmable, and the fraction of children able to spontaneously expectorate. Evidence for the accuracy of clinical diagnosis and the level of initial care seeking was limited, and published evidence was completely lacking for other parameters around referral and re-assessment. Hence, we based these on expert opinion. Unit costs used in the analysis are shown in Table 2.

The intervention increased the sensitivity to detect true TB by over 10 percentage points in each country and resulted in around a 4-fold increase in the proportion of TB patients diagnosed that are bacteriologically confirmed. Specificity showed little change under the intervention (<1% change). In both countries, the proportion of children referred (or self-referred) to higher levels of care after seeking care at PHC level fell by more than 2-fold. In both countries, the average total number of assessments for children with presumptive TB increased from around 2 under SOC to around 2.5 per child with the intervention, and the total number of bacteriological investigations increased more than 3-fold (Table 3).

The relative number of children initiated on ATT increased by 19-25% under the intervention. A larger fraction (~40% relative increase) of children received ATT with the intervention, and more children (~10% point increase) initiated ATT at PHC level (Table 3). Restricting to children under 5, we found bigger increases in the number of bacteriological investigations (+30-fold), and the proportion of TB cases diagnosed that are bacteriologically confirmed (+50%). We also found a larger reduction in referrals of children with presumptive TB to higher levels of care in both countries (almost 3-fold). (see Appendix 3 (Additional results), Tables A1 and A2).

In both countries, the increase in sensitivity of a TB diagnosis under the intervention generated a corresponding reduction in mortality: a 14%-20% relative reduction in the fraction of children with presumptive TB dying (Table 3). In both countries, costs increased under the intervention, and the base-case (using smear microscopy in the SOC) ICERs were $132/DALY averted in Ethiopia and $94/DALY averted in Indonesia (Figure 2). Restricting the analysis to children under 5 resulted in cost-savings with ICERs of -$78/DALY averted in Ethiopia and increased the ICER to $209/DALY averted in Indonesia.

Uncertainty and sensitivity analyses

Model projections showed large uncertainty (Figure 2) that included cost savings under intervention (25% of the runs for Ethiopia and 28% for Indonesia), but also some increases in mortality (1.2% of the runs for Ethiopia and 2.8% for Indonesia). At a cost-effectiveness threshold of 0.5xGDP our analysis projected a 87% probability of being cost effective in Ethiopia and a 96% probability of being cost-effective in Indonesia (see Appendix 3). The corresponding probabilities for a 1xGDP threshold were 95% (Ethiopia)

For peer review only - http://bmjopen.bmj.com/site/about/guidelines.xhtml

and 97% (Indonesia). Tornado plots (Figure 3) show that prevalence of true TB among presumptive TB, the sensitivity of stool, and the fraction of children able to expectorate were the largest drivers of uncertainty (see also Appendix 3).

Under the assumption that Xpert was used in the SOC, the ICERs were $138/DALY averted in Ethiopia and $115/DALY averted in Indonesia. Assuming half the prevalence of true TB among presumptive TB patients changed the ICERs to $178/DALY averted in Ethiopia and $150/DALY averted in Indonesia. Finally, assuming a 0% discount rate changed the ICERs to $55/DALY averted in Ethiopia and $38/DALY averted in Indonesia, whereas 5% discount rate generated ICERs of $199/DALY averted in Ethiopia and $142/DALY averted in Indonesia.

Discussion

In this modelling analysis, we found that the introduction of routine Xpert stool-based diagnostics (using the SOS method) was cost-effective in both Ethiopia and Indonesia. In the context of predominantly clinical diagnosis of TB in children, particularly among those aged <5 years, we found a 14-20% relative reduction in mortality driven by an increase in sensitivity to detect true TB. However, it is crucial that clinical assessment is still undertaken alongside negative bacteriological test results because bacteriological testing has a low negative predictive value especially in young children.[3, 4] Relying on bacteriological testing alone can reduce sensitivity to diagnose true TB and increase mortality, especially if referrals and re-assessments are common under the standard of care (data not shown). We estimated ICERs of $132 and $94 per DALY averted in the base-case analyses for Ethiopia and Indonesia, respectively. These ICERs are less than 0.5 x GDP, which has been suggested as a rule of thumb for cost-effectiveness thresholds,[26] as well as country-specific estimates of supply-side thresholds.[27, 28] The intervention would be especially cost-effective for children under 5 years of age.

Children age <5 years are at higher risk of dying from untreated TB than older children and have the greatest difficulty in spontaneously expectorating sputum. Under the intervention, we found greater increases in bacteriologically diagnosis and greater decreases in referrals in the <5 year age group (see age stratified results in Appendix 3). We found that the cost of introducing the intervention was partially offset by reduced referrals from PHC facilities to hospitals. In Ethiopia, this produced a projected cost saving in the under 5 age group, despite a slight increase in the average total number of assessments done. In taking a health system perspective, we did not include patient costs in our analysis, but health-seeking costs are a major driver of catastrophic costs in TB.[29]

There are large uncertainties associated with many parameters describing processes and pathways for paediatric TB. We found no directly applicable estimates of rates of reassessments or (self)referral at different stages of care, and had to rely on expert opinion. Additionally, the sensitivity and specificity of clinical assessment for paediatric TB is poorly quantified in the literature. Because of this, we placed a particular emphasis on including uncertainty in results, as well as systematically exploring their sensitivity to one-way variation in parameters, and discrete alternative assumptions. For example, because our estimate of true TB prevalence among children with presumptive TB was based on data mainly from hospitals which may have higher prevalence than PHC level, we halved prevalence resulting in increased ICERs by less than a factor of two without changing our qualitative conclusions. Despite these

For peer review only - http://bmjopen.bmj.com/site/about/guidelines.xhtml

uncertainties, the intervention showed probabilities of being cost-effective > 85% in each country across a wide range of cost-effectiveness thresholds. This conclusion was also robust to assuming the SOC used Xpert rather than sputum-smear microscopy at PHC level, and to different choices of discount rate.

Some aspects were deliberately simplified or omitted in the modelling. First, we did not model HIV because paediatric HIV rates in Ethiopia and Indonesia are relatively low at 0.09% and 0.03%,[30] respectively. This may underestimate the benefit from the intervention due to underestimated TB mortality, especially if stool-based methods are more effective at diagnosing TB in children with HIV compared to sputum. Secondly, we did not model drug-resistant TB because of low rates of multidrug-resistant (MDR) TB among new TB cases (all ages) in Ethiopia (1.02% (0.49 - 1.54%)) and Indonesia (2.4% (1.8-3.3%)). This may underestimate the intervention costs since the higher fractions of cases bacteriologically confirmed via Xpert MTB/Rif mean that more MDR TB will be diagnosed and require more costly second-line treatment. Thirdly, we did not consider the private sector, which in Indonesia is substantial, and less likely to closely follow guidelines. Our intervention is conceived as being implemented in the public sector, but patients seeking care across both sectors may mean we overestimate the savings to the (public) health system from reduced referrals. Fourthly, additional one-off costs for widely introducing Xpert stool testing, such as costs for training and supervision, were not included in our analyses. Both countries are moving to fully replacing sputum smear microscopy by Xpert testing as the primary diagnostic for TB in all patient groups. This may increase logistical issues in both countries which need to be dealt with, such as cartridge shelf life (which is shorter for the Ultra than the G4 cartridge) and module maintenance. Lastly, we modelled the impact of making a stool Xpert-based diagnosis available at the PHC level. This assumes that all PHC facilities have access to a GeneXpert machine, either on-site or through an effective sample transportation system. Thus, until full access to Xpert testing is available, the coverage of the intervention will be limited.

Furthermore, due to the lack of data from randomized controlled trials and operational studies, we were reliant on early experience to determine acceptability and feasibility of stool-based sampling and diagnostics. Hence, difficulties in implementation that dilute effects or increase costs may be overlooked. However, the recent recommendation to use stool as a primary sample for diagnosing childhood TB[5] has generated interest in Xpert stool testing at national TB programs. Apart from the SOS stool method, two other centrifuge-free stool processing methods are being developed,[9, 10] which are included in a head-to-head comparison study to compare their performance in diagnosing childhood TB against sputum or gastric aspirate culture. This project has a health economic component, estimating cost-effectiveness of the best performing method. Results of this project are expected at the end of 2021. The TB Speed decentralization operational research study will report results from use of Xpert on nasopharyngeal aspirate and stool samples at PHC level in early 2022. A small study comparing the SOS stool method to the stool processing kit involved in the head-to-head comparison study[10] concluded that taking into account the sample processing time, consumable requirements and error rates, the SOS stool method would be the method that would be best scalable in low and middle income countries.[31]

Additional evidence from studies and implementation is needed to inform the optimal use of new sample and diagnostic approaches for paediatric TB within real health systems. Studies to quantify referral patterns, the pathways and outcomes of individual patients, and the costs of real-world implementation would be particularly valuable. Further analyses could include context-specific operational research to

For peer review only - http://bmjopen.bmj.com/site/about/guidelines.xhtml

help design referral systems that best utilize Xpert machines and minimize cartridge expiry, as well as budget impact analyses to help national programmes plan roll-out and seek funding. Clinical diagnosis remains an important tool for children with TB; helping clinicians diagnose TB in children without bacteriological results or with negative results should be part of intervention design and the role of clinical diagnosis in current and novel diagnostic pathways a topic for further research.

Conclusion

In this modelling analysis, we projected that introduction of routine stool-based Xpert diagnostics at primary health care and hospital level would increase the proportion of bacteriologically confirmed TB in children, while reducing child mortality and life-years lost in both Ethiopia and Indonesia. Our analysis suggests that this intervention would be cost-effective in both countries.

For peer review only

Acknowledgements

We thank Anthea Sutton, an Information Specialist at the University of Sheffield, for setting up and conducting the literature database searches; Sarah van de Berg for her assistance in assessing and extracting data from the systematic reviews; Key informants for the expert-solicited parameters from Ethiopia and Indonesia; the NTP and related stakeholders, including pediatricians, scientists, technical agencies and funders implementing childhood TB projects in both countries.

Author contributions

Conceptualization: NM, EK, IS, JL, DS, PdH, PD, ET

Systematic reviewing: EK, IS, JL, ET

Modelling and quantitative analyses: NM, DS, PD

First draft: NM, EK, IS, JL, DS, PD, ET

Review of country epidemiological and health system approach: NK, AB, RT, MG

Review of approach, editing article and approval of final manuscript: all authors.

Conflicts of interest

The authors declare no conflicts of interest.

Funding / Support statement

This work was funded by the TB Modelling and Analysis Consortium (Bill and Melinda Gates Foundation, OPP1084276). PJD was supported by a fellowship from the UK Medical Research Council (MR/P022081/1); this UK funded award is part of the EDCTP2 programme supported by the European Union.

Data availability

Data and code to run these are analyses are available on reasonable request.

For peer review only

References

1. World Health Organization. Global tuberculosis report 2019: World Health Organization; 2019 [Available from: https://www.who.int/teams/global-tuberculosis-programme/tb-reports/global-report-2019.
2. World Health Organization. Roadmap towards ending TB in children and adolescents 2018 [Available from: https://apps.who.int/iris/bitstream/handle/10665/274374/9789241514668-eng.pdf.
3. MacLean E, Sulis G, Denkinger CM, et al. Diagnostic Accuracy of Stool Xpert MTB/RIF for Detection of Pulmonary Tuberculosis in Children: a Systematic Review and Meta-analysis. *J Clin Microbiol*. 2019;57(6):e02057-18.
4. Mesman AW, Rodriguez C, Ager E, et al. Diagnostic accuracy of molecular detection of Mycobacterium tuberculosis in pediatric stool samples: A systematic review and meta-analysis. *Tuberculosis (Edinb)*. 2019;119:101878-101878.
5. World Health Organization. Rapid Communication: Molecular assays as initial tests for the diagnosis of tuberculosis and rifampicin resistance 2020 [Available from: https://www.who.int/publications/i/item/9789240000339.
6. Ministry of Health Ethiopia. Guidelines for Clinical and Programmatic Management of TB, DR-TB and Leprosy in Ethiopia, 7th edition. Addis Ababa, Ethiopia: Ministry of Health Ethiopia; 2020.
7. Andriyoko B, Janiar H, Kusumadewi R, et al. Simple stool processing method for the diagnosis of pulmonary tuberculosis using GeneXpert MTB/RIF. *Eur Respir J*. 2018;53(3):1801832.
8. Banada PP, Naidoo U, Deshpande S, et al. A Novel Sample Processing Method for Rapid Detection of Tuberculosis in the Stool of Pediatric Patients Using the Xpert MTB/RIF Assay. *PLoS One*. 2016;11(3):e0151980-e0151980.
9. Lounnas M, Diack A, Nicol MP, et al. Laboratory development of a simple stool sample processing method diagnosis of pediatric tuberculosis using Xpert Ultra. *Tuberculosis (Edinb)*. 2020;125:102002.
10. Walters E, Scott L, Nabeta P, et al. Molecular Detection of Mycobacterium tuberculosis from Stools in Young Children by Use of a Novel Centrifugation-Free Processing Method. *J Clin Microbiol*. 2018;56(9):e00781-18.
11. de Haas P, Yenew B, Mengesha E, et al. The Simple One-Step (SOS) Stool Processing Method for Use with the Xpert MTB/RIF Assay for a Child-Friendly Diagnosis of Tuberculosis Closer to the Point of Care. *J Clin Microbiol*. 2021;59(8).
12. Ministry of Health Republic of Indonesia. National guideline for TB control. Jakarta, Indonesia: Ministry of Health Republic of Indonesia; 2016.
13. Dodd PJ. HEdtree: A Package For Decision Tree Modelling 2021 [Available from: https://github.com/petedodd/HEdtree.
14. R Core Team. R: A language and environment for statistical computing. Vienna, Austria: R Foundation for Statistical Computing; 2020.
15. Dodd PJ, Yuen CM, Sismanidis C, et al. The global burden of tuberculosis mortality in children: a mathematical modelling study. *Lancet Glob Health*. 2017;5(9):e898-e906.
16. Jenkins HE, Yuen CM, Rodriguez CA, et al. Mortality in children diagnosed with tuberculosis: a systematic review and meta-analysis. *Lancet Infect Dis*. 2017;17(3):285-295.
17. World Bank. World Bank national accounts data, and OECD National Accounts data files. GDP deflator: The World Bank; 2021 [Available from: https://data.worldbank.org/indicator/NY.GDP.DEFL.ZS.
18. World Bank. PPP conversion factor, GDP (LCU per international $): International Comparison Program, World Bank; 2021 [Available from: https://data.worldbank.org/indicator/PA.NUS.PPP.

For peer review only - http://bmjopen.bmj.com/site/about/guidelines.xhtml

19. Stenberg K, Lauer JA, Gkountouras G, et al. Econometric estimation of WHO-CHOICE country-specific costs for inpatient and outpatient health service delivery. *Cost Eff Resour Alloc*. 2018;16:11-11.
20. Jarrah Z CD, Hafidz F. The cost of scaling up TB services in Indonesia.: TB CARE I – Management Sciences for Health.; 2013.
21. Tesfaye A, Fiseha D, Assefa D, et al. Modeling the patient and health system impacts of alternative xpert® MTB/RIF algorithms for the diagnosis of pulmonary tuberculosis in Addis Ababa, Ethiopia. *BMC Infect Dis*. 2017;17(1):318-318.
22. World Health Organization. OneHealth Tool Geneva, Switzerland: World Health Organization; 2020 [Available from: https://www.who.int/choice/onehealthtool/en/.
23. Peter JG, Theron G, Pooran A, et al. Comparison of two methods for acquisition of sputum samples for diagnosis of suspected tuberculosis in smear-negative or sputum-scarce people: a randomised controlled trial. *Lancet Respir Med*. 2013;1(6):471-478.
24. Paediatric Operational Sustainability Expertise Exchange group. Paediatric Operational Sustainability Expertise Exchange group (POSEE group) budgeting tools 2020 [Available from: https://www.dropbox.com/sh/rmzlqoot9m1muxe/AACOPh8SuS4WwMQD_vfN-oHCa?dl=0.
25. Stop TB Partnership | Global Drug Facility. Global Drug Facility (GDF) Medicines catalogue January 2021 2021 [Available from: http://www.stoptb.org/assets/documents/gdf/drugsupply/GDFMedicinesCatalog.pdf.
26. Chi YL, Blecher M, Chalkidou K, et al. What next after GDP-based cost-effectiveness thresholds? *Gates Open Res*. 2020;4:176-176.
27. Woods B, Revill P, Sculpher M, et al. Country-Level Cost-Effectiveness Thresholds: Initial Estimates and the Need for Further Research. *Value Health*. 2016;19(8):929-935.
28. Ochalek J, Lomas J, Claxton K. Estimating health opportunity costs in low-income and middle-income countries: a novel approach and evidence from cross-country data. *BMJ Glob Health*. 2018;3(6):e000964-e000964.
29. Tanimura T, Jaramillo E, Weil D, et al. Financial burden for tuberculosis patients in low- and middle-income countries: a systematic review. *Eur Respir J*. 2014;43(6):1763-1775.
30. Joint United Nations Programme on HIV/AIDS. HIV estimates with uncertainty bounds 1990-Present | UNAIDS. Geneva: UNAIDS2021 [Available from: http://aidsinfo.unaids.org.
31. Jasumback CL, Dlamini Q, Kahari J, et al. Laboratory comparison of stool processing methods for Xpert(®) Ultra. *Public Health Action*. 2021;11(2):55-57.
32. Detjen AK, DiNardo AR, Leyden J, et al. Xpert MTB/RIF assay for the diagnosis of pulmonary tuberculosis in children: a systematic review and meta-analysis. *Lancet Respir Med*. 2015;3(6):451-61.
33. Marais BJ, Gie RP, Hesseling AC, et al. A refined symptom-based approach to diagnose pulmonary tuberculosis in children. *Pediatrics*. 2006;118(5):e1350-9.
34. Fekadu L, Hanson C, Osberg M, et al. Increasing Access to Tuberculosis Services in Ethiopia: Findings From a Patient-Pathway Analysis. *J Infect Dis*. 2017;216(suppl_7):S696-s701.
35. Surya A, Setyaningsih B, Suryani Nasution H, et al. Quality Tuberculosis Care in Indonesia: Using Patient Pathway Analysis to Optimize Public–Private Collaboration. *J Infect Dis*. 2017;216(suppl_7):S724-S732.

For peer review only - http://bmjopen.bmj.com/site/about/guidelines.xhtml

Figures legends

Figure 1. Simplified diagram of pathways of care. Grey lines indicate referrals up. See Appendix 2a for more details on pathway and parametrization

MTB: mycobacterium tuberculosis; TB: tuberculosis; TB Tx: TB diagnosis and anti-TB treatment; PHC = primary health care

Figure 2. Cost-effectiveness plane (Ethiopia left, Indonesia right)

ICER: incremental cost effectiveness ratio; SoC: Standard of Care

Figure 3. Tornado plots showing one-way sensitivity of incremental deaths (top row) and incremental costs (bottom row) to parameters for Ethiopia (left) and Indonesia (right). *spont.sputo5: spontaneous sputum possible (0-4 years), r1: referral from PHC to Hospital after clinical re-assessment following bacteriological negative result, r2: referral from PHC to Hospital after initial clinical assessment without bacteriological test result, fracu5: fraction of presumptive TB under 5, c_f.phc: cost of TB treatment at PHC after clinical re-assessment, c_d.phc: cost of TB treatment at PHC after initial clinical assessment, c_a.phc: cost of clinical and bacteriological TB assessment at PHC, c_clin.h: cost of clinical TB assessment at Hospital, c_clin.phc: cost of clinical TB assessment at PHC (Only tope 3 parameters on each plot defined here. Please refer to Appendix 2a, Tables A7-12 and Appendix 2b, Table for the rest of the parameter definitions).*

For peer review only - http://bmjopen.bmj.com/site/about/guidelines.xhtml

For peer review only

Tables

Table 1 Table of parameters used in modelling and underlying evidence. More details on parameter distributions, parameter naming, and methods are available in Appendix 2a. C+: culture positive, CFR: case fatality rate, PHC: primary health care, PTLTFU: pre-treatment lost to follow-up, SM: smear microscopy.

DESCRIPTION	SOURCE	REFERENCES	Mean (IQR)
sensitivity of Xpert on stool in bacteriologically positive children	existing review	Mesman 2019[4]	0.571 (0.515 - 0.627)
specificity of Xpert on stool in bacteriologically positive children	existing review	Mesman 2019[4]	0.981 (0.975 - 0.986)
sensitivity of Xpert on sputum in C+	existing review	Detjen 2015[32]	0.621 (0.582 - 0.659)
specificity of Xpert on sputum in C+	existing review	Detjen 2015[32]	0.980 (0.977 - 0.984)
sensitivity of SM on sputum in C+	existing review	Detjen 2015[32]	0.257 (0.215 - 0.302)
specificity of SM on sputum in C+	existing review	Detjen 2015[32]	0.995 (0.994 - 0.997)
spontaneous sputum possible (0-4 years)	our review	see Appendix 2a	0.024 (0.020 - 0.027)
spontaneous sputum possible (5-14 years)	our review	see Appendix 2a	0.377 (0.254 - 0.512)
fraction of children bacteriologically confirmable <5 years	our review	see Appendix 2a	0.380 (0.363 - 0.397)
fraction of children bacteriologically confirmable 5-14 years	our review	see Appendix 2a	0.684 (0.659 - 0.711)
prevalence of true TB in presumptive	our review	see Appendix 2a	0.453 (0.289 - 0.607)
specificity of clinical diagnosis < 5 years	our review	Marais 2006 (see Appendix 2a)	0.928 (0.908 - 0.945)
sensitivity of clinical diagnosis < 5 years	our review	Marais 2006[33]	0.518 (0.482 - 0.554)
specificity of clinical diagnosis 5-14 years	our review	Marais 2006[33]	0.901 (0.878 - 0.921)
sensitivity of clinical diagnosis 5-14 years	our review	Marais 2006[33]	0.627 (0.592 - 0.661)

For peer review only - http://bmjopen.bmj.com/site/about/guidelines.xhtml

proportion of first care-seeking at PHC for **Ethiopia**	our review	Fekadu et al, 2017[34]	0.896 (0.777 - 0.973)
proportion of first care-seeking at PHC for **Indonesia**	our review	Surya et al, 2017[35]	0.928 (0.801 - 0.992)
fraction of presumptive TB under 5 years **Ethiopia**	routine data	fraction of WHO TB < 5	0.371 (0.300 - 0.447)
fraction of presumptive TB under 5 years **Indonesia**	routine data	fraction of WHO TB < 5	0.514 (0.485 - 0.543)
referral PHC -> Hospital after clinical re-assessment following bacteriological negative result **Ethiopia**	expert opinion	see Appendix 2a	0.045 (0.019 - 0.088)
referral PHC -> Hospital after clinical re-assessment following bacteriological negative result **Indonesia**	expert opinion	see Appendix 2a	0.200 (0.107 - 0.272)
referral PHC -> Hospital after initial clinical assessment without bacteriological test result **Ethiopia**	expert opinion	see Appendix 2a	0.800 (0.728 - 0.899)
referral PHC -> Hospital after initial clinical assessment without bacteriological test result **Indonesia**	expert opinion	see Appendix 2a	0.500(0.391 - 0.607)
clinical re-assessment, PHC **Ethiopia**	expert opinion	see Appendix 2a	0.045 (0.019 - 0.088)
clinical re-assessment, PHC **Indonesia**	expert opinion	see Appendix 2a	0.045 (0.019 - 0.088)
1-PTLTFU bacteriologically positive, PHC	assumption		0.953 (0.937 - 0.966)
1-PTLTFU bacteriologically positive, Hospital	assumption		0.953 (0.937 - 0.966)
clinical re-assessment after bacteriologically negative, PHC	assumption		0.045 (0.019 - 0.088)
clinical re-assessment after bacteriologically negative, Hospital	assumption		0.045 (0.019 - 0.088)
clinical re-assessment, Hospital	assumption		0.045 (0.019 - 0.088)
referral PHC -> Hospital after clinical re-assessment without bacteriological test result	assumption		0.500(0.391 - 0.607)
CFR children <5 years on TB treatment	existing review	Jenkins et al 2017[16]	0.019 (0.012 - 0.029)
CFR children 5-14 years on TB treatment	existing review	Jenkins et al 2017[16]	0.008 (0.006 - 0.011)
CFR children <5 years without TB treatment	existing review	Jenkins et al 2017[16]	0.436 (0.413 - 0.460)
CFR children 5-14 years without TB treatment	existing review	Jenkins et al 2017[16]	0.149 (0.137 - 0.162)

For peer review only - http://bmjopen.bmj.com/site/about/guidelines.xhtml

Table 2 Unit costs for different activities. See Appendix 2b for methods and naming conventions

Cost description	Unit cost, US$ (SD)	
	Ethiopia	Indonesia
TB assessment at health centre	10.22 (5.29)	43.35 (24.24)
TB reassessment at health centre	5.11 (2.25)	21.68 (10.52)
Self-expectorated sputum sample	2.32 (0.58)	1.74 (0.43)
Stool sample	1.67 (0.42)	1.67 (0.42)
Sputum smear microscopy examination	3.39 (1.44)	7.54 (1.58)
GeneXpert test	26.04 (7.09)	23.70 (7.11)
TB treatment at health centre	398.74 (177.22)	161.03 (78.59)
TB assessment at hospital	14.37 (6.59)	61.00 (30.23)
TB reassessment at health centre	5.11 (2.25)	21.68 (10.52)
TB treatment at hospital	548.46 (208.38)	213.98 (91.47)

For peer review only

Table 3 Outcomes per 100 children seeking care (*asterisk indicates different denominators) under standard of care (SOC) and intervention (INT) in each country. Quoted as Mean (95% quantiles). TB = tuberculosis; ATT = anti-TB treatment; PHC = primary health care; USD = United States Dollars

Quantity per 100 children with presumptive TB (unless stated):	Ethiopia			Indonesia		
	Standard of care	Intervention	Difference	Standard of care	Intervention	Difference
children with true TB	45.5 (8.7 - 85.0)	45.5 (8.7 - 85.0)	0.0 (0.0 - 0.0)	45.5 (8.7 - 85.0)	45.5 (8.7 - 85.0)	0.0 (0.0 - 0.0)
assessments	201.8 (171.8 - 230.9)	246.2 (207.3 - 283.3)	44.4 (29.5 - 58.1)	204.2 (173.4 - 233.5)	249.9 (211.2 - 286.5)	45.7 (31.9 - 58.0)
bacteriological investigations	30.7 (8.7 - 57.5)	102.3 (86.8 - 112.0)	71.7 (41.5 - 96.3)	24.7 (7.8 - 43.2)	103.0 (87.5 - 112.6)	78.2 (54.5 - 98.4)
anti-TB treatments (ATT)	32.2 (13.2 - 54.5)	40.3 (17.6 - 64.4)	8.1 (0.6 - 20.3)	33.3 (14.1 - 55.3)	39.5 (17.1 - 63.3)	6.2 (0.1 - 15.2)
*ATT initiated at PHC	71.8 (62.3 - 79.6)	81.9 (71.6 - 89.5)	10.1 (5.8 - 14.2)	73.0 (63.2 - 80.3)	84.4 (73.2 - 91.2)	11.3 (7.1 - 15.4)
*percent of true-positive receiving ATT	58.3 (43.0 - 71.1)	73.0 (66.7 - 78.8)	14.7 (2.8 - 30.5)	60.3 (48.2 - 71.4)	71.8 (65.9 - 77.3)	11.5 (1.8 - 23.1)
*percent of ATT bacteriologically confirmed	8.0 (1.7 - 19.8)	32.8 (20.7 - 44.1)	24.8 (10.6 - 37.8)	5.9 (1.4 - 12.9)	32.5 (20.9 - 43.4)	26.6 (14.9 - 38.2)
*percent of ATT false-positive	21.9 (2.8 - 64.6)	21.9 (2.9 - 64.9)	0.0 (-3.0 - 4.0)	22.0 (2.8 - 65.1)	21.8 (2.9 - 64.6)	-0.3 (-3.5 - 3.5)
referrals, inc. self-referrals	29.5 (17.0 - 42.9)	13.8 (8.0 - 21.0)	-15.6 (-25.8 - -4.9)	33.0 (21.5 - 45.5)	14.5 (8.6 - 21.7)	-18.4 (-27.6 - -9.6)
deaths	4.9 (0.9 - 10.0)	3.9 (0.7 - 8.3)	-1.0 (-2.8 - - 0.1)	5.4 (1.0 - 10.9)	4.7 (0.9 - 9.3)	-0.8 (-2.2 - 0.0)
life-years lost	135.7 (25.1 - 276.9)	108.7 (19.7 - 228.5)	-27.0 (-75.9 - -1.6)	154.8 (29.3 - 310.1)	133.1 (24.7 - 264.6)	-21.7 (-61.7 - 0.2)
cost	15729.4 (6368.3 - 31027.5)	19297.7 (8413.8 - 35444.7)	3568.3 (-8472.2 - 16311.6)	12508.1 (7056.4 - 20279.0)	14525.7 (8603.6 - 22403.0)	2017.6 (-5421.3 - 9470.6)

For peer review only - http://bmjopen.bmj.com/site/about/guidelines.xhtml

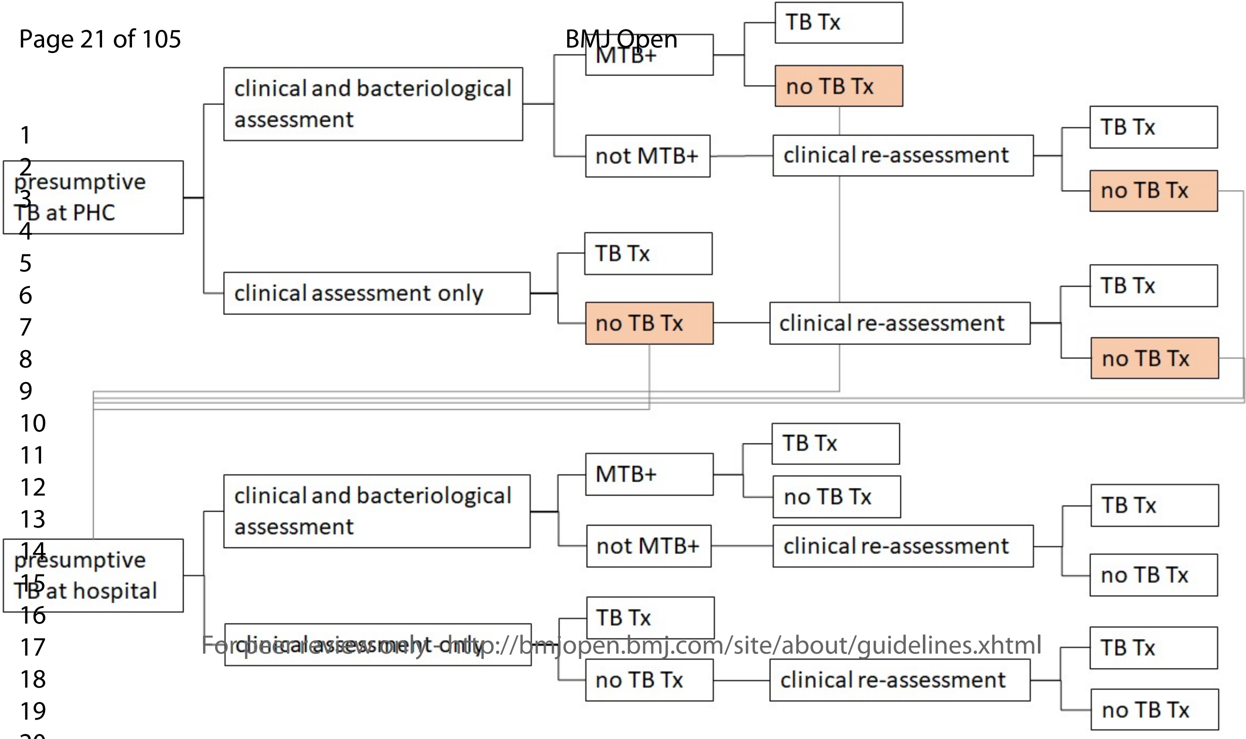

For peer review only - http://bmjopen.bmj.com/site/about/guidelines.xhtml

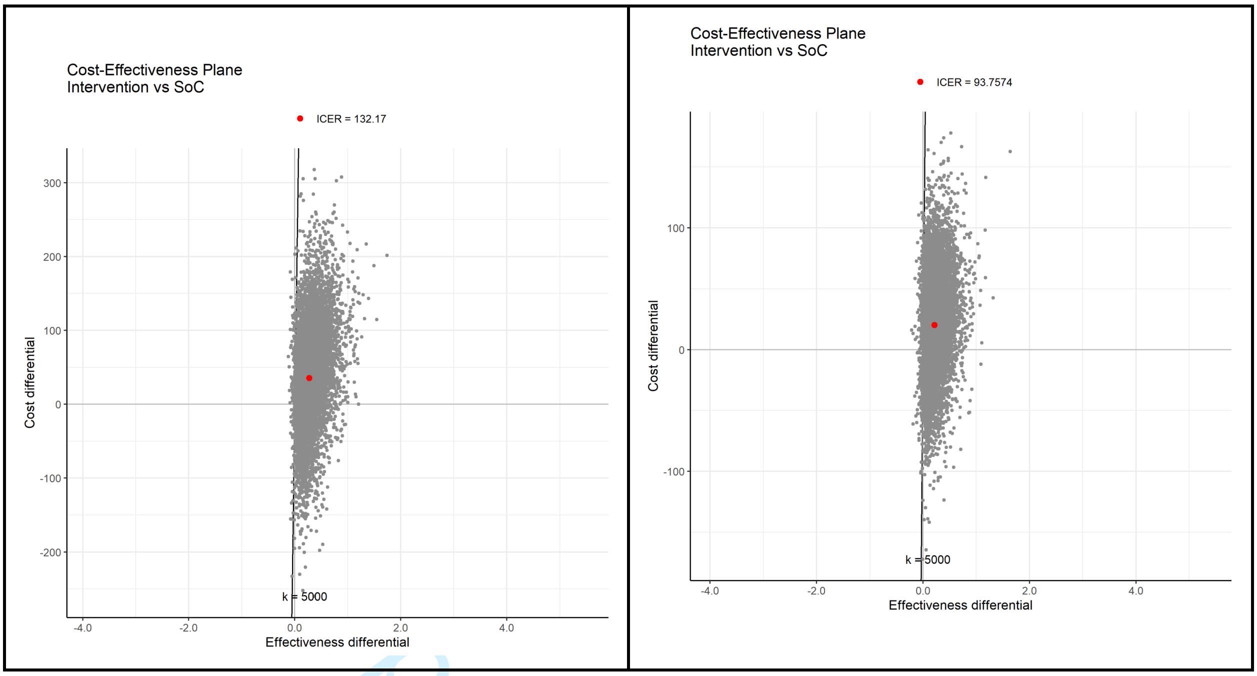

For peer review only

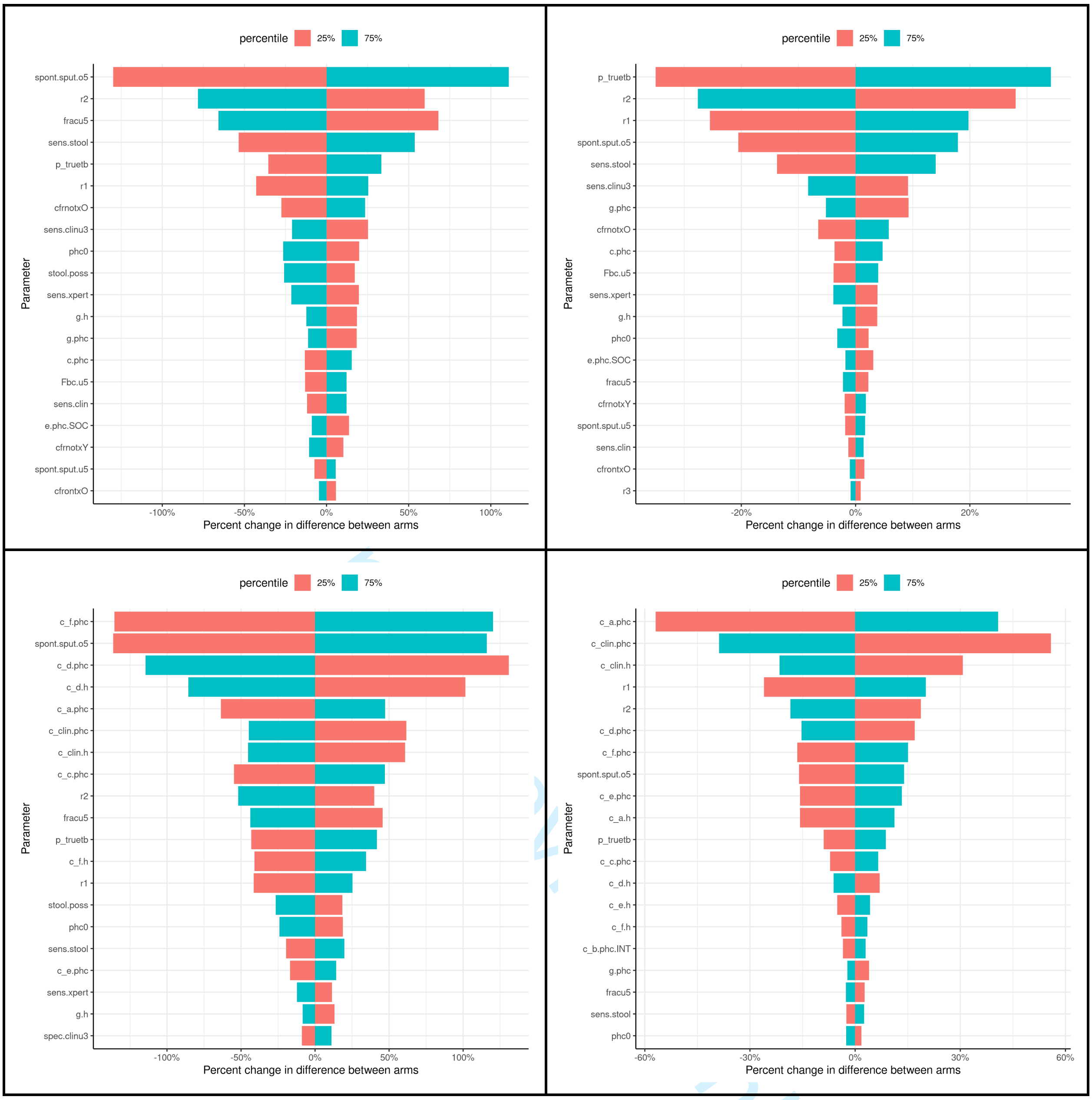
percentile 25% 75%
spont.sput.o5
r2
fracu5
sens.stool
p_truetb
r1
cfrnotxO
sens.clinu3
phc0
stool.poss
sens.xpert
g.h
g.phc
c.phc
Fbc.u5
sens.clin
e.phc.SOC
cfrnotxY
spont.sput.u5
cfrontxO
Parameter
-100% -50% 0% 50% 100%
Percent change in difference between arms
percentile 25% 75%
p_truetb
r2
r1
spont.sput.o5
sens.stool
sens.clinu3
g.phc
cfrnotxO
c.phc
Fbc.u5
sens.xpert
g.h
phc0
e.phc.SOC
fracu5
cfrnotxY
spont.sput.u5
sens.clin
cfrontxO
r3
Parameter
-20% 0% 20%
Percent change in difference between arms
percentile 25% 75%
c_f.phc
spont.sput.o5
c_d.phc
c_d.h
c_a.phc
c_clin.phc
c_clin.h
c_c.phc
r2
fracu5
p_truetb
c_f.h
r1
stool.poss
phc0
sens.stool
c_e.phc
sens.xpert
g.h
spec.clinu3
Parameter
-100% -50% 0% 50% 100%
Percent change in difference between arms
percentile 25% 75%
c_a.phc
c_clin.phc
c_clin.h
r1
r2
c_d.phc
c_f.phc
spont.sput.o5
c_e.phc
c_a.h
p_truetb
c_c.phc
c_d.h
c_e.h
c_f.h
c_b.phc.INT
g.phc
fracu5
sens.stool
phc0
Parameter
-60% -30% 0% 30% 60%
Percent change in difference between arms

Xpert on stool to diagnose tuberculosis in children is cost-effective in Ethiopia and Indonesia: a model-based cost-effectiveness analysis

APPENDIX 1: Literature search

To inform the model parameters presented in Appendix 2a, we extracted data from systematic reviews and from papers identified through an extensive targeted systematic literature search. This information was supplemented with information from papers identified from the authors' personal databases where relevant.

Data collection from published peer-reviewed systematic-reviews

We identified relevant systematic reviews and meta-analyses on TB in children in PubMed (www.pubmed.ncbi.nlm.nih.gov). Search details are provided in Box A1.1.

Box A1. Search strategy for systematic reviews

Searched in Pubmed for "systematic review meta-analysis tuberculosis children", which is interpreted by the search engine as:

(("systematic review"[Publication Type] OR "systematic reviews as topic"[MeSH Terms]) OR "systematic review"[All Fields]) AND (("meta-analysis"[Publication Type] OR "meta-analysis as topic"[MeSH Terms]) OR "meta-analysis"[All Fields]) AND (((("tuberculosi"[All Fields] OR "tuberculosis"[MeSH Terms]) OR "tuberculosis"[All Fields]) OR "tuberculoses"[All Fields]) OR "tuberculosis s"[All Fields]) AND (((((("child"[MeSH Terms] OR "child"[All Fields]) OR "children"[All Fields]) OR "child s"[All Fields]) OR "children s"[All Fields]) OR "childrens"[All Fields]) OR "childs"[All Fields])

Search date: 19 June 2020.

Of the 150 systematic reviews identified (of which one was a duplicate paper), 23 were judged relevant for full-text review (Figure A1.1). Of the 22 papers reviewed in full-text, four papers contained information about the accuracy of relevant microbiological tests for TB (1-4). However, one of these did not present meta-analytical estimates of the sensitivity and specificity of the test (Xpert Ultra, in this paper) for children (4). Two other papers presented data on the same subject and included roughly the same original work (2, 3), while for one of these, the pooled estimates presented were difficult to interpret as no comparison against culture or Xpert only was included (3). Thus, two papers provided relevant data for extraction (Figure 1): Detjen et al. (1) reported meta-analytic estimates of the sensitivity and specificity of sputum smear microscopy and Xpert on sputum, gastric lavage and nasopharyngeal aspirates (here summarized as 'respiratory samples') against culture of a respiratory sample. Mesman and colleagues (3) presented meta-analytic estimates of the sensitivity and specificity of Xpert stool testing against different reference standards (culture or Xpert on a respiratory sample, bacteriologically confirmed TB, and clinically diagnosed unconfirmed TB).

For peer review only - http://bmjopen.bmj.com/site/about/guidelines.xhtml

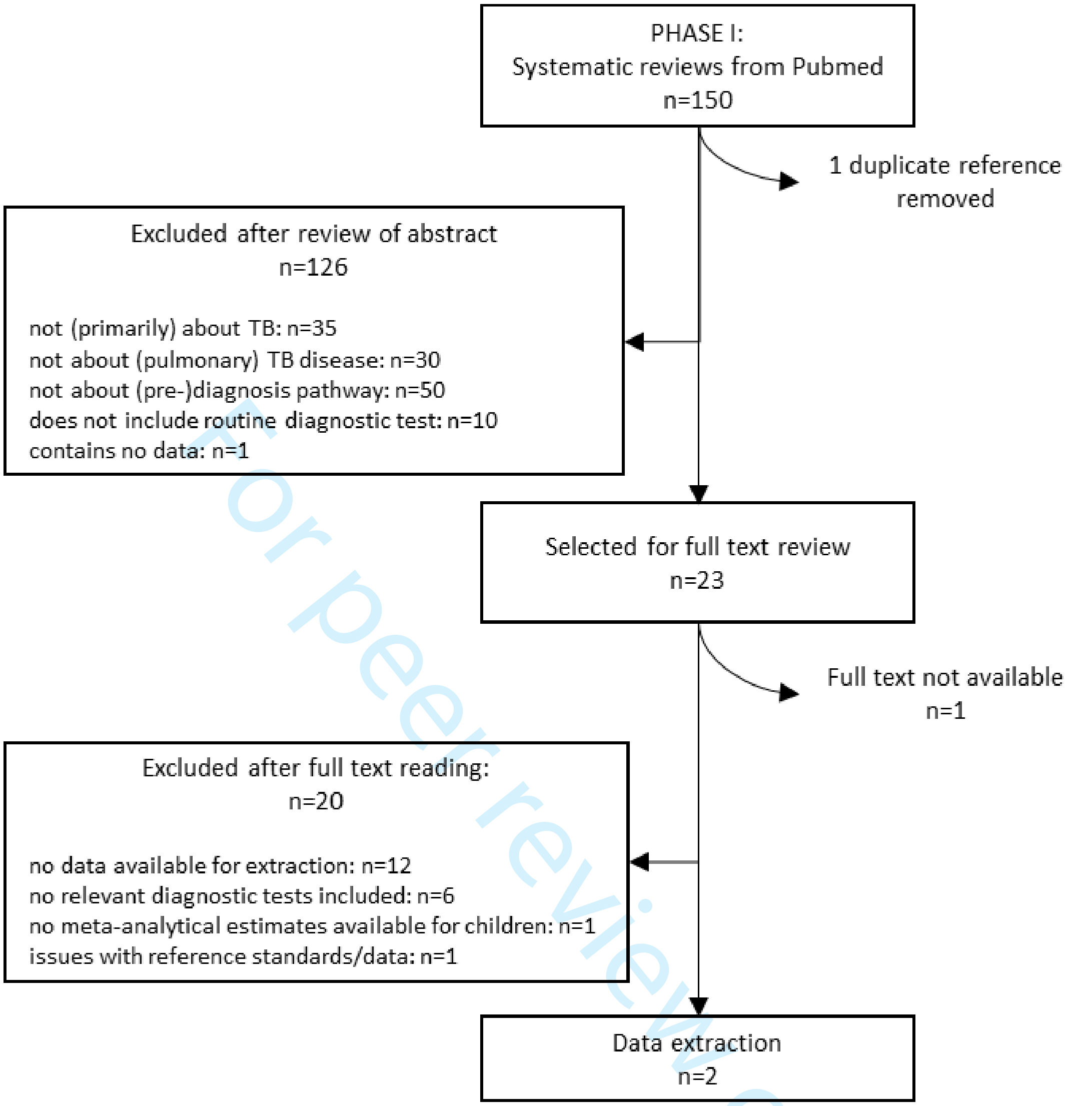

Figure A1. Prisma flow diagram for a search including published peer-reviewed systematic reviews and meta-analysis summarizing literature on TB in children.

Data collection from published peer-reviewed original papers

Next, we conducted a literature search to identify studies about TB diagnostic testing in infants and children, including health care seeking and health care cascade, with a focus on Ethiopia and Indonesia. A systematic search strategy was developed with assistance of an academic librarian from the University of Sheffield. The search strategy used a combination of free-text and thesaurus searching (where available) as outlined in Table A1. Papers with terms for Bacillus Calmette Guérin (BCG), latent tuberculosis, interferon-gamma release assay (IGRA) and tuberculin skin test in titles were excluded from the search as they are relevant to TB infection, but not to active pulmonary TB and were therefore deemed to retrieve irrelevant results. Case reports were excluded as these do not provide generalizable data. The searches were limited to English Language and Human studies published from 2010 - present where databases allowed, except for searches specifically addressing Indonesia/Ethiopia to which no such limits were applied (Table A1). Searches were conducted between 19 and 26 October 2020. Further details of the search strategy are provided in Box A1.2.

For peer review only - http://bmjopen.bmj.com/site/about/guidelines.xhtml

Table A1. Overview of search terms used for searching peer-reviewed original papers.

Exploded MeSH/lookup term	Occurring in title	Occurring in title or abstract[1]
The following were combined using 'AND':		
Tuberculosis or Diagnosis	tuberculosis or TB	
Child or Infant		child or infan[2]
Sputum or Feces		(sputum or stool or f?eces) and (test or sample or specimen)
		test* or diagnos* or screen*
Indonesia or Ethiopia or Developing Countries		Indonesia or Ethiopia or Africa or Asia or West Indies or specific countries[3]
The following were combined with the previous using 'NOT':		
case reports[4]		case report
	bacilli Calmette-Guerin or BCG	
	latent tuberculosis or LTBI or Interferon Gamma Release Assay or IGRA or tuberculin skin test or TST	

[1] In the Cochrane library, key word searches were also included here for all terms, except for the regions and countries; in Medline, this was done only for the terms *child* and *infan*; [2] In Cochrane and Science Citation Index via Web of Science, these terms were replaced with infant*; [3] Specific countries included: Angola, Bangladesh, Benin, Bolivia, Burkina Faso, Burkina Fasso, Burundi, Cambodia, Central African Republic, Chad, Congo, Cote d'Ivoire, Ivory Coast, Djibouti, Egypt, Eritrea, Gambia, Ghana, Guatemala, Guinea, India, Kenya, Korea, Lao PDR, Lesotho, Liberia, Madagascar, Malawi, Mali, Mauritania, Mozambique, Myanmar, Myanma, Nepal, Niger, Nigeria, Philippines, Philipines, Phillipines, Phillippines, Rwanda, Ruanda, Sao Tome, Senegal, Sri Lanka, Somalia, Sudan, Swaziland, Tanzania, Timor-Leste, Togo, Uganda, Vietnam, Viet Nam, Zambia, Zimbabwe; searches for other countries than Ethiopia and Indonesia were limited to English language and humans and *yr="2010 -Current"*; [4] Only included if this lookup term existed for the system (see box 2 for specifications).

In total, 2,974 unique titles were available for title screening, from which 770 were selected for abstract screening. Subsequently, we selected 260 papers for full-text review, of which, after review, data were extracted from 73 (Figure A1.2). The extracted information from these 73 papers was reviewed by the modeling team for its usefulness and applicability to inform the model. Finally, the extracted data for 21 papers was judged to be directly relevant to inform the model. Table A2 provides an overview of all 73 papers for which data was extracted, and specifies which papers were used to inform the model.

For peer review only - http://bmjopen.bmj.com/site/about/guidelines.xhtml

Box A2. Details of search strategy for peer-reviewed original publications

- Developed in MEDLINE
- studies about TB diagnostic testing in infants and children
- two sets of search results:
    - Indonesia or Ethiopia
    - other countries in Africa or Asia (adapting the Cochrane LMIC filter (https://epoc.cochrane.org/lmic-filters).
- Searches were further specified following examination of 100 references in pilot search, excluding terms for BCG, latent tuberculosis, IGRA and tuberculin skin test appearing in titles
- Case reports were excluded where possible

**Database**	**Date Searched**	**Number of References Retrieved (including duplicates)**	**Total N retrieved (including duplicates)**
Ovid MEDLINE(R)<br>1946 to Oct Week 3 2020	23/10/20	Indonesia & Ethiopia = 180<br>Other countries = 1,198	**Indonesia & Ethiopia = 537**<br><br>**Other countries = 4,348**
Ovid MEDLINE(R) and Epub Ahead of Print, In-Process & Other Non-Indexed Citations and Daily<br>2016 to Oct 22, 2020*	23/10/20	Indonesia & Ethiopia = 97<br>Other countries = 790	
Ovid Embase<br>1974 to Oct 23, 2020**	26/10/20	Indonesia & Ethiopia = 237<br>Other countries = 1,783	
Cochrane Database of Systematic Reviews<br>Issue 10 of 12, Oct 2020	19/10/20	Indonesia & Ethiopia = 2<br>Other countries = 11 (plus one protocol)	
Cochrane Central Register of Controlled Trials<br>Issue 10 of 12, Oct 2020	19/10/20	Indonesia & Ethiopia = 4<br>Other countries = 108	
Science Citation Index via Web of Science<br>1900-present	26/10/20<br>19/10/20	Indonesia & Ethiopia = 17<br>Other countries = 457	
Conference Proceedings Citation Index-Science (CPCI-S) via Web of Science<br>1990-present	26/10/20	Indonesia & Ethiopia = 1<br>Other countries = 2	

** English Language and Human limits removed as do not work correctly in MEDLINE In-Process; ** case report(s) not a publication type in Embase*

For peer review only - http://bmjopen.bmj.com/site/about/guidelines.xhtml

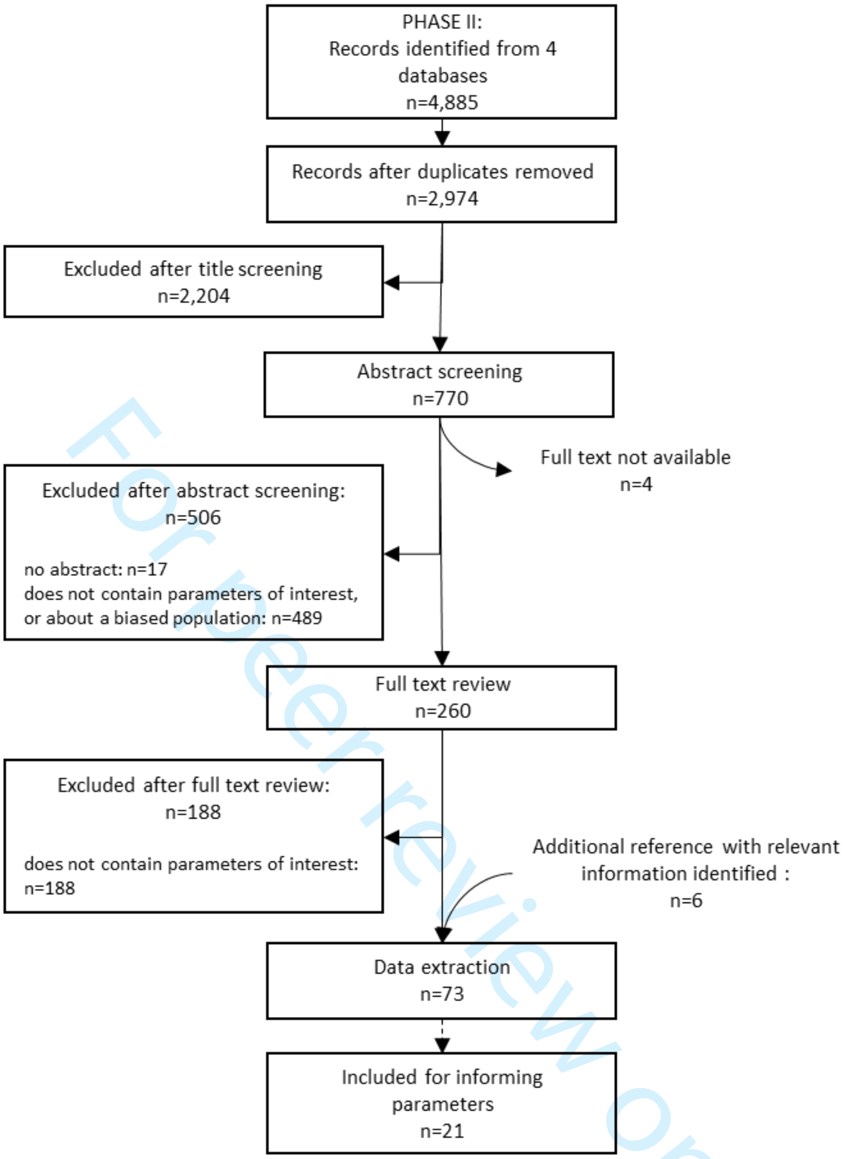

Figure A2. Prisma flow diagram for systematic literature review on TB diagnostic testing in infants and children.

For peer review only

For peer review only - http://bmjopen.bmj.com/site/about/guidelines.xhtml

Table A2. Overview of papers from which data was extracted in the comprehensive literature review of phase 2 (see Figure 2 for details).

Reference (first author and year of publication)	Country	Child population included (N)	Age range	Health care level (no. centers)	Data extracted	Model parameters informed	Comment (e.g., reason for not being considered for informing model parameters)
Andriyoko, 2019 (5)	Indonesia	lab-based, stool plus sputum/NGA submitted for TB diagnosis (36)	0-15y	level 3 (1)	% of presumptive TB cases with confirmed TB		unclear how study population was composed (laboratory study)
Ardizzoni, 2015 (6)	multiple	register review of samples with Xpert results (1,278)	0-14y	NA	% of presumptive TB cases with confirmed TB		Indonesia nor Ethiopia included; data shown on all ages but data extracted for children and (induced) sputum/NGA only
Assefa, 2015 (7)	Ethiopia	household contacts of SS+TB patients (230)	0-5y	level 1 (27)	% of child population seeking care		biased population (semi-active case finding)
Atwebembeire, 2016 (8)	Uganda	lab-based, string test and induced sputum samples (88)	NS	level 3 (1)	% of samples MTB-positive		No information on final TB diagnosis
Bacha, 2017 (9)	Tanzania	presumptive TB or referred for TB treatment (455)	0-14y	level 2/3 (1)	% of presumptive TB cases with confirmed, probable and possible TB	*p_truetb*	
Banada, 2016 (10)	South Africa	consecutive confirmed (20) and probable (20) TB cases	0-14y	level1 (NS) and 2 (NS)	% of samples MTB-positive, by type of sample		Only includes diagnosed TB patients
Bates, 2013 (11)	Zambia	primary or secondary admission diagnosis of TB (930)	0-15y	level 3 (1)	% of presumptive TB cases submitting respiratory specimen for TB diagnosis, by type of specimen and age group<br>% of children with true TB	*spont.sput*	
van Beekhuizen, 1998* (12)	Papua New Guinea	admitted for malnutrition, recurrent pneumonia, or signs/symptoms of TB (301)	0-16y	level 1 (1)	Sensitivity and specificity of clinical diagnosis		Evaluated the sensitivity and specificity of a TB score chart instead of pediatrician's diagnosis
Beneri, 2016 (13)	South Africa	presumptive TB in RCT on TPT for HIV-exposed and -infected (219)	<5y	NA	% of presumptive TB cases with confirmed, probable and possible TB; Sensitivity and specificity of clinical diagnosis		considered for informing *sens.clin* and *spec.clin*, but not a representative population (semi-active case finding)
Berggren-Palme, 2004 (14)	Ethiopia	clinically diagnosed with TB (355)	0-14y	level 3 (1)	% of TB cases submitting spontaneously expectorated sputum for TB diagnosis		only diagnosed TB patients included
Binua, 2019 (15)	Philippines	presumptive TB (incl. EPTB) (112)	4-18y	level 3 (1)	% of presumptive TB cases with definite (smear-positive) TB		abstract only, no detailed information; EPTB included

For peer review only - http://bmjopen.bmj.com/site/about/guidelines.xhtml

Reference (first author and year of publication)	Country	Child population included (N)	Age range	Health care level (no. centers)	Data extracted	Model parameters informed	Comment (e.g., reason for not being considered for informing model parameters)
Bojang, 2016 (16)	The Gambia	presumptive TB (24)	0-14y	level 1 (NS) & research unit (1)	% of presumptive TB cases with definite (Xpert) TB		No information on final TB diagnosis
Brent, 2017 (17)	Kenya	presumptive TB (1,442)	0-14y	level 2 (2)	% of presumptive TB cases with confirmed, highly probable and possible TB		did not use standard clinical case definitions, EPTB included which cannot be separated from pulmonary TB
Bunyasi, 2015 (18)	South Africa	investigated for incident TB in vaccine trial (active & passive FU) (1,020)	<4y	NA	% of presumptive TB cases with confirmed TB		No information on final TB diagnosis; non-representative population
Chipinduro, 2017 (19)	Zimbabwe	presumptive TB (221)	5-16y	level 1 (8)	% of presumptive TB cases submitting induced sputum for TB diagnosis		no data on % spontaneously expectorating sputum
Chisti, 2013 (20)	multiple	acute pneumonia with SAM and/or HIV infection (747)	<5y	NS (NS)	% of children with acute pneumonia being diagnosed with confirmed TB		population not representative for children with presumptive TB
Das, 2019 (21)	India	presumptive TB, partly admitted (171)	0-14y	level 3 (1)	% of presumptive TB cases with confirmed (smear/Xpert-positive) TB		no data on % clinically diagnosed with pulmonary TB
Dayal, 2020 (22)	India	diagnosed with probable TB (114)	0-13y	level 3 (1)	% of samples MTB-positive, by type of sample		Only includes diagnosed TB patients
Elhassan, 2016 (23)	Sudan	presumptive TB (197)	0-13y	level 1 (5)	% of presumptive TB cases with confirmed, probable and possible TB	*p_truetb*	
Eliso, 2015 (24)	Ethiopia	cough of any duration (43)	6-15y	level 1 (4)	% of presumptive TB cases with definite (smear-positive) TB		only smear-positive TB included
Fekadu, 2017* (25)	Ethiopia	NA	NA	level 1 (NA)	% of children seeking care at level 0/1 health facilities first	*phc0_e*	this study used data from multiple sources to estimate TB care cascade
Garcia-Basteiro, 2015 (26)	Mozambique	presumptive TB (766)	0-2y	NA (Research Center) (1)	% of presumptive TB cases with definite and probable TB		possible TB not presented in the paper, limited age bands
Giang, 2015 (27)	Vietnam	presumptive TB (150)	0-14y	level 3 (1)	% of presumptive TB cases with confirmed, probable and possible TB	*p_truetb*	
Gous, 2015 (28)	South Africa	presumptive TB (484)	0-14y	level 2 (1)	% of samples MTB-positive, by method		No information on final TB diagnosis
Hanrahan, 2019 (29)	South Africa	presumptive TB (119)	2m-10y	level 1 (1, high-volume)	% of presumptive TB cases submitting respiratory specimen for TB diagnosis, by type of specimen and age group	*spont.sput, p_truetb*	

For peer review only - http://bmjopen.bmj.com/site/about/guidelines.xhtml

Reference (first author and year of publication)	Country	Child population included (N)	Age range	Health care level (no. centers)	Data extracted	Model parameters informed	Comment (e.g., reason for not being considered for informing model parameters)
Kabir, 2018 (30)	Bangladesh	clinically diagnosed with TB (102)	0-14y	level 3 (1)	% of samples MTB-positive, by method		No information on final TB diagnosis
Kabir, 2020 (31)	Bangladesh	presumptive TB (448)	0-14y	level 3 (1)	% of presumptive TB cases submitting induced sputum and/or stool for TB diagnosis		no data on % spontaneously expectorating sputum
Kalra, 2020 (32)	India	presumptive TB (94,415)	0-14y	level 3 (1)	% of presumptive TB cases submitting any specimen for TB diagnosis, by type of specimen		no data on % spontaneously expectorating sputum
Kalu, 2013 (33)	Nigeria	presumptive TB (263)	3m-14y	level 3 (1)	% of presumptive TB cases with confirmed (culture-positive and/or smear-positive) TB		no data on % clinically diagnosed with pulmonary TB
Lopez-Varela, 2015** (34)	Mozambique	presumptive TB (789)	0-2y	NS (Research Center) (1)	% of presumptive TB cases with definite, probable and possible TB		limited age bands
Marais, 2006* (35)	South Africa	cough>2 weeks without response to oral antibiotics course (428)	0-12y	level 1 (5)	Sensitivity and specificity of clinical diagnosis	*sens.clin, spec.clin*	
Moussa, 2016 (36)	Egypt	presumptive TB (115)	0-15y	level 3 (1)	% of presumptive TB cases with confirmed, probable and possible TB	*p_truetb*	
Mukherjee, 2013 (37)	India	clinically diagnosed intrathoracic TB (403)	6m-15y	level 3 (2)	% of bacteriologically confirmed TB		only includes diagnosed TB patients
Mulenga, 2011 (38)	South Africa	two cohorts investigated for incident TB in two vaccine trials (active & passive FU) (1,445+740)	0-2y?	NA	% of child population seeking care<br><br>Sensitivity and specificity of clinical diagnosis		mixture of PHC and hospital care seeking, limited age bands;<br>contains % with different combinations of symptoms and signs of TB, but no data for parameter of interest
Mulenga, 2015 (39)	South Africa	investigated for incident TB in vaccine trial (active FU) (1,017)	0-2y	NA	Sensitivity and specificity of clinical diagnosis		contains % with different combinations of symptoms and signs of TB, but no data for parameter of interest
Munoz-Sellart, 2009 (40)	Ethiopia	diagnosed with TB (231)	0-14y	level 1 (7) and 2 (1)	% of smear-positive TB		only includes diagnosed TB patients
Mwangwa, 2017 (41)	Uganda	diagnosed with TB in HIV RCT (42)	0-15y	level 1 (32 communities)	% of bacteriologically confirmed TB cases started on treatment		likely higher than SOC
Myo, 2018 (42)	Myanmar	presumptive TB (231)	1m-12y	level 3 (1)	% of presumptive TB cases with confirmed and unconfirmed TB<br>% of bacteriologically confirmed TB cases started on treatment	*p_truetb*	 likely higher than SOC

For peer review only - http://bmjopen.bmj.com/site/about/guidelines.xhtml

Reference (first author and year of publication)	Country	Child population included (N)	Age range	Health care level (no. centers)	Data extracted	Model parameters informed	Comment (e.g., reason for not being considered for informing model parameters)
Nansumba, 2016 (43)	Uganda	presumptive TB (137)	3-14y	level 3 (1)	% of presumptive TB cases submitting respiratory specimen for TB diagnosis; % of presumptive TB cases with confirmed (culture-positive) TB; % of bacteriologically confirmed TB cases started on treatment		no data on % spontaneously expectorating sputum; no data on clinically diagnosed TB; likely higher than SOC
Negash, 2020 (44)	Ethiopia	lab-based, any sputum received for TB diagnosis (414)	4-14y	level 1 (4 hospitals, 34 HCs)	% of presumptive TB cases with Xpert-positive TB		no data on clinically diagnosed TB
Nhu, 2013 (45)	Vietnam	presumptive TB (73)	0-15y	level 3 (1)	% of bacteriologically confirmed TB		only includes diagnosed TB patients
Nicol, 2011 (46)	South Africa	admitted for presumptive TB (452)	0-15y	level 3 (2)	% of bacteriologically confirmed TB; % of bacteriologically confirmed TB cases started on treatment	*Fbc*	only includes in-patients in level-3 hospital likely higher than SOC
Nicol, 2013 (47)	South Africa	presumptive TB (115)	0-14y	level 1 (1) and level 3 (1)	% of presumptive TB cases with confirmed, probable and possible TB	*p_truetb*	none of the children was diagnosed with possible TB
Nicol, 2019 (48)	South Africa	presumptive TB (165)	0-14y	level 3 (1)	% of presumptive TB cases with confirmed and unconfirmed TB; % of bacteriologically confirmed TB cases started on treatment	*p_truetb*	likely higher than SOC
Nissen, 2012 (49)	Tanzania	presumptive TB (195)	0-14y	level 1/2 (1)	% returning for clinical re-evaluation after initial exclusion of TB		likely higher than SOC as asked to return by the study team
Oliwa, 2019 (50)	Kenya	admitted for presumptive TB (23,741)	0-15y	level 2 (13)	% of presumptive TB cases that gets TST, chest X-ray, and bacteriology offered		not regarded sufficiently representative for Ethiopia and Indonesia
Orikiriza, 2018 (51)	Uganda	presumptive TB, partly admitted (392)	1m-14y	level 2/3 (1)	% of presumptive TB cases with confirmed TB, % started on TB treatment		case definitions provided in Methods section were not used to present results
Pearce, 2012* (52)	NA	NA	NA	NA	Sensitivity and specificity of clinical diagnosis		review, no original data; only one study identified providing a sensitivity score
Ramos, 2013 (53)	Ethiopia	retrospective review of sputum reports (875)	0-14y	level 2 (1)	% of presumptive TB cases with smear-positive TB		only smear-positive TB included
Ramos, 2019 (54)	Ethiopia	diagnosed with smear-positive TB (862)	0-14y	level 2 (1)	% of TB patients diagnosed with smear-positive TB		only diagnosed TB patients included

For peer review only - http://bmjopen.bmj.com/site/about/guidelines.xhtml

Reference (first author and year of publication)	Country	Child population included (N)	Age range	Health care level (no. centers)	Data extracted	Model parameters informed	Comment (e.g., reason for not being considered for informing model parameters)
Raizada, 2014 (55)	India	presumptive pulmonary TB (4,600)	0-14y	network of level 1 HF (±400), microscopy centers (99), and sub-district TB units (18)	% of presumptive TB cases with bacteriologically confirmed (by SSM/ Xpert) TB		no data on clinically diagnosed TB
Raizada, 2015 (56)	India	presumptive pulmonary TB (517)	0-14y	as Razaida, 2014 (55)	% of presumptive TB cases with bacteriologically confirmed (by SSM/ Xpert) TB		no data on clinically diagnosed TB
Raizada, 2018a (57)	India	lab-based study, presumptive TB (3,045)	0-14y	central Xpert labs (4) receiving samples from all levels (public & private) in 4 big cities	% of bacteriologically confirmed TB cases started on treatment		also EPTB included, no differentiation by type of TB possible
Raizada, 2018b (58)	India	lab-based study, presumptive TB (465)	<2y	as Razaida, 2018a (57)	% of bacteriologically confirmed TB cases started on treatment		also EPTB included, no differentiation by type of TB possible
Reither, 2015 (59)	Uganda, Tanzania	presumptive TB (451)	2m-15y	NA (Research Center) (2), level 2 (1)	% of presumptive TB cases with confirmed, highly probable and probable TB	*p_truetb*	
Sabi, 2016 (60)	Tanzania	presumptive TB (192)	2m-12y	level 2 (1)	% of presumptive TB cases submitting respiratory specimen for TB diagnosis;<br>% of presumptive TB cases with confirmed, probable and possible TB	*p_truetb*	no data on % spontaneously expectorating sputum
Sabi, 2018 (61)	Tanzania	presumptive TB (277)	6m-16y	NS (Research Center) (2)	% of presumptive TB cases with confirmed, highly probable and probable TB		Focus of report on stored sputum samples tested with Xpert Ultra. More relevant data presented in Reither, 2015
Sekadde, 2013 (62)	Uganda	presumptive TB (235)	2m-12y	level 3 (1)	% of presumptive TB cases submitting induced sputum for TB diagnosis;<br>% of presumptive TB cases with confirmed TB		no data on % spontaneously expectorating sputum;<br>no data on clinically diagnosed TB
Sharma, 2020 (63)	India	non-expectorating with strong clinical suspicion of TB (210)	6m-12y	level 3 (1)	% of presumptive TB cases with bacteriologically confirmed TB		non-expectorating children only; no data on clinically diagnosed TB

For peer review only - http://bmjopen.bmj.com/site/about/guidelines.xhtml

Reference (first author and year of publication)	Country	Child population included (N)	Age range	Health care level (no. centers)	Data extracted	Model parameters informed	Comment (e.g., reason for not being considered for informing model parameters)
Shata, 1996 (64)	Malawi	presumptive TB (29)	3-15y	level 3 (1)	% of presumptive TB cases submitting induced sputum for TB diagnosis		no data on % spontaneously expectorating sputum
Singh, 2016 (65)	India	presumptive TB (50)	0-14y	NS (3)	% of presumptive TB cases with confirmed (SSM) and probable TB		no internationally accepted definition used for the clinical definition of TB
Sorsa, 2020 (66)	Ethiopia	presumptive TB (775)	0-14y	level 3 (1)	% of presumptive TB cases with confirmed (SSM/Xpert) and probable TB	*p_truetb*	
Ssengooba, 2020 (67)	Uganda	diagnosed with "minimal TB" participating in clinical trial (353)	0-15y	NS	% of samples MTB-positive, by type of sample		only diagnosed TB patients included
Surya, 2017* (68)	Indonesia	NA	NA	level 1 (NA)	% of children seeking care at level 0/1 health facilities first	*phc0_i*	this study used data from multiple sources to estimate TB care cascade
Swaminathan, 2008 (69)	India	presumptive TB (2,652)	6m-12y	level 3 (3)	% of presumptive TB cases with bacteriologically confirmed TB		no data on clinically diagnosed TB
Walters, 2017a (70)	South Africa	presumptive intrathoracic TB (188)	0-12y	level 3 (2)	% of presumptive TB cases with confirmed and unconfirmed TB		population is the same as presented in Walters, vd Zalm, 2017
Walters, 2017b (71)	South Africa	presumptive intrathoracic TB (379)	0-12y	level 3 (2)	% TB bacteriologically positive under ideal conditions<br>% of presumptive TB cases with confirmed and unconfirmed TB	*Fbc,*<br>*p_truetb*	
Walters, 2018 (72)	South Africa	presumptive TB (148)	0-15y	level 3 (2)	% of presumptive TB cases submitting stool for TB diagnosis;<br>% of presumptive TB cases with confirmed and unconfirmed TB<br>% started on TB treatment after clinical re-evaluation	*p_truetb*	no data on % spontaneously expectorating sputum<br>likely higher than SOC
Yadav, 2020 (73)	India	presumptive TB (155)	0-15y	level 3 (1)	% of presumptive TB cases submitting respiratory specimen for TB diagnosis, by type of specimen		not clear if spontaneous expectoration was attempted in all children
Zar, 2005 (74)	South Africa	admitted for presumptive TB (250)	1m-5y	level 3 (2)	% of presumptive TB cases with confirmed (SSM/culture) TB		no data on clinically diagnosed TB
Zar, 2012 (75)	South Africa	admitted for different severe conditions with presumptive TB (535)	0-14y	level 3 (2)	% TB bacteriologically positive under ideal conditions<br>% of presumptive TB cases with definite (Xpert/culture) and possible TB	*Fbc*	non-representative population (hospitalized with severe conditions)

For peer review only - http://bmjopen.bmj.com/site/about/guidelines.xhtml

Reference (first author and year of publication)	Country	Child population included (N)	Age range	Health care level (no. centers)	Data extracted	Model parameters informed	Comment (e.g., reason for not being considered for informing model parameters)
Zar, 2013 (76)	South Africa	presumptive TB (384)	0-14y	level 1 (1, high-volume)	% of presumptive TB cases with definite (Xpert/culture) and possible TB<br>% started on TB treatment after clinical re-evaluation	*p_truetb*	likely higher than SOC
Zar, 2019 (77)	South Africa	admitted for presumptive TB (195)	0-14y	level 3 (1)	% TB bacteriologically positive under ideal conditions<br>% of presumptive TB cases with confirmed and unconfirmed TB	*Fbc*	non-representative population (hospitalized with severe conditions)

Abbreviations used in table: EPTB: extrapulmonary tuberculosis, Fu: follow up, HC: health center, HF: healthcare facility, m: months, MTB: *Mycobacterium tuberculosis*, NA: not applicable, NS: not specified, RCT: randomized controlled trial, SOC: standard of care, SSM: sputum smear microscopy, TB: tuberculosis, y: years; * Identified from authors' personal databases, not through the systematic literature review; ** Identified as paper containing the source data of one of the papers identified in the systematic literature review (26).

For peer review only

For peer review only - http://bmjopen.bmj.com/site/about/guidelines.xhtml

References

1. Detjen AK, DiNardo AR, Leyden J, Steingart KR, Menzies D, Schiller I, Dendukuri N, Mandalakas AM. 2015. Xpert MTB/RIF assay for the diagnosis of pulmonary tuberculosis in children: a systematic review and meta-analysis. Lancet Respir Med 3:451-61.
2. MacLean E, Sulis G, Denkinger CM, Johnston JC, Pai M, Ahmad Khan F. 2019. Diagnostic Accuracy of Stool Xpert MTB/RIF for Detection of Pulmonary Tuberculosis in Children: a Systematic Review and Meta-analysis. J Clin Microbiol 57.
3. Mesman AW, Rodriguez C, Ager E, Coit J, Trevisi L, Franke MF. 2019. Diagnostic accuracy of molecular detection of Mycobacterium tuberculosis in pediatric stool samples: A systematic review and meta-analysis. Tuberculosis (Edinb) 119:101878.
4. Zhang M, Xue M, He JQ. 2020. Diagnostic accuracy of the new Xpert MTB/RIF Ultra for tuberculosis disease: A preliminary systematic review and meta-analysis. Int J Infect Dis 90:35-45.
5. Andriyoko B, Janiar H, Kusumadewi R, Klinkenberg E, de Haas P, Tiemersma E. 2019. Simple stool processing method for the diagnosis of pulmonary tuberculosis using GeneXpert MTB/RIF. European Respiratory Journal 53:03.
6. Ardizzoni E, Fajardo E, Saranchuk P, Casenghi M, Page AL, Varaine F, Kosack CS, Hepple P. 2015. Implementing the Xpert R MTB/RIF Diagnostic Test for Tuberculosis and Rifampicin Resistance: Outcomes and Lessons Learned in 18 Countries. PLoS ONE [Electronic Resource] 10:e0144656.
7. Assefa D, Klinkenberg E, Yosef G. 2015. Cross Sectional Study Evaluating Routine Contact Investigation in Addis Ababa, Ethiopia: A Missed Opportunity to Prevent Tuberculosis in Children. PLoS ONE [Electronic Resource] 10:e0129135.
8. Atwebembeire J, Orikiriza P, Bonnet M, Atwine D, Katawera V, Nansumba M, Nyehangane D, Bazira J, Mwanga-Amumpaire J, Byarugaba F, Boum Y. 2016. Xpert(R) MTB/RIF for detection of Mycobacterium tuberculosis from frozen string and induced sputum sediments. International Journal of Tuberculosis & Lung Disease 20:1113-7.
9. Bacha JM, Ngo K, Clowes P, Draper HR, Ntinginya EN, DiNardo A, Mangu C, Sabi I, Mtafya B, Mandalakas AM. 2017. Why being an expert - despite xpert -remains crucial for children in high TB burden settings. BMC Infectious Diseases 17:123.
10. Banada PP, Naidoo U, Deshpande S, Karim F, Flynn JL, O'Malley M, Jones M, Nanassy O, Jeena P, Alland D. 2016. A Novel Sample Processing Method for Rapid Detection of Tuberculosis in the Stool of Pediatric Patients Using the Xpert MTB/RIF Assay. PLoS ONE [Electronic Resource] 11:e0151980.
11. Bates M, O'Grady J, Maeurer M, Tembo J, Chilukutu L, Chabala C, Kasonde R, Mulota P, Mzyece J, Chomba M, Mukonda L, Mumba M, Kapata N, Rachow A, Clowes P, Hoelscher M, Mwaba P, Zumla A. 2013. Assessment of the Xpert MTB/RIF assay for diagnosis of tuberculosis with gastric lavage aspirates in children in sub-Saharan Africa: a prospective descriptive study. The Lancet Infectious Diseases 13:36-42.
12. van Beekhuizen HJ. 1998. Tuberculosis score chart in children in Aitape, Papua New Guinea. Trop Doct 28:155-60.
13. Beneri CA, Aaron L, Kim S, Jean-Philippe P, Madhi S, Violari A, Cotton MF, Mitchell C, Nachman S. 2016. Understanding NIH clinical case definitions for pediatric intrathoracic TB by applying them to a clinical trial. International Journal of Tuberculosis and Lung Disease 20:93-100.
14. Berggren Palme I, Gudetta B, Bruchfeld J, Eriksson M, Giesecke J. 2004. Detection of Mycobacterium tuberculosis in gastric aspirate and sputum collected from Ethiopian HIV-positive and HIV-negative children in a mixed in- and outpatient setting. Acta Paediatrica 93:311-5.
15. Binua F, Tuazon A. 2019. The yield of AFB smear and tb culture in the diagnosis of childhood tb using sputum induction with n-acetylcysteine: a randomized controlled trial. Pediatric pulmonology 54:S79-.
16. Bojang AL, Mendy FS, Tientcheu LD, Otu J, Antonio M, Kampmann B, Agbla S, Sutherland JS. 2016. Comparison of TB-LAMP, GeneXpert MTB/RIF and culture for diagnosis of pulmonary tuberculosis in The Gambia. Journal of Infection 72:332-7.
17. Brent AJ, Mugo D, Musyimi R, Mutiso A, Morpeth SC, Levin M, Scott JAG. 2017. Bacteriological diagnosis of childhood TB: a prospective observational study. Scientific Reports 7:11808.
18. Bunyasi EW, Tameris M, Geldenhuys H, Schmidt BM, Luabeya AK, Mulenga H, Scriba TJ, Hanekom WA, Mahomed H, McShane H, Hatherill M. 2015. Evaluation of Xpert R MTB/RIF Assay in Induced Sputum and Gastric Lavage Samples from Young Children with Suspected Tuberculosis from the MVA85A TB Vaccine Trial. PLoS ONE [Electronic Resource] 10:e0141623.

For peer review only - http://bmjopen.bmj.com/site/about/guidelines.xhtml

19. Chipinduro M, Mateveke K, Makamure B, Ferrand RA, Gomo E. 2017. Stool Xpert^R MTB/RIF test for the diagnosis of childhood pulmonary tuberculosis at primary clinics in Zimbabwe. International Journal of Tuberculosis & Lung Disease 21:161-166.
20. Chisti MJ, Ahmed T, Pietroni MA, Faruque AS, Ashraf H, Bardhan PK, Hossain I, Das SK, Salam MA. 2013. Pulmonary tuberculosis in severely-malnourished or HIV-infected children with pneumonia: a review. Journal of Health, Population & Nutrition 31:308-13.
21. Das A, Anupurba S, Mishra OP, Banerjee T, Tripathi R. 2019. Evaluation of Xpert MTB/RIF Assay for Diagnosis of Tuberculosis in Children. Journal of Tropical Pediatrics 65:14-20.
22. Dayal R, Yadav A, Agarwal D, Kumar M, Kamal R, Singh D, Bhatnagar S. 2020. Comparison of Diagnostic Yield of Tuberculosis Loop-Mediated Isothermal Amplification Assay With Cartridge-Based Nucleic Acid Amplification Test, Acid-Fast Bacilli Microscopy, and Mycobacteria Growth Indicator Tube Culture in Children With Pulmonary Tuberculosis. Journal of the Pediatric Infectious Diseases Societ 10:10.
23. Elhassan MM, Elmekki MA, Osman AL, Hamid ME. 2016. Challenges in diagnosing tuberculosis in children: a comparative study from Sudan. International Journal of Infectious Diseases 43:25-29.
24. Eliso E, Medhin G, Belay M. 2015. Prevalence of smear positive pulmonary tuberculosis among outpatients presenting with cough of any duration in Shashogo Woreda, Southern Ethiopia. BMC Public Health 15:112.
25. Fekadu L, Hanson C, Osberg M, Makayova J, Mingkwan P, Chin D. 2017. Increasing Access to Tuberculosis Services in Ethiopia: Findings From a Patient-Pathway Analysis. J Infect Dis 216:S696-S701.
26. Garcia-Basteiro AL, Lopez-Varela E, Augusto OJ, Gondo K, Munoz J, Sacarlal J, Marais B, Alonso PL, Ribo JL. 2015. Radiological findings in young children investigated for tuberculosis in Mozambique. PLoS ONE [Electronic Resource] 10:e0127323.
27. Giang do C, Duong TN, Ha DT, Nhan HT, Wolbers M, Nhu NT, Heemskerk D, Quang ND, Phuong DT, Hang PT, Loc TH, Lan NT, Dung NH, Farrar J, Caws M. 2015. Prospective evaluation of GeneXpert for the diagnosis of HIV- negative pediatric TB cases. BMC Infectious Diseases 15:70.
28. Gous N, Scott LE, Khan S, Reubenson G, Coovadia A, Stevens W. 2015. Diagnosing childhood pulmonary tuberculosis using a single sputum specimen on Xpert MTB/RIF at point of care. South African Medical Journal Suid-Afrikaanse Tydskrif Vir Geneeskunde 105:1044-8.
29. Hanrahan CF, Dansey H, Mutunga L, France H, Omar SV, Ismail N, Bassett J, Van Rie A. 2019. Diagnostic strategies for childhood tuberculosis in the context of primary care in a high burden setting: the value of alternative sampling methods. Paediatrics & international Child Health 39:88-94.
30. Kabir S, Uddin MKM, Chisti MJ, Fannana T, Haque ME, Uddin MR, Banu S, Ahmed T. 2018. Role of PCR method using IS6110 primer in detecting Mycobacterium tuberculosis among the clinically diagnosed childhood tuberculosis patients at an urban hospital in Dhaka, Bangladesh. International Journal of Infectious Diseases 68:108-114.
31. Kabir S, Rahman SMM, Ahmed S, Islam MS, Banu RS, Shewade HD, Thekkur P, Anwar S, Banu NA, Nasrin R, Uddin MKM, Choudhury S, Ahmed S, Paul KK, Khatun R, Chisti MJ, Banu S. 2020. Xpert Ultra assay on stool to diagnose pulmonary tuberculosis in children. Clinical Infectious Diseases 18:18.
32. Kalra A, Parija D, Raizada N, Sachdeva KS, Rao R, Swaminathan S, Khanna A, Chopra KK, Hanif M, Singh V, Umadevi KR, Sheladia KN, Rao R, Vasundhara N, S A, A RN, Azeem A, Chhajlani V, Khurana J, Das NJ, Choudhury B, Nair SA, Mall S, Sen R, Chadha SS, Denkinger CM, Boehme C, Sarin S. 2020. Upfront Xpert MTB/RIF for diagnosis of pediatric TB-Does it work? Experience from India. PLoS ONE [Electronic Resource] 15:e0236057.
33. Kalu EI, Ojide CK, Ugochukwu NV. 2013. Gastric aspirate smear microscopy as a diagnostic tool for childhood pulmonary tuberculosis. Annals of Tropical Medicine and Public Health 6:608-613.
34. Lopez-Varela E, Augusto OJ, Gondo K, Garcia-Basteiro AL, Fraile O, Ira T, Ribo Aristizabal JL, Bulo H, Munoz Gutierrez J, Aponte J, Macete E, Sacarlal J, Alonso PL. 2015. Incidence of Tuberculosis Among Young Children in Rural Mozambique. Pediatric Infectious Disease Journal 34:686-92.
35. Marais BJ, Gie RP, Hesseling AC, Schaaf HS, Lombard C, Enarson DA, Beyers N. 2006. A refined symptom-based approach to diagnose pulmonary tuberculosis in children. Pediatrics 118:E1350-E1359.
36. Moussa H, Bayoumi FS, Mohamed AM. 2016. Gene Xpert for Direct Detection of Mycobacterium Tuberculosis in Stool Specimens from Children with Presumptive Pulmonary Tuberculosis. Annals of Clinical & Laboratory Science 46:198-203.

For peer review only

For peer review only - http://bmjopen.bmj.com/site/about/guidelines.xhtml

37. Mukherjee A, Singh S, Lodha R, Singh V, Hesseling AC, Grewal HM, Kabra SK, Delhi Pediatric TBSG. 2013. Ambulatory gastric lavages provide better yields of Mycobacterium tuberculosis than induced sputum in children with intrathoracic tuberculosis. Pediatric Infectious Disease Journal 32:1313-7.
38. Mulenga H, Moyo S, Workman L, Hawkridge T, Verver S, Tameris M, Geldenhuys H, Hanekom W, Mahomed H, Hussey G, Hatherill M. 2011. Phenotypic variability in childhood TB: implications for diagnostic endpoints in tuberculosis vaccine trials. Vaccine 29:4316-21.
39. Mulenga H, Tameris MD, Luabeya KKA, Geldenhuys H, Scriba TJ, Hussey GD, Mahomed H, Landry BS, Hanekom WA, McShane H, Hatherill M. 2015. The role of clinical symptoms in the diagnosis of intrathoracic tuberculosis in young children. Pediatric Infectious Disease Journal 34:1157-1162.
40. Munoz-Sellart M, Yassin MA, Tumato M, Merid Y, Cuevas LE. 2009. Treatment outcome in children with tuberculosis in southern Ethiopia. Scandinavian Journal of Infectious Diseases 41:450-5.
41. Mwangwa F, Chamie G, Kwarisiima D, Ayieko J, Owaraganise A, Ruel TD, Plenty A, Tram KH, Clark TD, Cohen CR, Bukusi EA, Petersen M, Kamya MR, Charlebois ED, Havlir DV, Marquez C. 2017. Gaps in the Child Tuberculosis Care Cascade in 32 Rural Communities in Uganda and Kenya. Journal of Clinical Tuberculosis and Other Mycobacterial Diseases 9:24-29.
42. Myo K, Zaw M, Swe TL, Kyaw YY, Thwin T, Myo TT, Aye KO, Myint AA. 2018. Evaluation of Xpert MTB/RIF assay as a diagnostic test for pulmonary tuberculosis in children in Myanmar. International Journal of Tuberculosis and Lung Disease 22:1051-1055.
43. Nansumba M, Kumbakumba E, Orikiriza P, Muller Y, Nackers F, Debeaudrap P, Boum Y, 2nd, Bonnet M. 2016. Detection Yield and Tolerability of String Test for Diagnosis of Childhood Intrathoracic Tuberculosis. Pediatric Infectious Disease Journal 35:146-51.
44. Negash H, Legese H, Adhanom G, Mardu F, Tesfay K, Gebremeskel SG, Berhe B. 2020. Six years trend analysis of tuberculosis in Northwestern Tigrai, Ethiopia; 2019: A retrospective study. Infection and Drug Resistance 13:643-649.
45. Nhu NT, Ha DT, Anh ND, Thu DD, Duong TN, Quang ND, Lan NT, Quyet TV, Tuyen NT, Ha VT, Giang DC, Dung NH, Wolbers M, Farrar J, Caws M. 2013. Evaluation of Xpert MTB/RIF and MODS assay for the diagnosis of pediatric tuberculosis. BMC Infectious Diseases 13:31.
46. Nicol MP, Zar HJ. 2011. New specimens and laboratory diagnostics for childhood pulmonary TB: progress and prospects. Paediatric Respiratory Reviews 12:16-21.
47. Nicol MP, Spiers K, Workman L, Isaacs W, Munro J, Black F, Zemanay W, Zar HJ. 2013. Xpert MTB/RIF testing of stool samples for the diagnosis of pulmonary tuberculosis in children. Clinical Infectious Diseases 57:e18-21.
48. Nicol MP, Wood RC, Workman L, Prins M, Whitman C, Ghebrekristos Y, Mbhele S, Olson A, Jones-Engel LE, Zar HJ, Cangelosi GA. 2019. Microbiological diagnosis of pulmonary tuberculosis in children by oral swab polymerase chain reaction. Scientific Reports 9:10789.
49. Nissen TN, Rose MV, Kimaro G, Bygbjerg IC, Mfinanga SG, Ravn P. 2012. Challenges of loss to follow-up in tuberculosis research. PLoS ONE [Electronic Resource] 7:e40183.
50. Oliwa JN, Gathara D, Ogero M, van Hensbroek MB, English M, Van't Hoog A, Clinical Information N. 2019. Diagnostic practices and estimated burden of tuberculosis among children admitted to 13 government hospitals in Kenya: An analysis of two years' routine clinical data. PLoS ONE [Electronic Resource] 14:e0221145.
51. Orikiriza P, Nansumba M, Nyehangane D, Bastard M, Mugisha IT, Nansera D, Mwanga-Amumpaire J, Boum Y, 2nd, Kumbakumba E, Bonnet M. 2018. Xpert MTB/RIF diagnosis of childhood tuberculosis from sputum and stool samples in a high TB-HIV-prevalent setting. European Journal of Clinical Microbiology & Infectious Diseases 37:1465-1473.
52. Pearce EC, Woodward JF, Nyandiko WM, Vreeman RC, Ayaya SO. 2012. A systematic review of clinical diagnostic systems used in the diagnosis of tuberculosis in children. Aids Research and Treatment:401896.
53. Ramos JM, Perez-Butragueno M, Tisiano G, Yohannes T, Reyes F, Gorgolas M. 2013. Evaluation of Ziehl-Neelsen smear for diagnosis of pulmonary tuberculosis in childhood in a rural hospital in Ethiopia. International Journal of Mycobacteriology 2:171-3.
54. Ramos JM, Perez-Butragueno M, Tesfamariam A, Reyes F, Tiziano G, Endirays J, Balcha S, Elala T, Biru D, Comeche B, Gorgolas M. 2019. Comparing tuberculosis in children aged under 5 versus 5 to 14 years old in a rural hospital in southern Ethiopia: an 18-year retrospective cross-sectional study. BMC Public Health 19:856.
55. Raizada N, Sachdeva KS, Nair SA, Kulsange S, Gupta RS, Thakur R, Parmar M, Gray C, Ramachandran R, Vadera B, Ekka S, Dhawan S, Babre A, Ghedia M, Alavadi U, Dewan P, Khetrapal M, Khanna A,

For peer review only - http://bmjopen.bmj.com/site/about/guidelines.xhtml

Boehme C, Paramsivan CN. 2014. Enhancing TB case detection: experience in offering upfront Xpert MTB/RIF testing to pediatric presumptive TB and DR TB cases for early rapid diagnosis of drug sensitive and drug resistant TB. PLoS ONE [Electronic Resource] 9:e105346.

56. Raizada N, Sachdeva KS, Swaminathan S, Kulsange S, Khaparde SD, Nair SA, Khanna A, Chopra KK, Hanif M, Sethi GR, Umadevi KR, Keshav Chander G, Saha B, Shah A, Parmar M, Ghediya M, Jaju J, Boehme C, Paramasivan CN. 2015. Piloting Upfront Xpert MTB/RIF Testing on Various Specimens under Programmatic Conditions for Diagnosis of TB & DR-TB in Paediatric Population. PLoS ONE [Electronic Resource] 10:e0140375.

57. Raizada N, Khaparde SD, Salhotra VS, Rao R, Kalra A, Swaminathan S, Khanna A, Chopra KK, Hanif M, Singh V, Umadevi KR, Nair SA, Huddart S, Prakash CHS, Mall S, Singh P, Saha BK, Denkinger CM, Boehme C, Sarin S. 2018. Accelerating access to quality TB care for pediatric TB cases through better diagnostic strategy in four major cities of India. PLoS ONE [Electronic Resource] 13:e0193194.

58. Raizada N, Khaparde SD, Rao R, Kalra A, Sarin S, Salhotra VS, Swaminathan S, Khanna A, Chopra KK, Hanif M, Singh V, Umadevi KR, Nair SA, Huddart S, Tripathi R, Surya Prakash CH, Saha BK, Denkinger CM, Boehme C. 2018. Upfront Xpert MTB/RIF testing on various specimen types for presumptive infant TB cases for early and appropriate treatment initiation. PLoS ONE [Electronic Resource] 13:e0202085.

59. Reither K, Manyama C, Clowes P, Rachow A, Mapamba D, Steiner A, Ross A, Mfinanga E, Sasamalo M, Nsubuga M, Aloi F, Cirillo D, Jugheli L, Lwilla F. 2015. Xpert MTB/RIF assay for diagnosis of pulmonary tuberculosis in children: a prospective, multi-centre evaluation. Journal of Infection 70:392-9.

60. Sabi I, Kabyemera R, Mshana SE, Kidenya BR, Kasanga G, Gerwing-Adima LE, Meremo A, Clowes P, Rachow A, Peck RN. 2016. Pulmonary TB bacteriologically confirmed by induced sputum among children at Bugando Medical Centre, Tanzania. International Journal of Tuberculosis & Lung Disease 20:228-34.

61. Sabi I, Rachow A, Mapamba D, Clowes P, Ntinginya NE, Sasamalo M, Kamwela L, Haraka F, Hoelscher M, Paris DH, Saathoff E, Reither K. 2018. Xpert MTB/RIF Ultra assay for the diagnosis of pulmonary tuberculosis in children: a multicentre comparative accuracy study. Journal of Infection 77:321-327.

62. Sekadde MP, Wobudeya E, Joloba ML, Ssengooba W, Kisembo H, Bakeera-Kitaka S, Musoke P. 2013. Evaluation of the Xpert MTB/RIF test for the diagnosis of childhood pulmonary tuberculosis in Uganda: a cross-sectional diagnostic study. BMC Infectious Diseases 13:133.

63. Sharma S, Sarin R, Sahu G, Shukla G. 2020. Demographic profile, clinical and microbiological predictors of mortality amongst admitted pediatric TB patients in a tertiary referral tuberculosis hospital. Indian Journal of Tuberculosis 67:312-319.

64. Shata AMA, Coulter JBS, Parry CM, Chingani G, Broadhead RL, Hart CA. 1996. Sputum induction for the diagnosis of tuberculosis. Archives of Disease in Childhood 74:535-537.

65. Singh M, Sethi GR, Mantan M, Khanna A, Hanif M. 2016. Xpert MTB/RIF assay for the diagnosis of pulmonary tuberculosis in children. International Journal of Tuberculosis and Lung Disease 20:839-843.

66. Sorsa A, Jerene D, Negash S, Habtamu A. 2020. Use of Xpert Contributes to Accurate Diagnosis, Timely Initiation, and Rational Use of Anti-TB Treatment Among Childhood Tuberculosis Cases in South Central Ethiopia. Pediatric Health Medicine & Therapeutics 11:153-160.

67. Ssengooba W, Iragena JD, Nakiyingi L, Mujumbi S, Wobudeya E, Mboizi R, Boulware D, Meya DB, Choo L, Crook AM, Lebeau K, Joloba M, Demers AM, Cresswell FV, Gibb DM. 2020. Accuracy of Xpert Ultra in Diagnosis of Pulmonary Tuberculosis among Children in Uganda: a Substudy from the SHINE Trial. Journal of Clinical Microbiology 58:24.

68. Surya A, Setyaningsih B, Suryani Nasution H, Gita Parwati C, Yuzwar YE, Osberg M, Hanson CL, Hymoff A, Mingkwan P, Makayova J, Gebhard A, Waworuntu W. 2017. Quality Tuberculosis Care in Indonesia: Using Patient Pathway Analysis to Optimize Public-Private Collaboration. J Infect Dis 216:S724-S732.

69. Swaminathan S, Datta M, Radhamani MP, Mathew S, Reetha AM, Rajajee S, Mathew R, Radkhakrishnan A, Raghu MB. 2008. A profile of bacteriologically confirmed pulmonary tuberculosis in children. Indian Pediatrics 45:743-747.

70. Walters E, Demers AM, van der Zalm MM, Whitelaw A, Palmer M, Bosch C, Draper HR, Gie RP, Hesseling AC. 2017. Stool Culture for Diagnosis of Pulmonary Tuberculosis in Children. Journal of Clinical Microbiology 55:3355-3365.

71. Walters E, van der Zalm MM, Palmer M, Bosch C, Demers AM, Draper H, Goussard P, Schaaf HS, Friedrich SO, Whitelaw A, Warren R, Gie RP, Hesseling AC. 2017. Xpert MTB/RIF on Stool Is Useful for the Rapid Diagnosis of Tuberculosis in Young Children With Severe Pulmonary Disease. Pediatric Infectious Disease Journal 36:837-843.

For peer review only

For peer review only - http://bmjopen.bmj.com/site/about/guidelines.xhtml

72. Walters E, Scott L, Nabeta P, Demers AM, Reubenson G, Bosch C, David A, van der Zalm M, Havumaki J, Palmer M, Hesseling AC, Ncayiyana J, Stevens W, Alland D, Denkinger C, Banada P. 2018. Molecular Detection of Mycobacterium tuberculosis from Stools in Young Children by Use of a Novel Centrifugation-Free Processing Method. Journal of Clinical Microbiology 56:09.

73. Yadav R, Vaidya P, Mathew JL, Singh S, Khaneja R, Agarwal P, Singh M, Sethi S. 2020. Diagnostic accuracy of Xpert MTB/RIF Ultra for detection of Mycobacterium tuberculosis in children: a prospective cohort study. Letters in Applied Microbiology 08:08.

74. Zar HJ, Honslo D, Apolles P, Swingler G, Hussey G. 2005. Induced sputum versus gastric lavage for microbiological confirmation of pulmonary tuberculosis in infants and young children: a prospective study. Lancet 365:130-134.

75. Zar HJ, Workman L, Isaacs W, Munro J, Black F, Eley B, Allen V, Boehme CC, Zemanay W, Nicol MP. 2012. Rapid molecular diagnosis of pulmonary tuberculosis in children using nasopharyngeal specimens. Clinical Infectious Diseases 55:1088-95.

76. Zar HJ, Workman L, Isaacs W, Dheda K, Zemanay W, Nicol MP. 2013. Rapid diagnosis of pulmonary tuberculosis in African children in a primary care setting by use of Xpert MTB/RIF on respiratory specimens: a prospective study. The Lancet Global Health 1:e97-e104.

77. Zar HJ, Workman LJ, Prins M, Bateman LJ, Mbhele SP, Whitman CB, Denkinger CM, Nicol MP. 2019. Tuberculosis Diagnosis in Children Using Xpert Ultra on Different Respiratory Specimens. American Journal of Respiratory & Critical Care Medicine 200:1531-1538.

For peer review only

For peer review only - http://bmjopen.bmj.com/site/about/guidelines.xhtml

Xpert on stool to diagnose tuberculosis in children is cost-effective in Ethiopia and Indonesia: a model-based cost-effectiveness analysis

Appendix 2a: Model parameter estimation

Model structure and description 2

Description of parameters from review 4

- Spontaneous sputum expectoration (parameter a) 4
- Fraction of TB bacteriologically positive under ideal conditions (parameter Fbc) 6
- Prevalence of true TB among presumptive TB 8
- Accuracy of clinical assessment in bacteriologically negative TB 13
- Accuracy of bacteriological tests 14
- Level of initial care-seeking 15
- Summary of model parameters from review and distributions 16
- Summary of other parameters 16
  - Parameters in common between countries from previous work 16
  - Parameters specific to Indonesia 17
  - Parameters specific to Ethiopia 17
  - Other parameters based on assumption 17

Description approach to expert opinion 17

References 19

For peer review only - http://bmjopen.bmj.com/site/about/guidelines.xhtml

Model structure and description

The model is implemented as a decision tree that matches our understanding of patient pathways of care. The structure of the tree is shown in Figure A1 along with the names of the probabilities of going down each path, and the names of the costs associated with each node (underneath node names; 0 if no costs are applied). Quantities such as probabilities or costs can depend on 'attributes' of patients entering; here, this means true TB status (yes or no) and child age group (0-4 or 5-14 years). The model calculates mean values of various quantities over the tree for a large number (10 thousand) of sampled input parameters and cohort characteristics (i.e., make up by attribute) to generate a probabilistic sensitivity analysis that is used for the generation of results. The quantities calculated over the tree are: the number of deaths; the cost to healthcare providers; the number of referrals; the number of assessments performed; the number of bacteriological assessments performed; the number of anti-TB treatments; the number of bacteriologically-confirmed anti-TB treatments; the number of anti-TB treatments initiated at PHC level; the number of anti-TB treatments initiated among bacteriologically-confirmed TB cases; a validation variable that should always total 1. The model was implemented in R using the HEdtree package.

All fundamental input parameters are treated as random variables with specified distributions to represent uncertainty. Labelled parameters on Figure A1 may depend in specified ways on these underlying fundamental input parameters. Most parameters appearing as labels in Figure A1 directly correspond to fundamental input parameters, and are named as such in parameter tables. However, there are three classes of exception: 1) parameters describing treatment and non-treatment outcomes; 2) parameters on early stages of the care cascade relating to bacteriological testing; 3) parameters describing the prevalence of attributes in the patient cohort, which are not shown on Figure A1.

The approach to outcomes (class 1 above) are based on previously published work[1] and are recapped below (see Table A9), along with some additional modelling details. This document focuses on the review work to inform new input parameters, many of which are related to parameters in classes 2 and 3 above.

Briefly, we assume that parameters *a* are determined by the ability of children in each age category to spontaneously expectorate sputum, i.e., an attempt to collect a spontaneously expectorate sputum is always made at PHC or hospital. Parameters *b* are based on data on the diagnostic accuracy of stool-approaches, but assume that only a fraction of all children in each age group (*Fbc*) are bacteriologically-confirmable under ideal circumstances. Since diagnostic accuracy is typically reported with respect to confirmed cases, we assume test sensitivity only applied to a fraction of *Fbc* of patients. Parameters *f, d,* and *h* for clinical assessments at PHC or hospital are assumed to be the same, and are informed by data we found to inform the diagnostic accuracy for clinical diagnosis of TB in each age group (i.e., these are sensitivity for true TB, and one minus specificity for true not TB). Importantly, we assume that under the intervention, a bacteriologically negative test is always followed up with clinical assessment (i.e., this assessment, which will be made in any case, is able to override a false-negative test result with unchanged sensitivity).

For peer review only - http://bmjopen.bmj.com/site/about/guidelines.xhtml

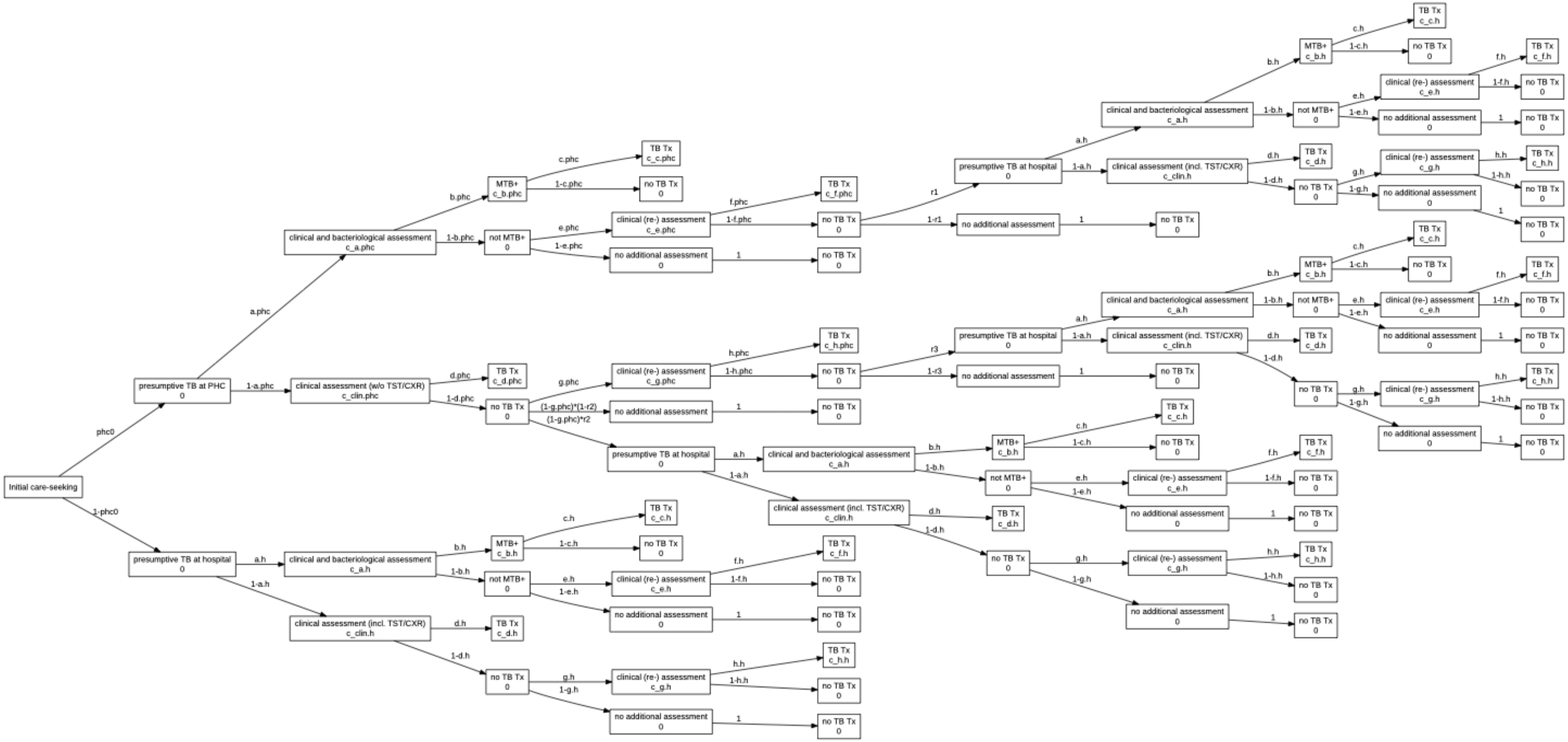

Figure A1 Diagram of modelled decision tree including the edge labels (names of probabilities for each path) and node cost names (underneath node names)

Description of parameters from review

The model was informed with parameters obtained from ongoing studies using the SOS stool method where available, a systematic literature search (see Appendix 1), and expert opinion for those parameters for which no published data was identified. This Appendix provides an overview of the original data and the summation methods used to quantify a parameter for each of the parameters used in the model.

Spontaneous sputum possible

For this parameter, we collected data from our own and published studies on the proportion of children that submitted a spontaneously expectorated sputum sample for diagnostic workup. We included those studies that accepted spontaneously expectorated sputum from all children that could produce such a sample and included other respiratory specimens (i.e., nasogastric (lavage) aspirates, nasopharyngeal aspirates, or induced sputum) for those children unable to spontaneously expectorate, reporting the number of specimens by type received per age group. Only two studies included in our comprehensive review of original peer-reviewed papers (Appendix 1) met these criteria[2, 3].

Table A1 Proportion of children who submitted spontaneously expectorated sputum, by age group.

Reference	Setting	age group	Number of children	Proportion spontaneous sputum (95% CI if provided)
Kaswandani, Tiemersma et al, unpublished	in- and outpatients with symptoms or signs of presumptive TB in 10 secondary and tertiary care hospitals on Java, Indonesia	0-4 years	222	1.80% (0.67% - 4.72%)
		5-10 years	82	13.41% (7.54% - 22.73%)
Bates et al[2]	in-patients with primary or secondary admission diagnosis of TB at pediatric and child health department of Lusaka University hospital in Zambia	0-4 years	663	2.30%
		5-9 years	124	45.20%
		10-14 years	138	50.00%
Hanrahan et al[3]	outpatients with presumptive TB at 1 primary care clinic in Johannesburg, South Africa	2 months - 4 years	202	3.90%
		5-9 years	17	58.80%

Bates et al[2] collected sputum samples from all children who could expectorate while gastric lavage aspirates were obtained from children unable to expectorate. Hanrahan et al[3] collected a spontaneous sputum sample whenever possible. Sputum collection was guided and overseen by a dedicated paediatrician. If the child was unable to expectorate, one nasopharyngeal aspirate and one induced sputum sample were obtained by a nurse. We also collected relevant data in a study in Indonesia on the diagnostic accuracy of the SOS stool method with Xpert. Sample collection was overseen as per routine procedures in the facilities, but was usually done by a nurse. Collection of a

For peer review only - http://bmjopen.bmj.com/site/about/guidelines.xhtml

spontaneously expectorated sputum or an alternative specimen (either sputum induction (generally for children of 2 years and older) or nasogastric aspiration (for younger children)) was conducted as per nurse's judgement.

Table A1 summarizes the data extracted while Figure A2 plots the same data with 95% confidence intervals, using binomial confidence intervals only where counts were provided.
The proportions from the Indonesian studies were lower than those reported from the two published studies, especially for the older children, but may in fact be closer to the reality on the ground, as in the Indonesian study, no special efforts were undertaken to obtain spontaneous sputum from all children.

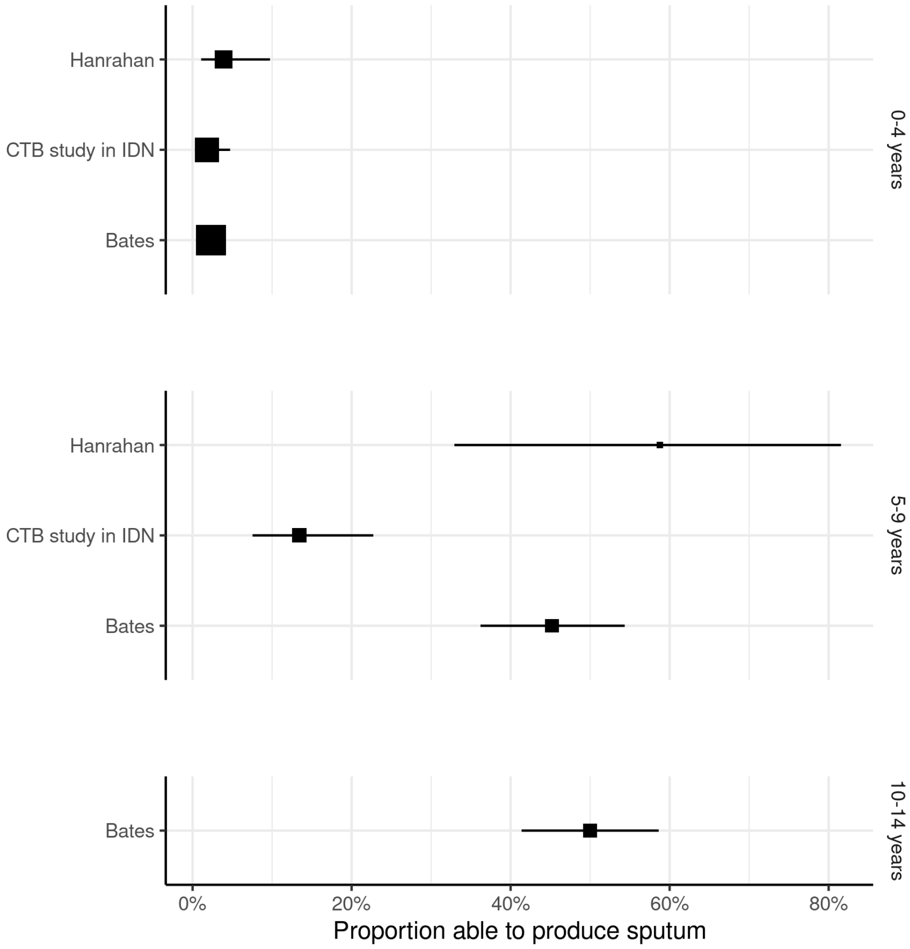

Figure A2. Proportion of children able to spontaneously expectorate sputum by age group with 95% uncertainty intervals.
Names refer to first authors of papers (also described in Table A1 and Appendix 1, Table A2). CTB: Challenge TB project; IDN: Indonesia.

The summary estimates from a random effects meta-analysis after pooling of the results were 2.4% (95% prediction interval (PI): 1.6 - 3.6%) for the rate of spontaneous sputum expectoration among children aged 0-4 years, and 38.9% (95% prediction interval [PI]: 0.098 - 78.8%) among children aged 5 years and above. Note that the 95%CI in this case was much narrower (21.7% - 59.4%).

Fraction of children bacteriologically confirmable

TB in children, especially children under 5 years of age, is often of paucibacillary nature and often no *M. tuberculosis* bacilli can be detected. Most evidence on the sensitivity of diagnostic tests is reported against a gold standard based on bacteriological confirmation; often sensitivity is very poor among children with bacteriologically negative TB. For our model, it was therefore important to understand what the maximum fraction of children for whom TB could be bacteriologically confirmed if an array of diagnostic tests were used. For this parameter, F_{bc}, we included studies that tested multiple different specimens of the same child, using sensitive diagnostics such as Mycobacteria Growth Indicator Tube (MGIT) culture and GeneXpert (Ultra). Four studies meeting these criteria were identified (Table A2). Figure A3 provides the point estimates with 95% uncertainty intervals.

It should be noted that all these four studies were conducted in Cape Town, South Africa, in only 4 different hospitals (Red Cross War Memorial Children's Hospital[4-6], New Somerset Hospital[4-6], Tygerberg Hospital[7] and Karl Bremer Hospital[7]), and included hospitalized children only. Restriction of the study populations to admitted (i.e., most ill) children may introduce bias to higher proportions of confirmable TB, as a positive correlation between bacterial load and seriousness of the illness is expected. For example, in another study including children with minimal TB (defined as non-severe, symptomatic, smear-negative TB), the disease was bacteriologically confirmed on a respiratory sample in only 14.16% of the cases.[8] All children had submitted at least 2 specimens of gastric lavage, gastric washing or sputum, which were tested by culture (MGIT and Lowenstein-Jensen medium), Xpert MTB/Rif and Xpert Ultra.

For peer review only

Table A2 Fraction of TB that was bacteriologically confirmed from studies applying sensitive diagnostics to multiple specimens.

**Reference**	**Setting**	**Type and number of specimens taken**	**Type of diagnostic tests conducted**	**Number of children enrolled**	**Number of children treated for TB**	**Bacteriological confirmation**	**Fraction with bacteriological confirmation of TB**
Nicol et al[4]	children aged <15 years admitted with presumptive pulmonary TB (incl. at least cough of >2weeks plus another sign or symptom) to 2 hospitals in Cape Town, South Africa	2 IS taken at least 4h apart; n=385 with 2 IS, n=67 with one IS specimen	Fluorescent smear microscopy and Xpert MTB/Rif on concentrated sample, MGIT culture	452	n=216: 69/70 definite TB , 147/216 possible TB (incl. 6 with Xpert MTB+ results)	n=76: 70 culture-positive, 6 Xpert positive, culture-negative	34.72%
Walters et al[9]	children aged <13 years presenting to 2 hospitals in Cape Town, South Africa with presumptive intrathoracic TB	sputum (5 years or older)/NGA (<5 years) + IS + NPA), stool (max 7 samples). All respiratory samples tested on smear + MGIT and partly on GX, stool GX	respiratory samples: fluorescent smear microscopy and Xpert MTB/Rif on concentrated sample, MGIT culture if collected by study team. Smear and culture if collected by hospital staff. Stool samples: Xpert and culture (the latter only until half-way the study)	379	n=170: 73 with bacteriologically confirmed TB, 69 with unconfirmed TB, 28 with unlikely TB	n=73: 71 culture-or Xpert positive on respiratory sample, 1 Xpert-positive on stool sample and 1 culture-positive on stool sample	42.94%
Zar et al (2012)[5]	Children aged <15 years with presumptive TB hospitalized in Cape Town, South Africa, because of severe pneumonia, need for oxygen/intravenous therapy, or social conditions precluding home care	2 NPA (taken at least 4h apart) and 2 IS (taken at least 30 min after NPA, and taken at least 4h apart); n=396 with 2 paired IS and NPA, n=139 with 1 paired IS and NPA	Fluorescent smear microscopy and Xpert MTB/Rif on concentrated sample, MGIT culture	535	n=283: 87 with definite TB, 194 with possible TB	n=98: 87 culture-positive and 11 Xpert NPA/IS positive and culture-negative (of whom 9 were treated)	33.92%
Zar et al (2019)[6]	Children aged <15 years, hospitalized for suspected TB in Cape Town, South Africa	2 NPA (taken at least 4h apart) and 2 IS (taken at least 30 min after NPA, and taken at least 4h apart); n=130 with 2 paired IS and NPA, n=65 with 1 paired IS and NPA	Fluorescent smear microscopy and Xpert Ultra (for 2 NPA and 1 IS) on concentrated sample, MGIT culture	195	n=144: 40 with confirmed TB, 104 with unconfirmed TB (not sure though if all were treated)	n=48: 40 culture-positive, between 5 and 9 Xpert Ultra NPA/IS positive*, culture-negative	31.25 - 34.03%*

* The exact number of Xpert-positive, culture-negative cases does not become clear from the paper: there were between 5 (IS and NPA results completely overlap) and 9 (no overlap between IS and NPA results) of such cases.

For peer review only - http://bmjopen.bmj.com/site/about/guidelines.xhtml

The summary estimate from a random effects meta-analysis was 38.0% (95% prediction interval [PI]: 33.1 - 43.1%).

For the above studies, most children were under 5 years of age. The fraction of children with TB in whom it is possible to obtain bacteriological confirmation is thought to be higher for the 5-14 years age group, but we did not find any suitable data to directly inform this. For the 5-14 year old age group, we therefore divided the proportion of children aged 5-14 reported from South African enhanced surveillance data in du Preez et al.[10] by the spontaneous sputum fraction for this age group from above. This assumes that the fraction of children bacteriologically confirmed in routine practice is the product of the fraction who can spontaneously expectorate sputum, and the fraction who would be bacteriologically confirmed with enhanced sample collection and multiple testing (F_{bc}). This yielded an estimate of 58.0% (50.5 - 65.8%) for F_{bc} in this age group.

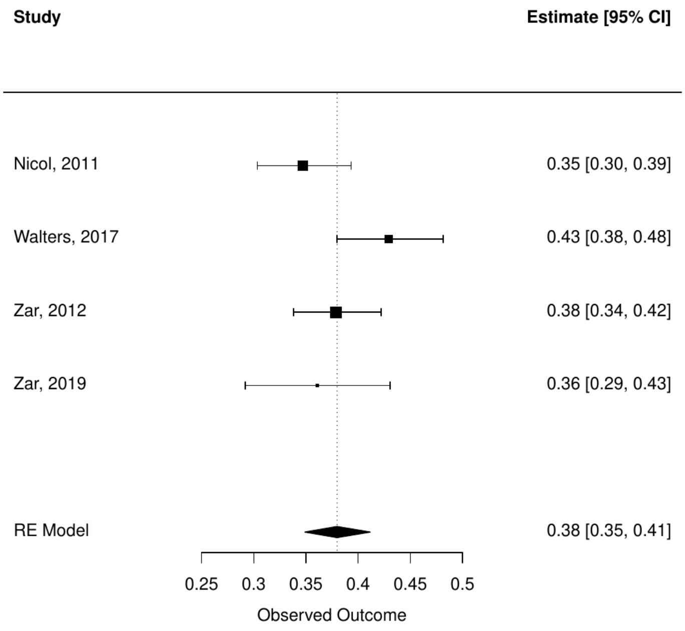

Figure A3. Meta-analytic results for the fraction of children with bacteriologically positive TB under ideal circumstances.

only

Prevalence of true TB in presumptive

For this parameter, from our systematic review (Appendix 1), we selected studies that reported the number of children with presumptive TB and the number of children diagnosed with TB (by method) during the study period.

We restricted to studies that reported using case definitions based on one of the Graham consensus definitions,[11, 12] or the NIH definition, including confirmed/probable TB as TB or the number starting treatment if this was stated (see Table A3). Where age categories reported were not exactly 0-4 years, 5-14 years or 0-14 years, we approximated the age category reported by the studies by its closest match, aggregating over counts if necessary. We performed a random-effects meta-analysis for each age grouping (see Figure A4), finding a summary estimate of 45% (95% prediction interval [PI]: 7.7 - 89%). There was high heterogeneity and wide prediction intervals, with no clear difference between the 0-4 year and 5-14 year age group. We therefore based our parametrization on the pooled 0-14 year analysis, using the midpoint and prediction interval to inform a beta distribution.

Table A3 Studies reporting TB prevalence among presumptive TB patients using NIH or Graham case-definitions.

Author	Year	Design	Setting	clinical diagnosis	specimens and tests	age group	included	diagnosed with TB	type of diagnosis
Bacha[13]	2017	Retrospective descriptive	children evaluated for presumptive TB and/or referred for TB treatment in 1 regional referral hospital in Southern Highlands Zone of Tanzania serving a child population of 3.2 mln children	as per internationally proposed criteria (see Graham et al. 2015)	SSM and Xpert on sputum or IS if unable to expectorate. Culture only if there was a 2nd sample (89.4% of children)	0-14 years	455	120	21 confirmed, 99 probable, 37 possible TB
Elhassan[14]	2016	Cross-sectional	Children with presumptive TB presenting to 5 TB centers in Khartoum state, Sudan	Confirmed TB: cough>2wks AND culture+, Probable: cough>2wk AND CXR abnormal AND HH contact; Possible: Cough>2wk AND HH contact AND TST+	SSM (ZN and auramine fluorescence), IS6110 PCR and LJ culture on sputum (if 7+y) or NGA (if<7y)	0-18y (0-15y as per Methods but 0-18y per Tables & Figures)	197	125	32 confirmed, 56 probable, 37 possible
								32	LJ culture confirmed
						<=6y	86	47	3 confirmed, 29 probable, 15 possible
								3	LJ culture confirmed
						7-12 y	63	40	10 confirmed, 17 probable, 13 possible
								10	LJ culture confirmed
						13-18y	48	38	19 confirmed, 10 probable, 9 possible
								19	LJ culture confirmed
Giang[15]	2015	Cross-sectional	HIV negative children presenting with presumptive TB at a sub-national TB referral hospital in Ho Chi Minh City, Vietnam	at least 1 symptom suggestive of TB plus a positive culture or smear, or plus CXR suggesting TB, positive response to TB therapy, documented close contact with TB patient, or positive TST	concentrated SSM and Xpert, MGIT culture on an average of 2 samples (NS)	0-14 years	150	131	38 confirmed, 60 probable, 33 possible
							150	38	culture or smear-positive (confirmed) PTB
							150	46	Xpert-positive (among confirmed, probable and possible cases only)
							150	39	culture-positive (among confirmed, probable and possible cases only)
Hanrahan [3]	2019	cross-sectional	children with presumptive TB incl. symptomatic child HH contacts of adult TB patients regardless of symptom duration presenting at a high-volume, primary health-care clinic which provides outpatient care for a densely populated urban and peri-urban	Per Graham, 2015. Confirmed TB: microbiologically positive by SSM, culture or Xpert on any sample. Unconfirmed TB: no microbiological confirmation but >=2 of CXR suggesting TB, positive	concentrated FM, Xpert, and MGIT culture on 1 spontaneous sputum sample or 1 NPA+1 IS if unable to produce sputum; 1 stool	60 days to ≤10 years	119	105	4 confirmed, 101 unconfirmed TB
								4	confirmed TB

For peer review only

Author	Year	Design	Setting	clinical diagnosis	specimens and tests	age group	included	diagnosed with TB	type of diagnosis
			impoverished community of about 200,000–300,000 (18% children <10y)	response to TB treatment, TB contact history, or TST+.					
Moussa[16]	2016	Cross-sectional	children with clinical signs of PTB presenting at 1 tertiary care hospital in Cairo, Egypt	at least 1 symptom suggestive of TB plus "microbiological confirmation", or plus CXR suggesting TB, positive response to TB therapy, documented close contact with TB patient, or immunological evidence of MTB infection	SSM, LJ culture on 2 (induced) sputum samples, Xpert MTB/Rif on 2 stool samples	1-15 years	115	107	36 confirmed, 61 probable, 10 possible
							115	36	confirmed PTB
						1-5 years	41	38	10 confirmed, 25 probable, 3 possible
						6-15 years	74	69	26 confirmed, 36 probable, 7 possible
						1-5 years	41	10	confirmed PTB
						6-15 years	74	26	confirmed PTB
Myo[17]	2018	Cross-sectional	Children with suspected PTB presenting at tertiary care pediatric hospital in Mandalay, Myanmar	revised NIH classification: culture or Xpert positive, or at least 2 of symptoms/ signs suggesting TB, CXR consistent with TB, TB exposure or immunological evidence of MTB, or a positive response to TB treatment	concentrated SSM, direct Xpert MTB/Rif and LJ culture on 1 GLA	1 month-12 years	231	121	38 confirmed, 83 unconfirmed
							231	38	culture- or Xpert-positive (confirmed) PTB
Nicol[18]	2013	Cross-sectional	Children presenting with presumptive TB at 1 primary healthcare clinic and 1 tertiary care hospital in Cape Town, South Africa	culture-positive or any other started on TB treatment, or not started on TB treatment but with persistent TB suggestive symptoms and signs at 3-month follow-up	concentrated Xpert and MGIT on 2 IS and Xpert testing of 2 aliquots from 1 stool	0-14 years	115	65	17 definite, 48 possible
							115	17	culture-positive (definite) PTB
Nicol[19]	2019	Cross-sectional	Children presenting with presumptive TB at 1 tertiary care hospital in Cape Town, South Africa	culture-positive or any other started on TB treatment, or not started on TB treatment but with persistent TB suggestive symptoms and signs at 3-month follow-up	Xpert and MGIT on 2 IS (2 oral swabs with quantitative PCR, not incl. in diagnosis)	0-14 years	165	121	40 confirmed, 81 unconfirmed
							165	40	culture-positive (confirmed) PTB
Reither[20]	2014	cross-sectional	Children presenting with presumptive TB at 2 research sites in Tanzania and 1 hospital in Kampala, Uganda	symptoms suggestive of TB and AFB+ smear or abnormal CXR suggestive for TB, or CXR not clearly suggesting	concentrated SSM, Xpert, MGIT and LJ culture on sputum/IS (1-5 samples per child)	2 months-15 years	451	147	37 confirmed, 48 highly probable, 62 probable PTB
							451	37	culture-positive (confirmed) PTB
						2 months-5 years	211	74	16 confirmed, 26 highly probable, 32 probable PTB

For peer review only

Author	Year	Design	Setting	clinical diagnosis	specimens and tests	age group	included	diagnosed with TB	type of diagnosis
				TB but no alternative Dx and complete resolution of symptoms/signs on TB treatment			211	16	culture-positive (confirmed) PTB
						6-10 years	133	39	10 confirmed, 13 highly probable, 16 probable PTB
							133	10	culture-positive (confirmed) PTB
						11-15 years	106	34	11 confirmed, 9 highly probable, 14 probable PTB
							106	11	culture-positive (confirmed) PTB
Sabi[21]	2016	cross-sectional	Children presenting with presumptive TB in 1 zonal hospital in NW Tanzania serving a population of 13 mln. 91% of children admitted to hospital	using 4 different published clinical score charts incl. TST and CXR resutls; but for analysis using Graham et al 2012	FM, Xpert, LJ culture on IS	2 months-12 years	192	40	10 confirmed, 10 probable, 20 possible PTB
							192	10	culture positive (confirmed) PTB
Sorsa[22]	2020	Retrospective document review (historical cross-sectional before- after study)	Children presenting with presumptive TB at Asella Teaching and Referral hospital serving a population of approx. 4 mln in South-Central Ethiopia; Jan 2014-Dec 2017 with Xpert as intervention installed Jan 2016	Confirmed: >=1 TB symptom (cough>=2 wk, contact with TB patient, fever, weight loss, failure to gain weight) and microbiologically confirmed by SSM/Xpert; Probable: >=2 of TB contact history, clinical feature suggesting TB, TST+, CXR abnormal suggesting TB	not specified, but likely direct SSM or Xpert on a sputum sample, not clear if NGA was also done.	<15 y	775	453	142 confirmed, 311 probable
								142	confirmed (SSM/Xpert)
							404 ('before'-period)	254	54 confirmed, 200 probable
								54	confirmed (SSM)
							371 ('after'-period)	199	88 confirmed, 111 probable
								88	confirmed (Xpert)
Walters[9]	2018	prospective cohort	Children (12.5% HIV+) with suspected PTB at two public referral hospitals offering general and specialized pediatric care (Rahima Moosa M&C hospital Johannesburg and Desmond Tutu TB center serving 2 hospitals in Cape Town)	per Graham, 2015	SSM, Xpert and MGIT culture on 1-2 respiratory specimens (1 spontaneous sputum or IS, + 1 GA in subset of children aged <5)	no range provided; median 15.5 month, IQR, 10.9–24.3 months	148	42	treated for TB
								63	3 confirmed, 60 unconfirmed
								3	confirmed TB (culture or Xpert+)
								2	Culture+
Walters , van der Zalm[7]	2017	prospective	Children with suspected intrathoracic TB presenting to Tygerberg Hospital and Karl Bremer Hospital in Cape Town, South Africa, Apr 2012-Aug 2015	per Graham, 2015	2x(Sputum/GA + IS + NPA), stool (max 7 samples). All respiratory samples tested on FM + MGIT and partly on GX, stool GX	<13 y; med: 15.9 months IQR 9.2-29.3	379	258	73 confirmed, 185 unconfirmed TB
								73	confirmed TB (71 detected on culture/Xpert non-stool samples, 1 on stool culture, 1 on stool Xpert)
								71	confirmed TB (on non-stool samples)
								170	TB treatment initiated (reference standard)

Author	Year	Design	Setting	clinical diagnosis	specimens and tests	age group	included	diagnosed with TB	type of diagnosis
Zar[23]	2013	prospective	Children presenting with suspected pulmonary TB at 1 primary care clinic in Khayelitsha, Cape Town, South Africa, Aug 2010-Jul 2012	definite TB: culture+; Possible TB: receiving TB treatment + all whose symptoms/ signs at FU did not resolve if not receiving TB treatment	concentrated FM, concentrated Xpert, MGIT culture on IS+NPA: 80% 2 paired IS+NPA; 20% 1 paired IS+NPA	<15 y; median 38.3 m (IQR:21.2-56.5)	384	197	30 definite, 167 possible TB
								30	definite TB
								180	started on TB treatment

For peer review only

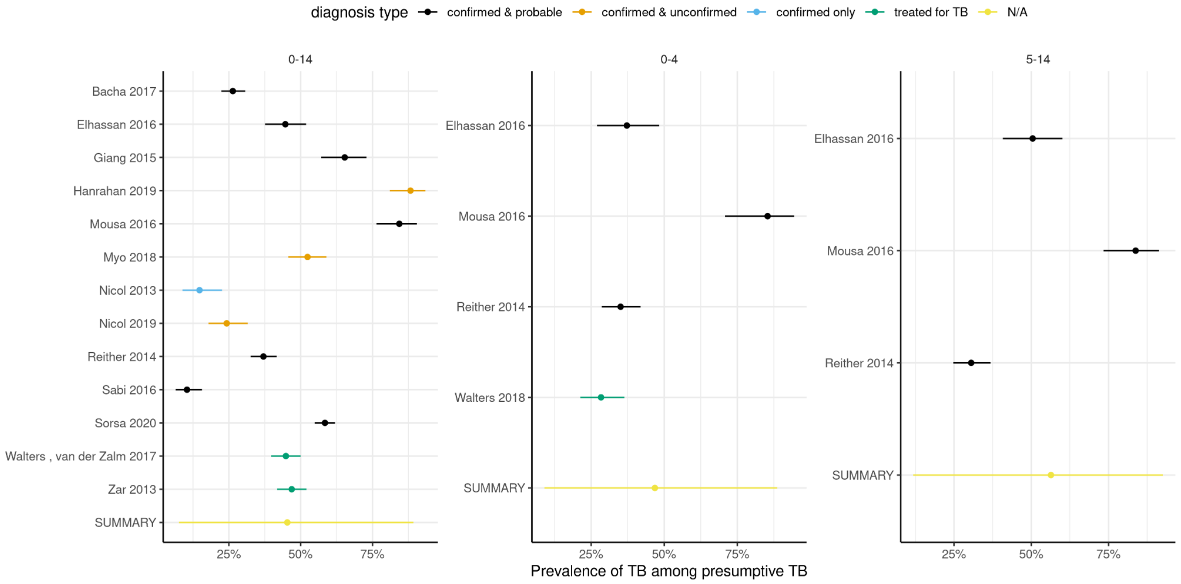

Figure A4 Forest plots for age groups 0-14 years, 0-4 years and 5-14 years of the prevalence of TB among presumptive TB

Accuracy of clinical assessment in bacteriologically negative TB

Sensitivity and specificity of clinical diagnosis

Assessing the diagnostic accuracy of clinical diagnosis for TB in children under routine care is challenging given the absence of a gold standard. Pearce et al.[24] systematically reviewed the accuracy of score-based approaches to diagnosing TB in children, and found one study (van Beekhuizen[25]) which can be interpreted as giving a sensitivity assessment of 62%, and specificity of 95%. The more recent cohort study by Marais et al[26] suggested a sensitivity of 62.6% and specificity of 89.8% among 428 children aged ≤13 years investigated for TB in South Africa. Restricting to children under 3 or those living with HIV, sensitivity was lower: sensitivity 51.8% (specificity 92.5%) for HIV-negative children under 3 years of age; sensitivity of 56.2% (specificity 61.8%) for children living with HIV.

The study of Beneri et al.[27] compared two case definitions within a trial context. Excluding bacteriologically confirmed TB, and counting NIH-unlikely TB as negative gives the cross-tabulation in Table A4.

Table A4 Aggregated data from Beneri et al.[27]

classification for bacteriologically negative children		National Institute of Health (NIH)	
		TB+	TB-
P1041	TB+	93	1
	TB-	44	7

For peer review only

Evaluating the stricter trial (P1041) against the NIH case definition yields a sensitivity of 67.9% and a specificity of 87.5%.

A recent paper by Gunasekera et al.[28] developed an optimized scoring approach to TB diagnosis in children in South Africa, and reported a sensitivity of 71.5% when restricting the tool to inputs only from clinical evaluation (i.e. excluding Xpert MTB/Rif and chest X-ray).

We also considered the WHO estimated case-detection ratio for TB in each country and age group (typically 40-50% in relevant contexts). This approach is problematic because these estimates have large uncertainty, and also because CDR factors in children who did not present for care and who were diagnosed but not reported. It therefore is likely a lower bound for the sensitivity of TB detection algorithms in each country.

Given these data, and the likelihood that trials and optimized diagnostic scores may overestimate accuracy, we opted to use the estimates from Marais et al.[26] We used the estimate for HIV-negative children under 3 for all children under 5, and the overall estimate for children aged 5-14 years (see Table A5).

Table A5 Parameters used for accuracy of clinical diagnosis

NAME	DISTRIBUTION	DESCRIPTION	SOURCE	Mean (IQR)
spec.clinu3	B(83.25,6.75)	specificity of clinical dx < 5 years	Marais 2006[26]	0.928 (0.908 - 0.945)
sens.clinu3	B(46.62,43.38)	sensitivity of clinical dx < 5 years	Marais 2006[26]	0.518 (0.482 - 0.554)
spec.clin	B(80.82,9.18)	specificity of clinical dx 5-14 years	Marais 2006[26]	0.901 (0.878 - 0.921)
sens.clin	B(56.34,33.66)	sensitivity of clinical dx 5-14 years	Marais 2006[26]	0.627 (0.592 - 0.661)

Accuracy of bacteriological tests

Sensitivity and specificity of Xpert and smear microscopy on sputum, and of Xpert on stool

For diagnostic tests other than stool, we used the accuracies quoted by the systematic review of Detjen et al.,[29] see Table A6.

Table A6 Diagnostic accuracies reported by Detjen et al.[29]

diagnostic	sample	reference	Sensitivity (95% CI & PI)	specificity (95% CI & PI)
Xpert	Exp/Induced Sputum	culture	62% (51–73; 30–87)	98% (97–99; 90–100)
Xpert	gastric lavage	culture	66% (51–81; 33–91)	98% (96–99; 91–100)
Xpert	Exp/Induced Sputum	clinical for C-ve	2% (1–3; 0–6)	100% (99–100; 99–100)
microscopy	Exp/Induced	culture	26% (14–39; 4–69)	100% (99–100; 94–100)

For peer review only - http://bmjopen.bmj.com/site/about/guidelines.xhtml

	Sputum			
microscopy	gastric lavage	culture	22% (12–35; 6–51)	99% (97–100; 93–100)

For the accuracy of stool for diagnosing TB in children, we used the two systematic reviews and meta-analyses: MacLean et al.[30] and Mesman et al.[31] (Table A7).

Table A7 Parameters found by review on diagnostic test accuracy

NAME	DISTRIBUTION	DESCRIPTION	SOURCE	Mean (IQR)
sens.stool	B(20.39,15.3)	sensitivity of Xpert on stool in bac+ children	Mesman 2019[31]	*0.571 (0.515 - 0.627)*
spec.stool	B(326.97,6.67)	specificity of stool in bac+ children	Mesman 2019[31]	*0.981 (0.975 - 0.986)*
sens.xpert	B(45.75,28.04)	sensitivity for C+ of Xpert on sputum	Detjen 2015[29]	*0.621 (0.582 - 0.659)*
spec.xpert	B(736.91,15.03)	specificity for C+ of Xpert on sputum	Detjen 2015[29]	*0.980 (0.977 - 0.984)*
sens.sm	B(12.03,34.26)	sensitivity for C+ of SM on sputum	Detjen 2015[29]	*0.257 (0.215 - 0.302)*
spec.sm	B(759.66,3.81)	specificity for C+ of SM on sputum	Detjen 2015[29]	*0.995 (0.994 - 0.997)*

Level of initial care-seeking

We found no data specific to paediatric TB to inform the proportion of children initially seeking care at primary healthcare level. We ultimately relied on estimates of initial care seeking for Ethiopia and Indonesia made in two TB patient pathway analysis (PPA) papers, namely Fekadu et al.[32] and Surya et al.[33] We included care sought in the private and public sectors, mapping primary care level to the levels L0 and L1 defined in the papers. The former suggested 89.6% of children initially seek care at primary level in Ethiopia; the latter that 92.8% of children initially seek care at primary level in Indonesia. In the absence of data to inform uncertainty, and given the quality of this evidence for our question, we assumed the 95% uncertainty interval was at +/- 10% points around the central estimate.

For peer review only - http://bmjopen.bmj.com/site/about/guidelines.xhtml

Summary of model parameters from review and distributions

Table A8 Parameters informed by analyses above

NAME	DISTRIBUTION	DESCRIPTION	SOURCE	Mean (IQR)
spont.sput.u5	B(21.572,877.28)	spontaneous sputum possible (0-4)	see methods	0.024 (0.020 - 0.027)
spont.sput.o5	B(2.59,4.07)	spontaneous sputum possible (5-14)	see methods	0.377 (0.254 - 0.512)
Fbc.u5	B(137.19,223.84)	fraction of children bacteriologically confirmable <5	see methods	0.380 (0.363 - 0.397)
Fbc.o5	B(97.76,45.12)	fraction of children bacteriologically confirmable 5-14	see methods	0.684 (0.659 - 0.711)
p_truetb	B(3.10,1.85)	prevalence of true TB in presumptive	see methods	0.625 (0.484 - 0.783)
spec.clinu3	B(83.25,6.75)	specificity of clinical dx	Marais 2006[26]	0.928 (0.908 - 0.945)
sens.clinu3	B(46.62,43.38)	sensitivity of clinical dx	Marais 2006[26]	0.518 (0.482 - 0.554)
spec.clin	B(80.82,9.18)	specificity of clinical dx	Marais 2006[26]	0.901 (0.878 - 0.921)
sens.clin	B(56.34,33.66)	sensitivity of clinical dx	Marais 2006[26]	0.627 (0.592 - 0.661)
phc0_e	B(31.18,3.62)	proportion of first care-seeking at PHC for Ethiopia	Fekadu 2017[32]	0.896 (0.777 - 0.973)
phc0_i	B(22.89,1.78)	proportion of first care-seeking at PHC for Indonesia	Surya 2017[33]	0.928 (0.801 - 0.992)

Summary of other parameters

Parameters in common between countries from previous work

Most the of the CFR parameters are based on Jenkins et al.[1]

Table A9 Parameters informed by the literature review

NAME	DISTRIBUTION	DESCRIPTION	SOURCE	Mean (IQR)
cfrontxY	LN(-3.96,0.64)	CFR children <5 on TB treatment	Jenkins et al 2017[1]	*0.019 (0.012 - 0.029)*
cfrontxO	LN(-4.82,0.48)	CFR children 5-14 on TB treatment	Jenkins et al 2017[1]	*0.008 (0.006 - 0.011)*
cfrnotxY	LN(-0.83,0.08)	CFR children <5 without TB treatment	Jenkins et al 2017[1]	*0.436 (0.413 - 0.460)*
cfrnotxO	LN(-1.90,0.12)	CFR children 5-14 without TB treatment	Jenkins et al 2017[1]	*0.149 (0.137 - 0.162)*

For peer review only - http://bmjopen.bmj.com/site/about/guidelines.xhtml

Parameters specific to Ethiopia

r2 in particular was adjusted upwards after consultation to reflect a low confidence with child TB diagnosis and management at primary level.

Table A10 Parameters specific to Ethiopia not included above

NAME	DISTRIBUTION	DESCRIPTION	SOURCE	Mean (IQR)
fracu5	B(7.504,12.47)	fraction of presumptive TB under 5	based on fraction of WHO TB < 5	*0.371 (0.300 - 0.447)*
r1	B(1,15)	referral PHC -> H after clinical re-assessment following bac-	Expert opinion	*0.045 (0.019 - 0.088)*
r2	B(8,2)	referral PHC -> H after initial clinical assessment w/o bac	Expert opinion	*0.800 (0.728 - 0.899)*
g.phc	B(1,15)	clincial re-assessment, PHC	Expert opinion	*0.045 (0.019 - 0.088)*

Parameters specific to Indonesia

r1 in particular was adjusted upwards after consultation to reflect a low confidence in bacteriologic testing for child TB.

Table A11 Parameters specific to Indonesia not included above

NAME	DISTRIBUTION	DESCRIPTION	SOURCE	Mean (IQR)
fracu5	B(69.37,65.49)	fraction of presumptive TB under 5*	based on fraction of WHO TB < 5	*0.514 (0.485 - 0.543)*
r1	B(2,8)	referral PHC -> H after clinical re-assessment following bac-	Expert opinion	*0.200 (0.107 - 0.272)*
r2	B(5,5)	referral PHC -> H after initial clinical assessment w/o bac	Expert opinion	*0.500(0.391 - 0.607)*
g.phc	B(1,15)	clincial re-assessment, PHC	Expert opinion	*0.045 (0.019 - 0.088)*

* based on the proportion of TB cases under 5 among all child TB cases (<15y)

Other parameters based on assumption

Note: many of these parameters could potentially be made country-specific, but currently are not.

Table A12 Parameters without direct evidence based on assumptions

NAME	DISTRIBUTION	DESCRIPTION	SOURCE	Mean (IQR)
c.phc	B(95,5)	1-PTLTFU bac+, PHC	assumption	*0.953 (0.937 - 0.966)*
c.h	B(95,5)	1-PTLTFU bac+, H	assumption	*0.953 (0.937 - 0.966)*
e.phc	B(1,15)	clinical re-assessment after bac-, PHC	assumption	*0.045 (0.019 - 0.088)*
e.h	B(1,15)	clinical re-assessment after bac-, H	assumption	*0.045 (0.019 - 0.088)*
g.h	B(1,15)	clincial re-assessment, H	assumption	*0.045 (0.019 - 0.088)*
r3	B(5,5)	referral PHC -> H after clinical re-assessment w/o bac	assumption	*0.500(0.391 - 0.607)*

For peer review only - http://bmjopen.bmj.com/site/about/guidelines.xhtml

Description approach to expert opinion

For parameters for which no data was found in published literature, our best estimate based on the practical experience from the authors working in Ethiopia (AB and MG) and Indonesia (NK and RT) was included. NK and RT are experienced pediatricians working in large tertiary care settings in Indonesia. Both are active in the TB section of the Indonesian Association of Pediatricians, of which currently is the chairperson. RT has ample research experience in the field of diagnosing childhood TB in primary, secondary and tertiary care settings. AB and MG are both working for the local KCNV office. AB is a pediatrician with experience in the clinical, research and programmatic settings. He is a member of the Ethiopian Pediatric Association. MG is an senior M&E advisor with up-to-date practical experience in rural and urban sites involved in childhood TB projects run by KNCV.

Data was needed to inform parameters on the proportion of children with bacteriologically confirmed TB started on treatment (*c.phc* and *c.h*), the proportion clinically reassessed after initial bacteriological exclusion of TB (*e.phc* and *e.h*), the proportion of children clinically reassessed after short broad-course of antibiotics (*g.phc* and *g.h*), and about referrals from the primary to higher (hospital) levels (*r1*, *r2* and *r3*).

A data collection tool was distributed to the experts, in which per parameter, their best guess and the minimum and maximum value they considered reasonable could be filled (Table). Then, several online sessions were organized. The first session served to explain the data collection tool. The second session served to discuss the completed tool and solve differences in opinion between the experts where needed. A third session was organized to present the model output using the experts' best estimates. In this session, the set of parameters was further adapted to come to model outputs that seemed reasonable for the country.

For peer review only - http://bmjopen.bmj.com/site/about/guidelines.xhtml

References

1. Jenkins, H.E., et al., *Mortality in children diagnosed with tuberculosis: a systematic review and meta-analysis.* The Lancet. Infectious diseases, 2017. **17**(3): p. 285-295.
2. Bates, M., et al., *Assessment of the Xpert MTB/RIF assay for diagnosis of tuberculosis with gastric lavage aspirates in children in sub-Saharan Africa: a prospective descriptive study.* The Lancet Infectious Diseases, 2013. **13**(1): p. 36-42.
3. Hanrahan, C.F., et al., *Diagnostic strategies for childhood tuberculosis in the context of primary care in a high burden setting: the value of alternative sampling methods.* Paediatrics and International Child Health, 2018. **39**(2): p. 88-94.
4. Nicol, M.P., et al., *Accuracy of the Xpert MTB/RIF test for the diagnosis of pulmonary tuberculosis in children admitted to hospital in Cape Town, South Africa: a descriptive study.* The Lancet. Infectious diseases, 2011. **11**(11): p. 819-824.
5. Zar, H.J., et al., *Rapid molecular diagnosis of pulmonary tuberculosis in children using nasopharyngeal specimens.* Clinical infectious diseases : an official publication of the Infectious Diseases Society of America, 2012. **55**(8): p. 1088-1095.
6. Zar, H.J., et al., *Tuberculosis Diagnosis in Children Using Xpert Ultra on Different Respiratory Specimens.* American journal of respiratory and critical care medicine, 2019. **200**(12): p. 1531-1538.
7. Walters, E., et al., *Xpert MTB/RIF on Stool Is Useful for the Rapid Diagnosis of Tuberculosis in Young Children With Severe Pulmonary Disease.* The Pediatric infectious disease journal, 2017. **36**(9): p. 837-843.
8. Ssengooba, W., et al., *Accuracy of Xpert Ultra in Diagnosis of Pulmonary Tuberculosis among Children in Uganda: a Substudy from the SHINE Trial.* Journal of clinical microbiology, 2020. **58**(9): p. e00410-20.
9. Walters, E., et al., *Molecular Detection of Mycobacterium tuberculosis from Stools in Young Children by Use of a Novel Centrifugation-Free Processing Method.* Journal of clinical microbiology, 2018. **56**(9): p. e00781-18.
10. du Preez, K., et al., *The Impact of the Evolving Human Immunodeficiency Virus Response on the Epidemiology of Tuberculosis in South African Children and Adolescents.* Clinical Infectious Diseases, 2021.
11. Graham, S.M., et al., *Evaluation of tuberculosis diagnostics in children: 1. Proposed clinical case definitions for classification of intrathoracic tuberculosis disease. Consensus from an expert panel.* The Journal of infectious diseases, 2012. **205 Suppl 2**(Suppl 2): p. S199-S208.
12. Graham, S.M., et al., *Clinical Case Definitions for Classification of Intrathoracic Tuberculosis in Children: An Update.* Clinical infectious diseases : an official publication of the Infectious Diseases Society of America, 2015. **61Suppl 3**(Suppl 3): p. S179-S187.
13. Bacha, J.M., et al., *Why being an expert - despite xpert -remains crucial for children in high TB burden settings.* BMC infectious diseases, 2017. **17**(1): p. 123-123.
14. Elhassan, M.M., et al., *Challenges in diagnosing tuberculosis in children: a comparative study from Sudan.* International Journal of Infectious Diseases, 2016. **43**: p. 25-29.
15. Giang, D.C., et al., *Prospective evaluation of GeneXpert for the diagnosis of HIV- negative pediatric TB cases.* BMC infectious diseases, 2015. **15**: p. 70-70.
16. Moussa, H.S., F.S. Bayoumi, and A.M.A. Mohamed, *Gene Xpert for direct detection of Mycobacterium tuberculosis in stool specimens from children with presumptive pulmonary tuberculosis.* Annals of Clinical & Laboratory Science, 2016. **46**(2): p. 198-203.
17. Myo, K., et al., *Evaluation of Xpert® MTB/RIF assay as a diagnostic test for pulmonary tuberculosis in children in Myanmar.* The International Journal of Tuberculosis and Lung Disease, 2018. **22**(9): p. 1051-1055.
18. Nicol, M.P., et al., *Xpert MTB/RIF testing of stool samples for the diagnosis of pulmonary tuberculosis in children.* Clinical infectious diseases : an official publication of the Infectious Diseases Society of America, 2013. **57**(3): p. e18-e21.
19. Nicol, M.P., et al., *Microbiological diagnosis of pulmonary tuberculosis in children by oral swab polymerase chain reaction.* Scientific reports, 2019. **9**(1): p. 10789-10789.

For peer review only

For peer review only - http://bmjopen.bmj.com/site/about/guidelines.xhtml

20. Reither, K., et al., *Xpert MTB/RIF assay for diagnosis of pulmonary tuberculosis in children: A prospective, multi-centre evaluation.* Journal of Infection, 2015. **70**(4): p. 392-399.
21. Sabi, I., et al., *Pulmonary TB bacteriologically confirmed by induced sputum among children at Bugando Medical Centre, Tanzania.* The International Journal of Tuberculosis and Lung Disease, 2016. **20**(2): p. 228-234.
22. Sorsa, A., et al., *Use of Xpert Contributes to Accurate Diagnosis, Timely Initiation, and Rational Use of Anti-TB Treatment Among Childhood Tuberculosis Cases in South Central Ethiopia.* Pediatric health, medicine and therapeutics, 2020. **11**: p. 153-160.
23. Zar, H.J., et al., *Rapid diagnosis of pulmonary tuberculosis in African children in a primary care setting by use of Xpert MTB/RIF on respiratory specimens: a prospective study.* The Lancet Global Health, 2013. **1**(2): p. e97-e104.
24. Pearce, E.C., et al., *A systematic review of clinical diagnostic systems used in the diagnosis of tuberculosis in children.* AIDS research and treatment, 2012. **2012**: p. 401896-401896.
25. van Beekhuizen, H.J., *Tuberculosis Score Chart in Children in Aitape, Papua New Guinea.* Tropical Doctor, 1998. **28**(3): p. 155-160.
26. Marais, B.J., et al., *A Refined Symptom-Based Approach to Diagnose Pulmonary Tuberculosis in Children.* Pediatrics, 2006. **118**(5): p. e1350-e1359.
27. Beneri, C.A., et al., *Understanding NIH clinical case definitions for pediatric intrathoracic TB by applying them to a clinical trial.* The international journal of tuberculosis and lung disease : the official journal of the International Union against Tuberculosis and Lung Disease, 2016. **20**(1): p. 93-100.
28. Gunasekera, K.S., et al., *Development of a Treatment-decision Algorithm for Human Immunodeficiency Virus–uninfected Children Evaluated for Pulmonary Tuberculosis.* Clinical Infectious Diseases, 2021.
29. Detjen, A.K., et al., *Xpert MTB/RIF assay for the diagnosis of pulmonary tuberculosis in children: a systematic review and meta-analysis.* The Lancet. Respiratory medicine, 2015. **3**(6): p. 451-461.
30. MacLean, E., et al., *Diagnostic Accuracy of Stool Xpert MTB/RIF for Detection of Pulmonary Tuberculosis in Children: a Systematic Review and Meta-analysis.* Journal of clinical microbiology, 2019. **57**(6): p. e02057-18.
31. Mesman, A.W., et al., *Diagnostic accuracy of molecular detection of Mycobacterium tuberculosis in pediatric stool samples: A systematic review and meta-analysis.* Tuberculosis (Edinburgh, Scotland), 2019. **119**: p. 101878-101878.
32. Fekadu, L., et al., *Increasing Access to Tuberculosis Services in Ethiopia: Findings From a Patient-Pathway Analysis.* The Journal of infectious diseases, 2017. **216**(suppl_7): p. S696-S701.
33. Surya, A., et al., *Quality Tuberculosis Care in Indonesia: Using Patient Pathway Analysis to Optimize Public-Private Collaboration.* The Journal of infectious diseases, 2017. **216**(suppl_7): p. S724-S732.

For peer review only

Xpert on stool to diagnose tuberculosis in children is cost-effective in Ethiopia and Indonesia: a model-based cost-effectiveness analysis

Appendix 2b: Overview of cost parameters

Contents

General costing assumptions 3
Identifying resources used 3
Measuring resource utilization 3
Valuation of resources 4
Clinical assessment 4
TB assessment at health centre 4
TB assessment at hospital 5
TST 6
CXR 6
Sample collection 6
Bacteriological assessment 6
Sputum smear microscopy (SSM) examination 6
GeneXpert test 6
Clinical (re-) assessment 7
TB reassessment at health centre 7
TB reassessment at hospital 7
TB treatment 7
TB treatment medications 7
TB treatment follow-up at health centre 7
TB treatment follow-up at hospital 7
Laboratory monitoring 8
Comparison with other cost estimates 8
Costs tables 9
Table A1 Costs for Ethiopia & Indonesia 9
Table A2 Comparison of estimated unit costs for diagnostic visit, sputum collection, smear microscopy examination and XpertMTB/RIF test with recently published Better estimates of the costs of TB control (Value TB) project[13]. 10
References 11

For peer review only - http://bmjopen.bmj.com/site/about/guidelines.xhtml

For peer review only

For peer review only - http://bmjopen.bmj.com/site/about/guidelines.xhtml

General costing assumptions

Identifying resources used

A careful review of the diagnostic and treatment algorithm for childhood TB for Ethiopia (ETH)[1] and Indonesia (IDN)[2] was performed to identify resource use and costs associated with the diagnosis and treatment for childhood TB in the current standard of care (SOC) and under the intervention of using stool with Xpert for the diagnosis of TB **(Figures 1-3)**. The main activities in both the SOC and the intervention included symptom-based screening for TB, clinical evaluation, sample collection, bacteriological examination, radiological examination, empiric antibiotic therapy to exclude other diseases, treatment initiation for diagnosed TB cases, TB treatment follow-up and TB treatment monitoring laboratory tests. The following cost components were identified; health facility visit (for TB screening, rescreening, diagnosis and treatment), sample collection (spontaneous sputum, stool), bacteriological examination (smear microscopy, GeneXpert), and medicines (anti-TB medicines, pyridoxine).

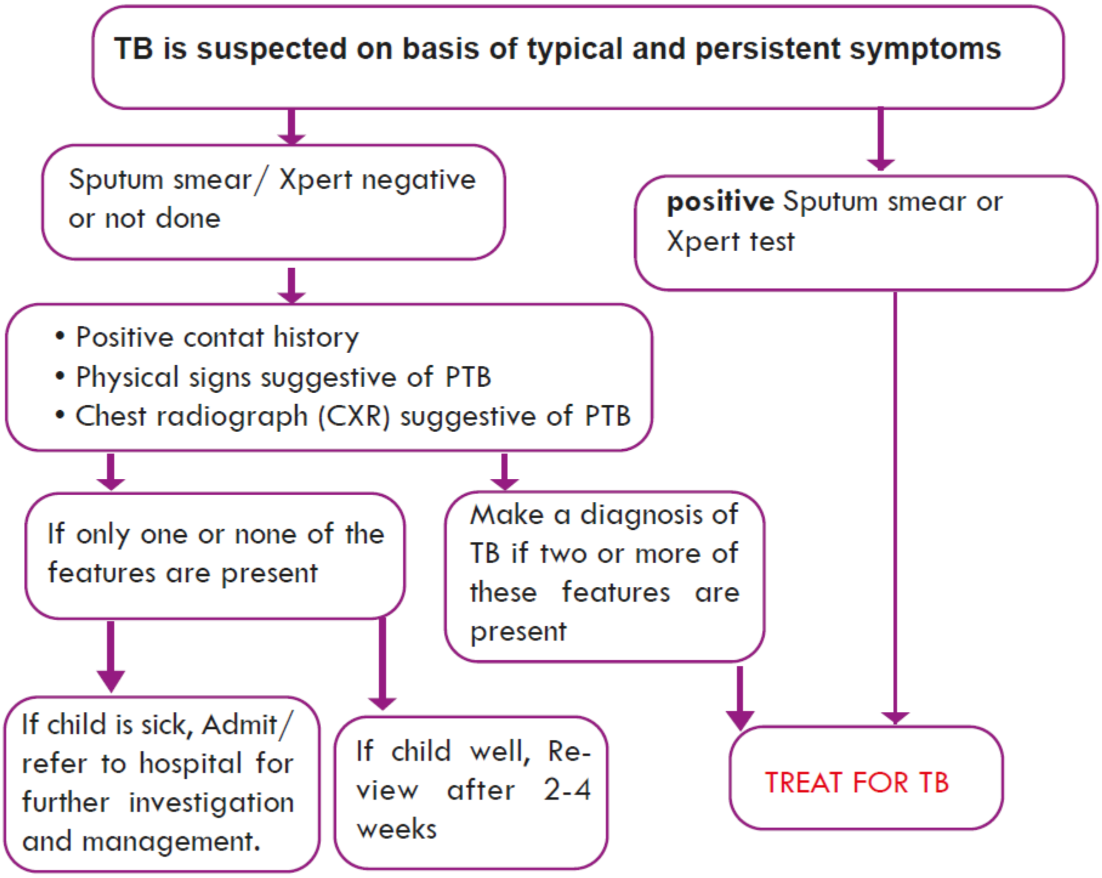

Figure 1. Algorithm for diagnosis of tuberculosis in HIV uninfected children in Ethiopia[1]

Measuring resource utilization

The quantities of each resource type consumed in the diagnostic and treatment algorithm for childhood TB was informed by the national guidelines for the management of childhood TB in each country (**Figures 1-3**). Local TB experts collaborating with KNCV on childhood TB related studies provided country-specific input on resource use.

For peer review only - http://bmjopen.bmj.com/site/about/guidelines.xhtml

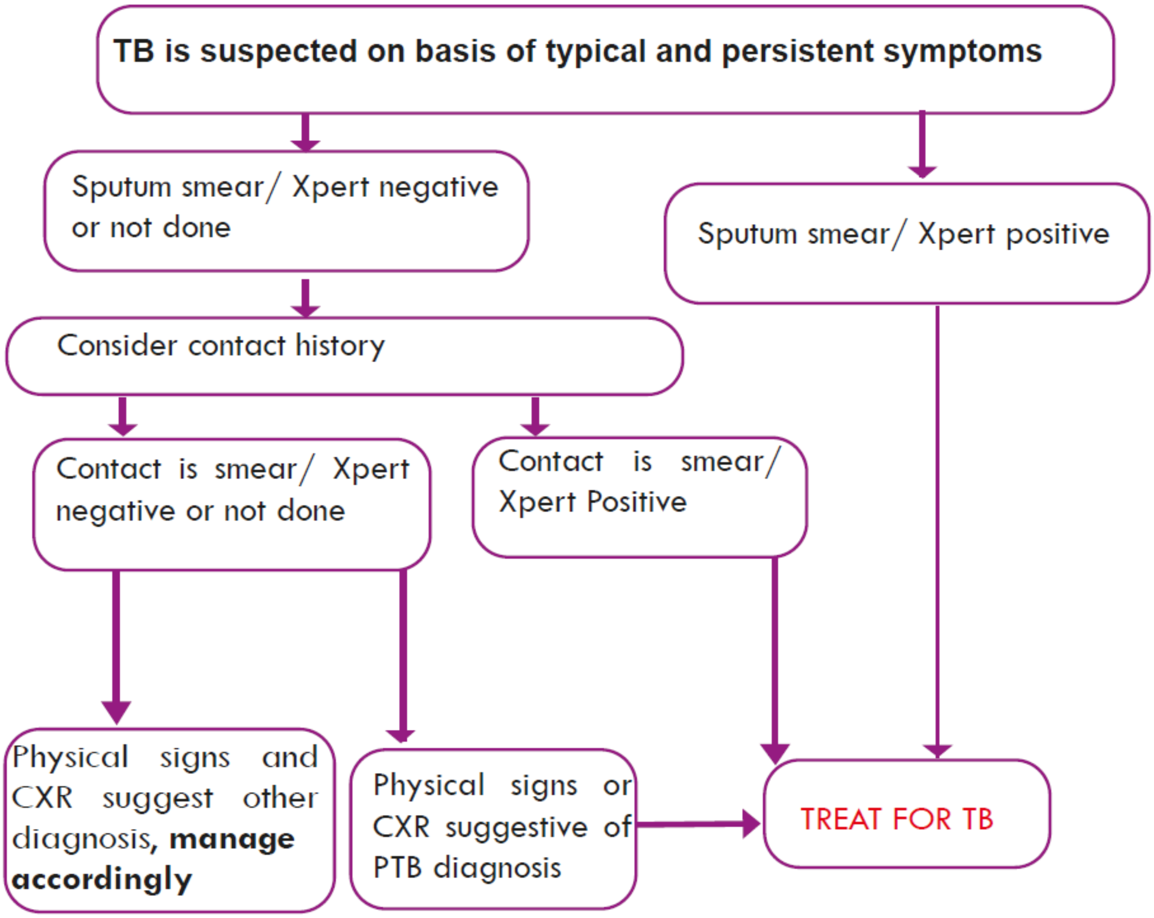

Figure 2. Algorithm for diagnosis of tuberculosis in HIV-infected children in Ethiopia[1]

Valuation of resources

The costs for each resource were estimated by attaching monetary values using relevant unit costs for each country. Summing up these costs (per patient) gives an estimate of the total cost. All costs were estimated from the healthcare provider's perspective and are reported in 2019 USD. Historical costs were adjusted for inflation to 2019 prices using relevant GDP deflators[3] and costs from other countries were transferred to Ethiopia and Indonesia by applying relevant purchasing power parity conversion factors[3]. Costs were assumed to accrue in the present, with no discounting applied. The following costs were estimated.

Clinical assessment

This cost comprises of the cost of clinical assessment to investigate children with presumptive TB and the cost associated with collecting the necessary samples for bacteriological evaluation.

TB assessment at health centre

We assumed the cost for the initial TB assessment at the primary health centre to be equivalent to the country-specific cost of two outpatient visits (range; 1-3 visits) to a health centre with no beds based on WHO-CHOICE estimates[4]. This equates to \$10.22 (95% UI; 4.93-15.50) in ETH and \$43.35 (95% UI; 19.11-67.59) in IDN.

For peer review only - http://bmjopen.bmj.com/site/about/guidelines.xhtml

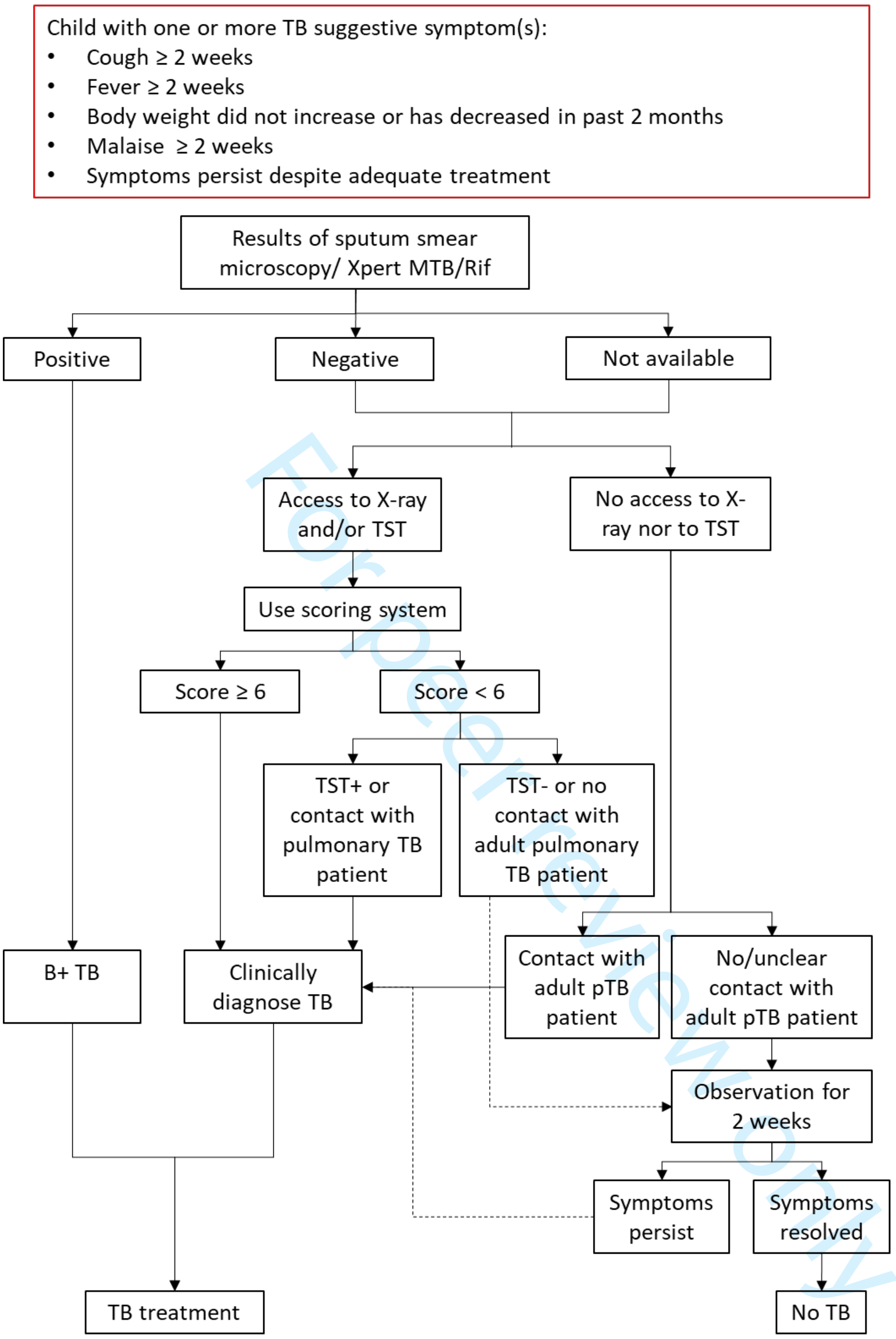

Figure 3. Algorithm for diagnosis of tuberculosis in children in Indonesia[2]

TB assessment at hospital

Similarly, we assumed the cost for the initial TB assessment at the hospital to be equivalent to the country-specific cost of two outpatient visits (range; 1-3 visits) to a primary hospital, defined as a 'hospitals intended primarily for treating simple cases (e.g. "district hospital")' [4]. This results in an estimate of $14.37 (95% UI; 7.79-20.96) in ETH and $61.00 (95% UI; 30.76-91.23) in IDN. The costs of tuberculin skin test (TST) and chest X-ray were separately addressed (see below).

For peer review only - http://bmjopen.bmj.com/site/about/guidelines.xhtml

TST

The Indonesian NTP uses a scoring system for the diagnosis of TB if a bacteriological diagnosis cannot be made (Figure 1). The scoring system uses a combination of tuberculin skin test (TST) results, chest X-ray results (CXR), symptoms and history of contact with TB patients (Table 1). TST and CXR are often not available especially at the primary health centres, hence these costs are currently not modelled.

CXR

The unit cost for CXR for Ethiopia ($8.75) was based on the cost per radiograph reported by the Ethiopian NTP[5]. The unit cost applied for CXR for Indonesia ($11.52) was based on a previous MSH estimate[6]. This cost is applied to a proportion of children assessed at the hospital (80-90%) only since chest X-rays are not available at the primary health centres.

Sample collection

Availability of advanced sample collection procedures is generally limited in both countries, with sputum induction occurring only in big hospitals in Indonesia and nasogastric aspiration only available at big teaching hospitals in Ethiopia. We assumed only self-expectorated sample collection is available and applied the adjusted unit costs for collecting two samples for testing with smear microscopy ($4.64 in ETH and $3.48 in IDN) or one sample for testing with GeneXpert ($2.32 in ETH and $1.74 in IDN) per child based on a study done in adults in South Africa [7]. The unit costs applied for procedures for collecting a single stool sample ($1.67) in the intervention are based on estimates provided by the Paediatric Operational Sustainability Expertise Exchange group (POSEE group) [8]. The POSEE group developed a budgeting tool to assist national TB programs in estimating the costs related to the procurement of devices and consumables needed for sample collection in the paediatric population. These POSEE group cost estimates currently exclude staff, space, training, sample transportation and overheads costs.

Bacteriological assessment

The cost for bacteriological assessment for TB comprises of the costs of testing using either a sputum smear microscopy (SSM) examination or a single GeneXpert test, depending on the availability of the test at each level of care (primary health centre versus hospital) in Ethiopia and Indonesia. Bacteriological testing with the GeneXpert is not widely available in both countries and most testing centres use sputum smear microscopy while some centres refer samples to a GeneXpert testing facility. We therefore assumed sole use of smear microscopy in both countries in the standard of care where two samples are collected for testing in the base case.

Sputum smear microscopy (SSM) examination

The unit cost for SSM for Ethiopia was based on the microscopy cost per test ($1.50) reported by the Ethiopian NTP[5] resulting in an adjusted cost of $1.69. The unit cost for SSM in Indonesia ($3.77) was based on a previous MSH estimate[6].

GeneXpert test

The unit cost for the GeneXpert test was estimated based on country specific data available from the OneHealth Tool[9]. These data include staff times, staff salaries, the Xpert cartridge and consumables. The cost of the GeneXpert equipment was estimated based on the procurement cost of the Xpert MTB/RIF 4-module machine and its annual maintenance cost available from the Global Drug Facility[10]. Costs associated with unused GeneXpert

For peer review only - http://bmjopen.bmj.com/site/about/guidelines.xhtml

equipment capacity were estimated by accounting for the number of tests performed per day in relation to an assumed daily maximum capacity of 16 tests[11]. Overhead costs were estimated as 5% of the total direct costs based on recent studies showing overhead costs contributing 1-10% of total Xpert costs[11-13]. The estimated unit costs for the GeneXpert test are $ 26.04 (95% UI; 18.95-33.13) for ETH and $23.70 (95% UI; 16.59-30.81).

Clinical (re-) assessment

This cost comprised of the cost of clinical re-assessment of children with significant clinical manifestations of TB following exclusion of TB during the initial assessment.

TB reassessment at health centre

We assumed the cost for TB re-assessment at the primary health centre to be equivalent to the country-specific cost of a single outpatient visit to a health centre with no beds[4]. This equated to $5.11 (95% UI; 2.86-7.35) in ETH and $21.68 (95% UI; 11.16-32.19) in IDN.

TB reassessment at hospital

Similarly, we assumed the cost for TB re-assessment at the hospital to be equivalent to the country-specific cost of a single outpatient visit to a primary hospital, defined as a 'hospitals intended primarily for treating simple cases (e.g. "district hospital")' [4]. This resulted in an estimate of $7.19 (95% UI; 4.51-9.87) in ETH and $30.50 (95% UI; 17.84-43.16) in IDN.

TB treatment

Treatment cost for bacteriologically confirmed TB comprises of the cost of anti-tuberculosis drugs including pyridoxine, the costs of follow-up visits (drug pickups or medical review) at the healthcare facilities and costs of laboratory monitoring.

TB treatment medications

The costs of anti-tuberculosis drugs and pyridoxine were estimated using weight band based dosing and applying unit costs available from the Global Drug Facility[14]. We assumed a treatment duration of 6 months. This resulted in the following costs: $11.38, $22.77, $34.15, and $45.54 for children in the weight bands 4-7kg, 8-11kg, 12-15kg and 16-24kg, respectively. The cost of pyridoxine for the duration of TB treatment was estimated to be $2.52[14].

TB treatment follow-up at health centre

We assume the cost for each TB treatment follow-up visit at the primary health centre to be equivalent to the country-specific cost of a single outpatient visit to a health centre with no beds (see above)[4]. This unit cost was multiplied by the number of follow-up visits dictated by the national TB treatment algorithm to estimate the total cost for TB treatment follow-up. Based on input from the local TB experts, we assumed 6 follow-up visits per child on TB treatment in IND and 72 follow-up visits per child on TB treatment in ETH where clinic-based directly observed therapy is used in children. This resulted in the TB treatment follow-up cost at the health centre of $367.76 (95% UI; 167.54-814.45) in ETH and $130.05 (95% UI; 55.51-307.92) in IDN.

TB treatment follow-up at hospital

Similarly, we assumed the cost for each TB treatment follow-up visit at the hospital to be equivalent to the country-specific cost of a single outpatient visit to a primary hospital,

For peer review only - http://bmjopen.bmj.com/site/about/guidelines.xhtml

defined as a ‘hospitals intended primarily for treating simple cases (e.g. “district hospital”)’ [4]. This unit cost was multiplied by the number of follow-up visits to estimate the total cost for TB treatment follow-up. Based on input from the two local TB experts, we assumed 6 follow-up visits per child on TB treatment in IND and 72 follow-up visits per child on TB treatment in ETH where directly observed therapy is used in children. This resulted in the TB treatment follow-up cost of $517.48 (95% UI; 261.52-1033.08) in ETH and $183.00 (95% UI; 86.65-390.58) in IDN.

Laboratory monitoring

Laboratory monitoring is usually not done in children, unless they can spontaneously expectorate a sample. This is only the case for a certain proportion of the oldest age group, which is likely lower than the estimate that we use for the oldest age class (see Table A8 in Appendix 2a). Therefore, we currently do not include the cost of laboratory monitoring in our analysis.

Comparison with other cost estimates

We evaluated the accuracy of our unit cost estimates by comparing them to the recently published cost estimates for Ethiopia by the Better estimates of the costs of TB control (Value TB) project[15]. We compared the unit costs for a diagnostic visit, sputum sample collection, sputum smear microscopy examination, XpertMTB/RIF test and treatment monitoring visit. Although not exactly the same, our unit costs were quite comparable to the Value-TB cost estimates.

For peer review only

For peer review only - http://bmjopen.bmj.com/site/about/guidelines.xhtml

Costs tables

Table A1 Costs for Ethiopia & Indonesia

Cost parameter	Description	Ethiopia	Indonesia	References
c_a_phc<br>c_clin.phc	TB clinical assessment at health centre	10.22 (4.93 - 15.50)	43.35 (19.11 - 67.59)	[4]
c_e_phc<br>c_g.phc	TB clinical reassessment at health centre	5.11 (2.86 - 7.35)	21.68 (11.16 - 32.19)	[4]
c_b.phc.sess<br>c_b.h.sess	Self-expectorated sputum sample	2.32 (1.74 - 2.90)	1.74 (1.30 - 2.17)	[8]
c_b.phc.ss<br>c_b.h.ss	Stool sample	1.67 (1.25 - 2.09)	1.67 (1.25 - 2.09)	[8]
c_b.phc.ssm<br>c_b.h.ssm	Sputum smear microscopy	3.39 (1.94 - 4.83)	7.54 (5.96 - 9.12)	[5, 6]
c_b.phc.xpert<br>c_b.h.xpert	GeneXpert test	26.04 (18.95 - 33.13)	23.70 (16.59 - 30.81)	Estimated based on data from OneHealth Tool[9]
c_c.phc<br>c_d.phc<br>c_f.phc<br>c_h.phc	TB treatment at health centre	396.22 (220.27 - 572.18)	158.51 (81.18 - 235.85)	[4, 14]
c_a.h<br>c_clin.h	TB clinical assessment at hospital	14.37 (7.79 - 20.96)	61.00 (30.76 - 91.23)	[4]
c_e.h<br>c_g.h	TB clinical reassessment at hospital	7.19 (4.51 - 9.87)	30.50 (17.84 - 43.16)	[4]
c_c.h<br>c_f.h<br>c_d.h<br>c_h.h	TB treatment at hospital	396.22 (220.27 - 572.18)	158.51 (81.18 - 235.85)	[4, 14]

For peer review only - http://bmjopen.bmj.com/site/about/guidelines.xhtml

Table A2 Comparison of estimated unit costs for diagnostic visit, sputum collection, smear microscopy examination and XpertMTB/RIF test with recently published Better estimates of the costs of TB control (Value TB) project[13].

	Country	Diagnostic visit	Sputum collection	Smear microscopy examination	XpertMTB/RIF test
Health centre	ETH	5.84 (1.14-18.14)	4.64	1.69 (2.40-4.96)	26.47 (16.67-49.03)
	IND	9.91 (1.89-30.54)	4.22	3.77	27.07 (17.04-49.40)
	Value-TB ETH	3.33 (1.00-9.18)	3.28 (1-7.77)	4.53 (1.00-10.30)	20.83 (16-26.69)
Primary hospital	ETH	5.84 (1.61-26.39)	4.64	1.69 (2.40-4.96)	26.47 (16.67-49.03)
	IND	9.91 (2.75-18.76)	4.22	3.77	27.07 (17.04-49.40)
	Value-TB ETH	6.41 (1-14.66)	5.12 (3.00-6.91)	6.24 (4.00-8.43)	37.87 (19-57.78)

For peer review only

For peer review only - http://bmjopen.bmj.com/site/about/guidelines.xhtml

References

1. Ministry of Health Ethiopia., *Guidelines for Clinical and Programmatic Management of TB, DR-TB and Leprosy in Ethiopia, 7th edition*. 2020, Ministry of Health Ethiopia: Addis Ababa, Ethiopia.
2. Ministry of Health Republic of Indonesia., *National guideline for TB control*. 2016, Ministry of Health Republic of Indonesia: Jakarta, Indonesia.
3. World Bank. *World Bank national accounts data, and OECD National Accounts data files. GDP deflator*. 2021 14/05/2021]; Available from: https://data.worldbank.org/indicator/NY.GDP.DEFL.ZS.
4. Stenberg, K., et al., *Econometric estimation of WHO-CHOICE country-specific costs for inpatient and outpatient health service delivery.* Cost Eff Resour Alloc, 2018. **16**: p. 11.
5. Tesfaye, A., et al., *Modeling the patient and health system impacts of alternative xpert® MTB/RIF algorithms for the diagnosis of pulmonary tuberculosis in Addis Ababa, Ethiopia.* BMC infectious diseases, 2017. **17**(1): p. 318-318.
6. Jarrah Z, C.D., Hafidz F. , *The cost of scaling up TB services in Indonesia. TB CARE I–management sciences for health. Cambridge: Management Science for Health. .* 2013.
7. Peter, J.G., et al., *Comparison of two methods for acquisition of sputum samples for diagnosis of suspected tuberculosis in smear-negative or sputum-scarce people: a randomised controlled trial.* Lancet Respir Med, 2013. **1**(6): p. 471-8.
8. Paediatric Operational Sustainability Expertise Exchange group. *Paediatric Operational Sustainability Expertise Exchange group (POSEE group) budgeting tools* 2020 [cited 16/02/2021; Available from: https://www.dropbox.com/sh/rmzlqoot9m1muxe/AACOPh8SuS4WwMQD_vfN-oHCa?dl=0.
9. World Health Organization. *OneHealth Tool*. 2020; Available from: http://www.who.int/choice/onehealthtool/en/.
10. Stop TB Partnership | Global Drug Facility, *Global Drug Facility (GDF) Diagnostics catalogue August 2020.* 2021.
11. Sarin, S., et al., *Cost and operational impact of promoting upfront GeneXpert MTB/RIF test referrals for presumptive pediatric tuberculosis patients in India.* PLOS ONE, 2019. **14**(4): p. e0214675.
12. Sohn, H., et al., *Cost and affordability analysis of TB-LAMP and Xpert MTB/RIF assays as routine diagnostic tests in peripheral laboratories in Malawi and Vietnam.* J Glob Health Sci, 2019. **1**(1).
13. Pooran, A., et al., *Point of care Xpert MTB/RIF versus smear microscopy for tuberculosis diagnosis in southern African primary care clinics: a multicentre economic evaluation.* The Lancet. Global health, 2019. **7**(6): p. e798-e807.
14. Stop TB Partnership | Global Drug Facility, *Global Drug Facility (GDF) Medicines catalogue January 2021.* 2021.
15. Sweeney, S., et al., *Value TB Dataset: costs per intervention*. 2021, Harvard Dataverse.

For peer review only - http://bmjopen.bmj.com/site/about/guidelines.xhtml

Xpert on stool to diagnose tuberculosis in children is cost-effective in Ethiopia and Indonesia: a model-based cost-effectiveness analysis

Appendix 3: Supplementary results

Additional results for base case analysis 2
Age-specific results 2
Age 0-4 years 2
Age 5-14 years 4
Cost-effectiveness acceptability curves 6

Results for low prevalence sensitivity analysis 8
Age-specific results 8
All ages: 0-14 years 8
Age 0-4 years 10
Age 5-14 years 12

Results for Xpert baseline sensitivity analysis 14
Age-specific results 14
All ages: 0-14 years 14
Age 0-4 years 16
Age 5-14 years 18

Results for 0% discount rate sensitivity analysis 20
Age-specific results 20
All ages: 0-14 years 20
Age 0-4 years 22
Age 5-14 years 24

Results for 5% discount rate sensitivity analysis 26
Age-specific results 26
All ages: 0-14 years 26
Age 0-4 years 28
Age 5-14 years 30

Comparison of ICERs for sensitivity analyses 32

For peer review only

Additional results for base case analysis

Age-specific results

Age 0-4 years

Table A1 Ethiopia

Quantity per 100 children with presumptive TB (unless stated):	Standard of care	Intervention	Difference
children with true TB	45.5 (8.7 - 85.0)	45.5 (8.7 - 85.0)	0.0 (0.0 - 0.0)
assessments	208.1 (172.8 - 241.7)	252.9 (217.5 - 286.1)	44.7 (32.9 - 55.1)
bacteriological investigations	3.4 (2.1 - 4.9)	103.1 (87.6 - 112.5)	99.8 (84.3 - 109.1)
anti-TB treatments (ATT)	36.6 (15.3 - 59.7)	36.4 (15.2 - 59.3)	-0.2 (-2.8 - 2.7)
ATT initiated at PHC	68.3 (59.6 - 75.7)	81.0 (70.9 - 88.4)	12.7 (8.0 - 17.4)
percent of true-positive receiving ATT	66.2 (55.9 - 75.4)	66.8 (58.4 - 74.6)	0.7 (-3.4 - 5.1)
percent of ATT bacteriologically confirmed	0.6 (0.3 - 1.0)	31.4 (20.4 - 42.2)	30.8 (19.9 - 41.4)
percent of ATT false-positive	21.7 (2.4 - 65.8)	20.7 (2.4 - 63.4)	-1.0 (-4.2 - 1.9)
referrals, inc. self-referrals	40.1 (28.3 - 51.7)	14.7 (9.1 - 21.8)	-25.4 (-33.2 - -17.4)
deaths	7.5 (1.4 - 15.2)	7.3 (1.4 - 14.7)	-0.1 (-1.2 - 0.7)
life-years lost	205.9 (37.9 - 418.6)	202.4 (37.5 - 405.7)	-3.4 (-32.0 - 19.9)
cost	17934.1 (7124.0 - 35159.0)	17667.9 (7614.0 - 32685.5)	-266.2 (-13326.6 - 12081.4)

For peer review only

Table A2 Indonesia

Quantity per 100 children with presumptive TB (unless stated):	Standard of care	Intervention	Difference
children with true TB	45.5 (8.7 - 85.0)	45.5 (8.7 - 85.0)	0.0 (0.0 - 0.0)
assessments	209.4 (173.8 - 242.9)	255.2 (219.1 - 289.2)	45.9 (33.8 - 56.0)
bacteriological investigations	3.4 (2.1 - 5.0)	103.6 (88.2 - 113.0)	100.2 (84.9 - 109.5)
anti-TB treatments (ATT)	36.8 (15.4 - 60.4)	36.5 (15.2 - 59.6)	-0.3 (-3.0 - 2.4)
ATT initiated at PHC	70.1 (61.1 - 77.4)	83.6 (72.6 - 90.3)	13.4 (8.6 - 18.1)
percent of true-positive receiving ATT	66.6 (56.3 - 76.0)	67.0 (58.5 - 74.8)	0.4 (-3.7 - 4.8)
percent of ATT bacteriologically confirmed	0.6 (0.3 - 1.0)	31.4 (20.4 - 42.1)	30.8 (20.0 - 41.3)
percent of ATT false-positive	21.8 (2.4 - 65.8)	20.7 (2.4 - 63.7)	-1.1 (-4.3 - 1.9)
referrals, inc. self-referrals	41.5 (29.2 - 53.1)	15.3 (9.5 - 22.4)	-26.3 (-34.4 - -17.9)
deaths	7.4 (1.4 - 15.1)	7.3 (1.4 - 14.6)	-0.1 (-1.1 - 0.8)
life-years lost	209.8 (38.7 - 429.1)	207.8 (39.0 - 416.6)	-2.0 (-31.0 - 23.2)
cost	13672.3 (7286.9 - 22370.9)	14090.7 (8344.4 - 21727.0)	418.4 (-8064.8 - 9011.5)

For peer review only - http://bmjopen.bmj.com/site/about/guidelines.xhtml

Age 5-14 years

Table A3 Ethiopia

Quantity per 100 children with presumptive TB (unless stated):	Standard of care	Intervention	Difference
children with true TB	45.5 (8.7 - 85.0)	45.5 (8.7 - 85.0)	0.0 (0.0 - 0.0)
assessments	197.9 (169.6 - 225.2)	242.2 (200.8 - 281.7)	44.3 (24.8 - 62.7)
bacteriological investigations	47.1 (12.5 - 82.7)	101.9 (86.2 - 111.6)	54.8 (16.5 - 91.1)
anti-TB treatments (ATT)	29.6 (10.6 - 53.8)	42.6 (18.5 - 67.8)	13.0 (1.5 - 30.3)
ATT initiated at PHC	74.6 (63.8 - 84.3)	82.4 (71.8 - 90.2)	7.8 (2.1 - 12.9)
percent of true-positive receiving ATT	53.6 (31.9 - 72.9)	76.8 (69.4 - 83.1)	23.2 (5.1 - 45.4)
percent of ATT bacteriologically confirmed	14.5 (2.4 - 39.5)	33.7 (20.3 - 46.1)	19.2 (-5.9 - 37.4)
percent of ATT false-positive	21.6 (2.6 - 65.3)	22.4 (2.8 - 66.2)	0.8 (-2.5 - 6.2)
referrals, inc. self-referrals	23.1 (8.8 - 39.1)	13.3 (7.3 - 20.5)	-9.7 (-22.7 - 4.4)
deaths	3.4 (0.6 - 7.4)	1.9 (0.3 - 3.9)	-1.5 (-4.0 - -0.2)
life-years lost	93.7 (16.9 - 203.0)	52.5 (9.6 - 107.9)	-41.1 (-111.6 - -4.3)
cost	14407.8 (5303.7 - 29936.5)	20277.4 (8872.7 - 37127.0)	5869.6 (-6634.5 - 19361.5)

For peer review only

Table A4 Indonesia

Quantity per 100 children with presumptive TB (unless stated):	Standard of care	Intervention	Difference
children with true TB	45.5 (8.7 - 85.0)	45.5 (8.7 - 85.0)	0.0 (0.0 - 0.0)
assessments	198.8 (170.2 - 226.2)	244.3 (202.0 - 284.1)	45.5 (25.6 - 64.3)
bacteriological investigations	47.3 (12.7 - 83.3)	102.3 (86.7 - 112.2)	54.9 (16.6 - 91.6)
anti-TB treatments (ATT)	29.6 (10.5 - 54.2)	42.7 (18.5 - 68.1)	13.1 (1.4 - 30.8)
ATT initiated at PHC	77.2 (65.5 - 87.0)	85.1 (73.6 - 92.2)	7.9 (1.5 - 13.5)
percent of true-positive receiving ATT	53.6 (31.5 - 73.0)	76.9 (69.6 - 83.3)	23.3 (4.9 - 45.9)
percent of ATT bacteriologically confirmed	14.1 (2.3 - 38.3)	33.7 (20.2 - 46.1)	19.5 (-4.9 - 37.4)
percent of ATT false-positive	21.7 (2.6 - 65.3)	22.4 (2.8 - 66.3)	0.8 (-2.6 - 6.1)
referrals, inc. self-referrals	23.9 (9.1 - 40.3)	13.8 (7.5 - 21.2)	-10.1 (-23.7 - 4.3)
deaths	3.4 (0.6 - 7.4)	1.9 (0.4 - 3.9)	-1.5 (-4.1 - -0.1)
life-years lost	96.6 (17.4 - 210.3)	53.9 (10.0 - 111.1)	-42.7 (-115.7 - -4.3)
cost	11270.2 (6013.6 - 18958.7)	14987.1 (8815.0 - 23229.9)	3716.9 (-3812.4 - 10646.6)

For peer review only - http://bmjopen.bmj.com/site/about/guidelines.xhtml

Cost-effectiveness acceptability curves

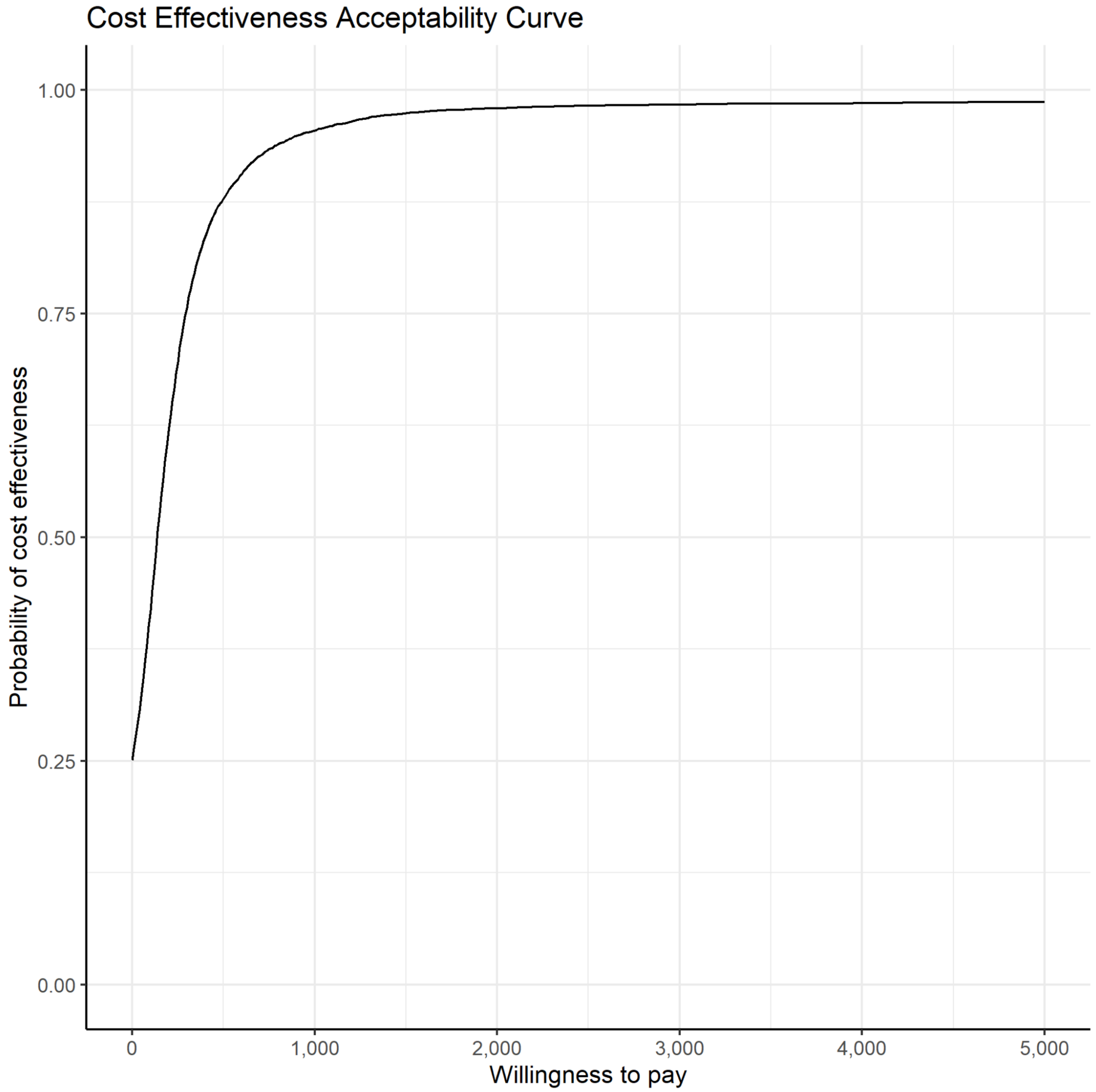

Figure A1 Ethiopia

For peer review only - http://bmjopen.bmj.com/site/about/guidelines.xhtml

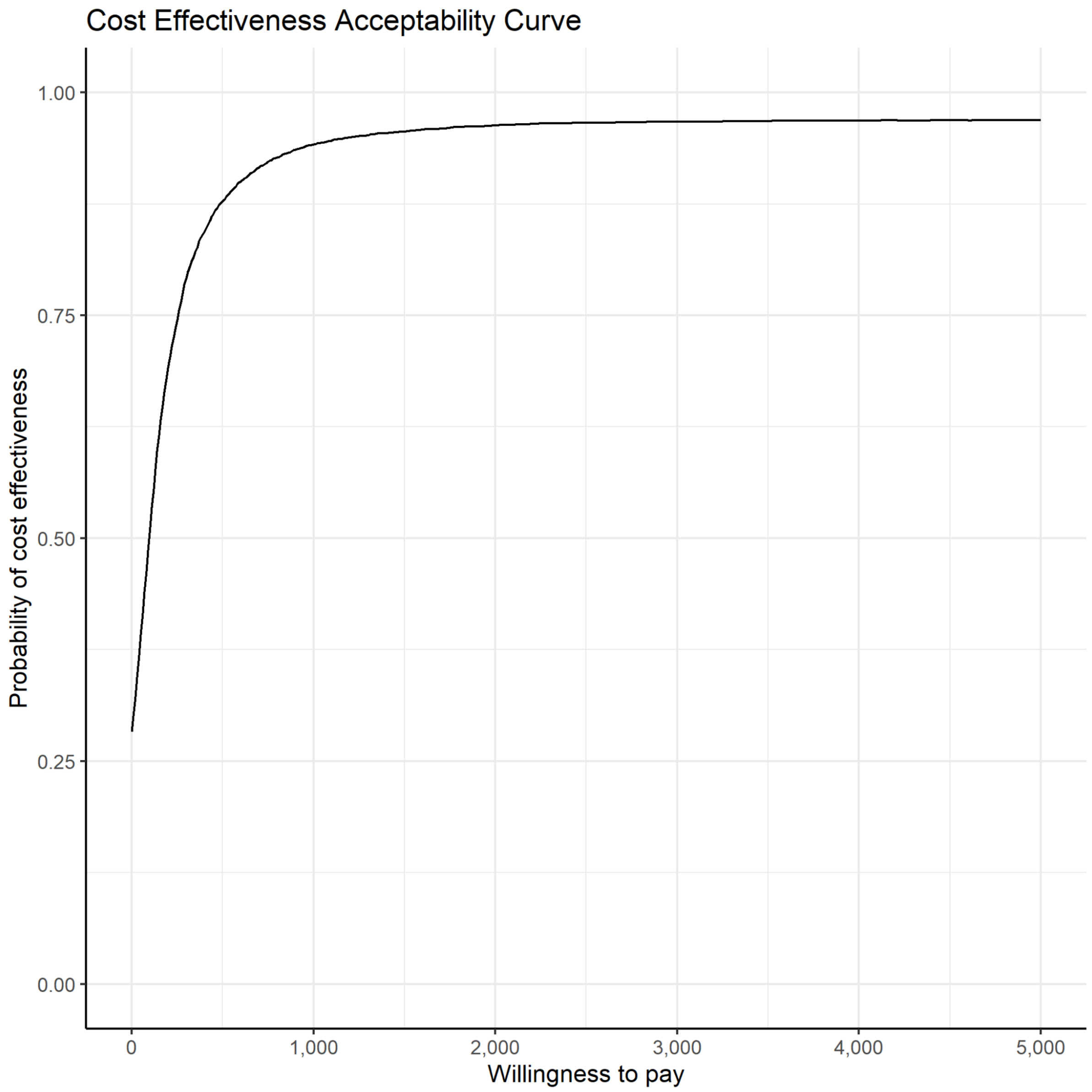

Figure A2 Indonesia

For peer review only - http://bmjopen.bmj.com/site/about/guidelines.xhtml

Results for low prevalence sensitivity analysis

Age-specific results

All ages: 0-14 years

Table A5 Ethiopia

Quantity per 100 children with presumptive TB (unless stated):	Standard of care	Intervention	Difference
children with true TB	22.8 (4.4 - 42.5)	22.8 (4.4 - 42.5)	0.0 (0.0 - 0.0)
assessments	224.4 (203.7 - 248.1)	260.5 (239.5 - 281.9)	36.0 (22.2 - 53.2)
bacteriological investigations	32.9 (9.7 - 60.1)	98.3 (85.7 - 110.0)	65.3 (36.2 - 91.3)
anti-TB treatments (ATT)	22.3 (11.0 - 35.6)	25.5 (13.5 - 38.0)	3.2 (-3.1 - 11.4)
ATT initiated at PHC	66.7 (56.8 - 76.9)	84.0 (72.4 - 93.3)	17.3 (8.7 - 26.2)
percent of true-positive receiving ATT	60.4 (44.3 - 74.6)	71.2 (64.4 - 77.6)	10.8 (-2.7 - 27.7)
percent of ATT bacteriologically confirmed	6.9 (1.5 - 17.2)	29.0 (17.8 - 39.9)	22.1 (9.7 - 34.0)
percent of ATT false-positive	41.2 (17.1 - 79.8)	39.7 (16.7 - 78.8)	-1.5 (-5.9 - 4.2)
referrals, inc. self-referrals	41.7 (22.6 - 60.9)	9.4 (2.0 - 21.6)	-32.2 (-52.2 - -12.2)
deaths	2.3 (0.4 - 4.8)	2.1 (0.4 - 4.4)	-0.2 (-1.1 - 0.3)
life-years lost	64.0 (11.7 - 131.4)	57.3 (10.4 - 120.7)	-6.7 (-30.5 - 9.3)
cost	11688.2 (5594.7 - 21367.9)	12666.3 (6357.3 - 21679.1)	978.1 (-7407.9 - 9219.3)

For peer review only - http://bmjopen.bmj.com/site/about/guidelines.xhtml

Table A6 Indonesia

Quantity per 100 children with presumptive TB (unless stated):	Standard of care	Intervention	Difference
children with true TB	22.8 (4.4 - 42.5)	22.8 (4.4 - 42.5)	0.0 (0.0 - 0.0)
assessments	221.3 (204.3 - 239.6)	271.1 (248.7 - 292.5)	49.8 (37.8 - 60.8)
bacteriological investigations	25.5 (8.1 - 43.9)	105.5 (90.4 - 113.9)	80.0 (56.7 - 100.3)
anti-TB treatments (ATT)	22.1 (11.2 - 34.2)	26.0 (14.1 - 38.5)	4.0 (-0.4 - 9.9)
ATT initiated at PHC	70.8 (61.5 - 78.1)	82.7 (71.8 - 89.5)	11.9 (7.2 - 16.4)
percent of true-positive receiving ATT	60.3 (48.2 - 71.4)	71.8 (65.9 - 77.3)	11.5 (1.8 - 23.1)
percent of ATT bacteriologically confirmed	5.0 (1.2 - 11.2)	28.7 (17.8 - 39.1)	23.6 (13.1 - 34.3)
percent of ATT false-positive	40.8 (16.7 - 79.7)	40.5 (17.4 - 79.3)	-0.3 (-4.1 - 5.0)
referrals, inc. self-referrals	38.2 (27.9 - 48.3)	17.4 (12.9 - 23.8)	-20.8 (-29.8 - -10.8)
deaths	2.7 (0.5 - 5.4)	2.3 (0.4 - 4.6)	-0.4 (-1.1 - 0.0)
life-years lost	77.4 (14.7 - 155.1)	66.5 (12.4 - 132.3)	-10.9 (-30.9 - 0.1)
cost	10894.9 (6314.8 - 17164.5)	12513.6 (7709.2 - 18868.1)	1618.7 (-4945.3 - 8121.1)

For peer review only - http://bmjopen.bmj.com/site/about/guidelines.xhtml

Age 0-4 years

Table A7 Ethiopia

Quantity per 100 children with presumptive TB (unless stated):	Standard of care	Intervention	Difference
children with true TB	22.8 (4.4 - 42.5)	22.8 (4.4 - 42.5)	0.0 (0.0 - 0.0)
assessments	235.5 (209.9 - 262.1)	264.5 (244.8 - 284.9)	29.0 (13.7 - 47.9)
bacteriological investigations	3.7 (2.3 - 5.5)	98.6 (86.2 - 110.5)	94.8 (82.5 - 106.6)
anti-TB treatments (ATT)	25.5 (12.3 - 39.3)	22.6 (11.0 - 34.7)	-3.0 (-6.9 - 0.4)
ATT initiated at PHC	62.6 (53.4 - 72.7)	83.6 (71.9 - 93.1)	21.0 (11.6 - 30.7)
percent of true-positive receiving ATT	69.3 (57.6 - 79.5)	64.8 (55.8 - 73.1)	-4.5 (-10.6 - 2.6)
percent of ATT bacteriologically confirmed	0.6 (0.3 - 1.0)	28.8 (17.8 - 39.7)	28.2 (17.4 - 38.9)
percent of ATT false-positive	40.7 (14.3 - 80.7)	37.8 (14.0 - 77.9)	-2.9 (-6.6 - 1.8)
referrals, inc. self-referrals	56.0 (35.1 - 73.4)	9.8 (2.1 - 22.3)	-46.2 (-64.8 - -25.7)
deaths	3.4 (0.6 - 7.2)	3.9 (0.7 - 7.8)	0.4 (-0.2 - 1.4)
life-years lost	94.9 (17.3 - 197.9)	106.6 (19.9 - 213.9)	11.7 (-6.3 - 37.5)
cost	13498.5 (6292.1 - 24528.2)	11443.3 (5638.1 - 19702.2)	-2055.2 (-11147.7 - 5839.4)

For peer review only - http://bmjopen.bmj.com/site/about/guidelines.xhtml

Table A8 Indonesia

Quantity per 100 children with presumptive TB (unless stated):	Standard of care	Intervention	Difference
children with true TB	22.8 (4.4 - 42.5)	22.8 (4.4 - 42.5)	0.0 (0.0 - 0.0)
assessments	228.3 (208.1 - 249.0)	274.5 (252.9 - 294.7)	46.2 (34.0 - 55.8)
bacteriological investigations	3.5 (2.2 - 5.2)	106.0 (90.9 - 114.3)	102.5 (87.3 - 110.8)
anti-TB treatments (ATT)	24.4 (11.7 - 37.5)	23.8 (11.8 - 36.2)	-0.6 (-3.0 - 1.4)
ATT initiated at PHC	67.8 (59.3 - 75.2)	82.1 (71.5 - 88.8)	14.2 (9.1 - 18.9)
percent of true-positive receiving ATT	66.6 (56.3 - 76.0)	67.0 (58.5 - 74.8)	0.4 (-3.7 - 4.8)
percent of ATT bacteriologically confirmed	0.5 (0.3 - 1.0)	28.7 (17.6 - 39.6)	28.1 (17.2 - 38.9)
percent of ATT false-positive	40.3 (14.1 - 80.3)	38.8 (14.6 - 78.5)	-1.5 (-4.8 - 3.1)
referrals, inc. self-referrals	47.5 (38.8 - 55.3)	17.9 (13.4 - 24.4)	-29.6 (-35.9 - -22.1)
deaths	3.7 (0.7 - 7.5)	3.7 (0.7 - 7.3)	-0.0 (-0.5 - 0.4)
life-years lost	104.9 (19.4 - 214.5)	103.9 (19.5 - 208.3)	-1.0 (-15.5 - 11.6)
cost	11896.1 (6406.6 - 19196.8)	12176.0 (7451.0 - 18467.1)	279.8 (-7399.6 - 7997.8)

For peer review only - http://bmjopen.bmj.com/site/about/guidelines.xhtml

Age 5-14 years

Table A9 Ethiopia

Quantity per 100 children with presumptive TB (unless stated):	Standard of care	Intervention	Difference
children with true TB	22.8 (4.4 - 42.5)	22.8 (4.4 - 42.5)	0.0 (0.0 - 0.0)
assessments	217.8 (198.9 - 241.4)	258.0 (236.2 - 280.4)	40.3 (25.0 - 58.7)
bacteriological investigations	50.4 (13.8 - 85.9)	98.1 (85.4 - 109.7)	47.6 (10.1 - 85.7)
anti-TB treatments (ATT)	20.3 (8.1 - 35.7)	27.2 (14.0 - 40.7)	6.9 (-2.2 - 18.6)
ATT initiated at PHC	70.2 (58.8 - 81.9)	84.2 (72.7 - 93.4)	14.1 (4.7 - 23.9)
percent of true-positive receiving ATT	55.1 (32.5 - 75.7)	75.1 (67.2 - 82.0)	20.0 (0.5 - 43.3)
percent of ATT bacteriologically confirmed	12.7 (2.1 - 35.4)	29.4 (16.9 - 41.7)	16.6 (-5.9 - 33.0)
percent of ATT false-positive	40.6 (15.3 - 80.4)	40.2 (16.0 - 79.7)	-0.4 (-5.3 - 6.7)
referrals, inc. self-referrals	33.1 (12.2 - 55.5)	9.2 (1.9 - 21.2)	-23.8 (-47.1 - -1.3)
deaths	1.6 (0.3 - 3.6)	1.0 (0.2 - 2.1)	-0.6 (-1.9 - -0.0)
life-years lost	45.5 (8.0 - 100.0)	27.7 (5.1 - 57.5)	-17.8 (-51.9 - -0.3)
cost	10603.2 (4477.3 - 20733.1)	13401.2 (6616.6 - 22999.5)	2798.0 (-6051.3 - 11513.5)

For peer review only - http://bmjopen.bmj.com/site/about/guidelines.xhtml

Table A10 Indonesia

Quantity per 100 children with presumptive TB (unless stated):	Standard of care	Intervention	Difference
children with true TB	22.8 (4.4 - 42.5)	22.8 (4.4 - 42.5)	0.0 (0.0 - 0.0)
assessments	213.8 (198.0 - 233.1)	267.4 (243.3 - 290.7)	53.6 (39.5 - 69.5)
bacteriological investigations	48.9 (13.3 - 84.6)	105.1 (89.9 - 113.6)	56.2 (18.0 - 93.5)
anti-TB treatments (ATT)	19.6 (7.9 - 34.4)	28.5 (15.0 - 42.2)	8.8 (0.5 - 20.4)
ATT initiated at PHC	75.1 (63.7 - 85.5)	83.2 (72.2 - 90.3)	8.1 (0.7 - 14.4)
percent of true-positive receiving ATT	53.6 (31.5 - 73.0)	76.9 (69.6 - 83.3)	23.3 (4.9 - 45.9)
percent of ATT bacteriologically confirmed	12.2 (1.9 - 34.3)	29.1 (16.6 - 41.3)	16.9 (-4.8 - 33.0)
percent of ATT false-positive	40.2 (15.1 - 79.9)	41.4 (16.8 - 80.4)	1.2 (-3.2 - 8.0)
referrals, inc. self-referrals	28.3 (11.5 - 44.1)	16.9 (12.3 - 23.3)	-11.4 (-26.4 - 5.6)
deaths	1.7 (0.3 - 3.7)	0.9 (0.2 - 2.0)	-0.8 (-2.0 - -0.1)
life-years lost	48.3 (8.7 - 105.1)	26.9 (5.0 - 55.6)	-21.4 (-57.9 - -2.1)
cost	9830.4 (5352.0 - 15979.3)	12871.7 (7911.6 - 19463.3)	3041.3 (-3390.0 - 8592.6)

For peer review only

Results for Xpert baseline sensitivity analysis

Age-specific results

All ages: 0-14 years

Table A11 Ethiopia

Quantity per 100 children with presumptive TB (unless stated):	Standard of care	Intervention	Difference
children with true TB	45.5 (8.7 - 85.0)	45.5 (8.7 - 85.0)	0.0 (0.0 - 0.0)
assessments	199.4 (168.3 - 230.2)	246.2 (207.3 - 283.3)	46.8 (33.2 - 59.9)
bacteriological investigations	30.7 (8.7 - 57.5)	102.3 (86.8 - 112.0)	71.7 (41.5 - 96.3)
anti-TB treatments (ATT)	34.3 (14.2 - 56.8)	40.3 (17.6 - 64.4)	6.0 (0.1 - 15.0)
ATT initiated at PHC	73.6 (63.9 - 81.8)	81.9 (71.6 - 89.5)	8.4 (3.2 - 13.2)
percent of true-positive receiving ATT	62.6 (51.4 - 72.7)	73.0 (66.7 - 78.8)	10.5 (1.6 - 22.0)
percent of ATT bacteriologically confirmed	14.0 (3.0 - 32.6)	32.8 (20.7 - 44.1)	18.8 (-0.2 - 34.6)
percent of ATT false-positive	21.3 (2.7 - 63.8)	21.9 (2.9 - 64.9)	0.6 (-2.4 - 5.1)
referrals, inc. self-referrals	29.5 (17.0 - 42.9)	13.8 (8.0 - 21.0)	-15.6 (-25.8 - -4.9)
deaths	4.6 (0.9 - 9.4)	3.9 (0.7 - 8.3)	-0.7 (-2.0 - 0.0)
life-years lost	127.7 (23.8 - 260.7)	108.7 (19.7 - 228.5)	-19.0 (-55.1 - 0.1)
cost	16678.2 (6843.1 - 32205.5)	19297.7 (8413.8 - 35444.7)	2619.5 (-9141.3 - 14513.1)

For peer review only - http://bmjopen.bmj.com/site/about/guidelines.xhtml

Table A12 Indonesia

Quantity per 100 children with presumptive TB (unless stated):	Standard of care	Intervention	Difference
children with true TB	45.5 (8.7 - 85.0)	45.5 (8.7 - 85.0)	0.0 (0.0 - 0.0)
assessments	202.3 (170.6 - 232.9)	249.9 (211.2 - 286.5)	47.7 (34.7 - 59.6)
bacteriological investigations	24.7 (7.8 - 43.2)	103.0 (87.5 - 112.6)	78.2 (54.5 - 98.4)
anti-TB treatments (ATT)	35.1 (14.8 - 57.5)	39.5 (17.1 - 63.3)	4.4 (-0.4 - 11.1)
ATT initiated at PHC	74.4 (64.7 - 81.7)	84.4 (73.2 - 91.2)	10.0 (5.4 - 14.6)
percent of true-positive receiving ATT	63.8 (54.3 - 72.8)	71.8 (65.9 - 77.3)	8.0 (0.6 - 16.6)
percent of ATT bacteriologically confirmed	10.7 (2.6 - 22.7)	32.5 (20.9 - 43.4)	21.7 (7.7 - 35.3)
percent of ATT false-positive	21.6 (2.7 - 64.4)	21.8 (2.9 - 64.6)	0.2 (-2.8 - 4.2)
referrals, inc. self-referrals	33.0 (21.5 - 45.5)	14.5 (8.6 - 21.7)	-18.4 (-27.6 - -9.5)
deaths	5.2 (1.0 - 10.3)	4.7 (0.9 - 9.3)	-0.5 (-1.6 - 0.1)
life-years lost	147.8 (28.1 - 294.2)	133.1 (24.7 - 264.6)	-14.7 (-45.7 - 3.5)
cost	12852.5 (7260.6 - 20698.7)	14525.7 (8603.6 - 22403.0)	1673.3 (-5630.9 - 8936.4)

For peer review only

For peer review only - http://bmjopen.bmj.com/site/about/guidelines.xhtml

Age 0-4 years

Table A13 Ethiopia

Quantity per 100 children with presumptive TB (unless stated):	Standard of care	Intervention	Difference
children with true TB	45.5 (8.7 - 85.0)	45.5 (8.7 - 85.0)	0.0 (0.0 - 0.0)
assessments	208.0 (172.6 - 241.6)	252.9 (217.5 - 286.1)	44.9 (33.1 - 55.2)
bacteriological investigations	3.4 (2.1 - 4.9)	103.1 (87.6 - 112.5)	99.8 (84.3 - 109.1)
anti-TB treatments (ATT)	36.7 (15.4 - 59.9)	36.4 (15.2 - 59.3)	-0.3 (-2.9 - 2.5)
ATT initiated at PHC	68.4 (59.7 - 75.8)	81.0 (70.9 - 88.4)	12.6 (7.9 - 17.2)
percent of true-positive receiving ATT	66.5 (56.2 - 75.7)	66.8 (58.4 - 74.6)	0.3 (-3.7 - 4.8)
percent of ATT bacteriologically confirmed	1.1 (0.5 - 1.7)	31.4 (20.4 - 42.2)	30.3 (19.7 - 41.0)
percent of ATT false-positive	21.7 (2.4 - 65.7)	20.7 (2.4 - 63.4)	-1.0 (-4.1 - 1.9)
referrals, inc. self-referrals	40.1 (28.3 - 51.7)	14.7 (9.1 - 21.8)	-25.4 (-33.2 - -17.4)
deaths	7.4 (1.4 - 15.1)	7.3 (1.4 - 14.7)	-0.1 (-1.1 - 0.8)
life-years lost	204.3 (37.6 - 416.1)	202.4 (37.5 - 405.7)	-1.8 (-29.6 - 21.8)
cost	18004.2 (7149.6 - 35253.8)	17667.9 (7614.0 - 32685.5)	-336.2 (-13382.6 - 11963.8)

For peer review only - http://bmjopen.bmj.com/site/about/guidelines.xhtml

Table A14 Indonesia

Quantity per 100 children with presumptive TB (unless stated):	Standard of care	Intervention	Difference
children with true TB	45.5 (8.7 - 85.0)	45.5 (8.7 - 85.0)	0.0 (0.0 - 0.0)
assessments	209.2 (173.5 - 242.9)	255.2 (219.1 - 289.2)	46.1 (34.0 - 56.2)
bacteriological investigations	3.4 (2.1 - 5.0)	103.6 (88.2 - 113.0)	100.2 (84.9 - 109.5)
anti-TB treatments (ATT)	37.0 (15.5 - 60.7)	36.5 (15.2 - 59.6)	-0.5 (-3.1 - 2.2)
ATT initiated at PHC	70.3 (61.3 - 77.5)	83.6 (72.6 - 90.3)	13.3 (8.4 - 17.9)
percent of true-positive receiving ATT	67.0 (56.6 - 76.3)	67.0 (58.5 - 74.8)	0.1 (-3.9 - 4.5)
percent of ATT bacteriologically confirmed	1.1 (0.5 - 1.7)	31.4 (20.4 - 42.1)	30.3 (19.6 - 40.9)
percent of ATT false-positive	21.8 (2.4 - 65.7)	20.7 (2.4 - 63.7)	-1.0 (-4.2 - 1.9)
referrals, inc. self-referrals	41.5 (29.2 - 53.1)	15.3 (9.5 - 22.4)	-26.3 (-34.4 - -17.9)
deaths	7.3 (1.4 - 15.0)	7.3 (1.4 - 14.6)	-0.0 (-1.0 - 0.9)
life-years lost	208.1 (38.4 - 426.3)	207.8 (39.0 - 416.6)	-0.3 (-28.4 - 25.5)
cost	13704.3 (7307.0 - 22423.2)	14090.7 (8344.4 - 21727.0)	386.4 (-8103.9 - 8967.9)

For peer review only - http://bmjopen.bmj.com/site/about/guidelines.xhtml

Age 5-14 years

Table A15 Ethiopia

Quantity per 100 children with presumptive TB (unless stated):	Standard of care	Intervention	Difference
children with true TB	45.5 (8.7 - 85.0)	45.5 (8.7 - 85.0)	0.0 (0.0 - 0.0)
assessments	194.2 (164.3 - 224.2)	242.2 (200.8 - 281.7)	48.0 (31.6 - 65.4)
bacteriological investigations	47.1 (12.5 - 82.7)	101.9 (86.2 - 111.6)	54.8 (16.5 - 91.1)
anti-TB treatments (ATT)	32.9 (12.5 - 56.8)	42.6 (18.5 - 67.8)	9.7 (0.9 - 22.5)
ATT initiated at PHC	77.2 (66.1 - 87.6)	82.4 (71.8 - 90.2)	5.2 (-2.2 - 11.8)
percent of true-positive receiving ATT	60.2 (44.6 - 74.9)	76.8 (69.4 - 83.1)	16.5 (3.3 - 32.6)
percent of ATT bacteriologically confirmed	23.5 (4.2 - 56.3)	33.7 (20.3 - 46.1)	10.2 (-22.8 - 33.6)
percent of ATT false-positive	20.7 (2.5 - 64.1)	22.4 (2.8 - 66.2)	1.7 (-1.9 - 8.3)
referrals, inc. self-referrals	23.0 (8.8 - 39.1)	13.3 (7.3 - 20.5)	-9.7 (-22.7 - 4.4)
deaths	3.0 (0.6 - 6.3)	1.9 (0.3 - 3.9)	-1.1 (-2.9 - -0.1)
life-years lost	81.8 (15.2 - 172.9)	52.5 (9.6 - 107.9)	-29.3 (-80.4 - -2.9)
cost	15884.6 (6196.1 - 31393.0)	20277.4 (8872.7 - 37127.0)	4392.8 (-7520.5 - 16511.0)

For peer review only - http://bmjopen.bmj.com/site/about/guidelines.xhtml

Table A16 Indonesia

Quantity per 100 children with presumptive TB (unless stated):	Standard of care	Intervention	Difference
children with true TB	45.5 (8.7 - 85.0)	45.5 (8.7 - 85.0)	0.0 (0.0 - 0.0)
assessments	194.9 (164.8 - 225.1)	244.3 (202.0 - 284.1)	49.4 (32.8 - 67.1)
bacteriological investigations	47.3 (12.7 - 83.3)	102.3 (86.7 - 112.2)	54.9 (16.6 - 91.6)
anti-TB treatments (ATT)	33.1 (12.6 - 57.0)	42.7 (18.5 - 68.1)	9.7 (0.8 - 22.5)
ATT initiated at PHC	79.6 (67.8 - 90.1)	85.1 (73.6 - 92.2)	5.5 (-2.4 - 12.6)
percent of true-positive receiving ATT	60.5 (44.7 - 75.1)	76.9 (69.6 - 83.3)	16.5 (3.1 - 32.6)
percent of ATT bacteriologically confirmed	23.5 (4.3 - 56.3)	33.7 (20.2 - 46.1)	10.2 (-22.6 - 33.6)
percent of ATT false-positive	20.8 (2.5 - 64.3)	22.4 (2.8 - 66.3)	1.7 (-1.9 - 8.3)
referrals, inc. self-referrals	23.8 (9.0 - 40.3)	13.8 (7.5 - 21.2)	-10.1 (-23.7 - 4.3)
deaths	2.9 (0.6 - 6.3)	1.9 (0.4 - 3.9)	-1.1 (-2.9 - -0.1)
life-years lost	83.9 (15.8 - 178.7)	53.9 (10.0 - 111.1)	-30.1 (-83.3 - -2.7)
cost	11945.9 (6523.9 - 19684.3)	14987.1 (8815.0 - 23229.9)	3041.3 (-4161.7 - 9508.2)

For peer review only

Results for 0% discount rate sensitivity analysis

Age-specific results

All ages: 0-14 years

Table A17 Ethiopia

Quantity per 100 children with presumptive TB (unless stated):	Standard of care	Intervention	Difference
children with true TB	45.5 (8.7 - 85.0)	45.5 (8.7 - 85.0)	0.0 (0.0 - 0.0)
assessments	201.8 (171.8 - 230.9)	246.2 (207.3 - 283.3)	44.4 (29.5 - 58.1)
bacteriological investigations	30.7 (8.7 - 57.5)	102.3 (86.8 - 112.0)	71.7 (41.5 - 96.3)
anti-TB treatments (ATT)	32.2 (13.2 - 54.5)	40.3 (17.6 - 64.4)	8.1 (0.6 - 20.3)
ATT initiated at PHC	71.8 (62.3 - 79.6)	81.9 (71.6 - 89.5)	10.1 (5.8 - 14.2)
percent of true-positive receiving ATT	58.3 (43.0 - 71.1)	73.0 (66.7 - 78.8)	14.7 (2.8 - 30.5)
percent of ATT bacteriologically confirmed	8.0 (1.7 - 19.8)	32.8 (20.7 - 44.1)	24.8 (10.6 - 37.8)
percent of ATT false-positive	21.9 (2.8 - 64.6)	21.9 (2.9 - 64.9)	0.0 (-3.0 - 4.0)
referrals, inc. self-referrals	29.5 (17.0 - 42.9)	13.8 (8.0 - 21.0)	-15.6 (-25.8 - -4.9)
deaths	4.9 (0.9 - 10.0)	3.9 (0.7 - 8.3)	-1.0 (-2.8 - -0.1)
life-years lost	339.1 (62.7 - 692.0)	271.6 (49.2 - 570.9)	-67.5 (-189.8 - -4.1)
cost	15729.4 (6368.3 - 31027.5)	19297.7 (8413.8 - 35444.7)	3568.3 (-8472.2 - 16311.6)

For peer review only - http://bmjopen.bmj.com/site/about/guidelines.xhtml

Table A18 Indonesia

Quantity per 100 children with presumptive TB (unless stated):	Standard of care	Intervention	Difference
children with true TB	45.5 (8.7 - 85.0)	45.5 (8.7 - 85.0)	0.0 (0.0 - 0.0)
assessments	204.2 (173.4 - 233.5)	249.9 (211.2 - 286.5)	45.7 (31.9 - 58.0)
bacteriological investigations	24.7 (7.8 - 43.2)	103.0 (87.5 - 112.6)	78.2 (54.5 - 98.4)
anti-TB treatments (ATT)	33.3 (14.1 - 55.3)	39.5 (17.1 - 63.3)	6.2 (0.1 - 15.2)
ATT initiated at PHC	73.0 (63.2 - 80.3)	84.4 (73.2 - 91.2)	11.3 (7.1 - 15.4)
percent of true-positive receiving ATT	60.3 (48.2 - 71.4)	71.8 (65.9 - 77.3)	11.5 (1.8 - 23.1)
percent of ATT bacteriologically confirmed	5.9 (1.4 - 12.9)	32.5 (20.9 - 43.4)	26.6 (14.9 - 38.2)
percent of ATT false-positive	22.0 (2.8 - 65.1)	21.8 (2.9 - 64.6)	-0.3 (-3.5 - 3.5)
referrals, inc. self-referrals	33.0 (21.5 - 45.5)	14.5 (8.6 - 21.7)	-18.4 (-27.6 - -9.6)
deaths	5.4 (1.0 - 10.9)	4.7 (0.9 - 9.3)	-0.8 (-2.2 - 0.0)
life-years lost	391.6 (74.2 - 784.5)	336.7 (62.6 - 669.4)	-55.0 (-156.1 - 0.6)
cost	12508.1 (7056.4 - 20279.0)	14525.7 (8603.6 - 22403.0)	2017.6 (-5421.3 - 9470.6)

For peer review only - http://bmjopen.bmj.com/site/about/guidelines.xhtml

Age 0-4 years

Table A19 Ethiopia

Quantity per 100 children with presumptive TB (unless stated):	Standard of care	Intervention	Difference
children with true TB	45.5 (8.7 - 85.0)	45.5 (8.7 - 85.0)	0.0 (0.0 - 0.0)
assessments	208.1 (172.8 - 241.7)	252.9 (217.5 - 286.1)	44.7 (32.9 - 55.1)
bacteriological investigations	3.4 (2.1 - 4.9)	103.1 (87.6 - 112.5)	99.8 (84.3 - 109.1)
anti-TB treatments (ATT)	36.6 (15.3 - 59.7)	36.4 (15.2 - 59.3)	-0.2 (-2.8 - 2.7)
ATT initiated at PHC	68.3 (59.6 - 75.7)	81.0 (70.9 - 88.4)	12.7 (8.0 - 17.4)
percent of true-positive receiving ATT	66.2 (55.9 - 75.4)	66.8 (58.4 - 74.6)	0.7 (-3.4 - 5.1)
percent of ATT bacteriologically confirmed	0.6 (0.3 - 1.0)	31.4 (20.4 - 42.2)	30.8 (19.9 - 41.4)
percent of ATT false-positive	21.7 (2.4 - 65.8)	20.7 (2.4 - 63.4)	-1.0 (-4.2 - 1.9)
referrals, inc. self-referrals	40.1 (28.3 - 51.7)	14.7 (9.1 - 21.8)	-25.4 (-33.2 - -17.4)
deaths	7.5 (1.4 - 15.2)	7.3 (1.4 - 14.7)	-0.1 (-1.2 - 0.7)
life-years lost	514.5 (94.8 - 1046.0)	505.9 (93.8 - 1013.6)	-8.6 (-79.9 - 49.8)
cost	17934.1 (7124.0 - 35159.0)	17667.9 (7614.0 - 32685.5)	-266.2 (-13326.6 - 12081.4)

For peer review only

For peer review only - http://bmjopen.bmj.com/site/about/guidelines.xhtml

Table A20 Indonesia

Quantity per 100 children with presumptive TB (unless stated):	Standard of care	Intervention	Difference
children with true TB	45.5 (8.7 - 85.0)	45.5 (8.7 - 85.0)	0.0 (0.0 - 0.0)
assessments	209.4 (173.8 - 242.9)	255.2 (219.1 - 289.2)	45.9 (33.8 - 56.0)
bacteriological investigations	3.4 (2.1 - 5.0)	103.6 (88.2 - 113.0)	100.2 (84.9 - 109.5)
anti-TB treatments (ATT)	36.8 (15.4 - 60.4)	36.5 (15.2 - 59.6)	-0.3 (-3.0 - 2.4)
ATT initiated at PHC	70.1 (61.1 - 77.4)	83.6 (72.6 - 90.3)	13.4 (8.6 - 18.1)
percent of true-positive receiving ATT	66.6 (56.3 - 76.0)	67.0 (58.5 - 74.8)	0.4 (-3.7 - 4.8)
percent of ATT bacteriologically confirmed	0.6 (0.3 - 1.0)	31.4 (20.4 - 42.1)	30.8 (20.0 - 41.3)
percent of ATT false-positive	21.8 (2.4 - 65.8)	20.7 (2.4 - 63.7)	-1.1 (-4.3 - 1.9)
referrals, inc. self-referrals	41.5 (29.2 - 53.1)	15.3 (9.5 - 22.4)	-26.3 (-34.4 - -17.9)
deaths	7.4 (1.4 - 15.1)	7.3 (1.4 - 14.6)	-0.1 (-1.1 - 0.8)
life-years lost	530.6 (97.9 - 1085.3)	525.6 (98.7 - 1053.7)	-5.0 (-78.3 - 58.6)
cost	13672.3 (7286.9 - 22370.9)	14090.7 (8344.4 - 21727.0)	418.4 (-8064.8 - 9011.5)

For peer review only - http://bmjopen.bmj.com/site/about/guidelines.xhtml

Age 5-14 years

Table A21 Ethiopia

Quantity per 100 children with presumptive TB (unless stated):	Standard of care	Intervention	Difference
children with true TB	45.5 (8.7 - 85.0)	45.5 (8.7 - 85.0)	0.0 (0.0 - 0.0)
assessments	197.9 (169.6 - 225.2)	242.2 (200.8 - 281.7)	44.3 (24.8 - 62.7)
bacteriological investigations	47.1 (12.5 - 82.7)	101.9 (86.2 - 111.6)	54.8 (16.5 - 91.1)
anti-TB treatments (ATT)	29.6 (10.6 - 53.8)	42.6 (18.5 - 67.8)	13.0 (1.5 - 30.3)
ATT initiated at PHC	74.6 (63.8 - 84.3)	82.4 (71.8 - 90.2)	7.8 (2.1 - 12.9)
percent of true-positive receiving ATT	53.6 (31.9 - 72.9)	76.8 (69.4 - 83.1)	23.2 (5.1 - 45.4)
percent of ATT bacteriologically confirmed	14.5 (2.4 - 39.5)	33.7 (20.3 - 46.1)	19.2 (-5.9 - 37.4)
percent of ATT false-positive	21.6 (2.6 - 65.3)	22.4 (2.8 - 66.2)	0.8 (-2.5 - 6.2)
referrals, inc. self-referrals	23.1 (8.8 - 39.1)	13.3 (7.3 - 20.5)	-9.7 (-22.7 - 4.4)
deaths	3.4 (0.6 - 7.4)	1.9 (0.3 - 3.9)	-1.5 (-4.0 - -0.2)
life-years lost	234.0 (42.2 - 507.3)	131.2 (24.1 - 269.5)	-102.8 (-278.9 - -10.6)
cost	14407.8 (5303.7 - 29936.5)	20277.4 (8872.7 - 37127.0)	5869.6 (-6634.5 - 19361.5)

For peer review only - http://bmjopen.bmj.com/site/about/guidelines.xhtml

Table A22 Indonesia

Quantity per 100 children with presumptive TB (unless stated):	Standard of care	Intervention	Difference
children with true TB	45.5 (8.7 - 85.0)	45.5 (8.7 - 85.0)	0.0 (0.0 - 0.0)
assessments	198.8 (170.2 - 226.2)	244.3 (202.0 - 284.1)	45.5 (25.6 - 64.3)
bacteriological investigations	47.3 (12.7 - 83.3)	102.3 (86.7 - 112.2)	54.9 (16.6 - 91.6)
anti-TB treatments (ATT)	29.6 (10.5 - 54.2)	42.7 (18.5 - 68.1)	13.1 (1.4 - 30.8)
ATT initiated at PHC	77.2 (65.5 - 87.0)	85.1 (73.6 - 92.2)	7.9 (1.5 - 13.5)
percent of true-positive receiving ATT	53.6 (31.5 - 73.0)	76.9 (69.6 - 83.3)	23.3 (4.9 - 45.9)
percent of ATT bacteriologically confirmed	14.1 (2.3 - 38.3)	33.7 (20.2 - 46.1)	19.5 (-4.9 - 37.4)
percent of ATT false-positive	21.7 (2.6 - 65.3)	22.4 (2.8 - 66.3)	0.8 (-2.6 - 6.1)
referrals, inc. self-referrals	23.9 (9.1 - 40.3)	13.8 (7.5 - 21.2)	-10.1 (-23.7 - 4.3)
deaths	3.4 (0.6 - 7.4)	1.9 (0.4 - 3.9)	-1.5 (-4.1 - -0.1)
life-years lost	244.3 (44.1 - 531.9)	136.3 (25.4 - 281.0)	-108.0 (-292.8 - -10.8)
cost	11270.2 (6013.6 - 18958.7)	14987.1 (8815.0 - 23229.9)	3716.9 (-3812.4 - 10646.6)

For peer review only - http://bmjopen.bmj.com/site/about/guidelines.xhtml

Results for 5% discount rate sensitivity analysis

Age-specific results

All ages: 0-14 years

Table A23 Ethiopia

Quantity per 100 children with presumptive TB (unless stated):	Standard of care	Intervention	Difference
children with true TB	45.5 (8.7 - 85.0)	45.5 (8.7 - 85.0)	0.0 (0.0 - 0.0)
assessments	201.8 (171.8 - 230.9)	246.2 (207.3 - 283.3)	44.4 (29.5 - 58.1)
bacteriological investigations	30.7 (8.7 - 57.5)	102.3 (86.8 - 112.0)	71.7 (41.5 - 96.3)
anti-TB treatments (ATT)	32.2 (13.2 - 54.5)	40.3 (17.6 - 64.4)	8.1 (0.6 - 20.3)
ATT initiated at PHC	71.8 (62.3 - 79.6)	81.9 (71.6 - 89.5)	10.1 (5.8 - 14.2)
percent of true-positive receiving ATT	58.3 (43.0 - 71.1)	73.0 (66.7 - 78.8)	14.7 (2.8 - 30.5)
percent of ATT bacteriologically confirmed	8.0 (1.7 - 19.8)	32.8 (20.7 - 44.1)	24.8 (10.6 - 37.8)
percent of ATT false-positive	21.9 (2.8 - 64.6)	21.9 (2.9 - 64.9)	0.0 (-3.0 - 4.0)
referrals, inc. self-referrals	29.5 (17.0 - 42.9)	13.8 (8.0 - 21.0)	-15.6 (-25.8 - -4.9)
deaths	4.9 (0.9 - 10.0)	3.9 (0.7 - 8.3)	-1.0 (-2.8 - -0.1)
life-years lost	89.0 (16.5 - 181.7)	71.3 (12.9 - 149.9)	-17.7 (-49.8 - -1.1)
cost	15729.4 (6368.3 - 31027.5)	19297.7 (8413.8 - 35444.7)	3568.3 (-8472.2 - 16311.6)

For peer review only

Table A24 Indonesia

Quantity per 100 children with presumptive TB (unless stated):	Standard of care	Intervention	Difference
children with true TB	45.5 (8.7 - 85.0)	45.5 (8.7 - 85.0)	0.0 (0.0 - 0.0)
assessments	204.2 (173.4 - 233.5)	249.9 (211.2 - 286.5)	45.7 (31.9 - 58.0)
bacteriological investigations	24.7 (7.8 - 43.2)	103.0 (87.5 - 112.6)	78.2 (54.5 - 98.4)
anti-TB treatments (ATT)	33.3 (14.1 - 55.3)	39.5 (17.1 - 63.3)	6.2 (0.1 - 15.2)
ATT initiated at PHC	73.0 (63.2 - 80.3)	84.4 (73.2 - 91.2)	11.3 (7.1 - 15.4)
percent of true-positive receiving ATT	60.3 (48.2 - 71.4)	71.8 (65.9 - 77.3)	11.5 (1.8 - 23.1)
percent of ATT bacteriologically confirmed	5.9 (1.4 - 12.9)	32.5 (20.9 - 43.4)	26.6 (14.9 - 38.2)
percent of ATT false-positive	22.0 (2.8 - 65.1)	21.8 (2.9 - 64.6)	-0.3 (-3.5 - 3.5)
referrals, inc. self-referrals	33.0 (21.5 - 45.5)	14.5 (8.6 - 21.7)	-18.4 (-27.6 - -9.6)
deaths	5.4 (1.0 - 10.9)	4.7 (0.9 - 9.3)	-0.8 (-2.2 - 0.0)
life-years lost	101.1 (19.2 - 202.4)	86.9 (16.2 - 172.7)	-14.2 (-40.3 - 0.2)
cost	12508.1 (7056.4 - 20279.0)	14525.7 (8603.6 - 22403.0)	2017.6 (-5421.3 - 9470.6)

For peer review only - http://bmjopen.bmj.com/site/about/guidelines.xhtml

Age 0-4 years

Table A25 Ethiopia

Quantity per 100 children with presumptive TB (unless stated):	Standard of care	Intervention	Difference
children with true TB	45.5 (8.7 - 85.0)	45.5 (8.7 - 85.0)	0.0 (0.0 - 0.0)
assessments	208.1 (172.8 - 241.7)	252.9 (217.5 - 286.1)	44.7 (32.9 - 55.1)
bacteriological investigations	3.4 (2.1 - 4.9)	103.1 (87.6 - 112.5)	99.8 (84.3 - 109.1)
anti-TB treatments (ATT)	36.6 (15.3 - 59.7)	36.4 (15.2 - 59.3)	-0.2 (-2.8 - 2.7)
ATT initiated at PHC	68.3 (59.6 - 75.7)	81.0 (70.9 - 88.4)	12.7 (8.0 - 17.4)
percent of true-positive receiving ATT	66.2 (55.9 - 75.4)	66.8 (58.4 - 74.6)	0.7 (-3.4 - 5.1)
percent of ATT bacteriologically confirmed	0.6 (0.3 - 1.0)	31.4 (20.4 - 42.2)	30.8 (19.9 - 41.4)
percent of ATT false-positive	21.7 (2.4 - 65.8)	20.7 (2.4 - 63.4)	-1.0 (-4.2 - 1.9)
referrals, inc. self-referrals	40.1 (28.3 - 51.7)	14.7 (9.1 - 21.8)	-25.4 (-33.2 - -17.4)
deaths	7.5 (1.4 - 15.2)	7.3 (1.4 - 14.7)	-0.1 (-1.2 - 0.7)
life-years lost	135.1 (24.9 - 274.6)	132.8 (24.6 - 266.1)	-2.3 (-21.0 - 13.1)
cost	17934.1 (7124.0 - 35159.0)	17667.9 (7614.0 - 32685.5)	-266.2 (-13326.6 - 12081.4)

For peer review only

Table A26 Indonesia

Quantity per 100 children with presumptive TB (unless stated):	Standard of care	Intervention	Difference
children with true TB	45.5 (8.7 - 85.0)	45.5 (8.7 - 85.0)	0.0 (0.0 - 0.0)
assessments	209.4 (173.8 - 242.9)	255.2 (219.1 - 289.2)	45.9 (33.8 - 56.0)
bacteriological investigations	3.4 (2.1 - 5.0)	103.6 (88.2 - 113.0)	100.2 (84.9 - 109.5)
anti-TB treatments (ATT)	36.8 (15.4 - 60.4)	36.5 (15.2 - 59.6)	-0.3 (-3.0 - 2.4)
ATT initiated at PHC	70.1 (61.1 - 77.4)	83.6 (72.6 - 90.3)	13.4 (8.6 - 18.1)
percent of true-positive receiving ATT	66.6 (56.3 - 76.0)	67.0 (58.5 - 74.8)	0.4 (-3.7 - 4.8)
percent of ATT bacteriologically confirmed	0.6 (0.3 - 1.0)	31.4 (20.4 - 42.1)	30.8 (20.0 - 41.3)
percent of ATT false-positive	21.8 (2.4 - 65.8)	20.7 (2.4 - 63.7)	-1.1 (-4.3 - 1.9)
referrals, inc. self-referrals	41.5 (29.2 - 53.1)	15.3 (9.5 - 22.4)	-26.3 (-34.4 - -17.9)
deaths	7.4 (1.4 - 15.1)	7.3 (1.4 - 14.6)	-0.1 (-1.1 - 0.8)
life-years lost	136.9 (25.3 - 280.0)	135.6 (25.5 - 271.9)	-1.3 (-20.2 - 15.1)
cost	13672.3 (7286.9 - 22370.9)	14090.7 (8344.4 - 21727.0)	418.4 (-8064.8 - 9011.5)

For peer review only - http://bmjopen.bmj.com/site/about/guidelines.xhtml

Age 5-14 years

Table A27 Ethiopia

Quantity per 100 children with presumptive TB (unless stated):	Standard of care	Intervention	Difference
children with true TB	45.5 (8.7 - 85.0)	45.5 (8.7 - 85.0)	0.0 (0.0 - 0.0)
assessments	197.9 (169.6 - 225.2)	242.2 (200.8 - 281.7)	44.3 (24.8 - 62.7)
bacteriological investigations	47.1 (12.5 - 82.7)	101.9 (86.2 - 111.6)	54.8 (16.5 - 91.1)
anti-TB treatments (ATT)	29.6 (10.6 - 53.8)	42.6 (18.5 - 67.8)	13.0 (1.5 - 30.3)
ATT initiated at PHC	74.6 (63.8 - 84.3)	82.4 (71.8 - 90.2)	7.8 (2.1 - 12.9)
percent of true-positive receiving ATT	53.6 (31.9 - 72.9)	76.8 (69.4 - 83.1)	23.2 (5.1 - 45.4)
percent of ATT bacteriologically confirmed	14.5 (2.4 - 39.5)	33.7 (20.3 - 46.1)	19.2 (-5.9 - 37.4)
percent of ATT false-positive	21.6 (2.6 - 65.3)	22.4 (2.8 - 66.2)	0.8 (-2.5 - 6.2)
referrals, inc. self-referrals	23.1 (8.8 - 39.1)	13.3 (7.3 - 20.5)	-9.7 (-22.7 - 4.4)
deaths	3.4 (0.6 - 7.4)	1.9 (0.3 - 3.9)	-1.5 (-4.0 - -0.2)
life-years lost	61.4 (11.1 - 133.2)	34.5 (6.3 - 70.8)	-27.0 (-73.2 - -2.8)
cost	14407.8 (5303.7 - 29936.5)	20277.4 (8872.7 - 37127.0)	5869.6 (-6634.5 - 19361.5)

For peer review only

Table A28 Indonesia

Quantity per 100 children with presumptive TB (unless stated):	Standard of care	Intervention	Difference
children with true TB	45.5 (8.7 - 85.0)	45.5 (8.7 - 85.0)	0.0 (0.0 - 0.0)
assessments	198.8 (170.2 - 226.2)	244.3 (202.0 - 284.1)	45.5 (25.6 - 64.3)
bacteriological investigations	47.3 (12.7 - 83.3)	102.3 (86.7 - 112.2)	54.9 (16.6 - 91.6)
anti-TB treatments (ATT)	29.6 (10.5 - 54.2)	42.7 (18.5 - 68.1)	13.1 (1.4 - 30.8)
ATT initiated at PHC	77.2 (65.5 - 87.0)	85.1 (73.6 - 92.2)	7.9 (1.5 - 13.5)
percent of true-positive receiving ATT	53.6 (31.5 - 73.0)	76.9 (69.6 - 83.3)	23.3 (4.9 - 45.9)
percent of ATT bacteriologically confirmed	14.1 (2.3 - 38.3)	33.7 (20.2 - 46.1)	19.5 (-4.9 - 37.4)
percent of ATT false-positive	21.7 (2.6 - 65.3)	22.4 (2.8 - 66.3)	0.8 (-2.6 - 6.1)
referrals, inc. self-referrals	23.9 (9.1 - 40.3)	13.8 (7.5 - 21.2)	-10.1 (-23.7 - 4.3)
deaths	3.4 (0.6 - 7.4)	1.9 (0.4 - 3.9)	-1.5 (-4.1 - -0.1)
life-years lost	63.0 (11.4 - 137.2)	35.2 (6.5 - 72.5)	-27.9 (-75.5 - -2.8)
cost	11270.2 (6013.6 - 18958.7)	14987.1 (8815.0 - 23229.9)	3716.9 (-3812.4 - 10646.6)

For peer review only - http://bmjopen.bmj.com/site/about/guidelines.xhtml

Comparison of ICERs for sensitivity analyses

Table A29 ICERs by sensitivity analysis for each country

scenario	Ethiopia	Indonesia
basecase	132.2	93.8
Xpert SOC	137.8	114.8
Low prevalence	178.1	150.4
0% discount rate	54.8	38.3
5% discount rate	199.3	142.2

For peer review only

For peer review only - http://bmjopen.bmj.com/site/about/guidelines.xhtml

Xpert on stool to diagnose tuberculosis in children is cost-effective in Ethiopia and Indonesia: a model-based cost-effectiveness analysis

Appendix 4: Consolidated Health Economic Evaluation Reporting Standards (CHEERS) checklist – Items to include when reporting economic evaluations of health interventions

Section/item	Item No	Recommendation	Reported on page No/ line No
**Title and abstract**			
Title	1	Identify the study as an economic evaluation or use more specific terms such as "cost-effectiveness analysis", and describe the interventions compared.	page 1, line 1 to 2
Abstract	2	Provide a structured summary of objectives, perspective, setting, methods (including study design and inputs), results (including base case and uncertainty analyses), and conclusions.	page 2, line 1 to 29
**Introduction**			
Background and objectives	3	Provide an explicit statement of the broader context for the study.	page 3, line 27 to 33
		Present the study question and its relevance for health policy or practice decisions.	page 3, line 27 to 33
**Methods**			
Target population and subgroups	4	Describe characteristics of the base case population and subgroups analysed, including why they were chosen.	page 4, line 1 to 13; page 5, line 1 to 9
Setting and location	5	State relevant aspects of the system(s) in which the decision(s) need(s) to be made.	page 4, line 1 to 33
Study perspective	6	Describe the perspective of the study and relate this to the costs being evaluated.	page 5, line 34 to 37
Comparators	7	Describe the interventions or strategies being compared and state why they were chosen.	page 3, line 13 to 33; page 4, line 15 to 27
Time horizon	8	State the time horizon(s) over which costs and consequences are being evaluated and say why appropriate.	page 5, line 37; page 6, line 18 to 21
Discount rate	9	Report the choice of discount rate(s) used for costs and outcomes and say why appropriate.	page 6, line 32 to 33
Choice of health outcomes	10	Describe what outcomes were used as the measure(s) of benefit in the evaluation and their relevance for the type of analysis performed.	page 6, line 23 to 27;
Measurement of effectiveness	11a	*Single study-based estimates:* Describe fully the design features of the single effectiveness study and why the single study was a sufficient source of clinical effectiveness data.	not applicable
	11b	*Synthesis-based estimates*: Describe fully the methods used for identification of included studies and synthesis of clinical effectiveness data.	page 5, line 14 to 31; page 7, line 2 to 9; Table 1; Appendix 2a
Measurement and valuation of preference based outcomes	12	If applicable, describe the population and methods used to elicit preferences for outcomes.	not applicable
Estimating resources and costs	13a	*Single study-based economic evaluation:* Describe approaches used to estimate resource use associated with the alternative interventions. Describe primary or	not applicable

For peer review only

Section/item	Item No	Recommendation	Reported on page No/ line No
		secondary research methods for valuing each resource item in terms of its unit cost. Describe any adjustments made to approximate to opportunity costs.	
	13b	*Model-based economic evaluation:* Describe approaches and data sources used to estimate resource use associated with model health states. Describe primary or secondary research methods for valuing each resource item in terms of its unit cost. Describe any adjustments made to approximate to opportunity costs.	page 5, line 34 to page 6, line 16; Table 2; Appendix 2b
Currency, price date, and conversion	14	Report the dates of the estimated resource quantities and unit costs. Describe methods for adjusting estimated unit costs to the year of reported costs if necessary. Describe methods for converting costs into a common currency base and the exchange rate.	page 5 line 34 to 37; Appendix 2b
Choice of model	15	Describe and give reasons for the specific type of decision-analytical model used. Providing a figure to show model structure is strongly recommended.	page 5, line 2 to 9; page 14, figure 1; Appendix 2a, figure 1
Assumptions	16	Describe all structural or other assumptions underpinning the decision-analytical model.	page 4, line 3 to 40; page 5, line 2 to 9; page 14, figure 1; Appendix 2a, figure 1
Analytical methods	17	Describe all analytical methods supporting the evaluation. This could include methods for dealing with skewed, missing, or censored data; extrapolation methods; methods for pooling data; approaches to validate or make adjustments (such as half cycle corrections) to a model; and methods for handling population heterogeneity and uncertainty.	page 6, line 23 to 34
**Results**			
Study parameters	18	Report the values, ranges, references, and, if used, probability distributions for all parameters. Report reasons or sources for distributions used to represent uncertainty where appropriate. Providing a table to show the input values is strongly recommended.	page 17, table 1; page 20, table 2: Appendix 2a; Appendix 2b
Incremental costs and outcomes	19	For each intervention, report mean values for the main categories of estimated costs and outcomes of interest, as well as mean differences between the comparator groups. If applicable, report incremental cost-effectiveness ratios.	page 7, line 29 to 32; page 21, table 3; Appendix 3
Characterising uncertainty	20a	*Single study-based economic evaluation:* Describe the effects of sampling uncertainty for the estimated incremental cost and incremental effectiveness parameters, together with the impact of methodological assumptions (such as discount rate, study perspective).	not applicable
	20b	*Model-based economic evaluation:* Describe the effects on the results of uncertainty for all input parameters, and uncertainty related to the structure of the model and assumptions.	page 7, line 34 to page 8, line 6; page 15, figure 2: page 16, figure 3; Appendix 3
Characterising heterogeneity	21	If applicable, report differences in costs, outcomes, or cost-effectiveness that can be explained by variations between subgroups of patients with different baseline characteristics or other observed variability in effects that are not reducible by more information.	page 7, line 11 to 32; Appendix 3
**Discussion**			

For peer review only

Section/item	Item No	Recommendation	Reported on page No/ line No
Study findings, limitations, generalisability, and current knowledge	22	Summarise key study findings and describe how they support the conclusions reached. Discuss limitations and the generalisability of the findings and how the findings fit with current knowledge.	page 8, line 9 to page 10, line 7
**Other**			
Source of funding	23	Describe how the study was funded and the role of the funder in the identification, design, conduct, and reporting of the analysis. Describe other non-monetary sources of support.	page 11, line 18 to 21; Information provided via the submission system
Conflicts of interest	24	Describe any potential for conflict of interest of study contributors in accordance with journal policy. In the absence of a journal policy, we recommend authors comply with International Committee of Medical Journal Editors recommendations.	page 11, line 16; Information provided via the submission system

For consistency, the CHEERS statement checklist format is based on the format of the CONSORT statement checklist

For peer review only

BMJ Open

Xpert Ultra stool testing to diagnose tuberculosis in children in Ethiopia and Indonesia: a model-based cost-effectiveness analysis.

Journal:	*BMJ Open*
Manuscript ID	bmjopen-2021-058388.R1
Article Type:	Original research
Date Submitted by the Author:	27-Apr-2022
Complete List of Authors:	Mafirakureva, Nyashadzaishe; The University of Sheffield, ScHARR<br>Klinkenberg, Eveline; Connect TB; Amsterdam University Medical Center, Global Health and Amsterdam Institute for Global Health and Development<br>Spruijt, Ineke; KNCV Tuberculosis Foundation<br>Levy, Jens; KNCV Tuberculosis Foundation<br>Shaweno, Debebe; The University of Sheffield, ScHARR<br>de Haas, Petra; KNCV Tuberculosis Foundation<br>Kaswandani, Nastiti; University of Indonesia Faculty of Medicine, Pediatric Department, RSCM Hospital<br>Bedru, Ahmed; KNCV Tuberculosis Foundation<br>Triasih, Rina; Universitas Gadjah Mada Fakultas Kedokteran, Department of Paediatrics<br>Gebremichel, Melaku; KNCV Tuberculosis Foundation<br>Dodd, PJ; The University of Sheffield, ScHARR<br>Tiemersma, Everdina; KNCV Tuberculosis Foundation, Technical Division
**Primary Subject Heading**:	Health economics
Secondary Subject Heading:	Epidemiology, Global health, Health policy, Infectious diseases, Paediatrics
Keywords:	Health economics < HEALTH SERVICES ADMINISTRATION & MANAGEMENT, Tuberculosis < INFECTIOUS DISEASES, EPIDEMIOLOGY, HEALTH ECONOMICS, INFECTIOUS DISEASES, Paediatric infectious disease & immunisation < PAEDIATRICS

SCHOLARONE™
Manuscripts

I, the Submitting Author has the right to grant and does grant on behalf of all authors of the Work (as defined in the below author licence), an exclusive licence and/or a non-exclusive licence for contributions from authors who are: i) UK Crown employees; ii) where BMJ has agreed a CC-BY licence shall apply, and/or iii) in accordance with the terms applicable for US Federal Government officers or employees acting as part of their official duties; on a worldwide, perpetual, irrevocable, royalty-free basis to BMJ Publishing Group Ltd ("BMJ") its licensees and where the relevant Journal is co-owned by BMJ to the co-owners of the Journal, to publish the Work in this journal and any other BMJ products and to exploit all rights, as set out in our licence.

The Submitting Author accepts and understands that any supply made under these terms is made by BMJ to the Submitting Author unless you are acting as an employee on behalf of your employer or a postgraduate student of an affiliated institution which is paying any applicable article publishing charge ("APC") for Open Access articles. Where the Submitting Author wishes to make the Work available on an Open Access basis (and intends to pay the relevant APC), the terms of reuse of such Open Access shall be governed by a Creative Commons licence – details of these licences and which Creative Commons licence will apply to this Work are set out in our licence referred to above.

Other than as permitted in any relevant BMJ Author's Self Archiving Policies, I confirm this Work has not been accepted for publication elsewhere, is not being considered for publication elsewhere and does not duplicate material already published. I confirm all authors consent to publication of this Work and authorise the granting of this licence.

For peer review only

Xpert Ultra stool testing to diagnose tuberculosis in children in Ethiopia and Indonesia: a model-based cost-effectiveness analysis.

Authors

Nyashadzaishe Mafirakureva: n.mafirakureva@sheffield.ac.uk[1]

Eveline Klinkenberg: eveline@connecttb.org[2,3]

Ineke Spruijt: ineke.spruijt@kncvtbc.org[4]

Jens Levy: jens.levy@kncvtbc.org[4]

Debebe S Adewo: d.shaweno@sheffield.ac.uk[1]

Petra de Haas: Petra.deHaas@kncvtbc.org[4]

Nastiti Kaswandani: nkaswandani@ikafkui.net[5]

Ahmed Bedru: ahmed.bedru@kncvtbc.org[6]

Rina Triasih: rina_triasih@yahoo.com[7]

Melaku Gebremichel: melaku.gebremichel@kncvtbc.org[6]

Peter J Dodd: p.j.dodd@sheffield.ac.uk[1]

Everdina W Tiemersma: edine.tiemersma@kncvtbc.org [4]

Affiliations

[1] School of Health and Related Research, University of Sheffield, Sheffield, UK

[2] Independent consultant, Connect TB, The Hague, The Netherlands

[3] Department of Global Health and Amsterdam Institute for Global Health and Development, Amsterdam University Medical Centers, Amsterdam, The Netherlands.

[4] KNCV Tuberculosis Foundation, Maanweg 174, The Hague, The Netherlands

[5] Paediatric Department, RSCM Hospital, Faculty of Medicine, University of Indonesia, Jakarta, Indonesia

[6] KNCV Tuberculosis Foundation, Addis Ababa, Ethiopia

[7] Department of Paediatrics, Faculty of Medicine, Universitas Gadjah Mada/Dr. Sardjito Hospital, Yogyakarta, Indonesia.

Corresponding author: Nyashadzaishe Mafirakureva: n.mafirakureva@sheffield.ac.uk

Word count: ~ 660+1520+657+1244 = 4081

Keywords: paediatric TB, cost-effectiveness analysis, systematic review

For peer review only

Abstract

Objectives

The World Health Organization currently recommends stool testing using on GeneXpert MTB/Rif (Xpert) for the diagnosis of paediatric tuberculosis (TB). The simple one-step (SOS) stool method enables processing for Xpert testing at the primary healthcare (PHC) level. We modelled the impact and cost-effectiveness of implementing the SOS stool method at PHC for the diagnosis of paediatric TB in Ethiopia and Indonesia, compared to standard of care.

Setting

All children (age <15 years) presenting with presumptive TB at primary healthcare or hospital level in Ethiopia and Indonesia.

Primary outcome

Cost-effectiveness estimated as incremental costs compared with incremental disability-adjusted life-years saved.

Methods

Decision tree modelling was used to represent pathways of patient care and referral. We based model parameters on ongoing studies and surveillance, systematic literature review, and expert opinion. We estimated costs using data available publicly and in-country experts. Health outcomes were based on modelled mortality and discounted life-years lost.

Results

The intervention increased the sensitivity of TB diagnosis by 19-25% in both countries leading to a 14-20% relative reduction in mortality. Under the intervention, fewer children seeking care at PHC were referred (or self-referred) to higher levels of care; the number of children initiating anti-TB treatment (ATT) increased by 18-25%; and more children (85%) initiated ATT at PHC level. Costs increased under the intervention compared to a base-case using smear microscopy in the SOC resulting in incremental cost-effectiveness ratios of $132 and $94 per DALY averted in Ethiopia and Indonesia, respectively. At a cost-effectiveness threshold of 0.5xGDP per capita, the projected probability of the intervention being cost-effective in Ethiopia and Indonesia was 87% and 96%, respectively. The intervention remained cost-effective under sensitivity analyses.

Conclusions

The addition of the SOS stool method to national algorithms for diagnosing TB in children is likely to be cost-effective in both Ethiopia and Indonesia.

For peer review only

For peer review only - http://bmjopen.bmj.com/site/about/guidelines.xhtml

Strengths and limitations of this study

- The first study to evaluate the impact and cost-effectiveness of including the SOS method to the existing national algorithms for diagnosing TB in children.
- The evaluation used a systematic literature review to inform model parameters.
- The analysis is based on two very diverse settings and is likely to have global relevance to countries with high TB burden.
- Limited availability of local data to inform some important parameters, including referral rates and primary cost data.
- Patient costs were not included, meaning any patient benefits from reduced referrals were not captured.

For peer review only

For peer review only - http://bmjopen.bmj.com/site/about/guidelines.xhtml

Background

It is estimated that in 2018, around 1.1 million children below 15 years of age fell ill from tuberculosis (TB).[1] In the same year, 250,000 children died of TB, mostly because TB was not diagnosed or was diagnosed too late. It is estimated that 55% of TB cases are missed, particularly in the youngest age group.[2] TB in children presents with nonspecific signs and symptoms, and *M. tuberculosis* bacilli are usually not detected.[2] Partly, this is because the main specimen used for diagnosing pulmonary TB is sputum, which is challenging to obtain, especially from young children. Therefore, (semi-)invasive methods such as nasogastric aspiration and sputum induction are often required. These methods are painful and stressful for children and care givers and sometimes require hospitalization. Moreover, not all primary health care (PHC) facilities in TB endemic areas, where parents with children usually first seek care, have facilities to perform these procedures. Alternative, non-invasive specimens, such as stool, can be used for the diagnosis of TB in children using Xpert MTB/RIF (Xpert) technology.[3, 4]

Since January 2020, WHO recommends Xpert testing of stool specimens as a primary diagnostic test for TB in children with signs and symptoms of pulmonary TB.[5] This recommendation has the potential to improve bacteriological confirmation of TB in children, and is increasingly being adopted by national TB programs, for example, Ethiopia[6]. However, to make the test fit for use at the PHC level, a simple, non-hazardous, and cheap method to process stool for Xpert testing was needed. Several centrifuge-free methods have been proposed,[7-10] but all need additional equipment and/or consumables which may not be (easily) available in peripheral lower-level public health laboratories. Therefore, we developed a simple one-step (SOS) stool processing method for Xpert testing. This method can be applied in any laboratory with an Xpert machine, as it does not require additional equipment or consumables than those delivered routinely with the Xpert cartridges.[11] Limited preliminary data suggest that Xpert Ultra on stool samples processed using the SOS stool method has higher sensitivity compared to other stool processing methods. Available systematic reviews on the diagnostic accuracy of stool testing have reported sensitivity of 50-67% [3, 4, 12]. The variation in sensitivity estimates may be explained by a variation in studies included, and thus, variation in study populations, stool processing methods, and reference standards (sputum culture[4, 12] or a combination of sputum culture and sputum Xpert[3]) included in each review. This method has the potential to significantly impact the number of children receiving a bacteriological confirmation of TB, including rifampicin resistance profile. Consequently, more paediatric TB patients can be diagnosed at lower levels of the healthcare system, with a reduced time to diagnosis because no referrals are needed to higher levels of healthcare as well as reduced costs for both the healthcare system and families.

However, evidence on the impact and cost-effectiveness of the SOS Xpert stool processing method is needed to inform implementation and scale-up in routine healthcare systems. Therefore, we modelled the potential impact and cost-effectiveness of bringing this test to the lower healthcare level where children present first, focusing on Ethiopia and Indonesia. Specifically, we aimed to estimate the impact of implementing the Xpert stool test for the diagnosis of pulmonary TB among children at the PHC level on rates of bacteriological confirmation of TB and mortality among children, the costs to the healthcare provider, and the incremental cost-effectiveness of the approach. Ethiopia and Indonesia are currently among the 30 high TB burden countries in the world[13]. The incidence of TB was estimated to be 301 (276-328) per 100,000 population with 824 000 (755 000-897 000) people falling ill with TB in 2020 in Indonesia. In Ethiopia, the incidence of TB was estimated to be 132 (92-178) per 100,000 population with 151 000 (106 000-205 000) people falling ill with TB in 2020. While TB diagnosis and treatment in Ethiopia largely occur in the public sector, the private sector plays a substantial role in Indonesia.

For peer review only - http://bmjopen.bmj.com/site/about/guidelines.xhtml

Methods

Conceptual approach

We developed a conceptual model of care pathways for children (age <15 years) with presumptive TB presenting at either PHC facilities or hospitals, referral (including self-referral) between these levels, and clinical and bacteriological assessment and re-assessment (see Figure 1). This description of stages in patient care was based on national guidelines and local knowledge, and were broad enough to capture pathways in both Ethiopia and Indonesia, and incorporate the SOC as well as the intervention.

We defined presumptive TB patients, following clinical guidelines in both settings, as children with signs or symptoms suggestive of pulmonary TB (at least one) such as persistent cough, unexplained fever and/or night sweats, poor weight gain or weight loss, reduced playfulness or malaise, history of contact with a TB patient, or enlarged lymph nodes in the neck (only Ethiopia).

Under the SOC, national guidelines in both countries recommend the usage of GeneXpert[6, 14]. However, in both countries, sputum smear microscopy is allowed for diagnosis if the PHC has no access to GeneXpert. Despite this recommendation, in both countries, most PHC units do not have access to a GeneXpert machine, and therefore use sputum smear microscopy for the diagnosis of paediatric TB. For diagnosis of paediatric TB, in the primary (base-case) analyses, we assumed that sputum-smear microscopy was the bacteriological test used at PHC in SOC in both Ethiopia and Indonesia. As sensitivity analyses, we considered alternate scenarios with Xpert used for bacteriological testing for sputum in SOC.

The intervention was modelled as implementing the simple stool Xpert testing method at PHC and hospital level. Thus, considering spontaneous sputum expectoration to be limiting in obtaining a test result under SOC, we conceptualized the intervention as increasing the fraction of children with a bacteriological test result at both the primary and higher healthcare level.

We assumed that children with a negative bacteriological test under the intervention would receive clinical assessments for TB while only a small proportion would get clinical assessments under SOC. A clinical diagnosis can be made based on TB-suggestive signs or symptoms, X-ray results, and tuberculin skin test (Indonesia only) or contact history with a TB patient. Indonesian referral centres that do not have access to X-ray and/or tuberculosis TB skin tests use a score chart for clinical diagnosis.

Systematic review data on the sensitivity of stool-based diagnostics for identifying TB in children, indicate sensitivity of 50-67% [3, 4, 12] in children with bacteriologically confirmed TB, but very poor sensitivity (2-6%) in clinically diagnosed TB.[3, 4] We therefore assumed that stool testing would only detect a proportion of those children who would be bacteriologically positive under ideal circumstances. The accuracy of Xpert testing on stool using the SOS method was modelled based on a systematic review[4] which reported pooled sensitivity and specificity of stool Xpert of 57.1% (95% CI 51.5-62.7%) and 98.1% (CI 97.5-98.6%), respectively, compared to culture on a respiratory sample as the reference standard. The intervention was to reduce mortality through higher sensitivity for detecting TB, and to reduce referrals and re-assessments.

For peer review only - http://bmjopen.bmj.com/site/about/guidelines.xhtml

Modelling approach

The pathway of care shown in Figure 1 was coded into a decision tree using the HEdtree package in R.[15, 16] Referral endpoints from PHC level were modelled by adding an identical hospital care pathway to follow the three paths for referral from PHC level. All care outcomes were extended to either death or survival. The probability of children following different pathways through the tree was assumed to depend on: true TB status and age (0-4 years or 5-14 years). Mortality risk from TB by age group and anti-TB treatment (ATT) status was modelled using a published approach,[17] using case-fatality ratios (CFRs) based on systematic review data.[18] We neglected mortality in children who were truly negative for TB. We did not model drug-resistant TB or HIV status.

All parameters in the model were treated as uncertain and following specified distributions. All results were based on applying the model to calculate mean outcomes from the tree for each of 10,000 samples from these parameter distributions.

Literature review and parameterization

To inform the parameters needed in the decision tree model (Figure 1, parameters noted as such in Table 1), we followed a three-step data collection process. Firstly, we reviewed data from ongoing studies in Ethiopia[11] and Indonesia (Kaswandani N, Tiemersma EW, Janiar H, et al. Xpert MTB/RIF testing on stools using simple pre-processing methods to diagnose childhood pulmonary tuberculosis in Indonesia. 2019). Secondly, we systematically searched peer-reviewed literature for parameters not available from country study data. In brief, initially, systematic reviews on TB in children were sought by a search of PubMed including the terms “systematic review”, “meta-analysis”, “tuberculosis” and “children” on 19 June 2020. Subsequently, we constructed pooled estimates from primary literature, published from 2010 – present, about TB diagnostic testing in infants and children, including health care seeking and health care cascade with a focus on Ethiopia and Indonesia. For this, a systematic search strategy was developed by an information specialist combining free-text and thesaurus searching. Except for searches specifically addressing Indonesia/Ethiopia, we excluded case reports, non-English and non-human studies, and papers with terms for BCG, latent tuberculosis, IGRA and tuberculin skin test in titles because of their relevance to TB infection, not active pulmonary TB. Searches were conducted between 19 and 26 October 2020. Finally, to inform remaining parameters, we sought opinion from TB experts from each country (authors AB and MG for Ethiopia and NK and RT for Indonesia) in an iterative process using a questionnaire, and remote workshops to explain the model and focus on parameters identified as influential by one-way sensitivity analysis. More details are provided in Appendix 1 (Literature search) and Appendix 2a (Model parameter estimation).

Cost parameters and health economic approach

We collected costs (reported in 2019 USD) from the healthcare provider’s perspective and adjusted historical costs for inflation to 2019 prices using relevant GDP deflators.[19] We transferred costs from other countries to Ethiopia and Indonesia by applying relevant purchasing power parity conversion factors.[20] All costs were assumed to accrue in the present, with no discounting applied.

For peer review only

We assumed the cost for the initial TB assessment at the PHC was equivalent to the country-specific cost of two outpatient visits (or a single outpatient visit for re-assessment) to a health centre (health centre with no beds from WHO-CHOICE estimates[21]). Similar assumptions were used for hospital assessment and re-assessment with the corresponding WHO-CHOICE cost estimates.[21] The cost of bacteriological investigation in the SOC includes the country specific unit cost of either sputum smear microscopy (SSM)[22, 23] or Xpert, depending on availability at each level of care, adding the unit costs for collecting two sputum samples for testing with SSM or one sample for testing with Xpert. The unit costs for Xpert were estimated based on country specific data available from the OneHealth Tool (see Appendix 2b).[24] Country-specific unit costs for collecting sputum samples were not available and are based on a study done in adults from South Africa.[25] In the intervention, we applied the unit cost for collecting a single stool sample based on estimates provided by the Paediatric Operational Sustainability Expertise Exchange group.[26] Treatment cost for diagnosed TB comprises the cost of anti-tuberculosis drugs (including pyridoxine), from the Global Drug Facility,[27] the costs of follow-up visits (drug pickups or medical review) according to national TB treatment guidelines at the healthcare facilities based on WHO-CHOICE unit cost estimates, and the costs of laboratory monitoring in bacteriologically confirmed TB only (see Appendix 2b (Overview of cost parameters)).

We used a disability-adjusted life-year (DALY) framework, calculating the life-years saved over a life-time horizon with a discount rate of 3% based on United Nations Population Division country-specific life tables. A simple mean across ages included in the 0-4 and 5-14 year age groups was used, and decrements in health-related quality of life or subsequent survival were not modelled.

Metrics calculated

For every 100 children seeking care with presumptive TB in each country, we calculated the deaths, DALYs, costs, referrals, clinical assessments, bacteriological assessments, ATTs, percent of true TB receiving ATT, percent of those receiving ATT bacteriologically confirmed, percent of those receiving ATT initiated at PHC, percent of ATT that is false-positive, as well as the change in these quantities under the intervention. We report the incremental cost-effectiveness ratio (ICER). For each country, we produced plots of the cost-effectiveness plane, cost-effectiveness acceptability curve, and expected net benefit, and tornado plots illustrating the one-way sensitivity of outcomes to influential model parameters. We also undertook specific scenario analyses: 1) we considered a low TB prevalence scenario (half the base-case prevalence among presumptive TB patients); 2) we considered Xpert as the universally available bacteriological test instead of sputum-smear microscopy in SOC; 3) we considered discount rates of 0% and 5% for the life years. The results of these sensitivity analyses are included in Appendix 3 (Additional results). Results are presented following the Consolidated Health Economic Evaluation Reporting Standards Statement (Appendix 4).

Patient and Public Involvement

Study participants or the public were not involved in the design, or conduct, or reporting, or dissemination plans of our research.

For peer review only - http://bmjopen.bmj.com/site/about/guidelines.xhtml

Research ethics statement

Not applicable. This was a modelling study based on secondary data.

Results

Following our review of the literature, we developed the model parametrization shown in Table 1. The data sources and approach to synthesis for each parameter are described in detail respectively in Appendix 1 and Appendix 2a. Country-specific data was used to inform the proportion of children submitting a spontaneously expectorated sputum sample, the fraction of presumptive TB in children under 5 years, and the level of initial care-seeking at PHC. We used existing systematic reviews for the basis of parameters describing diagnostic test accuracy, our own pooled estimates of true TB prevalence among presumptive TB, the fraction of TB that is bacteriological confirmable, and the fraction of children able to spontaneously expectorate. Evidence for the accuracy of clinical diagnosis was limited, and published evidence was completely lacking for other parameters around referral and re-assessment. Hence, we based these on expert opinion. Unit costs used in the analysis are shown in Table 2.

The intervention increased the sensitivity to detect true TB by over 10 percentage points in each country and resulted in around a 4-fold increase in the proportion of TB patients diagnosed that are bacteriologically confirmed. Specificity showed little change under the intervention (<1% change). In both countries, the proportion of children referred (or self-referred) to higher levels of care after seeking care at PHC level fell by more than 2-fold. In both countries, the average total number of assessments for children with presumptive TB increased from around 2 under SOC to around 2.5 per child with the intervention, and the total number of bacteriological investigations increased more than 3-fold (Table 3).

The relative number of children initiated on ATT increased by 19-25% under the intervention. A larger fraction (~40% relative increase) of children received ATT with the intervention, and more children (~10% point increase) initiated ATT at PHC level (Table 3). Restricting to children under 5, we found bigger increases in the number of bacteriological investigations (+30-fold), and the proportion of TB cases diagnosed that are bacteriologically confirmed (+50%). We also found a larger reduction in referrals of children with presumptive TB to higher levels of care in both countries (almost 3-fold). (see Appendix 3 (Additional results).

In both countries, the increase in sensitivity of a TB diagnosis under the intervention generated a corresponding reduction in mortality: a 14%-20% relative reduction in the fraction of children with presumptive TB dying (Table 3). In both countries, costs increased under the intervention, and the base-case (using smear microscopy in the SOC) ICERs were $132/DALY averted in Ethiopia and $94/DALY averted in Indonesia (Figure 2). Restricting the analysis to children under 5 resulted in cost-savings with ICERs of -$78/DALY averted in Ethiopia and increased the ICER to $209/DALY averted in Indonesia.

Uncertainty and sensitivity analyses

Model projections showed large uncertainty (Figure 2) that included cost savings under intervention (25% of the runs for Ethiopia and 28% for Indonesia), but also some increases in mortality (1.2% of the runs for Ethiopia and 2.8% for Indonesia). At a cost-effectiveness threshold of 0.5xGDP our analysis projected a 87% probability of being cost effective in Ethiopia and a 96% probability of being cost-effective in

For peer review only - http://bmjopen.bmj.com/site/about/guidelines.xhtml

Indonesia (see Appendix 3). The corresponding probabilities for a 1xGDP threshold were 95% (Ethiopia) and 97% (Indonesia). Tornado plots (Figure 3) show that prevalence of true TB among presumptive TB, the sensitivity of stool, and the fraction of children able to expectorate were the largest drivers of uncertainty (see also Appendix 3).

Under the assumption that Xpert was used in the SOC, the ICERs were $138/DALY averted in Ethiopia and $115/DALY averted in Indonesia. Assuming half the prevalence of true TB among presumptive TB patients changed the ICERs to $178/DALY averted in Ethiopia and $150/DALY averted in Indonesia. Finally, assuming a 0% discount rate changed the ICERs to $55/DALY averted in Ethiopia and $38/DALY averted in Indonesia, whereas 5% discount rate generated ICERs of $199/DALY averted in Ethiopia and $142/DALY averted in Indonesia.

Discussion

In this modelling analysis, we found that the introduction of routine Xpert stool-based diagnostics (using the SOS method) was cost-effective in both Ethiopia and Indonesia. In the context of predominantly clinical diagnosis of TB in children, particularly among those aged <5 years, we found a 14-20% relative reduction in mortality driven by an increase in sensitivity to detect true TB. However, it is crucial that clinical assessment is still undertaken alongside negative bacteriological test results because bacteriological testing has a low negative predictive value especially in young children.[3, 4] Relying on bacteriological testing alone can reduce sensitivity to diagnose true TB and increase mortality, especially if referrals and re-assessments are common under the standard of care (data not shown). We estimated ICERs of $132 and $94 per DALY averted in the base-case analyses for Ethiopia and Indonesia, respectively. These ICERs are less than 0.5 x GDP, which has been suggested as a rule of thumb for cost-effectiveness thresholds,[28] as well as country-specific estimates of supply-side thresholds.[29, 30] The intervention would be especially cost-effective for children under 5 years of age.

Children age <5 years are at higher risk of dying from untreated TB than older children and have the greatest difficulty in spontaneously expectorating sputum. Under the intervention, we found greater increases in bacteriologically diagnosis and greater decreases in referrals in the <5 year age group (see age stratified results in Appendix 3). We found that the cost of introducing the intervention was partially offset by reduced referrals from PHC facilities to hospitals. In Ethiopia, this produced a projected cost saving in the under 5 age group, despite a slight increase in the average total number of assessments done. In taking a healthcare provider's perspective, we did not include patient costs in our analysis, but health-seeking costs are a major driver of catastrophic costs in TB.[31]

There are large uncertainties associated with many parameters describing processes and pathways for paediatric TB. We found no directly applicable estimates of rates of reassessments or (self)referral at different stages of care, and had to rely on expert opinion. Additionally, the sensitivity and specificity of clinical assessment for paediatric TB is poorly quantified in the literature. Because of this, we placed a particular emphasis on including uncertainty in results, as well as systematically exploring their sensitivity to one-way variation in parameters, and discrete alternative assumptions. For example, because our estimate of true TB prevalence among children with presumptive TB was based on data mainly from hospitals which may have higher prevalence than PHC level, we halved prevalence resulting in increased

For peer review only - http://bmjopen.bmj.com/site/about/guidelines.xhtml

ICERs by less than a factor of two without changing our qualitative conclusions. Despite these uncertainties, the intervention showed probabilities of being cost-effective > 85% in each country across a wide range of cost-effectiveness thresholds. This conclusion was also robust to assuming the SOC used Xpert rather than sputum-smear microscopy at PHC level, and to different choices of discount rate.

Some aspects were deliberately simplified or omitted in the modelling. First, we did not model HIV because paediatric HIV rates in Ethiopia and Indonesia are relatively low at 0.09% and 0.03%,[32] respectively. This may underestimate the benefit from the intervention due to underestimated TB mortality, especially if stool-based methods are more effective at diagnosing TB in children with HIV compared to sputum. Secondly, we did not model drug-resistant TB because of low rates of multidrug-resistant (MDR) TB among new TB cases (all ages) in Ethiopia (1.02% (0.49 - 1.54%)) and Indonesia (2.4% (1.8-3.3%)). This may underestimate the intervention costs since the higher fractions of cases bacteriologically confirmed via Xpert MTB/Rif mean that more MDR TB will be diagnosed and require more costly second-line treatment. Thirdly, we did not consider the private sector, which in Indonesia is substantial, and less likely to closely follow guidelines. Our intervention is conceived as being implemented in the public sector, but patients seeking care across both sectors may mean we overestimate the savings to the (public) health system from reduced referrals. Fourthly, for pragmatic reasons, country-specific primary cost analyses were not performed and additional one-off programmatic costs for widely introducing Xpert stool testing, such as costs for training and supervision, were not included in our analyses. Both countries are moving to fully replacing sputum smear microscopy by Xpert testing as the primary diagnostic for TB in all patient groups. This may increase logistical issues in both countries which need to be dealt with, such as cartridge shelf life (which is shorter for the Ultra than the G4 cartridge) and module maintenance. Lastly, we modelled the impact of making a stool Xpert-based diagnosis available at the PHC level. The analysis also assumes that all PHC facilities have access to a GeneXpert machine, either on-site or through an effective sample transportation system. Thus, until full access to Xpert testing is available, the coverage of the intervention will be limited.

Furthermore, due to the lack of data from randomized controlled trials and operational studies, we were reliant on early experience to determine acceptability and feasibility of stool-based sampling and diagnostics. Hence, difficulties in implementation that dilute effects or increase costs may be overlooked. However, the recent recommendation to use stool as a primary sample for diagnosing childhood TB[5] has generated interest in Xpert stool testing at national TB programs. Although we used an illustrative high acceptability rate for stool, this is supported by early experience from the two countries and recently published evidence[11, 33]. Apart from the SOS stool method, two other centrifuge-free stool processing methods are being developed,[9, 10] which are included in a head-to-head comparison study to compare their performance in diagnosing childhood TB against sputum or gastric aspirate culture. This project has a health economic component, estimating cost-effectiveness of the best performing method. Results of this project are expected at the end of 2021. The TB Speed decentralization operational research study will report results from use of Xpert on nasopharyngeal aspirate and stool samples at PHC level in early 2022. A small study comparing the SOS stool method to the stool processing kit involved in the head-to-head comparison study[10] concluded that taking into account the sample processing time, consumable requirements and error rates, the SOS stool method would be the method that would be best scalable in low and middle income countries.[34]

For peer review only - http://bmjopen.bmj.com/site/about/guidelines.xhtml

Additional evidence from studies and implementation is needed to inform the optimal use of new sample and diagnostic approaches for paediatric TB within real health systems. Studies to quantify referral patterns, the pathways and outcomes of individual patients, and the costs of real-world implementation would be particularly valuable. Further analyses could include context-specific operational research to help design referral systems that best utilize Xpert machines and minimize cartridge expiry, as well as budget impact analyses to help national programmes plan roll-out and seek funding. Clinical diagnosis remains an important tool for children with TB; helping clinicians diagnose TB in children without bacteriological results or with negative results should be part of intervention design and the role of clinical diagnosis in current and novel diagnostic pathways a topic for further research. The importance of clinical TB diagnosis for children limits the potential impact of bacteriological diagnostics.

Conclusion

In this modelling analysis, we projected that introduction of routine stool-based Xpert diagnostics at primary health care and hospital level would increase the proportion of bacteriologically confirmed TB in children, while reducing child mortality and life-years lost in both Ethiopia and Indonesia. Our analysis suggests that this intervention would be cost-effective in both countries.

For peer review only

Acknowledgements

We thank Anthea Sutton, an Information Specialist at the University of Sheffield, for setting up and conducting the literature database searches; Sarah van de Berg for her assistance in assessing and extracting data from the systematic reviews; Key informants for the expert-solicited parameters from Ethiopia and Indonesia; the NTP and related stakeholders, including pediatricians, scientists, technical agencies and funders implementing childhood TB projects in both countries.

Author contributions

Conceptualization: NM, EK, IS, JL, DS, PdH, PD, ET

Systematic reviewing: EK, IS, JL, ET

Modelling and quantitative analyses: NM, DS, PD

First draft: NM, EK, IS, JL, DS, PD, ET

Review of country epidemiological and health system approach: NK, AB, RT, MG

Review of approach, editing article and approval of final manuscript: all authors.

Conflicts of interest

The authors declare no conflicts of interest.

Funding / Support statement

This work was funded by the TB Modelling and Analysis Consortium (Bill and Melinda Gates Foundation, OPP1084276). PJD was supported by a fellowship from the UK Medical Research Council (MR/P022081/1); this UK funded award is part of the EDCTP2 programme supported by the European Union.

Data availability

Data and code to run these are analyses are available on reasonable request.

For peer review only

For peer review only - http://bmjopen.bmj.com/site/about/guidelines.xhtml

References

1. World Health Organization. Global tuberculosis report 2019: World Health Organization; 2019 [Available from: https://www.who.int/teams/global-tuberculosis-programme/tb-reports/global-report-2019.
2. World Health Organization. Roadmap towards ending TB in children and adolescents 2018 [Available from: https://apps.who.int/iris/bitstream/handle/10665/274374/9789241514668-eng.pdf.
3. MacLean E, Sulis G, Denkinger CM, et al. Diagnostic Accuracy of Stool Xpert MTB/RIF for Detection of Pulmonary Tuberculosis in Children: a Systematic Review and Meta-analysis. *J Clin Microbiol*. 2019;57(6):e02057-18.
4. Mesman AW, Rodriguez C, Ager E, et al. Diagnostic accuracy of molecular detection of Mycobacterium tuberculosis in pediatric stool samples: A systematic review and meta-analysis. *Tuberculosis (Edinb)*. 2019;119:101878-101878.
5. World Health Organization. Rapid Communication: Molecular assays as initial tests for the diagnosis of tuberculosis and rifampicin resistance 2020 [Available from: https://www.who.int/publications/i/item/9789240000339.
6. Ministry of Health Ethiopia. Guidelines for Clinical and Programmatic Management of TB, DR-TB and Leprosy in Ethiopia, 7th edition. Addis Ababa, Ethiopia: Ministry of Health Ethiopia; 2020.
7. Andriyoko B, Janiar H, Kusumadewi R, et al. Simple stool processing method for the diagnosis of pulmonary tuberculosis using GeneXpert MTB/RIF. *Eur Respir J*. 2018;53(3):1801832.
8. Banada PP, Naidoo U, Deshpande S, et al. A Novel Sample Processing Method for Rapid Detection of Tuberculosis in the Stool of Pediatric Patients Using the Xpert MTB/RIF Assay. *PLoS One*. 2016;11(3):e0151980-e0151980.
9. Lounnas M, Diack A, Nicol MP, et al. Laboratory development of a simple stool sample processing method diagnosis of pediatric tuberculosis using Xpert Ultra. *Tuberculosis (Edinb)*. 2020;125:102002.
10. Walters E, Scott L, Nabeta P, et al. Molecular Detection of Mycobacterium tuberculosis from Stools in Young Children by Use of a Novel Centrifugation-Free Processing Method. *J Clin Microbiol*. 2018;56(9):e00781-18.
11. de Haas P, Yenew B, Mengesha E, et al. The Simple One-Step (SOS) Stool Processing Method for Use with the Xpert MTB/RIF Assay for a Child-Friendly Diagnosis of Tuberculosis Closer to the Point of Care. *J Clin Microbiol*. 2021;59(8).
12. Gebre M, Cameron LH, Tadesse G, et al. Variable Diagnostic Performance of Stool Xpert in Pediatric Tuberculosis: A Systematic Review and Meta-analysis. *Open Forum Infect Dis*. 2021;8(8):ofaa627.
13. Organization WH. WHO releases new global lists of high-burden countries for TB, HIV-associated TB and drug-resistant TB. 2021.
14. Ministry of Health Republic of Indonesia. National guideline for TB control. Jakarta, Indonesia: Ministry of Health Republic of Indonesia; 2016.
15. Dodd PJ. HEdtree: A Package For Decision Tree Modelling 2021 [Available from: https://github.com/petedodd/HEdtree.
16. R Core Team. R: A language and environment for statistical computing. Vienna, Austria: R Foundation for Statistical Computing; 2020.
17. Dodd PJ, Yuen CM, Sismanidis C, et al. The global burden of tuberculosis mortality in children: a mathematical modelling study. *Lancet Glob Health*. 2017;5(9):e898-e906.
18. Jenkins HE, Yuen CM, Rodriguez CA, et al. Mortality in children diagnosed with tuberculosis: a systematic review and meta-analysis. *Lancet Infect Dis*. 2017;17(3):285-295.

For peer review only - http://bmjopen.bmj.com/site/about/guidelines.xhtml

19. World Bank. World Bank national accounts data, and OECD National Accounts data files. GDP deflator: The World Bank; 2021 [Available from: https://data.worldbank.org/indicator/NY.GDP.DEFL.ZS.
20. World Bank. PPP conversion factor, GDP (LCU per international $): International Comparison Program, World Bank; 2021 [Available from: https://data.worldbank.org/indicator/PA.NUS.PPP.
21. Stenberg K, Lauer JA, Gkountouras G, et al. Econometric estimation of WHO-CHOICE country-specific costs for inpatient and outpatient health service delivery. *Cost Eff Resour Alloc*. 2018;16:11-11.
22. Jarrah Z CD, Hafidz F. The cost of scaling up TB services in Indonesia.: TB CARE I – Management Sciences for Health.; 2013.
23. Tesfaye A, Fiseha D, Assefa D, et al. Modeling the patient and health system impacts of alternative xpert® MTB/RIF algorithms for the diagnosis of pulmonary tuberculosis in Addis Ababa, Ethiopia. *BMC Infect Dis*. 2017;17(1):318-318.
24. World Health Organization. OneHealth Tool Geneva, Switzerland: World Health Organization; 2020 [Available from: https://www.who.int/choice/onehealthtool/en/.
25. Peter JG, Theron G, Pooran A, et al. Comparison of two methods for acquisition of sputum samples for diagnosis of suspected tuberculosis in smear-negative or sputum-scarce people: a randomised controlled trial. *Lancet Respir Med*. 2013;1(6):471-478.
26. Paediatric Operational Sustainability Expertise Exchange group. Paediatric Operational Sustainability Expertise Exchange group (POSEE group) budgeting tools 2020 [Available from: https://www.dropbox.com/sh/rmzlqoot9m1muxe/AACOPh8SuS4WwMQD_vfN-oHCa?dl=0.
27. Stop TB Partnership | Global Drug Facility. Global Drug Facility (GDF) Medicines catalogue January 2021 2021 [Available from: http://www.stoptb.org/assets/documents/gdf/drugsupply/GDFMedicinesCatalog.pdf.
28. Chi YL, Blecher M, Chalkidou K, et al. What next after GDP-based cost-effectiveness thresholds? *Gates Open Res*. 2020;4:176-176.
29. Woods B, Revill P, Sculpher M, et al. Country-Level Cost-Effectiveness Thresholds: Initial Estimates and the Need for Further Research. *Value Health*. 2016;19(8):929-935.
30. Ochalek J, Lomas J, Claxton K. Estimating health opportunity costs in low-income and middle-income countries: a novel approach and evidence from cross-country data. *BMJ Glob Health*. 2018;3(6):e000964-e000964.
31. Tanimura T, Jaramillo E, Weil D, et al. Financial burden for tuberculosis patients in low- and middle-income countries: a systematic review. *Eur Respir J*. 2014;43(6):1763-1775.
32. Joint United Nations Programme on HIV/AIDS. HIV estimates with uncertainty bounds 1990-Present | UNAIDS. Geneva: UNAIDS2021 [Available from: http://aidsinfo.unaids.org.
33. Organization WH. Rapid communication on updated guidance on the management of tuberculosis in children and adolescents. 2021.
34. Jasumback CL, Dlamini Q, Kahari J, et al. Laboratory comparison of stool processing methods for Xpert(®) Ultra. *Public Health Action*. 2021;11(2):55-57.
35. Detjen AK, DiNardo AR, Leyden J, et al. Xpert MTB/RIF assay for the diagnosis of pulmonary tuberculosis in children: a systematic review and meta-analysis. *Lancet Respir Med*. 2015;3(6):451-61.
36. Marais BJ, Gie RP, Hesseling AC, et al. A refined symptom-based approach to diagnose pulmonary tuberculosis in children. *Pediatrics*. 2006;118(5):e1350-9.
37. Fekadu L, Hanson C, Osberg M, et al. Increasing Access to Tuberculosis Services in Ethiopia: Findings From a Patient-Pathway Analysis. *J Infect Dis*. 2017;216(suppl_7):S696-s701.
38. Surya A, Setyaningsih B, Suryani Nasution H, et al. Quality Tuberculosis Care in Indonesia: Using Patient Pathway Analysis to Optimize Public–Private Collaboration. *J Infect Dis*. 2017;216(suppl_7):S724-S732.

For peer review only - http://bmjopen.bmj.com/site/about/guidelines.xhtml

Figures legends

Figure 1. Simplified diagram of decision-analytic model showing the pathways of care for TB diagnosis and treatment. The decision tree shows children with presumptive TB presenting at either PHC facilities or hospitals where they undergo clinical evaluation with or without bacteriological testing. All children diagnosed with TB are considered for anti-tuberculosis treatment. Children with a negative bacteriological test or those not initially diagnosed with TB after clinical assessment only can be reassessed clinically. Coloured boxes depict the potential of referral to a higher-level facility and referrals (indicated by grey lines) from PHC to hospital for further assessment can occur for children without a diagnosis of TB. Each pathway extends to death or survival, however, these details are omitted here to keep the diagram simple. See Appendix 2a for more details on the pathway and parametrization of the model.

MTB: Mycobacterium tuberculosis; TB: tuberculosis; TB Tx: TB diagnosis and anti-TB treatment; PHC; primary health care.

Figure 2. Cost-effectiveness plane showing the differences in costs (y-axis) and disability-adjusted life-years (DALYs, x-axis) of using the SOS stool method for diagnosis of paediatric TB in Ethiopia (left) and Indonesia (right), compared to standard of care from 10 000 simulations. The red dot represents the mean incremental costs and DALYs.

ICER: incremental cost-effectiveness ratio; k: cost-effectiveness threshold, SoC: Standard of Care.

Figure 3. Tornado plots showing one-way sensitivity of incremental deaths (top row) and incremental costs (bottom row) to parameters for Ethiopia (left) and Indonesia (right). spont.sputo5: spontaneous sputum possible (5-14 years), p_truetb: prevalence of true TB in presumptive, r1: referral from PHC to Hospital after clinical re-assessment following bacteriological negative result, r2: referral from PHC to Hospital after initial clinical assessment without bacteriological test result, fracu5: fraction of presumptive TB under 5, c_f.phc: cost of TB treatment at PHC after clinical re-assessment, c_d.phc: cost of TB treatment at PHC after initial clinical assessment, c_a.phc: cost of clinical and bacteriological TB assessment at PHC, c_clin.h: cost of clinical TB assessment at Hospital, c_clin.phc: cost of clinical TB assessment at PHC (Only top 3 parameters on each plot defined here. Please refer to Appendix 2a, and Appendix 2b, for the rest of the parameter definitions).

For peer review only - http://bmjopen.bmj.com/site/about/guidelines.xhtml

Tables

Table 1 Table of parameters used in modelling and underlying evidence. More details on parameter distributions, parameter naming, and methods are available in Appendix 2a. C+: culture positive, CFR: case fatality rate, PHC: primary health care, PTLTFU: pre-treatment lost to follow-up, SM: smear microscopy.

DESCRIPTION	SOURCE	REFERENCES	Mean (IQR)
sensitivity of Xpert on stool in bacteriologically positive children	existing review	Mesman 2019[4]	0.571 (0.515 - 0.627)
specificity of Xpert on stool in bacteriologically positive children	existing review	Mesman 2019[4]	0.981 (0.975 - 0.986)
sensitivity of Xpert on sputum in C+	existing review	Detjen 2015[35]	0.621 (0.582 - 0.659)
specificity of Xpert on sputum in C+	existing review	Detjen 2015[35]	0.980 (0.977 - 0.984)
sensitivity of SM on sputum in C+	existing review	Detjen 2015[35]	0.257 (0.215 - 0.302)
specificity of SM on sputum in C+	existing review	Detjen 2015[35]	0.995 (0.994 - 0.997)
spontaneous sputum possible (0-4 years)	our review	see Appendix 2a	0.024 (0.020 - 0.027)
spontaneous sputum possible (5-14 years)	our review	see Appendix 2a	0.377 (0.254 - 0.512)
fraction of children bacteriologically confirmable <5 years	our review	see Appendix 2a	0.380 (0.363 - 0.397)
fraction of children bacteriologically confirmable 5-14 years	our review	see Appendix 2a	0.684 (0.659 - 0.711)
prevalence of true TB in presumptive	our review	see Appendix 2a	0.453 (0.289 - 0.607)
specificity of clinical diagnosis < 5 years	our review	Marais 2006 (see Appendix 2a)	0.928 (0.908 - 0.945)
sensitivity of clinical diagnosis < 5 years	our review	Marais 2006[36]	0.518 (0.482 - 0.554)
specificity of clinical diagnosis 5-14 years	our review	Marais 2006[36]	0.901 (0.878 - 0.921)
sensitivity of clinical diagnosis 5-14 years	our review	Marais 2006[36]	0.627 (0.592 - 0.661)

For peer review only - http://bmjopen.bmj.com/site/about/guidelines.xhtml

proportion of first care-seeking at PHC for **Ethiopia**	our review	Fekadu et al, 2017[37]	0.896 (0.777 - 0.973)
proportion of first care-seeking at PHC for **Indonesia**	our review	Surya et al, 2017[38]	0.928 (0.801 - 0.992)
fraction of presumptive TB under 5 years **Ethiopia**	routine data	fraction of WHO TB < 5	0.371 (0.300 - 0.447)
fraction of presumptive TB under 5 years **Indonesia**	routine data	fraction of WHO TB < 5	0.514 (0.485 - 0.543)
referral PHC -> Hospital after clinical re-assessment following bacteriological negative result **Ethiopia**	expert opinion	see Appendix 2a	0.045 (0.019 - 0.088)
referral PHC -> Hospital after clinical re-assessment following bacteriological negative result **Indonesia**	expert opinion	see Appendix 2a	0.200 (0.107 - 0.272)
referral PHC -> Hospital after initial clinical assessment without bacteriological test result **Ethiopia**	expert opinion	see Appendix 2a	0.800 (0.728 - 0.899)
referral PHC -> Hospital after initial clinical assessment without bacteriological test result **Indonesia**	expert opinion	see Appendix 2a	0.500(0.391 - 0.607)
clinical re-assessment, PHC **Ethiopia**	expert opinion	see Appendix 2a	0.045 (0.019 - 0.088)
clinical re-assessment, PHC **Indonesia**	expert opinion	see Appendix 2a	0.045 (0.019 - 0.088)
proportion of bacteriologically confirmed children initiating anti-TB treatment, PHC	assumption		0.953 (0.937 - 0.966)
proportion of bacteriologically confirmed children initiating anti-TB treatment, Hospital	assumption		0.953 (0.937 - 0.966)
clinical re-assessment after bacteriologically negative, PHC	assumption		0.045 (0.019 - 0.088)
clinical re-assessment after bacteriologically negative, Hospital	assumption		0.045 (0.019 - 0.088)
clinical re-assessment, Hospital	assumption		0.045 (0.019 - 0.088)
referral PHC -> Hospital after clinical re-assessment without bacteriological test result	assumption		0.500(0.391 - 0.607)
CFR children <5 years on TB treatment	existing review	Jenkins et al 2017[18]	0.019 (0.012 - 0.029)
CFR children 5-14 years on TB treatment	existing review	Jenkins et al 2017[18]	0.008 (0.006 - 0.011)
CFR children <5 years without TB treatment	existing review	Jenkins et al 2017[18]	0.436 (0.413 - 0.460)

For peer review only - http://bmjopen.bmj.com/site/about/guidelines.xhtml

CFR children 5-14 years without TB treatment	existing review	Jenkins et al 2017[18]	0.149 (0.137 - 0.162)

For peer review only

For peer review only - http://bmjopen.bmj.com/site/about/guidelines.xhtml

Table 2 Unit costs for different activities. See Appendix 2b for methods and naming conventions

Cost description	Unit cost, US$ (SD)	
	**Ethiopia**	**Indonesia**
TB assessment at health centre	10.22 (5.29)	43.35 (24.24)
TB reassessment at health centre	5.11 (2.25)	21.68 (10.52)
Self-expectorated sputum sample	2.32 (0.58)	1.74 (0.43)
Stool sample	1.67 (0.42)	1.67 (0.42)
Sputum smear microscopy examination	3.39 (1.44)	7.54 (1.58)
GeneXpert test	26.04 (7.09)	23.70 (7.11)
TB treatment at health centre	398.74 (177.22)	161.03 (78.59)
TB assessment at hospital	14.37 (6.59)	61.00 (30.23)
TB reassessment at health centre	5.11 (2.25)	21.68 (10.52)
TB treatment at hospital	548.46 (208.38)	213.98 (91.47)

For peer review only - http://bmjopen.bmj.com/site/about/guidelines.xhtml

Table 3 Outcomes per 100 children seeking care (*asterisk indicates different denominators) under standard of care (SOC) and intervention (INT) in each country. Quoted as Mean (95% quantiles). ϕanti-TB treatments (ATT) represent the number of children diagnosed with TB who initiate treatment out of 100 children with presumptive TB. TB: tuberculosis; ATT: anti-TB treatment; PHC: primary health care; USD: United States Dollars

Quantity per 100 children with presumptive TB (unless stated):	Ethiopia			Indonesia		
	Standard of care	Intervention	Difference	Standard of care	Intervention	Difference
children with true TB	45.5 (8.7 - 85.0)	45.5 (8.7 - 85.0)	0.0 (0.0 - 0.0)	45.5 (8.7 - 85.0)	45.5 (8.7 - 85.0)	0.0 (0.0 - 0.0)
assessments	201.8 (171.8 - 230.9)	246.2 (207.3 - 283.3)	44.4 (29.5 - 58.1)	204.2 (173.4 - 233.5)	249.9 (211.2 - 286.5)	45.7 (31.9 - 58.0)
bacteriological investigations	30.7 (8.7 - 57.5)	102.3 (86.8 - 112.0)	71.7 (41.5 - 96.3)	24.7 (7.8 - 43.2)	103.0 (87.5 - 112.6)	78.2 (54.5 - 98.4)
ϕanti-TB treatments (ATT)	32.2 (13.2 - 54.5)	40.3 (17.6 - 64.4)	8.1 (0.6 - 20.3)	33.3 (14.1 - 55.3)	39.5 (17.1 - 63.3)	6.2 (0.1 - 15.2)
*ATT initiated at PHC	71.8 (62.3 - 79.6)	81.9 (71.6 - 89.5)	10.1 (5.8 - 14.2)	73.0 (63.2 - 80.3)	84.4 (73.2 - 91.2)	11.3 (7.1 - 15.4)
* percent of true TB receiving ATT	58.3 (43.0 - 71.1)	73.0 (66.7 - 78.8)	14.7 (2.8 - 30.5)	60.3 (48.2 - 71.4)	71.8 (65.9 - 77.3)	11.5 (1.8 - 23.1)
*percent of ATT bacteriologically confirmed	8.0 (1.7 - 19.8)	32.8 (20.7 - 44.1)	24.8 (10.6 - 37.8)	5.9 (1.4 - 12.9)	32.5 (20.9 - 43.4)	26.6 (14.9 - 38.2)
*percent of ATT false-positive	21.9 (2.8 - 64.6)	21.9 (2.9 - 64.9)	0.0 (-3.0 - 4.0)	22.0 (2.8 - 65.1)	21.8 (2.9 - 64.6)	-0.3 (-3.5 - 3.5)
referrals, inc. self-referrals	29.5 (17.0 - 42.9)	13.8 (8.0 - 21.0)	-15.6 (-25.8 - -4.9)	33.0 (21.5 - 45.5)	14.5 (8.6 - 21.7)	-18.4 (-27.6 - -9.6)
deaths	4.9 (0.9 - 10.0)	3.9 (0.7 - 8.3)	-1.0 (-2.8 - -0.1)	5.4 (1.0 - 10.9)	4.7 (0.9 - 9.3)	-0.8 (-2.2 - 0.0)
life-years lost	135.7 (25.1 - 276.9)	108.7 (19.7 - 228.5)	-27.0 (-75.9 - -1.6)	154.8 (29.3 - 310.1)	133.1 (24.7 - 264.6)	-21.7 (-61.7 - 0.2)
cost	15729.4 (6368.3 - 31027.5)	19297.7 (8413.8 - 35444.7)	3568.3 (-8472.2 - 16311.6)	12508.1 (7056.4 - 20279.0)	14525.7 (8603.6 - 22403.0)	2017.6 (-5421.3 - 9470.6)

For peer review only - http://bmjopen.bmj.com/site/about/guidelines.xhtml

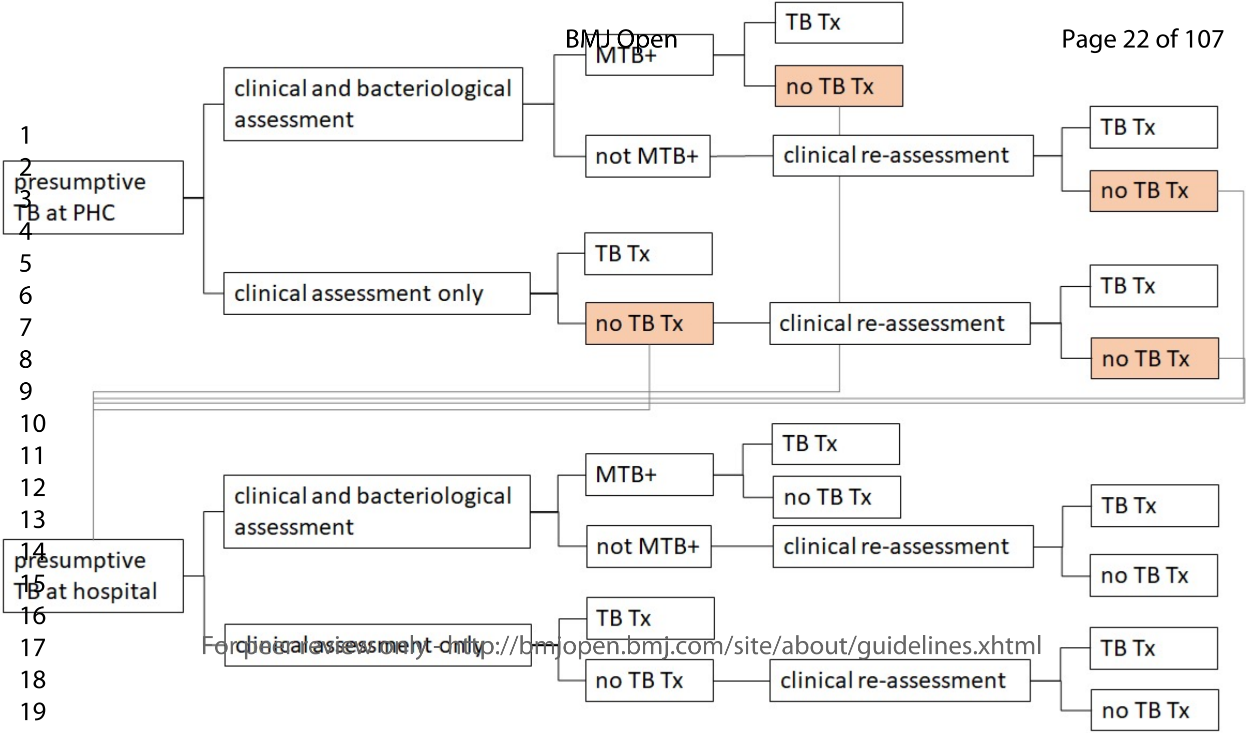
presumptive TB at PHC
clinical and bacteriological assessment
MTB+
TB Tx
no TB Tx
not MTB+
clinical re-assessment
TB Tx
no TB Tx
clinical assessment only
TB Tx
no TB Tx
clinical re-assessment
TB Tx
no TB Tx
presumptive TB at hospital
clinical and bacteriological assessment
MTB+
TB Tx
no TB Tx
not MTB+
clinical re-assessment
TB Tx
no TB Tx
clinical assessment only
TB Tx
no TB Tx
clinical re-assessment
TB Tx
no TB Tx

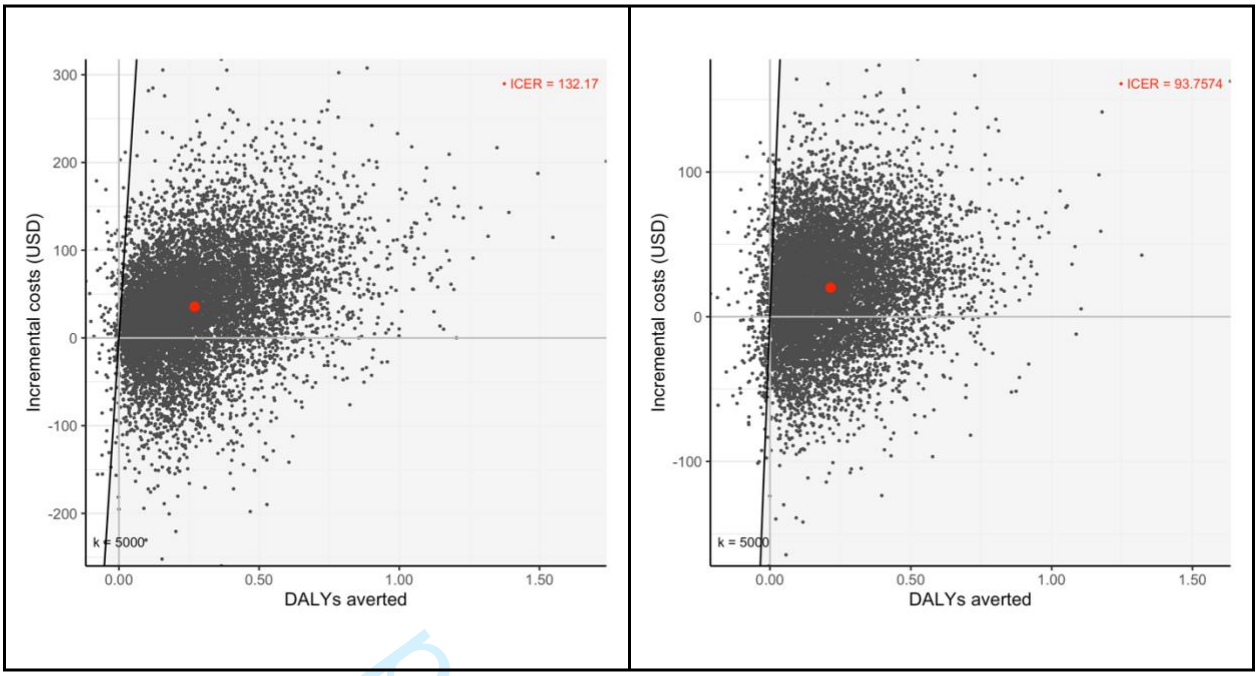

peer review only

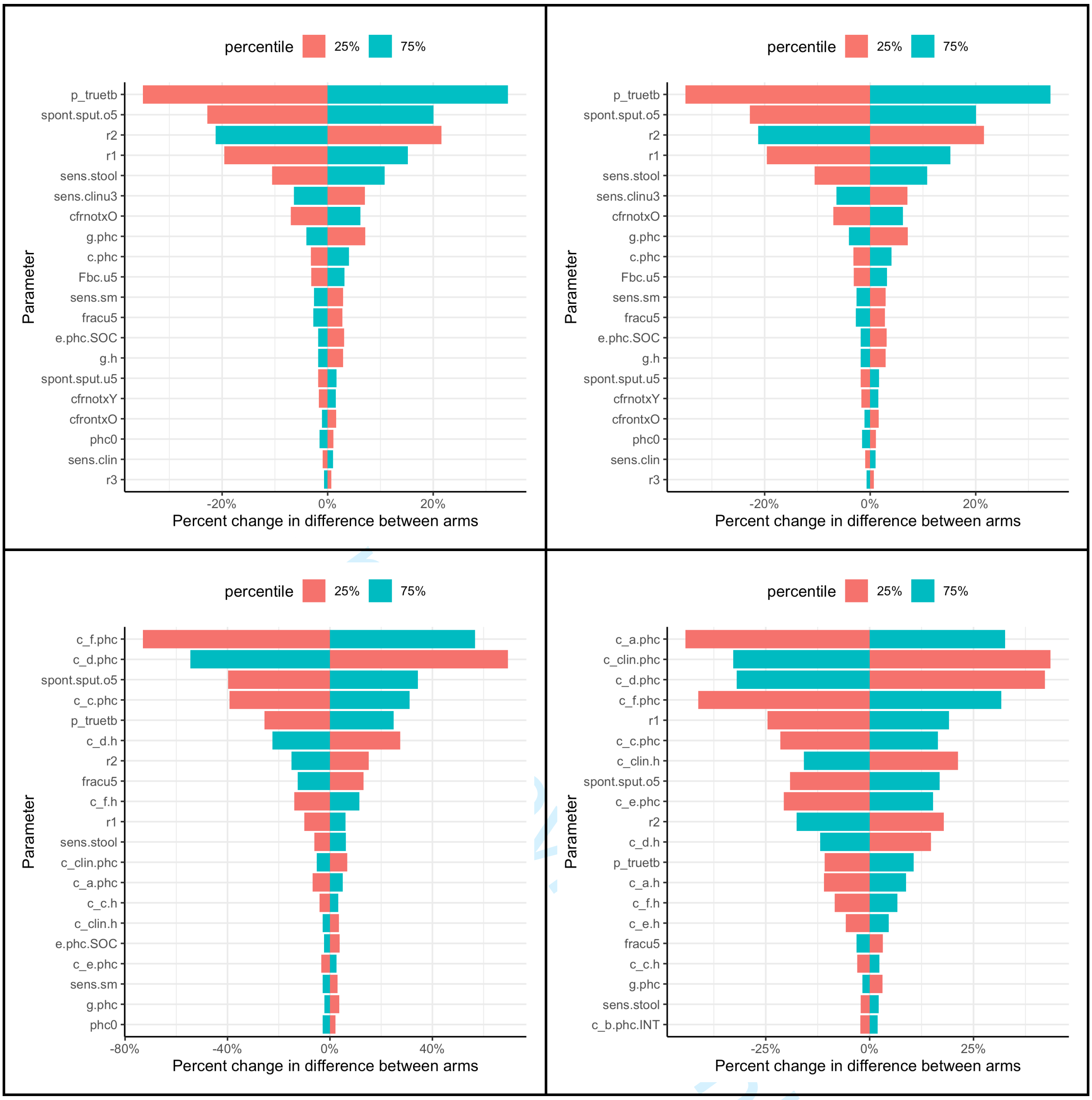
percentile
25%
75%
Parameter
p_truetb
spont.sput.o5
r2
r1
sens.stool
sens.clinu3
cfrnotxO
g.phc
c.phc
Fbc.u5
sens.sm
fracu5
e.phc.SOC
g.h
spont.sput.u5
cfrnotxY
cfrontxO
phc0
sens.clin
r3
-20%
0%
20%
Percent change in difference between arms
c_f.phc
c_d.phc
c_c.phc
c_d.h
c_f.h
c_clin.phc
c_a.phc
c_c.h
c_clin.h
c_e.phc
-80%
-40%
40%
c_e.h
c_a.h
c_b.phc.INT
-25%
25%

Xpert Ultra stool testing to diagnose tuberculosis in children in Ethiopia and Indonesia: a model-based cost-effectiveness analysis.

APPENDIX 1: Literature search

To inform the model parameters presented in Appendix 2a, we extracted data from systematic reviews and from papers identified through an extensive targeted systematic literature search. This information was supplemented with information from papers identified from the authors' personal databases where relevant.

Data collection from published peer-reviewed systematic-reviews

We identified relevant systematic reviews and meta-analyses on TB in children in PubMed (www.pubmed.ncbi.nlm.nih.gov). Search details are provided in Box A1.1.

Box A1. Search strategy for systematic reviews

Searched in Pubmed for "systematic review meta-analysis tuberculosis children", which is interpreted by the search engine as:

(("systematic review"[Publication Type] OR "systematic reviews as topic"[MeSH Terms]) OR "systematic review"[All Fields]) AND (("meta-analysis"[Publication Type] OR "meta-analysis as topic"[MeSH Terms]) OR "meta-analysis"[All Fields]) AND (((("tuberculosi"[All Fields] OR "tuberculosis"[MeSH Terms]) OR "tuberculosis"[All Fields]) OR "tuberculoses"[All Fields]) OR "tuberculosis s"[All Fields]) AND ((((("child"[MeSH Terms] OR "child"[All Fields]) OR "children"[All Fields]) OR "child s"[All Fields]) OR "children s"[All Fields]) OR "childrens"[All Fields]) OR "childs"[All Fields])

Search date: 19 June 2020.

Of the 150 systematic reviews identified (of which one was a duplicate paper), 23 were judged relevant for full-text review (Figure A1.1). Of the 22 papers reviewed in full-text, four papers contained information about the accuracy of relevant microbiological tests for TB (1-4). However, one of these did not present meta-analytical estimates of the sensitivity and specificity of the test (Xpert Ultra, in this paper) for children (4). Two other papers presented data on the same subject and included roughly the same original work (2, 3), while for one of these, the pooled estimates presented were difficult to interpret as no comparison against culture or Xpert only was included (3). Thus, two papers provided relevant data for extraction (Figure 1): Detjen et al. (1) reported meta-analytic estimates of the sensitivity and specificity of sputum smear microscopy and Xpert on sputum, gastric lavage and nasopharyngeal aspirates (here summarized as 'respiratory samples') against culture of a respiratory sample. Mesman and colleagues (3) presented meta-analytic estimates of the sensitivity and specificity of Xpert stool testing against different reference standards (culture or Xpert on a respiratory sample, bacteriologically confirmed TB, and clinically diagnosed unconfirmed TB).

For peer review only - http://bmjopen.bmj.com/site/about/guidelines.xhtml

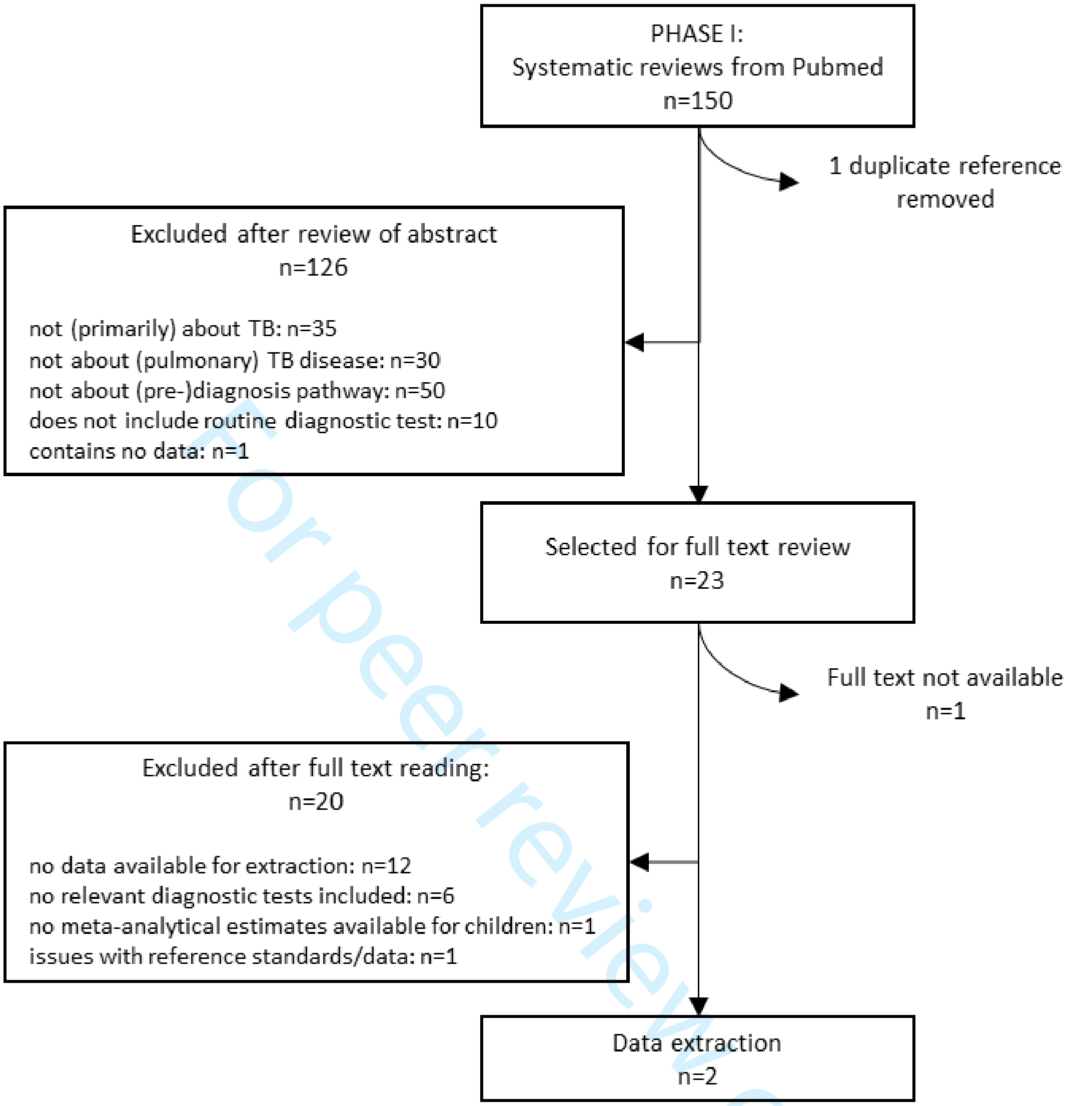

Figure A1. Prisma flow diagram for a search including published peer-reviewed systematic reviews and meta-analysis summarizing literature on TB in children.

Data collection from published peer-reviewed original papers

Next, we conducted a literature search to identify studies about TB diagnostic testing in infants and children, including health care seeking and health care cascade, with a focus on Ethiopia and Indonesia. A systematic search strategy was developed with assistance of an academic librarian from the University of Sheffield. The search strategy used a combination of free-text and thesaurus searching (where available) as outlined in Table A1. Papers with terms for Bacillus Calmette Guérin (BCG), latent tuberculosis, interferon-gamma release assay (IGRA) and tuberculin skin test in titles were excluded from the search as they are relevant to TB infection, but not to active pulmonary TB and were therefore deemed to retrieve irrelevant results. Case reports were excluded as these do not provide generalizable data. The searches were limited to English Language and Human studies published from 2010 - present where databases allowed, except for searches specifically addressing Indonesia/Ethiopia to which no such limits were applied (Table A1). Searches were conducted between 19 and 26 October 2020. Further details of the search strategy are provided in Box A1.2.

For peer review only - http://bmjopen.bmj.com/site/about/guidelines.xhtml

Table A1. Overview of search terms used for searching peer-reviewed original papers.

Exploded MeSH/lookup term	Occurring in title	Occurring in title or abstract[1]
The following were combined using 'AND':		
Tuberculosis or Diagnosis	tuberculosis or TB	
Child or Infant		child or infan[2]
Sputum or Feces		(sputum or stool or f?eces) and (test or sample or specimen)
		test* or diagnos* or screen*
Indonesia or Ethiopia or Developing Countries		Indonesia or Ethiopia or Africa or Asia or West Indies or specific countries[3]
The following were combined with the previous using 'NOT':		
case reports[4]		case report
	bacilli Calmette-Guerin or BCG	
	latent tuberculosis or LTBI or Interferon Gamma Release Assay or IGRA or tuberculin skin test or TST	

[1] In the Cochrane library, key word searches were also included here for all terms, except for the regions and countries; in Medline, this was done only for the terms *child* and *infan*; [2] In Cochrane and Science Citation Index via Web of Science, these terms were replaced with infant*; [3] Specific countries included: Angola, Bangladesh, Benin, Bolivia, Burkina Faso, Burkina Fasso, Burundi, Cambodia, Central African Republic, Chad, Congo, Cote d'Ivoire, Ivory Coast, Djibouti, Egypt, Eritrea, Gambia, Ghana, Guatemala, Guinea, India, Kenya, Korea, Lao PDR, Lesotho, Liberia, Madagascar, Malawi, Mali, Mauritania, Mozambique, Myanmar, Myanma, Nepal, Niger, Nigeria, Philippines, Philipines, Phillipines, Phillippines, Rwanda, Ruanda, Sao Tome, Senegal, Sri Lanka, Somalia, Sudan, Swaziland, Tanzania, Timor-Leste, Togo, Uganda, Vietnam, Viet Nam, Zambia, Zimbabwe; searches for other countries than Ethiopia and Indonesia were limited to English language and humans and *yr="2010 -Current"*; [4] Only included if this lookup term existed for the system (see box 2 for specifications).

In total, 2,974 unique titles were available for title screening, from which 770 were selected for abstract screening. Subsequently, we selected 260 papers for full-text review, of which, after review, data were extracted from 73 (Figure A1.2). The extracted information from these 73 papers was reviewed by the modeling team for its usefulness and applicability to inform the model. Finally, the extracted data for 21 papers was judged to be directly relevant to inform the model. Table A2 provides an overview of all 73 papers for which data was extracted, and specifies which papers were used to inform the model.

For peer review only - http://bmjopen.bmj.com/site/about/guidelines.xhtml

Box A2. Details of search strategy for peer-reviewed original publications

- Developed in MEDLINE
- studies about TB diagnostic testing in infants and children
- two sets of search results:
  - Indonesia or Ethiopia
  - other countries in Africa or Asia (adapting the Cochrane LMIC filter (https://epoc.cochrane.org/lmic-filters).
- Searches were further specified following examination of 100 references in pilot search, excluding terms for BCG, latent tuberculosis, IGRA and tuberculin skin test appearing in titles
- Case reports were excluded where possible

Database	Date Searched	Number of References Retrieved (including duplicates)	Total N retrieved (including duplicates)
Ovid MEDLINE(R)<br>1946 to Oct Week 3 2020	23/10/20	Indonesia & Ethiopia = 180<br>Other countries = 1,198	**Indonesia & Ethiopia = 537**<br><br>**Other countries = 4,348**
Ovid MEDLINE(R) and Epub Ahead of Print, In-Process & Other Non-Indexed Citations and Daily<br>2016 to Oct 22, 2020*	23/10/20	Indonesia & Ethiopia = 97<br>Other countries = 790	
Ovid Embase<br>1974 to Oct 23, 2020**	26/10/20	Indonesia & Ethiopia = 237<br>Other countries = 1,783	
Cochrane Database of Systematic Reviews<br>Issue 10 of 12, Oct 2020	19/10/20	Indonesia & Ethiopia = 2<br>Other countries = 11 (plus one protocol)	
Cochrane Central Register of Controlled Trials<br>Issue 10 of 12, Oct 2020	19/10/20	Indonesia & Ethiopia = 4<br>Other countries = 108	
Science Citation Index via Web of Science<br>1900-present	26/10/20<br>19/10/20	Indonesia & Ethiopia = 17<br>Other countries = 457	
Conference Proceedings Citation Index-Science (CPCI-S) via Web of Science<br>1990-present	26/10/20	Indonesia & Ethiopia = 1<br>Other countries = 2	

** English Language and Human limits removed as do not work correctly in MEDLINE In-Process; ** case report(s) not a publication type in Embase*

For peer review only - http://bmjopen.bmj.com/site/about/guidelines.xhtml

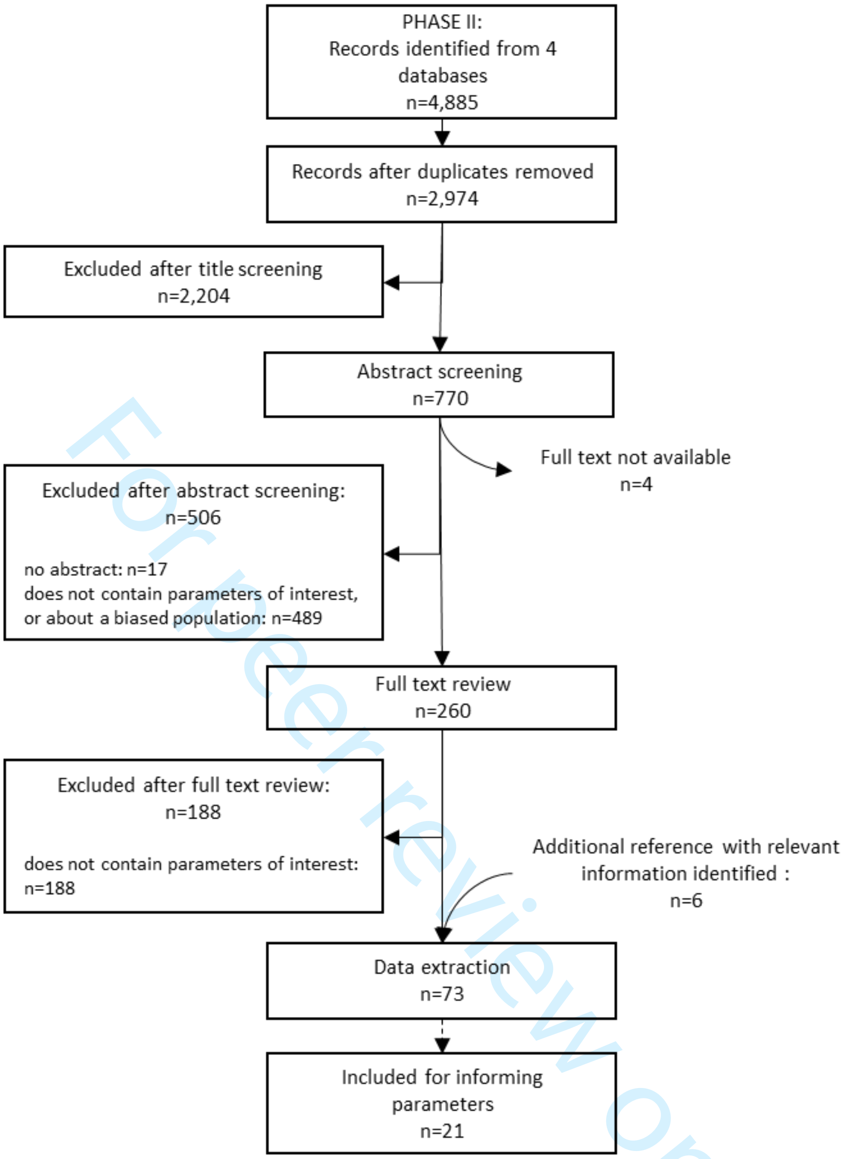

Figure A2. Prisma flow diagram for systematic literature review on TB diagnostic testing in infants and children.

For peer review only - http://bmjopen.bmj.com/site/about/guidelines.xhtml

Table A2. Overview of papers from which data was extracted in the comprehensive literature review of phase 2 (see Figure 2 for details).

Reference (first author and year of publication)	Country	Child population included (N)	Age range	Health care level (no. centers)	Data extracted	Model parameters informed	Comment (e.g., reason for not being considered for informing model parameters)
Andriyoko, 2019 (5)	Indonesia	lab-based, stool plus sputum/NGA submitted for TB diagnosis (36)	0-15y	level 3 (1)	% of presumptive TB cases with confirmed TB		unclear how study population was composed (laboratory study)
Ardizzoni, 2015 (6)	multiple	register review of samples with Xpert results (1,278)	0-14y	NA	% of presumptive TB cases with confirmed TB		Indonesia nor Ethiopia included; data shown on all ages but data extracted for children and (induced) sputum/NGA only
Assefa, 2015 (7)	Ethiopia	household contacts of SS+TB patients (230)	0-5y	level 1 (27)	% of child population seeking care		biased population (semi-active case finding)
Atwebembeire, 2016 (8)	Uganda	lab-based, string test and induced sputum samples (88)	NS	level 3 (1)	% of samples MTB-positive		No information on final TB diagnosis
Bacha, 2017 (9)	Tanzania	presumptive TB or referred for TB treatment (455)	0-14y	level 2/3 (1)	% of presumptive TB cases with confirmed, probable and possible TB	*p_truetb*	
Banada, 2016 (10)	South Africa	consecutive confirmed (20) and probable (20) TB cases	0-14y	level1 (NS) and 2 (NS)	% of samples MTB-positive, by type of sample		Only includes diagnosed TB patients
Bates, 2013 (11)	Zambia	primary or secondary admission diagnosis of TB (930)	0-15y	level 3 (1)	% of presumptive TB cases submitting respiratory specimen for TB diagnosis, by type of specimen and age group<br>% of children with true TB	*spont.sput*	
van Beekhuizen, 1998* (12)	Papua New Guinea	admitted for malnutrition, recurrent pneumonia, or signs/symptoms of TB (301)	0-16y	level 1 (1)	Sensitivity and specificity of clinical diagnosis		Evaluated the sensitivity and specificity of a TB score chart instead of pediatrician's diagnosis
Beneri, 2016 (13)	South Africa	presumptive TB in RCT on TPT for HIV-exposed and -infected (219)	<5y	NA	% of presumptive TB cases with confirmed, probable and possible TB; Sensitivity and specificity of clinical diagnosis		considered for informing *sens.clin* and *spec.clin*, but not a representative population (semi-active case finding)
Berggren-Palme, 2004 (14)	Ethiopia	clinically diagnosed with TB (355)	0-14y	level 3 (1)	% of TB cases submitting spontaneously expectorated sputum for TB diagnosis		only diagnosed TB patients included
Binua, 2019 (15)	Philippines	presumptive TB (incl. EPTB) (112)	4-18y	level 3 (1)	% of presumptive TB cases with definite (smear-positive) TB		abstract only, no detailed information; EPTB included

For peer review only - http://bmjopen.bmj.com/site/about/guidelines.xhtml

Reference (first author and year of publication)	Country	Child population included (N)	Age range	Health care level (no. centers)	Data extracted	Model parameters informed	Comment (e.g., reason for not being considered for informing model parameters)
Bojang, 2016 (16)	The Gambia	presumptive TB (24)	0-14y	level 1 (NS) & research unit (1)	% of presumptive TB cases with definite (Xpert) TB		No information on final TB diagnosis
Brent, 2017 (17)	Kenya	presumptive TB (1,442)	0-14y	level 2 (2)	% of presumptive TB cases with confirmed, highly probable and possible TB		did not use standard clinical case definitions, EPTB included which cannot be separated from pulmonary TB
Bunyasi, 2015 (18)	South Africa	investigated for incident TB in vaccine trial (active & passive FU) (1,020)	<4y	NA	% of presumptive TB cases with confirmed TB		No information on final TB diagnosis; non-representative population
Chipinduro, 2017 (19)	Zimbabwe	presumptive TB (221)	5-16y	level 1 (8)	% of presumptive TB cases submitting induced sputum for TB diagnosis		no data on % spontaneously expectorating sputum
Chisti, 2013 (20)	multiple	acute pneumonia with SAM and/or HIV infection (747)	<5y	NS (NS)	% of children with acute pneumonia being diagnosed with confirmed TB		population not representative for children with presumptive TB
Das, 2019 (21)	India	presumptive TB, partly admitted (171)	0-14y	level 3 (1)	% of presumptive TB cases with confirmed (smear/Xpert-positive) TB		no data on % clinically diagnosed with pulmonary TB
Dayal, 2020 (22)	India	diagnosed with probable TB (114)	0-13y	level 3 (1)	% of samples MTB-positive, by type of sample		Only includes diagnosed TB patients
Elhassan, 2016 (23)	Sudan	presumptive TB (197)	0-13y	level 1 (5)	% of presumptive TB cases with confirmed, probable and possible TB	*p_truetb*	
Eliso, 2015 (24)	Ethiopia	cough of any duration (43)	6-15y	level 1 (4)	% of presumptive TB cases with definite (smear-positive) TB		only smear-positive TB included
Fekadu, 2017* (25)	Ethiopia	NA	NA	level 1 (NA)	% of children seeking care at level 0/1 health facilities first	*phc0_e*	this study used data from multiple sources to estimate TB care cascade
Garcia-Basteiro, 2015 (26)	Mozambique	presumptive TB (766)	0-2y	NA (Research Center) (1)	% of presumptive TB cases with definite and probable TB		possible TB not presented in the paper, limited age bands
Giang, 2015 (27)	Vietnam	presumptive TB (150)	0-14y	level 3 (1)	% of presumptive TB cases with confirmed, probable and possible TB	*p_truetb*	
Gous, 2015 (28)	South Africa	presumptive TB (484)	0-14y	level 2 (1)	% of samples MTB-positive, by method		No information on final TB diagnosis
Hanrahan, 2019 (29)	South Africa	presumptive TB (119)	2m-10y	level 1 (1, high-volume)	% of presumptive TB cases submitting respiratory specimen for TB diagnosis, by type of specimen and age group	*spont.sput, p_truetb*	

For peer review only - http://bmjopen.bmj.com/site/about/guidelines.xhtml

Reference (first author and year of publication)	Country	Child population included (N)	Age range	Health care level (no. centers)	Data extracted	Model parameters informed	Comment (e.g., reason for not being considered for informing model parameters)
Kabir, 2018 (30)	Bangladesh	clinically diagnosed with TB (102)	0-14y	level 3 (1)	% of samples MTB-positive, by method		No information on final TB diagnosis
Kabir, 2020 (31)	Bangladesh	presumptive TB (448)	0-14y	level 3 (1)	% of presumptive TB cases submitting induced sputum and/or stool for TB diagnosis		no data on % spontaneously expectorating sputum
Kalra, 2020 (32)	India	presumptive TB (94,415)	0-14y	level 3 (1)	% of presumptive TB cases submitting any specimen for TB diagnosis, by type of specimen		no data on % spontaneously expectorating sputum
Kalu, 2013 (33)	Nigeria	presumptive TB (263)	3m-14y	level 3 (1)	% of presumptive TB cases with confirmed (culture-positive and/or smear-positive) TB		no data on % clinically diagnosed with pulmonary TB
Lopez-Varela, 2015** (34)	Mozambique	presumptive TB (789)	0-2y	NS (Research Center) (1)	% of presumptive TB cases with definite, probable and possible TB		limited age bands
Marais, 2006* (35)	South Africa	cough>2 weeks without response to oral antibiotics course (428)	0-12y	level 1 (5)	Sensitivity and specificity of clinical diagnosis	*sens.clin*, *spec.clin*	
Moussa, 2016 (36)	Egypt	presumptive TB (115)	0-15y	level 3 (1)	% of presumptive TB cases with confirmed, probable and possible TB	*p_truetb*	
Mukherjee, 2013 (37)	India	clinically diagnosed intrathoracic TB (403)	6m-15y	level 3 (2)	% of bacteriologically confirmed TB		only includes diagnosed TB patients
Mulenga, 2011 (38)	South Africa	two cohorts investigated for incident TB in two vaccine trials (active & passive FU) (1,445+740)	0-2y?	NA	% of child population seeking care<br><br>Sensitivity and specificity of clinical diagnosis		mixture of PHC and hospital care seeking, limited age bands;<br>contains % with different combinations of symptoms and signs of TB, but no data for parameter of interest
Mulenga, 2015 (39)	South Africa	investigated for incident TB in vaccine trial (active FU) (1,017)	0-2y	NA	Sensitivity and specificity of clinical diagnosis		contains % with different combinations of symptoms and signs of TB, but no data for parameter of interest
Munoz-Sellart, 2009 (40)	Ethiopia	diagnosed with TB (231)	0-14y	level 1 (7) and 2 (1)	% of smear-positive TB		only includes diagnosed TB patients
Mwangwa, 2017 (41)	Uganda	diagnosed with TB in HIV RCT (42)	0-15y	level 1 (32 communities)	% of bacteriologically confirmed TB cases started on treatment		likely higher than SOC
Myo, 2018 (42)	Myanmar	presumptive TB (231)	1m-12y	level 3 (1)	% of presumptive TB cases with confirmed and unconfirmed TB<br>% of bacteriologically confirmed TB cases started on treatment	*p_truetb*	 likely higher than SOC

For peer review only - http://bmjopen.bmj.com/site/about/guidelines.xhtml

Reference (first author and year of publication)	Country	Child population included (N)	Age range	Health care level (no. centers)	Data extracted	Model parameters informed	Comment (e.g., reason for not being considered for informing model parameters)
Nansumba, 2016 (43)	Uganda	presumptive TB (137)	3-14y	level 3 (1)	% of presumptive TB cases submitting respiratory specimen for TB diagnosis; % of presumptive TB cases with confirmed (culture-positive) TB; % of bacteriologically confirmed TB cases started on treatment		no data on % spontaneously expectorating sputum; no data on clinically diagnosed TB; likely higher than SOC
Negash, 2020 (44)	Ethiopia	lab-based, any sputum received for TB diagnosis (414)	4-14y	level 1 (4 hospitals, 34 HCs)	% of presumptive TB cases with Xpert-positive TB		no data on clinically diagnosed TB
Nhu, 2013 (45)	Vietnam	presumptive TB (73)	0-15y	level 3 (1)	% of bacteriologically confirmed TB		only includes diagnosed TB patients
Nicol, 2011 (46)	South Africa	admitted for presumptive TB (452)	0-15y	level 3 (2)	% of bacteriologically confirmed TB; % of bacteriologically confirmed TB cases started on treatment	*Fbc*	only includes in-patients in level-3 hospital likely higher than SOC
Nicol, 2013 (47)	South Africa	presumptive TB (115)	0-14y	level 1 (1) and level 3 (1)	% of presumptive TB cases with confirmed, probable and possible TB	*p_truetb*	none of the children was diagnosed with possible TB
Nicol, 2019 (48)	South Africa	presumptive TB (165)	0-14y	level 3 (1)	% of presumptive TB cases with confirmed and unconfirmed TB; % of bacteriologically confirmed TB cases started on treatment	*p_truetb*	likely higher than SOC
Nissen, 2012 (49)	Tanzania	presumptive TB (195)	0-14y	level 1/2 (1)	% returning for clinical re-evaluation after initial exclusion of TB		likely higher than SOC as asked to return by the study team
Oliwa, 2019 (50)	Kenya	admitted for presumptive TB (23,741)	0-15y	level 2 (13)	% of presumptive TB cases that gets TST, chest X-ray, and bacteriology offered		not regarded sufficiently representative for Ethiopia and Indonesia
Orikiriza, 2018 (51)	Uganda	presumptive TB, partly admitted (392)	1m-14y	level 2/3 (1)	% of presumptive TB cases with confirmed TB, % started on TB treatment		case definitions provided in Methods section were not used to present results
Pearce, 2012* (52)	NA	NA	NA	NA	Sensitivity and specificity of clinical diagnosis		review, no original data; only one study identified providing a sensitivity score
Ramos, 2013 (53)	Ethiopia	retrospective review of sputum reports (875)	0-14y	level 2 (1)	% of presumptive TB cases with smear-positive TB		only smear-positive TB included
Ramos, 2019 (54)	Ethiopia	diagnosed with smear-positive TB (862)	0-14y	level 2 (1)	% of TB patients diagnosed with smear-positive TB		only diagnosed TB patients included

For peer review only - http://bmjopen.bmj.com/site/about/guidelines.xhtml

Reference (first author and year of publication)	Country	Child population included (N)	Age range	Health care level (no. centers)	Data extracted	Model parameters informed	Comment (e.g., reason for not being considered for informing model parameters)
Raizada, 2014 (55)	India	presumptive pulmonary TB (4,600)	0-14y	network of level 1 HF (±400), microscopy centers (99), and sub-district TB units (18)	% of presumptive TB cases with bacteriologically confirmed (by SSM/ Xpert) TB		no data on clinically diagnosed TB
Raizada, 2015 (56)	India	presumptive pulmonary TB (517)	0-14y	as Razaida, 2014 (55)	% of presumptive TB cases with bacteriologically confirmed (by SSM/ Xpert) TB		no data on clinically diagnosed TB
Raizada, 2018a (57)	India	lab-based study, presumptive TB (3,045)	0-14y	central Xpert labs (4) receiving samples from all levels (public & private) in 4 big cities	% of bacteriologically confirmed TB cases started on treatment		also EPTB included, no differentiation by type of TB possible
Raizada, 2018b (58)	India	lab-based study, presumptive TB (465)	<2y	as Razaida, 2018a (57)	% of bacteriologically confirmed TB cases started on treatment		also EPTB included, no differentiation by type of TB possible
Reither, 2015 (59)	Uganda, Tanzania	presumptive TB (451)	2m-15y	NA (Research Center) (2), level 2 (1)	% of presumptive TB cases with confirmed, highly probable and probable TB	*p_truetb*	
Sabi, 2016 (60)	Tanzania	presumptive TB (192)	2m-12y	level 2 (1)	% of presumptive TB cases submitting respiratory specimen for TB diagnosis;<br>% of presumptive TB cases with confirmed, probable and possible TB	*p_truetb*	no data on % spontaneously expectorating sputum
Sabi, 2018 (61)	Tanzania	presumptive TB (277)	6m-16y	NS (Research Center) (2)	% of presumptive TB cases with confirmed, highly probable and probable TB		Focus of report on stored sputum samples tested with Xpert Ultra. More relevant data presented in Reither, 2015
Sekadde, 2013 (62)	Uganda	presumptive TB (235)	2m-12y	level 3 (1)	% of presumptive TB cases submitting induced sputum for TB diagnosis;<br>% of presumptive TB cases with confirmed TB		no data on % spontaneously expectorating sputum;<br>no data on clinically diagnosed TB
Sharma, 2020 (63)	India	non-expectorating with strong clinical suspicion of TB (210)	6m-12y	level 3 (1)	% of presumptive TB cases with bacteriologically confirmed TB		non-expectorating children only; no data on clinically diagnosed TB

For peer review only - http://bmjopen.bmj.com/site/about/guidelines.xhtml

Reference (first author and year of publication)	Country	Child population included (N)	Age range	Health care level (no. centers)	Data extracted	Model parameters informed	Comment (e.g., reason for not being considered for informing model parameters)
Shata, 1996 (64)	Malawi	presumptive TB (29)	3-15y	level 3 (1)	% of presumptive TB cases submitting induced sputum for TB diagnosis		no data on % spontaneously expectorating sputum
Singh, 2016 (65)	India	presumptive TB (50)	0-14y	NS (3)	% of presumptive TB cases with confirmed (SSM) and probable TB		no internationally accepted definition used for the clinical definition of TB
Sorsa, 2020 (66)	Ethiopia	presumptive TB (775)	0-14y	level 3 (1)	% of presumptive TB cases with confirmed (SSM/Xpert) and probable TB	*p_truetb*	
Ssengooba, 2020 (67)	Uganda	diagnosed with "minimal TB" participating in clinical trial (353)	0-15y	NS	% of samples MTB-positive, by type of sample		only diagnosed TB patients included
Surya, 2017* (68)	Indonesia	NA	NA	level 1 (NA)	% of children seeking care at level 0/1 health facilities first	*phc0_i*	this study used data from multiple sources to estimate TB care cascade
Swaminathan, 2008 (69)	India	presumptive TB (2,652)	6m-12y	level 3 (3)	% of presumptive TB cases with bacteriologically confirmed TB		no data on clinically diagnosed TB
Walters, 2017a (70)	South Africa	presumptive intrathoracic TB (188)	0-12y	level 3 (2)	% of presumptive TB cases with confirmed and unconfirmed TB		population is the same as presented in Walters, vd Zalm, 2017
Walters, 2017b (71)	South Africa	presumptive intrathoracic TB (379)	0-12y	level 3 (2)	% TB bacteriologically positive under ideal conditions<br>% of presumptive TB cases with confirmed and unconfirmed TB	*Fbc,*<br>*p_truetb*	
Walters, 2018 (72)	South Africa	presumptive TB (148)	0-15y	level 3 (2)	% of presumptive TB cases submitting stool for TB diagnosis;<br>% of presumptive TB cases with confirmed and unconfirmed TB<br>% started on TB treatment after clinical re-evaluation	*p_truetb*	no data on % spontaneously expectorating sputum<br>likely higher than SOC
Yadav, 2020 (73)	India	presumptive TB (155)	0-15y	level 3 (1)	% of presumptive TB cases submitting respiratory specimen for TB diagnosis, by type of specimen		not clear if spontaneous expectoration was attempted in all children
Zar, 2005 (74)	South Africa	admitted for presumptive TB (250)	1m-5y	level 3 (2)	% of presumptive TB cases with confirmed (SSM/culture) TB		no data on clinically diagnosed TB
Zar, 2012 (75)	South Africa	admitted for different severe conditions with presumptive TB (535)	0-14y	level 3 (2)	% TB bacteriologically positive under ideal conditions<br>% of presumptive TB cases with definite (Xpert/culture) and possible TB	*Fbc*	non-representative population (hospitalized with severe conditions)

For peer review only - http://bmjopen.bmj.com/site/about/guidelines.xhtml

Reference (first author and year of publication)	Country	Child population included (N)	Age range	Health care level (no. centers)	Data extracted	Model parameters informed	Comment (e.g., reason for not being considered for informing model parameters)
Zar, 2013 (76)	South Africa	presumptive TB (384)	0-14y	level 1 (1, high-volume)	% of presumptive TB cases with definite (Xpert/culture) and possible TB<br>% started on TB treatment after clinical re-evaluation	*p_truetb*	likely higher than SOC
Zar, 2019 (77)	South Africa	admitted for presumptive TB (195)	0-14y	level 3 (1)	% TB bacteriologically positive under ideal conditions<br>% of presumptive TB cases with confirmed and unconfirmed TB	*Fbc*	non-representative population (hospitalized with severe conditions)

Abbreviations used in table: EPTB: extrapulmonary tuberculosis, Fu: follow up, HC: health center, HF: healthcare facility, m: months, MTB: *Mycobacterium tuberculosis*, NA: not applicable, NS: not specified, RCT: randomized controlled trial, SOC: standard of care, SSM: sputum smear microscopy, TB: tuberculosis, y: years; * Identified from authors' personal databases, not through the systematic literature review; ** Identified as paper containing the source data of one of the papers identified in the systematic literature review (26).

For peer review only

For peer review only - http://bmjopen.bmj.com/site/about/guidelines.xhtml

References

1. Detjen AK, DiNardo AR, Leyden J, Steingart KR, Menzies D, Schiller I, Dendukuri N, Mandalakas AM. 2015. Xpert MTB/RIF assay for the diagnosis of pulmonary tuberculosis in children: a systematic review and meta-analysis. Lancet Respir Med 3:451-61.
2. MacLean E, Sulis G, Denkinger CM, Johnston JC, Pai M, Ahmad Khan F. 2019. Diagnostic Accuracy of Stool Xpert MTB/RIF for Detection of Pulmonary Tuberculosis in Children: a Systematic Review and Meta-analysis. J Clin Microbiol 57.
3. Mesman AW, Rodriguez C, Ager E, Coit J, Trevisi L, Franke MF. 2019. Diagnostic accuracy of molecular detection of Mycobacterium tuberculosis in pediatric stool samples: A systematic review and meta-analysis. Tuberculosis (Edinb) 119:101878.
4. Zhang M, Xue M, He JQ. 2020. Diagnostic accuracy of the new Xpert MTB/RIF Ultra for tuberculosis disease: A preliminary systematic review and meta-analysis. Int J Infect Dis 90:35-45.
5. Andriyoko B, Janiar H, Kusumadewi R, Klinkenberg E, de Haas P, Tiemersma E. 2019. Simple stool processing method for the diagnosis of pulmonary tuberculosis using GeneXpert MTB/RIF. European Respiratory Journal 53:03.
6. Ardizzoni E, Fajardo E, Saranchuk P, Casenghi M, Page AL, Varaine F, Kosack CS, Hepple P. 2015. Implementing the Xpert R MTB/RIF Diagnostic Test for Tuberculosis and Rifampicin Resistance: Outcomes and Lessons Learned in 18 Countries. PLoS ONE [Electronic Resource] 10:e0144656.
7. Assefa D, Klinkenberg E, Yosef G. 2015. Cross Sectional Study Evaluating Routine Contact Investigation in Addis Ababa, Ethiopia: A Missed Opportunity to Prevent Tuberculosis in Children. PLoS ONE [Electronic Resource] 10:e0129135.
8. Atwebembeire J, Orikiriza P, Bonnet M, Atwine D, Katawera V, Nansumba M, Nyehangane D, Bazira J, Mwanga-Amumpaire J, Byarugaba F, Boum Y. 2016. Xpert(R) MTB/RIF for detection of Mycobacterium tuberculosis from frozen string and induced sputum sediments. International Journal of Tuberculosis & Lung Disease 20:1113-7.
9. Bacha JM, Ngo K, Clowes P, Draper HR, Ntinginya EN, DiNardo A, Mangu C, Sabi I, Mtafya B, Mandalakas AM. 2017. Why being an expert - despite xpert -remains crucial for children in high TB burden settings. BMC Infectious Diseases 17:123.
10. Banada PP, Naidoo U, Deshpande S, Karim F, Flynn JL, O'Malley M, Jones M, Nanassy O, Jeena P, Alland D. 2016. A Novel Sample Processing Method for Rapid Detection of Tuberculosis in the Stool of Pediatric Patients Using the Xpert MTB/RIF Assay. PLoS ONE [Electronic Resource] 11:e0151980.
11. Bates M, O'Grady J, Maeurer M, Tembo J, Chilukutu L, Chabala C, Kasonde R, Mulota P, Mzyece J, Chomba M, Mukonda L, Mumba M, Kapata N, Rachow A, Clowes P, Hoelscher M, Mwaba P, Zumla A. 2013. Assessment of the Xpert MTB/RIF assay for diagnosis of tuberculosis with gastric lavage aspirates in children in sub-Saharan Africa: a prospective descriptive study. The Lancet Infectious Diseases 13:36-42.
12. van Beekhuizen HJ. 1998. Tuberculosis score chart in children in Aitape, Papua New Guinea. Trop Doct 28:155-60.
13. Beneri CA, Aaron L, Kim S, Jean-Philippe P, Madhi S, Violari A, Cotton MF, Mitchell C, Nachman S. 2016. Understanding NIH clinical case definitions for pediatric intrathoracic TB by applying them to a clinical trial. International Journal of Tuberculosis and Lung Disease 20:93-100.
14. Berggren Palme I, Gudetta B, Bruchfeld J, Eriksson M, Giesecke J. 2004. Detection of Mycobacterium tuberculosis in gastric aspirate and sputum collected from Ethiopian HIV-positive and HIV-negative children in a mixed in- and outpatient setting. Acta Paediatrica 93:311-5.
15. Binua F, Tuazon A. 2019. The yield of AFB smear and tb culture in the diagnosis of childhood tb using sputum induction with n-acetylcysteine: a randomized controlled trial. Pediatric pulmonology 54:S79-.
16. Bojang AL, Mendy FS, Tientcheu LD, Otu J, Antonio M, Kampmann B, Agbla S, Sutherland JS. 2016. Comparison of TB-LAMP, GeneXpert MTB/RIF and culture for diagnosis of pulmonary tuberculosis in The Gambia. Journal of Infection 72:332-7.
17. Brent AJ, Mugo D, Musyimi R, Mutiso A, Morpeth SC, Levin M, Scott JAG. 2017. Bacteriological diagnosis of childhood TB: a prospective observational study. Scientific Reports 7:11808.
18. Bunyasi EW, Tameris M, Geldenhuys H, Schmidt BM, Luabeya AK, Mulenga H, Scriba TJ, Hanekom WA, Mahomed H, McShane H, Hatherill M. 2015. Evaluation of Xpert R MTB/RIF Assay in Induced Sputum and Gastric Lavage Samples from Young Children with Suspected Tuberculosis from the MVA85A TB Vaccine Trial. PLoS ONE [Electronic Resource] 10:e0141623.

For peer review only - http://bmjopen.bmj.com/site/about/guidelines.xhtml

19. Chipinduro M, Mateveke K, Makamure B, Ferrand RA, Gomo E. 2017. Stool Xpert^R MTB/RIF test for the diagnosis of childhood pulmonary tuberculosis at primary clinics in Zimbabwe. International Journal of Tuberculosis & Lung Disease 21:161-166.
20. Chisti MJ, Ahmed T, Pietroni MA, Faruque AS, Ashraf H, Bardhan PK, Hossain I, Das SK, Salam MA. 2013. Pulmonary tuberculosis in severely-malnourished or HIV-infected children with pneumonia: a review. Journal of Health, Population & Nutrition 31:308-13.
21. Das A, Anupurba S, Mishra OP, Banerjee T, Tripathi R. 2019. Evaluation of Xpert MTB/RIF Assay for Diagnosis of Tuberculosis in Children. Journal of Tropical Pediatrics 65:14-20.
22. Dayal R, Yadav A, Agarwal D, Kumar M, Kamal R, Singh D, Bhatnagar S. 2020. Comparison of Diagnostic Yield of Tuberculosis Loop-Mediated Isothermal Amplification Assay With Cartridge-Based Nucleic Acid Amplification Test, Acid-Fast Bacilli Microscopy, and Mycobacteria Growth Indicator Tube Culture in Children With Pulmonary Tuberculosis. Journal of the Pediatric Infectious Diseases Societ 10:10.
23. Elhassan MM, Elmekki MA, Osman AL, Hamid ME. 2016. Challenges in diagnosing tuberculosis in children: a comparative study from Sudan. International Journal of Infectious Diseases 43:25-29.
24. Eliso E, Medhin G, Belay M. 2015. Prevalence of smear positive pulmonary tuberculosis among outpatients presenting with cough of any duration in Shashogo Woreda, Southern Ethiopia. BMC Public Health 15:112.
25. Fekadu L, Hanson C, Osberg M, Makayova J, Mingkwan P, Chin D. 2017. Increasing Access to Tuberculosis Services in Ethiopia: Findings From a Patient-Pathway Analysis. J Infect Dis 216:S696-S701.
26. Garcia-Basteiro AL, Lopez-Varela E, Augusto OJ, Gondo K, Munoz J, Sacarlal J, Marais B, Alonso PL, Ribo JL. 2015. Radiological findings in young children investigated for tuberculosis in Mozambique. PLoS ONE [Electronic Resource] 10:e0127323.
27. Giang do C, Duong TN, Ha DT, Nhan HT, Wolbers M, Nhu NT, Heemskerk D, Quang ND, Phuong DT, Hang PT, Loc TH, Lan NT, Dung NH, Farrar J, Caws M. 2015. Prospective evaluation of GeneXpert for the diagnosis of HIV- negative pediatric TB cases. BMC Infectious Diseases 15:70.
28. Gous N, Scott LE, Khan S, Reubenson G, Coovadia A, Stevens W. 2015. Diagnosing childhood pulmonary tuberculosis using a single sputum specimen on Xpert MTB/RIF at point of care. South African Medical Journal Suid-Afrikaanse Tydskrif Vir Geneeskunde 105:1044-8.
29. Hanrahan CF, Dansey H, Mutunga L, France H, Omar SV, Ismail N, Bassett J, Van Rie A. 2019. Diagnostic strategies for childhood tuberculosis in the context of primary care in a high burden setting: the value of alternative sampling methods. Paediatrics & international Child Health 39:88-94.
30. Kabir S, Uddin MKM, Chisti MJ, Fannana T, Haque ME, Uddin MR, Banu S, Ahmed T. 2018. Role of PCR method using IS6110 primer in detecting Mycobacterium tuberculosis among the clinically diagnosed childhood tuberculosis patients at an urban hospital in Dhaka, Bangladesh. International Journal of Infectious Diseases 68:108-114.
31. Kabir S, Rahman SMM, Ahmed S, Islam MS, Banu RS, Shewade HD, Thekkur P, Anwar S, Banu NA, Nasrin R, Uddin MKM, Choudhury S, Ahmed S, Paul KK, Khatun R, Chisti MJ, Banu S. 2020. Xpert Ultra assay on stool to diagnose pulmonary tuberculosis in children. Clinical Infectious Diseases 18:18.
32. Kalra A, Parija D, Raizada N, Sachdeva KS, Rao R, Swaminathan S, Khanna A, Chopra KK, Hanif M, Singh V, Umadevi KR, Sheladia KN, Rao R, Vasundhara N, S A, A RN, Azeem A, Chhajlani V, Khurana J, Das NJ, Choudhury B, Nair SA, Mall S, Sen R, Chadha SS, Denkinger CM, Boehme C, Sarin S. 2020. Upfront Xpert MTB/RIF for diagnosis of pediatric TB-Does it work? Experience from India. PLoS ONE [Electronic Resource] 15:e0236057.
33. Kalu EI, Ojide CK, Ugochukwu NV. 2013. Gastric aspirate smear microscopy as a diagnostic tool for childhood pulmonary tuberculosis. Annals of Tropical Medicine and Public Health 6:608-613.
34. Lopez-Varela E, Augusto OJ, Gondo K, Garcia-Basteiro AL, Fraile O, Ira T, Ribo Aristizabal JL, Bulo H, Munoz Gutierrez J, Aponte J, Macete E, Sacarlal J, Alonso PL. 2015. Incidence of Tuberculosis Among Young Children in Rural Mozambique. Pediatric Infectious Disease Journal 34:686-92.
35. Marais BJ, Gie RP, Hesseling AC, Schaaf HS, Lombard C, Enarson DA, Beyers N. 2006. A refined symptom-based approach to diagnose pulmonary tuberculosis in children. Pediatrics 118:E1350-E1359.
36. Moussa H, Bayoumi FS, Mohamed AM. 2016. Gene Xpert for Direct Detection of Mycobacterium Tuberculosis in Stool Specimens from Children with Presumptive Pulmonary Tuberculosis. Annals of Clinical & Laboratory Science 46:198-203.

For peer review only

For peer review only - http://bmjopen.bmj.com/site/about/guidelines.xhtml

37. Mukherjee A, Singh S, Lodha R, Singh V, Hesseling AC, Grewal HM, Kabra SK, Delhi Pediatric TBSG. 2013. Ambulatory gastric lavages provide better yields of Mycobacterium tuberculosis than induced sputum in children with intrathoracic tuberculosis. Pediatric Infectious Disease Journal 32:1313-7.
38. Mulenga H, Moyo S, Workman L, Hawkridge T, Verver S, Tameris M, Geldenhuys H, Hanekom W, Mahomed H, Hussey G, Hatherill M. 2011. Phenotypic variability in childhood TB: implications for diagnostic endpoints in tuberculosis vaccine trials. Vaccine 29:4316-21.
39. Mulenga H, Tameris MD, Luabeya KKA, Geldenhuys H, Scriba TJ, Hussey GD, Mahomed H, Landry BS, Hanekom WA, McShane H, Hatherill M. 2015. The role of clinical symptoms in the diagnosis of intrathoracic tuberculosis in young children. Pediatric Infectious Disease Journal 34:1157-1162.
40. Munoz-Sellart M, Yassin MA, Tumato M, Merid Y, Cuevas LE. 2009. Treatment outcome in children with tuberculosis in southern Ethiopia. Scandinavian Journal of Infectious Diseases 41:450-5.
41. Mwangwa F, Chamie G, Kwarisiima D, Ayieko J, Owaraganise A, Ruel TD, Plenty A, Tram KH, Clark TD, Cohen CR, Bukusi EA, Petersen M, Kamya MR, Charlebois ED, Havlir DV, Marquez C. 2017. Gaps in the Child Tuberculosis Care Cascade in 32 Rural Communities in Uganda and Kenya. Journal of Clinical Tuberculosis and Other Mycobacterial Diseases 9:24-29.
42. Myo K, Zaw M, Swe TL, Kyaw YY, Thwin T, Myo TT, Aye KO, Myint AA. 2018. Evaluation of Xpert MTB/RIF assay as a diagnostic test for pulmonary tuberculosis in children in Myanmar. International Journal of Tuberculosis and Lung Disease 22:1051-1055.
43. Nansumba M, Kumbakumba E, Orikiriza P, Muller Y, Nackers F, Debeaudrap P, Boum Y, 2nd, Bonnet M. 2016. Detection Yield and Tolerability of String Test for Diagnosis of Childhood Intrathoracic Tuberculosis. Pediatric Infectious Disease Journal 35:146-51.
44. Negash H, Legese H, Adhanom G, Mardu F, Tesfay K, Gebremeskel SG, Berhe B. 2020. Six years trend analysis of tuberculosis in Northwestern Tigrai, Ethiopia; 2019: A retrospective study. Infection and Drug Resistance 13:643-649.
45. Nhu NT, Ha DT, Anh ND, Thu DD, Duong TN, Quang ND, Lan NT, Quyet TV, Tuyen NT, Ha VT, Giang DC, Dung NH, Wolbers M, Farrar J, Caws M. 2013. Evaluation of Xpert MTB/RIF and MODS assay for the diagnosis of pediatric tuberculosis. BMC Infectious Diseases 13:31.
46. Nicol MP, Zar HJ. 2011. New specimens and laboratory diagnostics for childhood pulmonary TB: progress and prospects. Paediatric Respiratory Reviews 12:16-21.
47. Nicol MP, Spiers K, Workman L, Isaacs W, Munro J, Black F, Zemanay W, Zar HJ. 2013. Xpert MTB/RIF testing of stool samples for the diagnosis of pulmonary tuberculosis in children. Clinical Infectious Diseases 57:e18-21.
48. Nicol MP, Wood RC, Workman L, Prins M, Whitman C, Ghebrekristos Y, Mbhele S, Olson A, Jones-Engel LE, Zar HJ, Cangelosi GA. 2019. Microbiological diagnosis of pulmonary tuberculosis in children by oral swab polymerase chain reaction. Scientific Reports 9:10789.
49. Nissen TN, Rose MV, Kimaro G, Bygbjerg IC, Mfinanga SG, Ravn P. 2012. Challenges of loss to follow-up in tuberculosis research. PLoS ONE [Electronic Resource] 7:e40183.
50. Oliwa JN, Gathara D, Ogero M, van Hensbroek MB, English M, Van't Hoog A, Clinical Information N. 2019. Diagnostic practices and estimated burden of tuberculosis among children admitted to 13 government hospitals in Kenya: An analysis of two years' routine clinical data. PLoS ONE [Electronic Resource] 14:e0221145.
51. Orikiriza P, Nansumba M, Nyehangane D, Bastard M, Mugisha IT, Nansera D, Mwanga-Amumpaire J, Boum Y, 2nd, Kumbakumba E, Bonnet M. 2018. Xpert MTB/RIF diagnosis of childhood tuberculosis from sputum and stool samples in a high TB-HIV-prevalent setting. European Journal of Clinical Microbiology & Infectious Diseases 37:1465-1473.
52. Pearce EC, Woodward JF, Nyandiko WM, Vreeman RC, Ayaya SO. 2012. A systematic review of clinical diagnostic systems used in the diagnosis of tuberculosis in children. Aids Research and Treatment:401896.
53. Ramos JM, Perez-Butragueno M, Tisiano G, Yohannes T, Reyes F, Gorgolas M. 2013. Evaluation of Ziehl-Neelsen smear for diagnosis of pulmonary tuberculosis in childhood in a rural hospital in Ethiopia. International Journal of Mycobacteriology 2:171-3.
54. Ramos JM, Perez-Butragueno M, Tesfamariam A, Reyes F, Tiziano G, Endirays J, Balcha S, Elala T, Biru D, Comeche B, Gorgolas M. 2019. Comparing tuberculosis in children aged under 5 versus 5 to 14 years old in a rural hospital in southern Ethiopia: an 18-year retrospective cross-sectional study. BMC Public Health 19:856.
55. Raizada N, Sachdeva KS, Nair SA, Kulsange S, Gupta RS, Thakur R, Parmar M, Gray C, Ramachandran R, Vadera B, Ekka S, Dhawan S, Babre A, Ghedia M, Alavadi U, Dewan P, Khetrapal M, Khanna A,

For peer review only - http://bmjopen.bmj.com/site/about/guidelines.xhtml

Boehme C, Paramsivan CN. 2014. Enhancing TB case detection: experience in offering upfront Xpert MTB/RIF testing to pediatric presumptive TB and DR TB cases for early rapid diagnosis of drug sensitive and drug resistant TB. PLoS ONE [Electronic Resource] 9:e105346.

56. Raizada N, Sachdeva KS, Swaminathan S, Kulsange S, Khaparde SD, Nair SA, Khanna A, Chopra KK, Hanif M, Sethi GR, Umadevi KR, Keshav Chander G, Saha B, Shah A, Parmar M, Ghediya M, Jaju J, Boehme C, Paramasivan CN. 2015. Piloting Upfront Xpert MTB/RIF Testing on Various Specimens under Programmatic Conditions for Diagnosis of TB & DR-TB in Paediatric Population. PLoS ONE [Electronic Resource] 10:e0140375.

57. Raizada N, Khaparde SD, Salhotra VS, Rao R, Kalra A, Swaminathan S, Khanna A, Chopra KK, Hanif M, Singh V, Umadevi KR, Nair SA, Huddart S, Prakash CHS, Mall S, Singh P, Saha BK, Denkinger CM, Boehme C, Sarin S. 2018. Accelerating access to quality TB care for pediatric TB cases through better diagnostic strategy in four major cities of India. PLoS ONE [Electronic Resource] 13:e0193194.

58. Raizada N, Khaparde SD, Rao R, Kalra A, Sarin S, Salhotra VS, Swaminathan S, Khanna A, Chopra KK, Hanif M, Singh V, Umadevi KR, Nair SA, Huddart S, Tripathi R, Surya Prakash CH, Saha BK, Denkinger CM, Boehme C. 2018. Upfront Xpert MTB/RIF testing on various specimen types for presumptive infant TB cases for early and appropriate treatment initiation. PLoS ONE [Electronic Resource] 13:e0202085.

59. Reither K, Manyama C, Clowes P, Rachow A, Mapamba D, Steiner A, Ross A, Mfinanga E, Sasamalo M, Nsubuga M, Aloi F, Cirillo D, Jugheli L, Lwilla F. 2015. Xpert MTB/RIF assay for diagnosis of pulmonary tuberculosis in children: a prospective, multi-centre evaluation. Journal of Infection 70:392-9.

60. Sabi I, Kabyemera R, Mshana SE, Kidenya BR, Kasanga G, Gerwing-Adima LE, Meremo A, Clowes P, Rachow A, Peck RN. 2016. Pulmonary TB bacteriologically confirmed by induced sputum among children at Bugando Medical Centre, Tanzania. International Journal of Tuberculosis & Lung Disease 20:228-34.

61. Sabi I, Rachow A, Mapamba D, Clowes P, Ntinginya NE, Sasamalo M, Kamwela L, Haraka F, Hoelscher M, Paris DH, Saathoff E, Reither K. 2018. Xpert MTB/RIF Ultra assay for the diagnosis of pulmonary tuberculosis in children: a multicentre comparative accuracy study. Journal of Infection 77:321-327.

62. Sekadde MP, Wobudeya E, Joloba ML, Ssengooba W, Kisembo H, Bakeera-Kitaka S, Musoke P. 2013. Evaluation of the Xpert MTB/RIF test for the diagnosis of childhood pulmonary tuberculosis in Uganda: a cross-sectional diagnostic study. BMC Infectious Diseases 13:133.

63. Sharma S, Sarin R, Sahu G, Shukla G. 2020. Demographic profile, clinical and microbiological predictors of mortality amongst admitted pediatric TB patients in a tertiary referral tuberculosis hospital. Indian Journal of Tuberculosis 67:312-319.

64. Shata AMA, Coulter JBS, Parry CM, Chingani G, Broadhead RL, Hart CA. 1996. Sputum induction for the diagnosis of tuberculosis. Archives of Disease in Childhood 74:535-537.

65. Singh M, Sethi GR, Mantan M, Khanna A, Hanif M. 2016. Xpert MTB/RIF assay for the diagnosis of pulmonary tuberculosis in children. International Journal of Tuberculosis and Lung Disease 20:839-843.

66. Sorsa A, Jerene D, Negash S, Habtamu A. 2020. Use of Xpert Contributes to Accurate Diagnosis, Timely Initiation, and Rational Use of Anti-TB Treatment Among Childhood Tuberculosis Cases in South Central Ethiopia. Pediatric Health Medicine & Therapeutics 11:153-160.

67. Ssengooba W, Iragena JD, Nakiyingi L, Mujumbi S, Wobudeya E, Mboizi R, Boulware D, Meya DB, Choo L, Crook AM, Lebeau K, Joloba M, Demers AM, Cresswell FV, Gibb DM. 2020. Accuracy of Xpert Ultra in Diagnosis of Pulmonary Tuberculosis among Children in Uganda: a Substudy from the SHINE Trial. Journal of Clinical Microbiology 58:24.

68. Surya A, Setyaningsih B, Suryani Nasution H, Gita Parwati C, Yuzwar YE, Osberg M, Hanson CL, Hymoff A, Mingkwan P, Makayova J, Gebhard A, Waworuntu W. 2017. Quality Tuberculosis Care in Indonesia: Using Patient Pathway Analysis to Optimize Public-Private Collaboration. J Infect Dis 216:S724-S732.

69. Swaminathan S, Datta M, Radhamani MP, Mathew S, Reetha AM, Rajajee S, Mathew R, Radkhakrishnan A, Raghu MB. 2008. A profile of bacteriologically confirmed pulmonary tuberculosis in children. Indian Pediatrics 45:743-747.

70. Walters E, Demers AM, van der Zalm MM, Whitelaw A, Palmer M, Bosch C, Draper HR, Gie RP, Hesseling AC. 2017. Stool Culture for Diagnosis of Pulmonary Tuberculosis in Children. Journal of Clinical Microbiology 55:3355-3365.

71. Walters E, van der Zalm MM, Palmer M, Bosch C, Demers AM, Draper H, Goussard P, Schaaf HS, Friedrich SO, Whitelaw A, Warren R, Gie RP, Hesseling AC. 2017. Xpert MTB/RIF on Stool Is Useful for the Rapid Diagnosis of Tuberculosis in Young Children With Severe Pulmonary Disease. Pediatric Infectious Disease Journal 36:837-843.

For peer review only - http://bmjopen.bmj.com/site/about/guidelines.xhtml

72. Walters E, Scott L, Nabeta P, Demers AM, Reubenson G, Bosch C, David A, van der Zalm M, Havumaki J, Palmer M, Hesseling AC, Ncayiyana J, Stevens W, Alland D, Denkinger C, Banada P. 2018. Molecular Detection of Mycobacterium tuberculosis from Stools in Young Children by Use of a Novel Centrifugation-Free Processing Method. Journal of Clinical Microbiology 56:09.
73. Yadav R, Vaidya P, Mathew JL, Singh S, Khaneja R, Agarwal P, Singh M, Sethi S. 2020. Diagnostic accuracy of Xpert MTB/RIF Ultra for detection of Mycobacterium tuberculosis in children: a prospective cohort study. Letters in Applied Microbiology 08:08.
74. Zar HJ, Honslo D, Apolles P, Swingler G, Hussey G. 2005. Induced sputum versus gastric lavage for microbiological confirmation of pulmonary tuberculosis in infants and young children: a prospective study. Lancet 365:130-134.
75. Zar HJ, Workman L, Isaacs W, Munro J, Black F, Eley B, Allen V, Boehme CC, Zemanay W, Nicol MP. 2012. Rapid molecular diagnosis of pulmonary tuberculosis in children using nasopharyngeal specimens. Clinical Infectious Diseases 55:1088-95.
76. Zar HJ, Workman L, Isaacs W, Dheda K, Zemanay W, Nicol MP. 2013. Rapid diagnosis of pulmonary tuberculosis in African children in a primary care setting by use of Xpert MTB/RIF on respiratory specimens: a prospective study. The Lancet Global Health 1:e97-e104.
77. Zar HJ, Workman LJ, Prins M, Bateman LJ, Mbhele SP, Whitman CB, Denkinger CM, Nicol MP. 2019. Tuberculosis Diagnosis in Children Using Xpert Ultra on Different Respiratory Specimens. American Journal of Respiratory & Critical Care Medicine 200:1531-1538.

For peer review only

For peer review only - http://bmjopen.bmj.com/site/about/guidelines.xhtml

Xpert Ultra stool testing to diagnose tuberculosis in children in Ethiopia and Indonesia: a model-based cost-effectiveness analysis.

Appendix 2a: Model parameter estimation

Model structure and description 2

Description of parameters from review 4

Spontaneous sputum expectoration (parameter a) 4

Fraction of TB bacteriologically positive under ideal conditions (parameter Fbc) 6

Prevalence of true TB among presumptive TB 8

Accuracy of clinical assessment in bacteriologically negative TB 13

Accuracy of bacteriological tests 14

Level of initial care-seeking 15

Summary of model parameters from review and distributions 16

Summary of other parameters 16

Parameters in common between countries from previous work 16

Parameters specific to Indonesia 17

Parameters specific to Ethiopia 17

Other parameters based on assumption 17

Description approach to expert opinion 18

References 20

For peer review only - http://bmjopen.bmj.com/site/about/guidelines.xhtml

Model structure and description

The model is implemented as a decision tree that matches our understanding of patient pathways of care. The structure of the tree is shown in Figure A1 along with the names of the probabilities of going down each path, and the names of the costs associated with each node (underneath node names; 0 if no costs are applied). Quantities such as probabilities or costs can depend on 'attributes' of patients entering; here, this means true TB status (yes or no) and child age group (0-4 or 5-14 years). The model calculates mean values of various quantities over the tree for a large number (10 thousand) of sampled input parameters and cohort characteristics (i.e., make up by attribute) to generate a probabilistic sensitivity analysis that is used for the generation of results. The quantities calculated over the tree are: the number of deaths; the cost to healthcare providers; the number of referrals; the number of assessments performed; the number of bacteriological assessments performed; the number of anti-TB treatments; the number of bacteriologically-confirmed anti-TB treatments; the number of anti-TB treatments initiated at PHC level; the number of anti-TB treatments initiated among bacteriologically-confirmed TB cases; a validation variable that should always total 1. The model was implemented in R using the HEdtree package.

All fundamental input parameters are treated as random variables with specified distributions to represent uncertainty. Labelled parameters on Figure A1 may depend in specified ways on these underlying fundamental input parameters. Most parameters appearing as labels in Figure A1 directly correspond to fundamental input parameters, and are named as such in parameter tables. However, there are three classes of exception: 1) parameters describing treatment and non-treatment outcomes; 2) parameters on early stages of the care cascade relating to bacteriological testing; 3) parameters describing the prevalence of attributes in the patient cohort, which are not shown on Figure A1.

The approach to outcomes (class 1 above) are based on previously published work[1] and are recapped below (see Table A9), along with some additional modelling details. This document focuses on the review work to inform new input parameters, many of which are related to parameters in classes 2 and 3 above.

Briefly, we assume that parameters *a* are determined by the ability of children in each age category to spontaneously expectorate sputum, i.e., an attempt to collect a spontaneously expectorate sputum is always made at PHC or hospital. Parameters *b* are based on data on the diagnostic accuracy of stool-approaches, but assume that only a fraction of all children in each age group (*Fbc*) are bacteriologically-confirmable under ideal circumstances. Since diagnostic accuracy is typically reported with respect to confirmed cases, we assume test sensitivity only applied to a fraction of *Fbc* of patients. Parameters *f*, *d*, and *h* for clinical assessments at PHC or hospital are assumed to be the same, and are informed by data we found to inform the diagnostic accuracy for clinical diagnosis of TB in each age group (i.e., these are sensitivity for true TB, and one minus specificity for true not TB). Importantly, we assume that under the intervention, a bacteriologically negative test is always followed up with clinical assessment (i.e., this assessment, which will be made in any case, is able to override a false-negative test result with unchanged sensitivity).

For peer review only - http://bmjopen.bmj.com/site/about/guidelines.xhtml

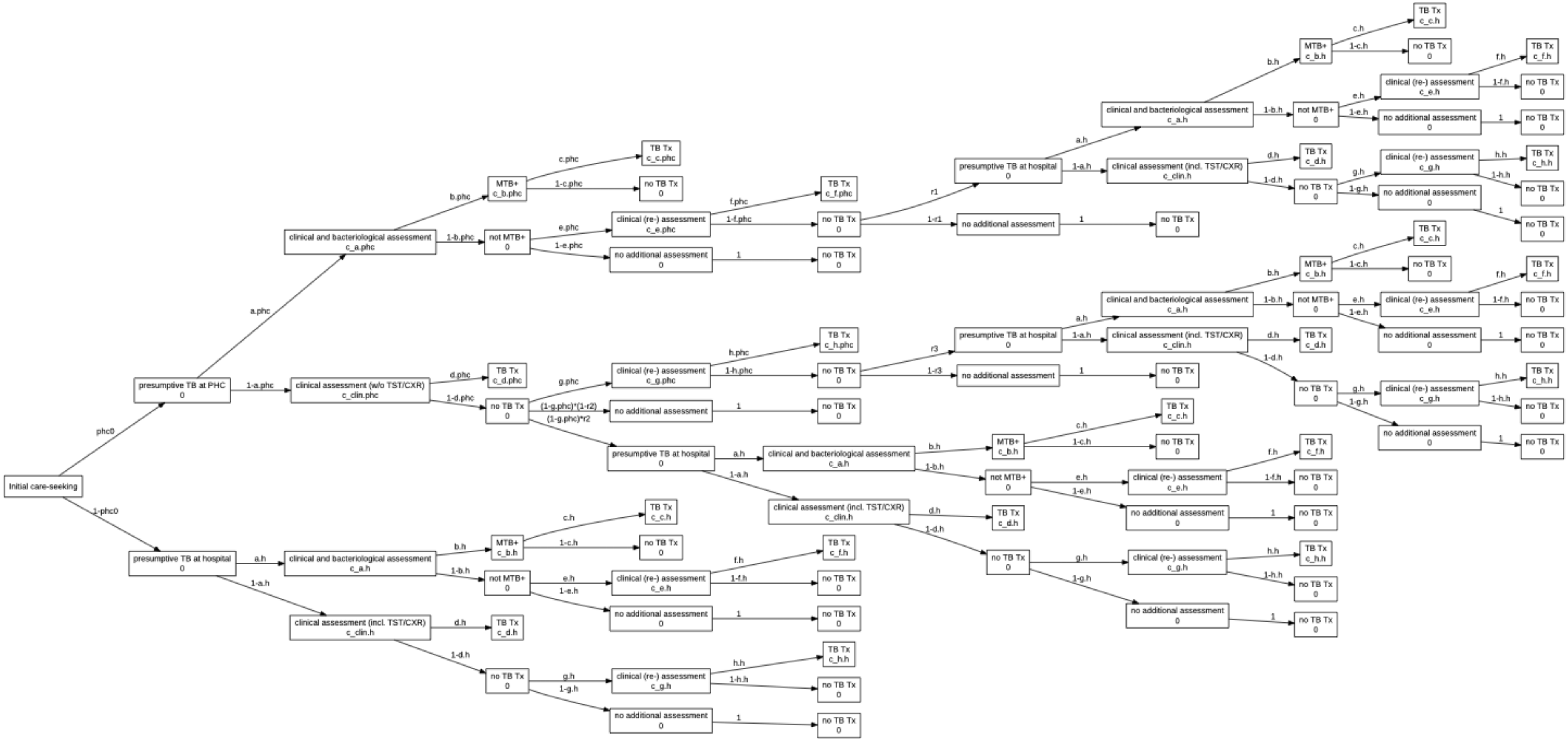

Figure A1 Diagram of modelled decision tree including the edge labels (names of probabilities for each path) and node cost names (underneath node names)

Description of parameters from review

The model was informed with parameters obtained from ongoing studies using the SOS stool method where available, a systematic literature search (see Appendix 1), and expert opinion for those parameters for which no published data was identified. This Appendix provides an overview of the original data and the summation methods used to quantify a parameter for each of the parameters used in the model.

Spontaneous sputum possible

For this parameter, we collected data from our own and published studies on the proportion of children that submitted a spontaneously expectorated sputum sample for diagnostic workup. We included those studies that accepted spontaneously expectorated sputum from all children that could produce such a sample and included other respiratory specimens (i.e., nasogastric (lavage) aspirates, nasopharyngeal aspirates, or induced sputum) for those children unable to spontaneously expectorate, reporting the number of specimens by type received per age group. Only two studies included in our comprehensive review of original peer-reviewed papers (Appendix 1) met these criteria[2, 3].

Table A1 Proportion of children who submitted spontaneously expectorated sputum, by age group.

Reference	Setting	Age group	Number of children	Proportion spontaneous sputum (95% CI if provided)
Kaswandani, Tiemersma et al, unpublished	in- and outpatients with symptoms or signs of presumptive TB in 10 secondary and tertiary care hospitals on Java, Indonesia	0-4 years	222	1.80% (0.67% - 4.72%)
		5-10 years	82	13.41% (7.54% - 22.73%)
Bates et al[2]	in-patients with primary or secondary admission diagnosis of TB at pediatric and child health department of Lusaka University hospital in Zambia	0-4 years	663	2.30%
		5-9 years	124	45.20%
		10-14 years	138	50.00%
Hanrahan et al[3]	outpatients with presumptive TB at 1 primary care clinic in Johannesburg, South Africa	2 months - 4 years	202	3.90%
		5-9 years	17	58.80%

Bates et al[2] collected sputum samples from all children who could expectorate while gastric lavage aspirates were obtained from children unable to expectorate. Hanrahan et al[3] collected a spontaneous sputum sample whenever possible. Sputum collection was guided and overseen by a dedicated paediatrician. If the child was unable to expectorate, one nasopharyngeal aspirate and one induced sputum sample were obtained by a nurse. We also collected relevant data in a study in Indonesia on the diagnostic accuracy of the SOS stool method with Xpert. Sample collection was overseen as per routine procedures in the facilities, but was usually done by a nurse. Collection of a

For peer review only - http://bmjopen.bmj.com/site/about/guidelines.xhtml

spontaneously expectorated sputum or an alternative specimen (either sputum induction (generally for children of 2 years and older) or nasogastric aspiration (for younger children)) was conducted as per nurse's judgement.

Table A1 summarizes the data extracted while Figure A2 plots the same data with 95% confidence intervals, using binomial confidence intervals only where counts were provided.
The proportions from the Indonesian studies were lower than those reported from the two published studies, especially for the older children, but may in fact be closer to the reality on the ground, as in the Indonesian study, no special efforts were undertaken to obtain spontaneous sputum from all children.

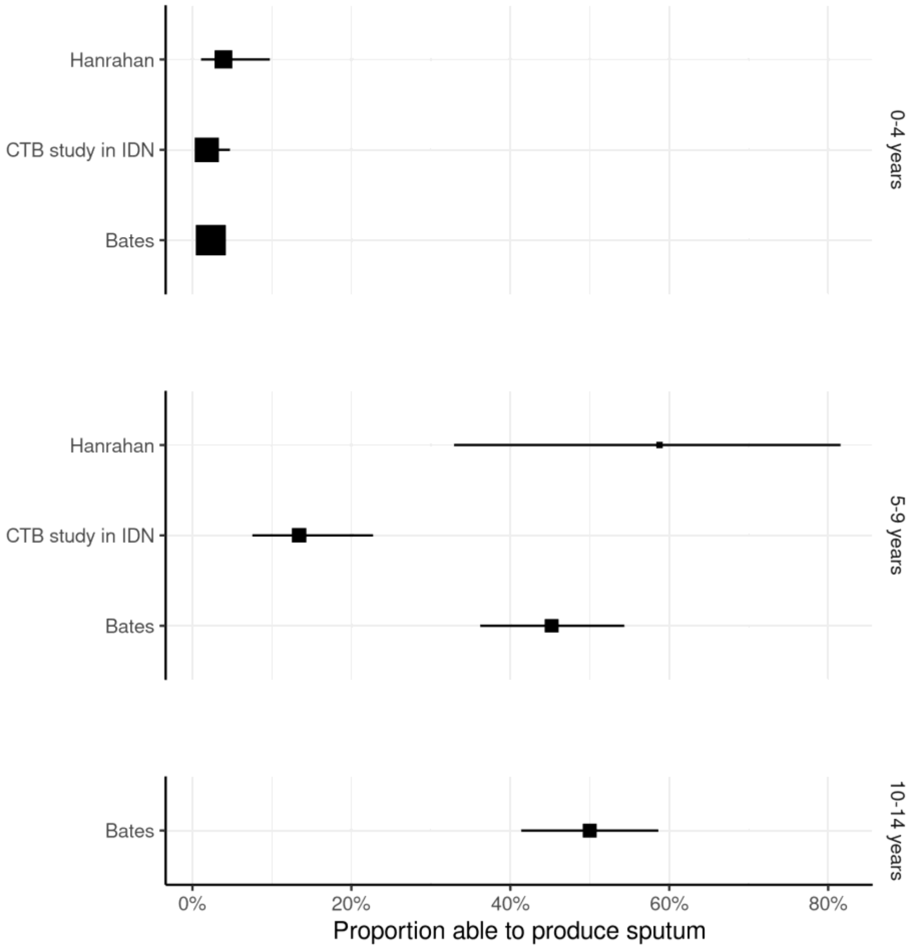

Figure A2. Proportion of children able to spontaneously expectorate sputum by age group with 95% uncertainty intervals.
Names refer to first authors of papers (also described in Table A1 and Appendix 1, Table A2). CTB: Challenge TB project; IDN: Indonesia.

The summary estimates from a random effects meta-analysis after pooling of the results were 2.4% (95% prediction interval (PI): 1.6 - 3.6%) for the rate of spontaneous sputum expectoration among children aged 0-4 years, and 38.9% (95% prediction interval [PI]: 0.098 - 78.8%) among children aged 5 years and above. Note that the 95%CI in this case was much narrower (21.7% - 59.4%).

Fraction of children bacteriologically confirmable

TB in children, especially children under 5 years of age, is often of paucibacillary nature and often no *M. tuberculosis* bacilli can be detected. Most evidence on the sensitivity of diagnostic tests is reported against a gold standard based on bacteriological confirmation; often sensitivity is very poor among children with bacteriologically negative TB. For our model, it was therefore important to understand what the maximum fraction of children for whom TB could be bacteriologically confirmed if an array of diagnostic tests were used. For this parameter, F_{bc}, we included studies that tested multiple different specimens of the same child, using sensitive diagnostics such as Mycobacteria Growth Indicator Tube (MGIT) culture and GeneXpert (Ultra). Four studies meeting these criteria were identified (Table A2). Figure A3 provides the point estimates with 95% uncertainty intervals.

It should be noted that all these four studies were conducted in Cape Town, South Africa, in only 4 different hospitals (Red Cross War Memorial Children's Hospital[4-6], New Somerset Hospital[4-6], Tygerberg Hospital[7] and Karl Bremer Hospital[7]), and included hospitalized children only. Restriction of the study populations to admitted (i.e., most ill) children may introduce bias to higher proportions of confirmable TB, as a positive correlation between bacterial load and seriousness of the illness is expected. For example, in another study including children with minimal TB (defined as non-severe, symptomatic, smear-negative TB), the disease was bacteriologically confirmed on a respiratory sample in only 14.16% of the cases.[8] All children had submitted at least 2 specimens of gastric lavage, gastric washing or sputum, which were tested by culture (MGIT and Lowenstein-Jensen medium), Xpert MTB/Rif and Xpert Ultra.

For peer review only - http://bmjopen.bmj.com/site/about/guidelines.xhtml

Table A2 Fraction of TB that was bacteriologically confirmed from studies applying sensitive diagnostics to multiple specimens.

Reference	Setting	Type and number of specimens taken	Type of diagnostic tests conducted	Number of children enrolled	Number of children treated for TB	Bacteriological confirmation	Fraction with bacteriological confirmation of TB
Nicol et al[4]	children aged <15 years admitted with presumptive pulmonary TB (incl. at least cough of >2weeks plus another sign or symptom) to 2 hospitals in Cape Town, South Africa	2 IS taken at least 4h apart; n=385 with 2 IS, n=67 with one IS specimen	Fluorescent smear microscopy and Xpert MTB/Rif on concentrated sample, MGIT culture	452	n=216: 69/70 definite TB , 147/216 possible TB (incl. 6 with Xpert MTB+ results)	n=76: 70 culture-positive, 6 Xpert positive, culture-negative	34.72%
Walters et al[9]	children aged <13 years presenting to 2 hospitals in Cape Town, South Africa with presumptive intrathoracic TB	sputum (5 years or older)/NGA (<5 years) + IS + NPA), stool (max 7 samples). All respiratory samples tested on smear + MGIT and partly on GX, stool GX	respiratory samples: fluorescent smear microscopy and Xpert MTB/Rif on concentrated sample, MGIT culture if collected by study team. Smear and culture if collected by hospital staff. Stool samples: Xpert and culture (the latter only until half-way the study)	379	n=170: 73 with bacteriologically confirmed TB, 69 with unconfirmed TB, 28 with unlikely TB	n=73: 71 culture-or Xpert positive on respiratory sample, 1 Xpert-positive on stool sample and 1 culture-positive on stool sample	42.94%
Zar et al (2012)[5]	Children aged <15 years with presumptive TB hospitalized in Cape Town, South Africa, because of severe pneumonia, need for oxygen/intravenous therapy, or social conditions precluding home care	2 NPA (taken at least 4h apart) and 2 IS (taken at least 30 min after NPA, and taken at least 4h apart); n=396 with 2 paired IS and NPA, n=139 with 1 paired IS and NPA	Fluorescent smear microscopy and Xpert MTB/Rif on concentrated sample, MGIT culture	535	n=283: 87 with definite TB, 194 with possible TB	n=98: 87 culture-positive and 11 Xpert NPA/IS positive and culture-negative (of whom 9 were treated)	33.92%
Zar et al (2019)[6]	Children aged <15 years, hospitalized for suspected TB in Cape Town, South Africa	2 NPA (taken at least 4h apart) and 2 IS (taken at least 30 min after NPA, and taken at least 4h apart); n=130 with 2 paired IS and NPA, n=65 with 1 paired IS and NPA	Fluorescent smear microscopy and Xpert Ultra (for 2 NPA and 1 IS) on concentrated sample, MGIT culture	195	n=144: 40 with confirmed TB, 104 with unconfirmed TB (not sure though if all were treated)	n=48: 40 culture-positive, between 5 and 9 Xpert Ultra NPA/IS positive*, culture-negative	31.25 - 34.03%*

* The exact number of Xpert-positive, culture-negative cases does not become clear from the paper: there were between 5 (IS and NPA results completely overlap) and 9 (no overlap between IS and NPA results) of such cases.

For peer review only - http://bmjopen.bmj.com/site/about/guidelines.xhtml

The summary estimate from a random effects meta-analysis was 38.0% (95% prediction interval [PI]: 33.1 - 43.1%).

For the above studies, most children were under 5 years of age. The fraction of children with TB in whom it is possible to obtain bacteriological confirmation is thought to be higher for the 5-14 years age group, but we did not find any suitable data to directly inform this. For the 5-14 year old age group, we therefore divided the proportion of children aged 5-14 reported from South African enhanced surveillance data in du Preez et al.[10] by the spontaneous sputum fraction for this age group from above. This assumes that the fraction of children bacteriologically confirmed in routine practice is the product of the fraction who can spontaneously expectorate sputum, and the fraction who would be bacteriologically confirmed with enhanced sample collection and multiple testing (F_{bc}). This yielded an estimate of 58.0% (50.5 - 65.8%) for F_{bc} in this age group.

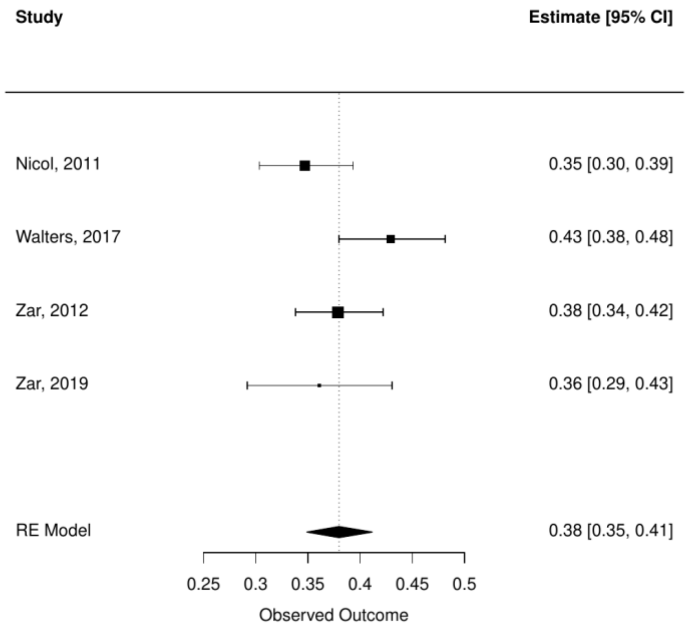

Figure A3. Meta-analytic results for the fraction of children with bacteriologically positive TB under ideal circumstances.

only

Prevalence of true TB in presumptive

For this parameter, from our systematic review (Appendix 1), we selected studies that reported the number of children with presumptive TB and the number of children diagnosed with TB (by method) during the study period.

We restricted to studies that reported using case definitions based on one of the Graham consensus definitions,[11, 12] or the NIH definition, including confirmed/probable TB as TB or the number starting treatment if this was stated (see Table A3). Where age categories reported were not exactly 0-4 years, 5-14 years or 0-14 years, we approximated the age category reported by the studies by its closest match, aggregating over counts if necessary. We performed a random-effects meta-analysis for each age grouping (see Figure A4), finding a summary estimate of 45% (95% prediction interval [PI]: 7.7 - 89%). There was high heterogeneity and wide prediction intervals, with no clear difference between the 0-4 year and 5-14 year age group. We therefore based our parametrization on the pooled 0-14 year analysis, using the midpoint and prediction interval to inform a beta distribution.

For peer review only - http://bmjopen.bmj.com/site/about/guidelines.xhtml

Table A3 Studies reporting TB prevalence among presumptive TB patients using NIH or Graham case-definitions.

Author	Year	Design	Setting	Clinical diagnosis	Specimens and tests	Age group	Included	Diagnosed with TB	Type of diagnosis
Bacha[13]	2017	Retrospective descriptive	children evaluated for presumptive TB and/or referred for TB treatment in 1 regional referral hospital in Southern Highlands Zone of Tanzania serving a child population of 3.2 mln children	as per internationally proposed criteria (see Graham et al. 2015)	SSM and Xpert on sputum or IS if unable to expectorate. Culture only if there was a 2nd sample (89.4% of children)	0-14 years	455	120	21 confirmed, 99 probable, 37 possible TB
Elhassan[14]	2016	Cross-sectional	Children with presumptive TB presenting to 5 TB centers in Khartoum state, Sudan	Confirmed TB: cough>2wks AND culture+, Probable: cough>2wk AND CXR abnormal AND HH contact; Possible: Cough>2wk AND HH contact AND TST+	SSM (ZN and auramine fluorescence), IS6110 PCR and LJ culture on sputum (if 7+y) or NGA (if<7y)	0-18y (0-15y as per Methods but 0-18y per Tables & Figures)	197	125	32 confirmed, 56 probable, 37 possible
								32	LJ culture confirmed
						<=6y	86	47	3 confirmed, 29 probable, 15 possible
								3	LJ culture confirmed
						7-12 y	63	40	10 confirmed, 17 probable, 13 possible
								10	LJ culture confirmed
						13-18y	48	38	19 confirmed, 10 probable, 9 possible
								19	LJ culture confirmed
Giang[15]	2015	Cross-sectional	HIV negative children presenting with presumptive TB at a sub-national TB referral hospital in Ho Chi Minh City, Vietnam	at least 1 symptom suggestive of TB plus a positive culture or smear, or plus CXR suggesting TB, positive response to TB therapy, documented close contact with TB patient, or positive TST	concentrated SSM and Xpert, MGIT culture on an average of 2 samples (NS)	0-14 years	150	131	38 confirmed, 60 probable, 33 possible
							150	38	culture or smear-positive (confirmed) PTB
							150	46	Xpert-positive (among confirmed, probable and possible cases only)
							150	39	culture-positive (among confirmed, probable and possible cases only)
Hanrahan [3]	2019	cross-sectional	children with presumptive TB incl. symptomatic child HH contacts of adult TB patients regardless of symptom duration presenting at a high-volume, primary health-care clinic which provides outpatient care for a densely populated urban and peri-urban impoverished community of about	Per Graham, 2015. Confirmed TB: microbiologically positive by SSM, culture or Xpert on any sample. Unconfirmed TB: no microbiological confirmation but >=2 of CXR suggesting TB, positive response to TB treatment, TB contact history, or TST+.	concentrated FM, Xpert, and MGIT culture on 1 spontaneous sputum sample or 1 NPA+1 IS if unable to produce sputum; 1 stool	60 days to ≤10 years	119	105	4 confirmed, 101 unconfirmed TB
								4	confirmed TB

For peer review only

Author	Year	Design	Setting	Clinical diagnosis	Specimens and tests	Age group	Included	Diagnosed with TB	Type of diagnosis
			200,000–300,000 (18% children <10y)						
Moussa[16]	2016	Cross-sectional	children with clinical signs of PTB presenting at 1 tertiary care hospital in Cairo, Egypt	at least 1 symptom suggestive of TB plus "microbiological confirmation", or plus CXR suggesting TB, positive response to TB therapy, documented close contact with TB patient, or immunological evidence of MTB infection	SSM, LJ culture on 2 (induced) sputum samples, Xpert MTB/Rif on 2 stool samples	1-15 years	115	107	36 confirmed, 61 probable, 10 possible
							115	36	confirmed PTB
						1-5 years	41	38	10 confirmed, 25 probable, 3 possible
						6-15 years	74	69	26 confirmed, 36 probable, 7 possible
						1-5 years	41	10	confirmed PTB
						6-15 years	74	26	confirmed PTB
Myo[17]	2018	Cross-sectional	Children with suspected PTB presenting at tertiary care pediatric hospital in Mandalay, Myanmar	revised NIH classification: culture or Xpert positive, or at least 2 of symptoms/ signs suggesting TB, CXR consistent with TB, TB exposure or immunological evidence of MTB, or a positive response to TB treatment	concentrated SSM, direct Xpert MTB/Rif and LJ culture on 1 GLA	1 month-12 years	231	121	38 confirmed, 83 unconfirmed
							231	38	culture- or Xpert-positive (confirmed) PTB
Nicol[18]	2013	Cross-sectional	Children presenting with presumptive TB at 1 primary healthcare clinic and 1 tertiary care hospital in Cape Town, South Africa	culture-positive or any other started on TB treatment, or not started on TB treatment but with persistent TB suggestive symptoms and signs at 3-month follow-up	concentrated Xpert and MGIT on 2 IS and Xpert testing of 2 aliquots from 1 stool	0-14 years	115	65	17 definite, 48 possible
							115	17	culture-positive (definite) PTB
Nicol[19]	2019	Cross-sectional	Children presenting with presumptive TB at 1 tertiary care hospital in Cape Town, South Africa	culture-positive or any other started on TB treatment, or not started on TB treatment but with persistent TB suggestive symptoms and signs at 3-month follow-up	Xpert and MGIT on 2 IS (2 oral swabs with quantitative PCR, not incl. in diagnosis)	0-14 years	165	121	40 confirmed, 81 unconfirmed
							165	40	culture-positive (confirmed) PTB
Reither[20]	2014	cross-sectional	Children presenting with presumptive TB at 2 research sites in Tanzania and 1 hospital in Kampala, Uganda	symptoms suggestive of TB and AFB+ smear or abnormal CXR suggestive for TB, or CXR not clearly suggesting TB but no alternative Dx and complete resolution of	concentrated SSM, Xpert, MGIT and LJ culture on sputum/IS (1-5 samples per child)	2 months-15 years	451	147	37 confirmed, 48 highly probable, 62 probable PTB
							451	37	culture-positive (confirmed) PTB
						2 months-5 years	211	74	16 confirmed, 26 highly probable, 32 probable PTB
							211	16	culture-positive (confirmed) PTB

For peer review only - http://bmjopen.bmj.com/site/about/guidelines.xhtml

Author	Year	Design	Setting	Clinical diagnosis	Specimens and tests	Age group	Included	Diagnosed with TB	Type of diagnosis
				symptoms/signs on TB treatment		6-10 years	133	39	10 confirmed, 13 highly probable, 16 probable PTB
							133	10	culture-positive (confirmed) PTB
						11-15 years	106	34	11 confirmed, 9 highly probable, 14 probable PTB
							106	11	culture-positive (confirmed) PTB
Sabi[21]	2016	cross-sectional	Children presenting with presumptive TB in 1 zonal hospital in NW Tanzania serving a population of 13 mln. 91% of children admitted to hospital	using 4 different published clinical score charts incl. TST and CXR resutls; but for analysis using Graham et al 2012	FM, Xpert, LJ culture on IS	2 months-12 years	192	40	10 confirmed, 10 probable, 20 possible PTB
							192	10	culture positive (confirmed) PTB
Sorsa[22]	2020	Retrospective document review (historical cross-sectional before- after study)	Children presenting with presumptive TB at Asella Teaching and Referral hospital serving a population of approx. 4 mln in South-Central Ethiopia; Jan 2014-Dec 2017 with Xpert as intervention installed Jan 2016	Confirmed: >=1 TB symptom (cough>=2 wk, contact with TB patient, fever, weight loss, failure to gain weight) and microbiologically confirmed by SSM/Xpert; Probable: >=2 of TB contact history, clinical feature suggesting TB, TST+, CXR abnormal suggesting TB	not specified, but likely direct SSM or Xpert on a sputum sample, not clear if NGA was also done.	<15 y	775	453	142 confirmed, 311 probable
								142	confirmed (SSM/Xpert)
							404 ('before'-period)	254	54 confirmed, 200 probable
								54	confirmed (SSM)
							371 ('after'-period)	199	88 confirmed, 111 probable
								88	confirmed (Xpert)
Walters[9]	2018	prospective cohort	Children (12.5% HIV+) with suspected PTB at two public referral hospitals offering general and specialized pediatric care (Rahima Moosa M&C hospital Johannesburg and Desmond Tutu TB center serving 2 hospitals in Cape Town)	per Graham, 2015	SSM, Xpert and MGIT culture on 1-2 respiratory specimens (1 spontaneous sputum or IS, + 1 GA in subset of children aged <5)	no range provided; median 15.5 month, IQR, 10.9–24.3 months	148	42	treated for TB
								63	3 confirmed, 60 unconfirmed
								3	confirmed TB (culture or Xpert+)
								2	Culture+
Walters , van der Zalm[7]	2017	prospective	Children with suspected intrathoracic TB presenting to Tygerberg Hospital and Karl Bremer Hospital in Cape Town, South Africa, Apr 2012-Aug 2015	per Graham, 2015	2x(Sputum/GA + IS + NPA), stool (max 7 samples). All respiratory samples tested on FM + MGIT and partly on GX, stool GX	<13 y; med: 15.9 months IQR 9.2-29.3	379	258	73 confirmed, 185 unconfirmed TB
								73	confirmed TB (71 detected on culture/Xpert non-stool samples, 1 on stool culture, 1 on stool Xpert)
								71	confirmed TB (on non-stool samples)
								170	TB treatment initiated (reference standard)
Zar[23]	2013	prospective					384	197	30 definite, 167 possible TB

For peer review only - http://bmjopen.bmj.com/site/about/guidelines.xhtml

Author	Year	Design	Setting	Clinical diagnosis	Specimens and tests	Age group	Included	Diagnosed with TB	Type of diagnosis
			Children presenting with suspected pulmonary TB at 1 primary care clinic in Khayelitsha, Cape Town, South Africa, Aug 2010-Jul 2012	definite TB: culture+; Possible TB: receiving TB treatment + all whose symptoms/ signs at FU did not resolve if not receiving TB treatment	concentrated FM, concentrated Xpert, MGIT culture on IS+NPA: 80% 2 paired IS+NPA; 20% 1 paired IS+NPA	<15 y; median 38.3 m (IQR:21.2-56.5)		30	definite TB
								180	started on TB treatment

For peer review only

For peer review only - http://bmjopen.bmj.com/site/about/guidelines.xhtml

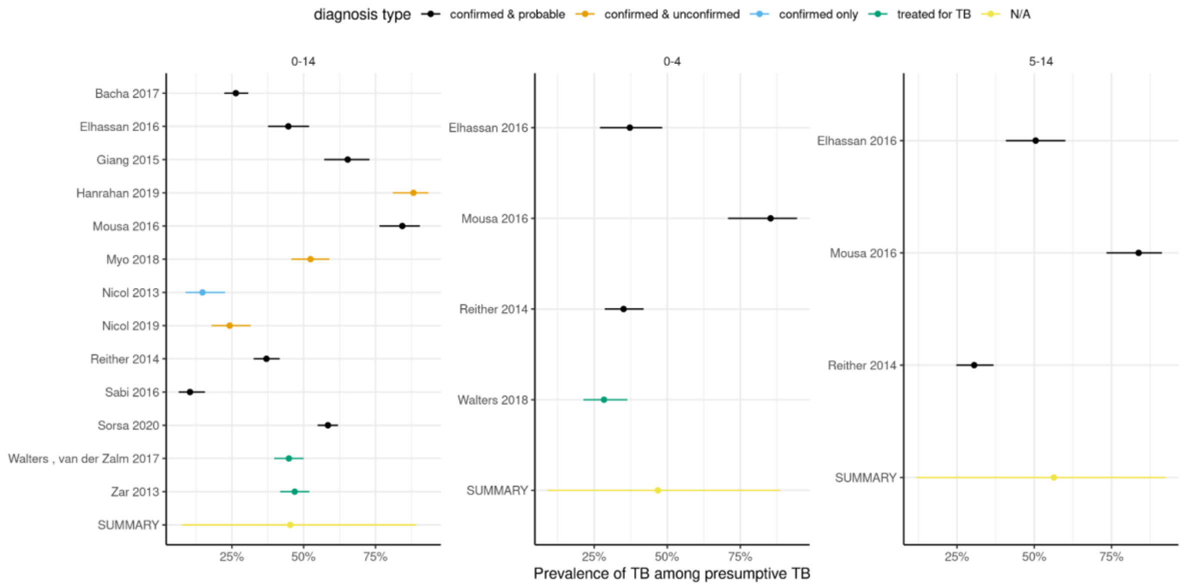

Figure A4 Forest plots for age groups 0-14 years, 0-4 years and 5-14 years of the prevalence of TB among presumptive TB

For peer review only

Accuracy of clinical assessment in bacteriologically negative TB

Sensitivity and specificity of clinical diagnosis

Assessing the diagnostic accuracy of clinical diagnosis for TB in children under routine care is challenging given the absence of a gold standard. Pearce et al.[24] systematically reviewed the accuracy of score-based approaches to diagnosing TB in children, and found one study (van Beekhuizen[25]) which can be interpreted as giving a sensitivity assessment of 62%, and specificity of 95%. The more recent cohort study by Marais et al[26] suggested a sensitivity of 62.6% and specificity of 89.8% among 428 children aged ≤13 years investigated for TB in South Africa. Restricting to children under 3 or those living with HIV, sensitivity was lower: sensitivity 51.8% (specificity 92.5%) for HIV-negative children under 3 years of age; sensitivity of 56.2% (specificity 61.8%) for children living with HIV.

The study of Beneri et al.[27] compared two case definitions within a trial context. Excluding bacteriologically confirmed TB, and counting NIH-unlikely TB as negative gives the cross-tabulation in Table A4.

Table A4 Aggregated data from Beneri et al.[27]

Classification for bacteriologically negative children		National Institute of Health (NIH)	
		TB+	TB-
P1041	TB+	93	1
	TB-	44	7

For peer review only - http://bmjopen.bmj.com/site/about/guidelines.xhtml

Evaluating the stricter trial (P1041) against the NIH case definition yields a sensitivity of 67.9% and a specificity of 87.5%.

A recent paper by Gunasekera et al.[28] developed an optimized scoring approach to TB diagnosis in children in South Africa, and reported a sensitivity of 71.5% when restricting the tool to inputs only from clinical evaluation (i.e. excluding Xpert MTB/Rif and chest X-ray).

We also considered the WHO estimated case-detection ratio for TB in each country and age group (typically 40-50% in relevant contexts). This approach is problematic because these estimates have large uncertainty, and also because CDR factors in children who did not present for care and who were diagnosed but not reported. It therefore is likely a lower bound for the sensitivity of TB detection algorithms in each country.

Given these data, and the likelihood that trials and optimized diagnostic scores may overestimate accuracy, we opted to use the estimates from Marais et al.[26] We used the estimate for HIV-negative children under 3 for all children under 5, and the overall estimate for children aged 5-14 years (see Table A5).

Table A5 Parameters used for accuracy of clinical diagnosis

**Name**	**Distribution**	**Description**	**Source**	**Mean (IQR)**
spec.clinu3	B(83.25,6.75)	Specificity of clinical dx < 5 years	Marais 2006[26]	0.928 (0.908 - 0.945)
sens.clinu3	B(46.62,43.38)	Sensitivity of clinical dx < 5 years	Marais 2006[26]	0.518 (0.482 - 0.554)
spec.clin	B(80.82,9.18)	Specificity of clinical dx 5-14 years	Marais 2006[26]	0.901 (0.878 - 0.921)
sens.clin	B(56.34,33.66)	Sensitivity of clinical dx 5-14 years	Marais 2006[26]	0.627 (0.592 - 0.661)

Accuracy of bacteriological tests

Sensitivity and specificity of Xpert and smear microscopy on sputum, and of Xpert on stool

For diagnostic tests other than stool, we used the accuracies quoted by the systematic review of Detjen et al.,[29] see Table A6.

Table A6 Diagnostic accuracies reported by Detjen et al.[29]

**Diagnostic**	**Sample**	**Reference**	**Sensitivity (95% CI & PI)**	**Specificity (95% CI & PI)**
Xpert	Expectorated/Induced Sputum	Culture	62% (51–73; 30–87)	98% (97–99; 90–100)
Xpert	Gastric lavage	Culture	66% (51–81; 33–91)	98% (96–99; 91–100)
Xpert	Expectorated /Induced Sputum	Clinical for C-ve	2% (1–3; 0–6)	100% (99–100; 99–100)
Microscopy	Expectorated	Culture	26% (14–39; 4–69)	100% (99–100; 94–100)

For peer review only - http://bmjopen.bmj.com/site/about/guidelines.xhtml

	/Induced Sputum			
Microscopy	Gastric lavage	Culture	22% (12–35; 6–51)	99% (97–100; 93–100)

For the accuracy of stool for diagnosing TB in children, we used the two systematic reviews and meta-analyses: MacLean et al.[30] and Mesman et al.[31] (Table A7).

Table A7 Parameters found by review on diagnostic test accuracy

**Name**	**Distribution**	**Description**	**Source**	**Mean (IQR)**
sens.stool	B(20.39,15.3)	Sensitivity of Xpert on stool in bac+ children	Mesman 2019[31]	0.571 (0.515 - 0.627)
spec.stool	B(326.97,6.67)	Specificity of stool in bac+ children	Mesman 2019[31]	0.981 (0.975 - 0.986)
sens.xpert	B(45.75,28.04)	Sensitivity for C+ of Xpert on sputum	Detjen 2015[29]	0.621 (0.582 - 0.659)
spec.xpert	B(736.91,15.03)	Specificity for C+ of Xpert on sputum	Detjen 2015[29]	0.980 (0.977 - 0.984)
sens.sm	B(12.03,34.26)	Sensitivity for C+ of SM on sputum	Detjen 2015[29]	0.257 (0.215 - 0.302)
spec.sm	B(759.66,3.81)	Specificity for C+ of SM on sputum	Detjen 2015[29]	0.995 (0.994 - 0.997)

Level of initial care-seeking

We found no data specific to paediatric TB to inform the proportion of children initially seeking care at primary healthcare level. We ultimately relied on estimates of initial care seeking for Ethiopia and Indonesia made in two TB patient pathway analysis (PPA) papers, namely Fekadu et al.[32] and Surya et al.[33] We included care sought in the private and public sectors, mapping primary care level to the levels L0 and L1 defined in the papers. The former suggested 89.6% of children initially seek care at primary level in Ethiopia; the latter that 92.8% of children initially seek care at primary level in Indonesia. In the absence of data to inform uncertainty, and given the quality of this evidence for our question, we assumed the 95% uncertainty interval was at +/- 10% points around the central estimate.

For peer review only - http://bmjopen.bmj.com/site/about/guidelines.xhtml

Summary of model parameters from review and distributions

Table A8 Parameters informed by analyses above

Name	Distribution	Description	Source	Mean (IQR)
spont.sput.u5	B(21.572,877.28)	Spontaneous sputum possible (0-4)	see methods	0.024 (0.020 - 0.027)
spont.sput.o5	B(2.59,4.07)	Spontaneous sputum possible (5-14)	see methods	0.377 (0.254 - 0.512)
Fbc.u5	B(137.19,223.84)	Fraction of children bacteriologically confirmable <5	see methods	0.380 (0.363 - 0.397)
Fbc.o5	B(97.76,45.12)	Fraction of children bacteriologically confirmable 5-14	see methods	0.684 (0.659 - 0.711)
p_truetb	B(3.10,1.85)	Prevalence of true TB in presumptive	see methods	0.625 (0.484 - 0.783)
spec.clinu3	B(83.25,6.75)	Specificity of clinical dx	Marais 2006[26]	0.928 (0.908 - 0.945)
sens.clinu3	B(46.62,43.38)	Sensitivity of clinical dx	Marais 2006[26]	0.518 (0.482 - 0.554)
spec.clin	B(80.82,9.18)	Specificity of clinical dx	Marais 2006[26]	0.901 (0.878 - 0.921)
sens.clin	B(56.34,33.66)	Sensitivity of clinical dx	Marais 2006[26]	0.627 (0.592 - 0.661)
phc0_e	B(31.18,3.62)	Proportion of first care-seeking at PHC for Ethiopia	Fekadu 2017[32]	0.896 (0.777 - 0.973)
phc0_i	B(22.89,1.78)	Proportion of first care-seeking at PHC for Indonesia	Surya 2017[33]	0.928 (0.801 - 0.992)

Summary of other parameters

Parameters in common between countries from previous work

Most the of the CFR parameters are based on Jenkins et al.[1]

Table A9 Parameters informed by the literature review

Name	Distribution	Description	Source	Mean (IQR)
cfrontxY	LN(-3.96,0.64)	CFR children <5 on TB treatment	Jenkins et al 2017[1]	0.019 (0.012 - 0.029)
cfrontxO	LN(-4.82,0.48)	CFR children 5-14 on TB treatment	Jenkins et al 2017[1]	0.008 (0.006 - 0.011)
cfrnotxY	LN(-0.83,0.08)	CFR children <5 without TB treatment	Jenkins et al 2017[1]	0.436 (0.413 - 0.460)
cfrnotxO	LN(-1.90,0.12)	CFR children 5-14 without TB treatment	Jenkins et al 2017[1]	0.149 (0.137 - 0.162)

For peer review only

Parameters specific to Ethiopia

r2 in particular was adjusted upwards after consultation to reflect a low confidence with child TB diagnosis and management at primary level.

Table A10 Parameters specific to Ethiopia not included above

**Name**	**Distribution**	**Description**	**Source**	**Mean (IQR)**
fracu5	B(7.504,12.47)	Fraction of presumptive TB under 5	Based on fraction of WHO TB < 5	0.371 (0.300 - 0.447)
r1	B(1,15)	Referral PHC -> H after clinical re-assessment following bac-	Expert opinion	0.045 (0.019 - 0.088)
r2	B(8,2)	Referral PHC -> H after initial clinical assessment w/o bac	Expert opinion	0.800 (0.728 - 0.899)
g.phc	B(1,15)	Clincial re-assessment, PHC	Expert opinion	0.045 (0.019 - 0.088)

Parameters specific to Indonesia

r1 in particular was adjusted upwards after consultation to reflect a low confidence in bacteriologic testing for child TB.

Table A11 Parameters specific to Indonesia not included above

**Name**	**Distribution**	**Description**	**Source**	**Mean (IQR)**
fracu5	B(69.37,65.49)	Fraction of presumptive TB under 5*	based on fraction of WHO TB < 5	0.514 (0.485 - 0.543)
r1	B(2,8)	Referral PHC -> H after clinical re-assessment following bac-	Expert opinion	0.200 (0.107 - 0.272)
r2	B(5,5)	Referral PHC -> H after initial clinical assessment w/o bac	Expert opinion	0.500(0.391 - 0.607)
g.phc	B(1,15)	Clincial re-assessment, PHC	Expert opinion	0.045 (0.019 - 0.088)

* based on the proportion of TB cases under 5 among all child TB cases (<15y)

Other parameters based on assumption

Note: many of these parameters could potentially be made country-specific, but currently are not.

Table A12 Parameters without direct evidence based on assumptions

**NAME**	**DISTRIBUTION**	**DESCRIPTION**	**SOURCE**	**Mean (IQR)**
c.phc	B(95,5)	Proportion of bacteriologically confirmed children initiating anti-TB treatment, PHC	assumption	0.953 (0.937 - 0.966)
c.h	B(95,5)	Proportion of bacteriologically confirmed children initiating anti-TB treatment, H	assumption	0.953 (0.937 - 0.966)
e.phc	B(1,15)	Clinical re-assessment after bac-, PHC	assumption	0.045 (0.019 - 0.088)
e.h	B(1,15)	Clinical re-assessment after bac-, H	assumption	0.045 (0.019 - 0.088)
g.h	B(1,15)	Clinical re-assessment, H	assumption	0.045 (0.019 - 0.088)

For peer review only - http://bmjopen.bmj.com/site/about/guidelines.xhtml

r3	B(5,5)	Referral PHC -> H after clinical re-assessment w/o bac	assumption	0.500(0.391 - 0.607)

For peer review only

Description approach to expert opinion

For parameters for which no data was found in published literature, our best estimate based on the practical experience from the authors working in Ethiopia (AB and MG) and Indonesia (NK and RT) was included. NK and RT are experienced pediatricians working in large tertiary care settings in Indonesia. Both are active in the TB section of the Indonesian Association of Pediatricians, of which currently is the chairperson. RT has ample research experience in the field of diagnosing childhood TB in primary, secondary and tertiary care settings. AB and MG are both working for the local KCNV office. AB is a pediatrician with experience in the clinical, research and programmatic settings. He is a member of the Ethiopian Pediatric Association. MG is an senior M&E advisor with up-to-date practical experience in rural and urban sites involved in childhood TB projects run by KNCV.

Data was needed to inform parameters on the proportion of children with bacteriologically confirmed TB started on treatment (*c.phc* and *c.h*), the proportion clinically reassessed after initial bacteriological exclusion of TB (*e.phc* and *e.h*), the proportion of children clinically reassessed after short broad-course of antibiotics (*g.phc* and *g.h*), and about referrals from the primary to higher (hospital) levels (*r1*, *r2* and *r3*).

A data collection tool was distributed to the experts, in which per parameter, their best guess and the minimum and maximum value they considered reasonable could be filled (Table). Then, several online sessions were organized. The first session served to explain the data collection tool. The second session served to discuss the completed tool and solve differences in opinion between the experts where needed. A third session was organized to present the model output using the experts' best estimates. In this session, the set of parameters was further adapted to come to model outputs that seemed reasonable for the country.

For peer review only - http://bmjopen.bmj.com/site/about/guidelines.xhtml

References

1. Jenkins, H.E., et al., *Mortality in children diagnosed with tuberculosis: a systematic review and meta-analysis.* The Lancet. Infectious diseases, 2017. **17**(3): p. 285-295.
2. Bates, M., et al., *Assessment of the Xpert MTB/RIF assay for diagnosis of tuberculosis with gastric lavage aspirates in children in sub-Saharan Africa: a prospective descriptive study.* The Lancet Infectious Diseases, 2013. **13**(1): p. 36-42.
3. Hanrahan, C.F., et al., *Diagnostic strategies for childhood tuberculosis in the context of primary care in a high burden setting: the value of alternative sampling methods.* Paediatrics and International Child Health, 2018. **39**(2): p. 88-94.
4. Nicol, M.P., et al., *Accuracy of the Xpert MTB/RIF test for the diagnosis of pulmonary tuberculosis in children admitted to hospital in Cape Town, South Africa: a descriptive study.* The Lancet. Infectious diseases, 2011. **11**(11): p. 819-824.
5. Zar, H.J., et al., *Rapid molecular diagnosis of pulmonary tuberculosis in children using nasopharyngeal specimens.* Clinical infectious diseases : an official publication of the Infectious Diseases Society of America, 2012. **55**(8): p. 1088-1095.
6. Zar, H.J., et al., *Tuberculosis Diagnosis in Children Using Xpert Ultra on Different Respiratory Specimens.* American journal of respiratory and critical care medicine, 2019. **200**(12): p. 1531-1538.
7. Walters, E., et al., *Xpert MTB/RIF on Stool Is Useful for the Rapid Diagnosis of Tuberculosis in Young Children With Severe Pulmonary Disease.* The Pediatric infectious disease journal, 2017. **36**(9): p. 837-843.
8. Ssengooba, W., et al., *Accuracy of Xpert Ultra in Diagnosis of Pulmonary Tuberculosis among Children in Uganda: a Substudy from the SHINE Trial.* Journal of clinical microbiology, 2020. **58**(9): p. e00410-20.
9. Walters, E., et al., *Molecular Detection of Mycobacterium tuberculosis from Stools in Young Children by Use of a Novel Centrifugation-Free Processing Method.* Journal of clinical microbiology, 2018. **56**(9): p. e00781-18.
10. du Preez, K., et al., *The Impact of the Evolving Human Immunodeficiency Virus Response on the Epidemiology of Tuberculosis in South African Children and Adolescents.* Clinical Infectious Diseases, 2021.
11. Graham, S.M., et al., *Evaluation of tuberculosis diagnostics in children: 1. Proposed clinical case definitions for classification of intrathoracic tuberculosis disease. Consensus from an expert panel.* The Journal of infectious diseases, 2012. **205 Suppl 2**(Suppl 2): p. S199-S208.
12. Graham, S.M., et al., *Clinical Case Definitions for Classification of Intrathoracic Tuberculosis in Children: An Update.* Clinical infectious diseases : an official publication of the Infectious Diseases Society of America, 2015. **61Suppl 3**(Suppl 3): p. S179-S187.
13. Bacha, J.M., et al., *Why being an expert - despite xpert -remains crucial for children in high TB burden settings.* BMC infectious diseases, 2017. **17**(1): p. 123-123.
14. Elhassan, M.M., et al., *Challenges in diagnosing tuberculosis in children: a comparative study from Sudan.* International Journal of Infectious Diseases, 2016. **43**: p. 25-29.
15. Giang, D.C., et al., *Prospective evaluation of GeneXpert for the diagnosis of HIV- negative pediatric TB cases.* BMC infectious diseases, 2015. **15**: p. 70-70.
16. Moussa, H.S., F.S. Bayoumi, and A.M.A. Mohamed, *Gene Xpert for direct detection of Mycobacterium tuberculosis in stool specimens from children with presumptive pulmonary tuberculosis.* Annals of Clinical & Laboratory Science, 2016. **46**(2): p. 198-203.
17. Myo, K., et al., *Evaluation of Xpert® MTB/RIF assay as a diagnostic test for pulmonary tuberculosis in children in Myanmar.* The International Journal of Tuberculosis and Lung Disease, 2018. **22**(9): p. 1051-1055.
18. Nicol, M.P., et al., *Xpert MTB/RIF testing of stool samples for the diagnosis of pulmonary tuberculosis in children.* Clinical infectious diseases : an official publication of the Infectious Diseases Society of America, 2013. **57**(3): p. e18-e21.
19. Nicol, M.P., et al., *Microbiological diagnosis of pulmonary tuberculosis in children by oral swab polymerase chain reaction.* Scientific reports, 2019. **9**(1): p. 10789-10789.

For peer review only

20. Reither, K., et al., *Xpert MTB/RIF assay for diagnosis of pulmonary tuberculosis in children: A prospective, multi-centre evaluation.* Journal of Infection, 2015. **70**(4): p. 392-399.
21. Sabi, I., et al., *Pulmonary TB bacteriologically confirmed by induced sputum among children at Bugando Medical Centre, Tanzania.* The International Journal of Tuberculosis and Lung Disease, 2016. **20**(2): p. 228-234.
22. Sorsa, A., et al., *Use of Xpert Contributes to Accurate Diagnosis, Timely Initiation, and Rational Use of Anti-TB Treatment Among Childhood Tuberculosis Cases in South Central Ethiopia.* Pediatric health, medicine and therapeutics, 2020. **11**: p. 153-160.
23. Zar, H.J., et al., *Rapid diagnosis of pulmonary tuberculosis in African children in a primary care setting by use of Xpert MTB/RIF on respiratory specimens: a prospective study.* The Lancet Global Health, 2013. **1**(2): p. e97-e104.
24. Pearce, E.C., et al., *A systematic review of clinical diagnostic systems used in the diagnosis of tuberculosis in children.* AIDS research and treatment, 2012. **2012**: p. 401896-401896.
25. van Beekhuizen, H.J., *Tuberculosis Score Chart in Children in Aitape, Papua New Guinea.* Tropical Doctor, 1998. **28**(3): p. 155-160.
26. Marais, B.J., et al., *A Refined Symptom-Based Approach to Diagnose Pulmonary Tuberculosis in Children.* Pediatrics, 2006. **118**(5): p. e1350-e1359.
27. Beneri, C.A., et al., *Understanding NIH clinical case definitions for pediatric intrathoracic TB by applying them to a clinical trial.* The international journal of tuberculosis and lung disease : the official journal of the International Union against Tuberculosis and Lung Disease, 2016. **20**(1): p. 93-100.
28. Gunasekera, K.S., et al., *Development of a Treatment-decision Algorithm for Human Immunodeficiency Virus–uninfected Children Evaluated for Pulmonary Tuberculosis.* Clinical Infectious Diseases, 2021.
29. Detjen, A.K., et al., *Xpert MTB/RIF assay for the diagnosis of pulmonary tuberculosis in children: a systematic review and meta-analysis.* The Lancet. Respiratory medicine, 2015. **3**(6): p. 451-461.
30. MacLean, E., et al., *Diagnostic Accuracy of Stool Xpert MTB/RIF for Detection of Pulmonary Tuberculosis in Children: a Systematic Review and Meta-analysis.* Journal of clinical microbiology, 2019. **57**(6): p. e02057-18.
31. Mesman, A.W., et al., *Diagnostic accuracy of molecular detection of Mycobacterium tuberculosis in pediatric stool samples: A systematic review and meta-analysis.* Tuberculosis (Edinburgh, Scotland), 2019. **119**: p. 101878-101878.
32. Fekadu, L., et al., *Increasing Access to Tuberculosis Services in Ethiopia: Findings From a Patient-Pathway Analysis.* The Journal of infectious diseases, 2017. **216**(suppl_7): p. S696-S701.
33. Surya, A., et al., *Quality Tuberculosis Care in Indonesia: Using Patient Pathway Analysis to Optimize Public-Private Collaboration.* The Journal of infectious diseases, 2017. **216**(suppl_7): p. S724-S732.

For peer review only

Xpert Ultra stool testing to diagnose tuberculosis in children in Ethiopia and Indonesia: a model-based cost-effectiveness analysis.

Appendix 2b: Overview of cost parameters

Contents

General costing assumptions 3
Identifying resources used 3
Measuring resource utilization 3
Valuation of resources 4
Clinical assessment 4
TB assessment at health centre 4
TB assessment at hospital 5
TST 6
CXR 6
Sample collection 6
Bacteriological assessment 6
Sputum smear microscopy (SSM) examination 6
GeneXpert test 6
Clinical (re-) assessment 7
TB reassessment at health centre 7
TB reassessment at hospital 7
TB treatment 7
TB treatment medications 7
TB treatment follow-up at health centre 7
TB treatment follow-up at hospital 7
Laboratory monitoring 8
Comparison with other cost estimates 8
Costs tables 9
Table A1 Costs for Ethiopia & Indonesia 9
Table A2 Comparison of estimated unit costs for diagnostic visit, sputum collection, smear microscopy examination and XpertMTB/RIF test with recently published Better estimates of the costs of TB control (Value TB) project[13]. 10
References 11

For peer review only - http://bmjopen.bmj.com/site/about/guidelines.xhtml

For peer review only

For peer review only - http://bmjopen.bmj.com/site/about/guidelines.xhtml

General costing assumptions

Identifying resources used

A careful review of the diagnostic and treatment algorithm for childhood TB for Ethiopia (ETH)[1] and Indonesia (IDN)[2] was performed to identify resource use and costs associated with the diagnosis and treatment for childhood TB in the current standard of care (SOC) and under the intervention of using stool with Xpert for the diagnosis of TB **(Figures 1-3)**. The main activities in both the SOC and the intervention included symptom-based screening for TB, clinical evaluation, sample collection, bacteriological examination, radiological examination, empiric antibiotic therapy to exclude other diseases, treatment initiation for diagnosed TB cases, TB treatment follow-up and TB treatment monitoring laboratory tests. The following cost components were identified; health facility visit (for TB screening, rescreening, diagnosis and treatment), sample collection (spontaneous sputum, stool), bacteriological examination (smear microscopy, GeneXpert), and medicines (anti-TB medicines, pyridoxine).

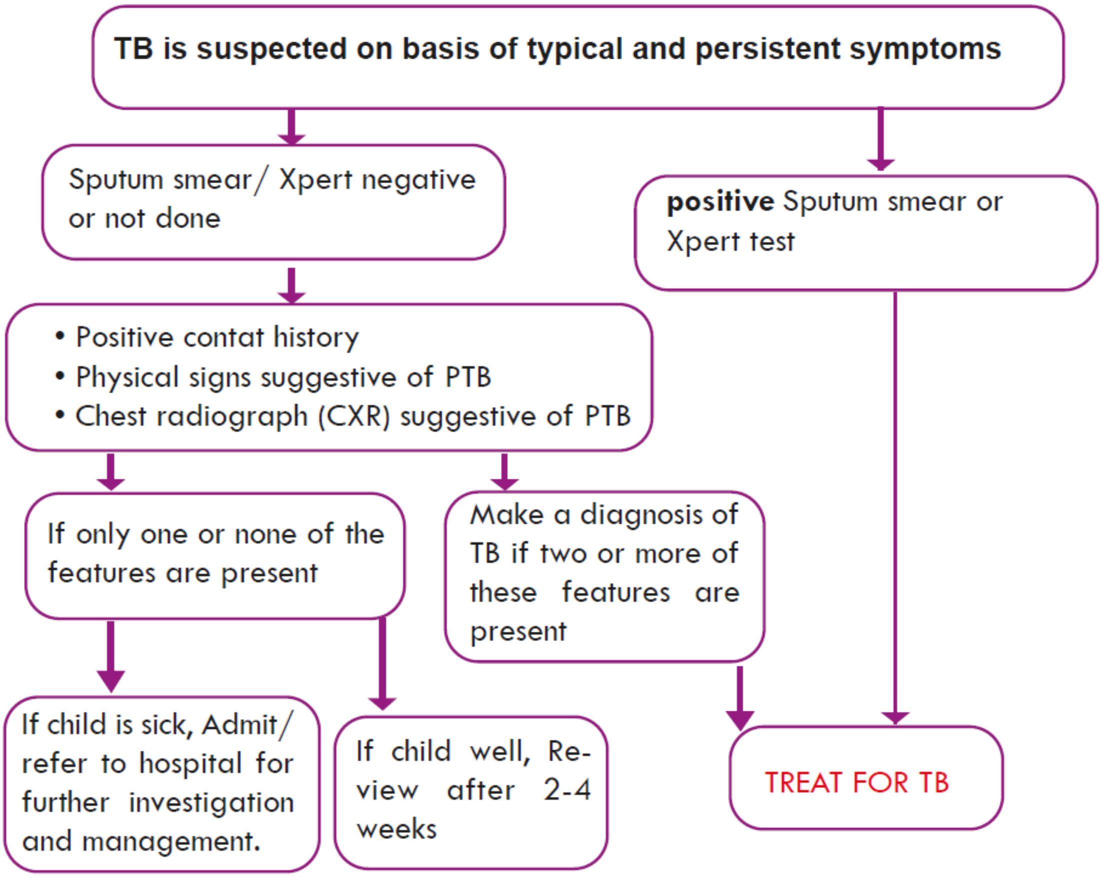

Figure 1. Algorithm for diagnosis of tuberculosis in HIV uninfected children in Ethiopia[1]

Measuring resource utilization

The quantities of each resource type consumed in the diagnostic and treatment algorithm for childhood TB was informed by the national guidelines for the management of childhood TB in each country (**Figures 1-3**). Local TB experts collaborating with KNCV on childhood TB related studies provided country-specific input on resource use.

For peer review only - http://bmjopen.bmj.com/site/about/guidelines.xhtml

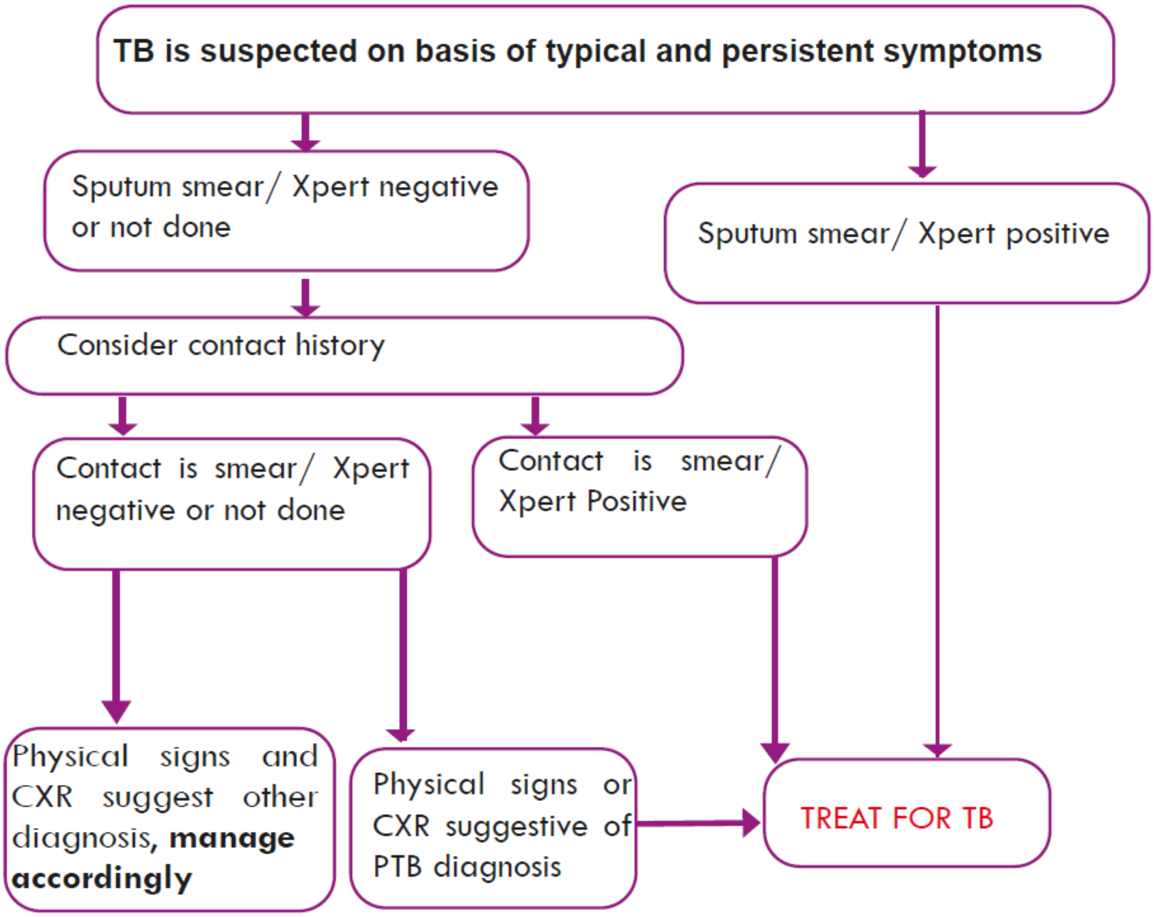

Figure 2. Algorithm for diagnosis of tuberculosis in HIV-infected children in Ethiopia[1]

Valuation of resources

The costs for each resource were estimated by attaching monetary values using relevant unit costs for each country. Summing up these costs (per patient) gives an estimate of the total cost. All costs were estimated from the healthcare provider's perspective and are reported in 2019 USD. Historical costs were adjusted for inflation to 2019 prices using relevant GDP deflators[3] and costs from other countries were transferred to Ethiopia and Indonesia by applying relevant purchasing power parity conversion factors[3]. Costs were assumed to accrue in the present, with no discounting applied. The following costs were estimated.

Clinical assessment

This cost comprises of the cost of clinical assessment to investigate children with presumptive TB and the cost associated with collecting the necessary samples for bacteriological evaluation.

TB assessment at health centre

We assumed the cost for the initial TB assessment at the primary health centre to be equivalent to the country-specific cost of two outpatient visits (range; 1-3 visits) to a health centre with no beds based on WHO-CHOICE estimates[4]. This equates to $10.22 (95% UI; 4.93-15.50) in ETH and $43.35 (95% UI; 19.11-67.59) in IDN.

For peer review only - http://bmjopen.bmj.com/site/about/guidelines.xhtml

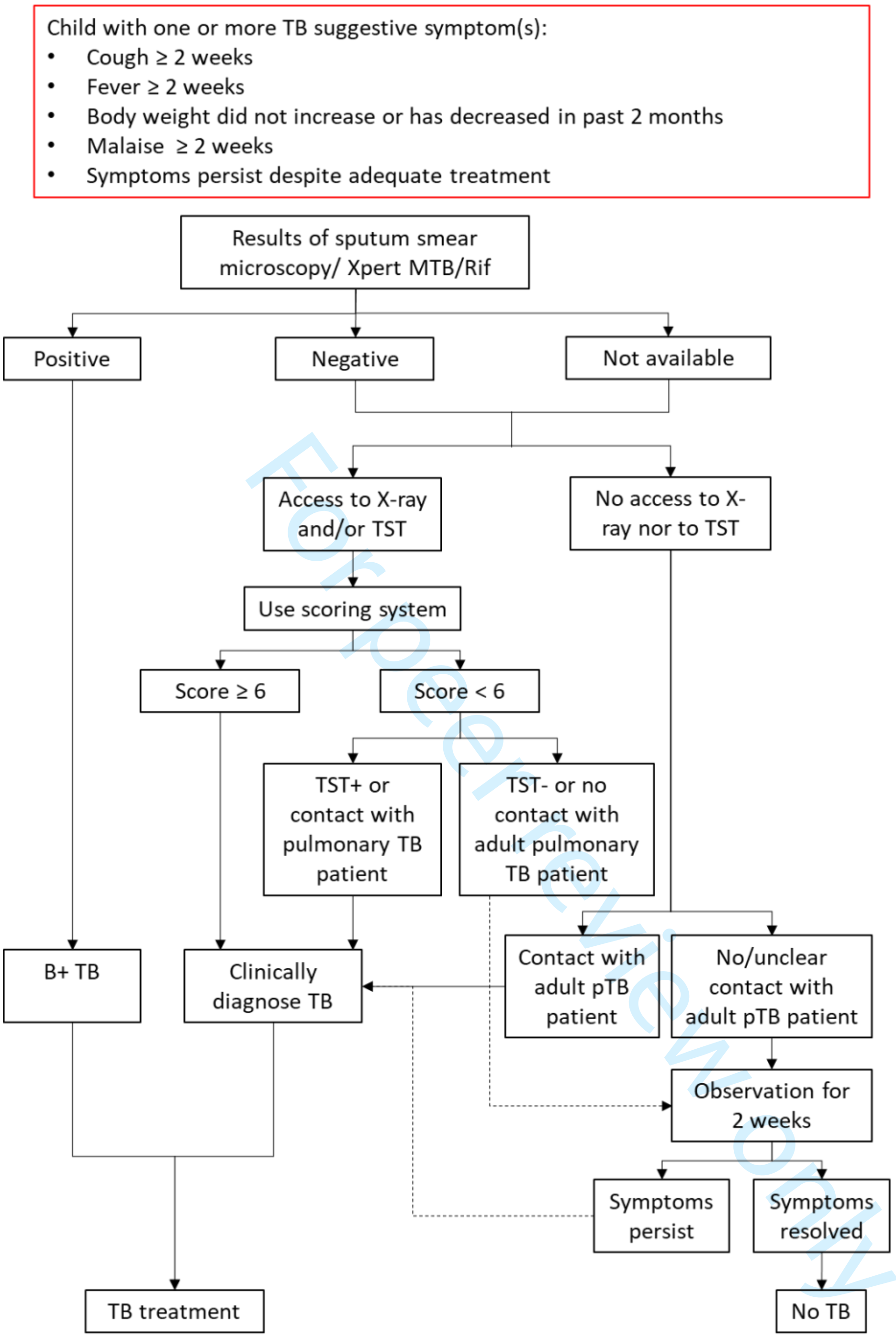

Figure 3. Algorithm for diagnosis of tuberculosis in children in Indonesia[2]

TB assessment at hospital

Similarly, we assumed the cost for the initial TB assessment at the hospital to be equivalent to the country-specific cost of two outpatient visits (range; 1-3 visits) to a primary hospital, defined as a ‘hospitals intended primarily for treating simple cases (e.g. “district hospital”)’ [4]. This results in an estimate of $14.37 (95% UI; 7.79-20.96) in ETH and $61.00 (95% UI; 30.76-91.23) in IDN. The costs of tuberculin skin test (TST) and chest X-ray were separately addressed (see below).

For peer review only - http://bmjopen.bmj.com/site/about/guidelines.xhtml

TST
The Indonesian NTP uses a scoring system for the diagnosis of TB if a bacteriological diagnosis cannot be made (Figure 1). The scoring system uses a combination of tuberculin skin test (TST) results, chest X-ray results (CXR), symptoms and history of contact with TB patients (Table 1). TST and CXR are often not available especially at the primary health centres, hence these costs are currently not modelled.

CXR
The unit cost for CXR for Ethiopia ($8.75) was based on the cost per radiograph reported by the Ethiopian NTP[5]. The unit cost applied for CXR for Indonesia ($11.52) was based on a previous MSH estimate[6]. This cost is applied to a proportion of children assessed at the hospital (80-90%) only since chest X-rays are not available at the primary health centres.

Sample collection
Availability of advanced sample collection procedures is generally limited in both countries, with sputum induction occurring only in big hospitals in Indonesia and nasogastric aspiration only available at big teaching hospitals in Ethiopia. We assumed only self-expectorated sample collection is available and applied the adjusted unit costs for collecting two samples for testing with smear microscopy ($4.64 in ETH and $3.48 in IDN) or one sample for testing with GeneXpert ($2.32 in ETH and $1.74 in IDN) per child based on a study done in adults in South Africa [7]. The unit costs applied for procedures for collecting a single stool sample ($1.67) in the intervention are based on estimates provided by the Paediatric Operational Sustainability Expertise Exchange group (POSEE group) [8]. The POSEE group developed a budgeting tool to assist national TB programs in estimating the costs related to the procurement of devices and consumables needed for sample collection in the paediatric population. These POSEE group cost estimates currently exclude staff, space, training, sample transportation and overheads costs.

Bacteriological assessment
The cost for bacteriological assessment for TB comprises of the costs of testing using either a sputum smear microscopy (SSM) examination or a single GeneXpert test, depending on the availability of the test at each level of care (primary health centre versus hospital) in Ethiopia and Indonesia. Bacteriological testing with the GeneXpert is not widely available in both countries and most testing centres use sputum smear microscopy while some centres refer samples to a GeneXpert testing facility. We therefore assumed sole use of smear microscopy in both countries in the standard of care where two samples are collected for testing in the base case.

Sputum smear microscopy (SSM) examination
The unit cost for SSM for Ethiopia was based on the microscopy cost per test ($1.50) reported by the Ethiopian NTP[5] resulting in an adjusted cost of $1.69. The unit cost for SSM in Indonesia ($3.77) was based on a previous MSH estimate[6].

GeneXpert test
The unit cost for the GeneXpert test was estimated based on country specific data available from the OneHealth Tool[9]. These data include staff times, staff salaries, the Xpert cartridge and consumables. The cost of the GeneXpert equipment was estimated based on the procurement cost of the Xpert MTB/RIF 4-module machine and its annual maintenance cost available from the Global Drug Facility[10]. Costs associated with unused GeneXpert

For peer review only - http://bmjopen.bmj.com/site/about/guidelines.xhtml

equipment capacity were estimated by accounting for the number of tests performed per day in relation to an assumed daily maximum capacity of 16 tests[11]. Overhead costs were estimated as 5% of the total direct costs based on recent studies showing overhead costs contributing 1-10% of total Xpert costs[11-13]. The estimated unit costs for the GeneXpert test are $ 26.04 (95% UI; 18.95-33.13) for ETH and $23.70 (95% UI; 16.59-30.81) for IDN.

Clinical (re-) assessment

This cost comprised of the cost of clinical re-assessment of children with significant clinical manifestations of TB following exclusion of TB during the initial assessment.

TB reassessment at health centre

We assumed the cost for TB re-assessment at the primary health centre to be equivalent to the country-specific cost of a single outpatient visit to a health centre with no beds[4]. This equated to $5.11 (95% UI; 2.86-7.35) in ETH and $21.68 (95% UI; 11.16-32.19) in IDN.

TB reassessment at hospital

Similarly, we assumed the cost for TB re-assessment at the hospital to be equivalent to the country-specific cost of a single outpatient visit to a primary hospital, defined as a 'hospitals intended primarily for treating simple cases (e.g. "district hospital")' [4]. This resulted in an estimate of $7.19 (95% UI; 4.51-9.87) in ETH and $30.50 (95% UI; 17.84-43.16) in IDN.

TB treatment

Treatment cost for bacteriologically confirmed TB comprises of the cost of anti-tuberculosis drugs including pyridoxine, the costs of follow-up visits (drug pickups or medical review) at the healthcare facilities and costs of laboratory monitoring.

TB treatment medications

The costs of anti-tuberculosis drugs and pyridoxine were estimated using weight band based dosing and applying unit costs available from the Global Drug Facility[14]. We assumed a treatment duration of 6 months. This resulted in the following costs: $11.38, $22.77, $34.15, and $45.54 for children in the weight bands 4-7kg, 8-11kg, 12-15kg and 16-24kg, respectively. The cost of pyridoxine for the duration of TB treatment was estimated to be $2.52[14].

TB treatment follow-up at health centre

We assume the cost for each TB treatment follow-up visit at the primary health centre to be equivalent to the country-specific cost of a single outpatient visit to a health centre with no beds (see above)[4]. This unit cost was multiplied by the number of follow-up visits dictated by the national TB treatment algorithm to estimate the total cost for TB treatment follow-up. Based on input from the local TB experts, we assumed 6 follow-up visits per child on TB treatment in IND and 72 follow-up visits per child on TB treatment in ETH where clinic-based directly observed therapy is used in children. This resulted in the TB treatment follow-up cost at the health centre of $367.76 (95% UI; 167.54-814.45) in ETH and $130.05 (95% UI; 55.51-307.92) in IDN.

TB treatment follow-up at hospital

Similarly, we assumed the cost for each TB treatment follow-up visit at the hospital to be equivalent to the country-specific cost of a single outpatient visit to a primary hospital,

For peer review only - http://bmjopen.bmj.com/site/about/guidelines.xhtml

defined as a ‘hospitals intended primarily for treating simple cases (e.g. “district hospital”)’ [4]. This unit cost was multiplied by the number of follow-up visits to estimate the total cost for TB treatment follow-up. Based on input from the two local TB experts, we assumed 6 follow-up visits per child on TB treatment in IND and 72 follow-up visits per child on TB treatment in ETH where directly observed therapy is used in children. This resulted in the TB treatment follow-up cost of $517.48 (95% UI; 261.52-1033.08) in ETH and $183.00 (95% UI; 86.65-390.58) in IDN.

Laboratory monitoring
Laboratory monitoring is usually not done in children, unless they can spontaneously expectorate a sample. This is only the case for a certain proportion of the oldest age group, which is likely lower than the estimate that we use for the oldest age class (see Table A8 in Appendix 2a). Therefore, we currently do not include the cost of laboratory monitoring in our analysis.

Comparison with other cost estimates
We evaluated the accuracy of our unit cost estimates by comparing them to the recently published cost estimates for Ethiopia by the Better estimates of the costs of TB control (Value TB) project[15]. We compared the unit costs for a diagnostic visit, sputum sample collection, sputum smear microscopy examination, XpertMTB/RIF test and treatment monitoring visit. Although not exactly the same, our unit costs were quite comparable to the Value-TB cost estimates.

For peer review only

For peer review only - http://bmjopen.bmj.com/site/about/guidelines.xhtml

Costs tables

Table A1 Costs for Ethiopia & Indonesia

Cost parameter	Description	Ethiopia	Indonesia	References
c_a_phc<br>c_clin.phc	TB clinical assessment at health centre	10.22 (4.93 - 15.50)	43.35 (19.11 - 67.59)	[4]
c_e_phc<br>c_g.phc	TB clinical reassessment at health centre	5.11 (2.86 - 7.35)	21.68 (11.16 - 32.19)	[4]
c_b.phc.sess<br>c_b.h.sess	Self-expectorated sputum sample	2.32 (1.74 - 2.90)	1.74 (1.30 - 2.17)	[8]
c_b.phc.ss<br>c_b.h.ss	Stool sample	1.67 (1.25 - 2.09)	1.67 (1.25 - 2.09)	[8]
c_b.phc.ssm<br>c_b.h.ssm	Sputum smear microscopy	3.39 (1.94 - 4.83)	7.54 (5.96 - 9.12)	[5, 6]
c_b.phc.xpert<br>c_b.h.xpert	GeneXpert test	26.04 (18.95 - 33.13)	23.70 (16.59 - 30.81)	Estimated based on data from OneHealth Tool[9]
c_c.phc<br>c_d.phc<br>c_f.phc<br>c_h.phc	TB treatment at health centre	396.22 (220.27 - 572.18)	158.51 (81.18 - 235.85)	[4, 14]
c_a.h<br>c_clin.h	TB clinical assessment at hospital	14.37 (7.79 - 20.96)	61.00 (30.76 - 91.23)	[4]
c_e.h<br>c_g.h	TB clinical reassessment at hospital	7.19 (4.51 - 9.87)	30.50 (17.84 - 43.16)	[4]
c_c.h<br>c_f.h<br>c_d.h<br>c_h.h	TB treatment at hospital	396.22 (220.27 - 572.18)	158.51 (81.18 - 235.85)	[4, 14]

For peer review only - http://bmjopen.bmj.com/site/about/guidelines.xhtml

Table A2 Comparison of estimated unit costs for diagnostic visit, sputum collection, smear microscopy examination and XpertMTB/RIF test with recently published Better estimates of the costs of TB control (Value TB) project[13].

	Country	Diagnostic visit	Sputum collection	Smear microscopy examination	XpertMTB/RIF test
Health centre	ETH	5.84 (1.14-18.14)	4.64	1.69 (2.40-4.96)	26.47 (16.67-49.03)
	IND	9.91 (1.89-30.54)	4.22	3.77	27.07 (17.04-49.40)
	Value-TB ETH	3.33 (1.00-9.18)	3.28 (1-7.77)	4.53 (1.00-10.30)	20.83 (16-26.69)
Primary hospital	ETH	5.84 (1.61-26.39)	4.64	1.69 (2.40-4.96)	26.47 (16.67-49.03)
	IND	9.91 (2.75-18.76)	4.22	3.77	27.07 (17.04-49.40)
	Value-TB ETH	6.41 (1-14.66)	5.12 (3.00-6.91)	6.24 (4.00-8.43)	37.87 (19-57.78)

For peer review only

For peer review only - http://bmjopen.bmj.com/site/about/guidelines.xhtml

References

1. Ministry of Health Ethiopia., *Guidelines for Clinical and Programmatic Management of TB, DR-TB and Leprosy in Ethiopia, 7th edition*. 2020, Ministry of Health Ethiopia: Addis Ababa, Ethiopia.
2. Ministry of Health Republic of Indonesia., *National guideline for TB control*. 2016, Ministry of Health Republic of Indonesia: Jakarta, Indonesia.
3. World Bank. *World Bank national accounts data, and OECD National Accounts data files. GDP deflator*. 2021 14/05/2021]; Available from: https://data.worldbank.org/indicator/NY.GDP.DEFL.ZS.
4. Stenberg, K., et al., *Econometric estimation of WHO-CHOICE country-specific costs for inpatient and outpatient health service delivery.* Cost Eff Resour Alloc, 2018. **16**: p. 11.
5. Tesfaye, A., et al., *Modeling the patient and health system impacts of alternative xpert® MTB/RIF algorithms for the diagnosis of pulmonary tuberculosis in Addis Ababa, Ethiopia.* BMC infectious diseases, 2017. **17**(1): p. 318-318.
6. Jarrah Z, C.D., Hafidz F. , *The cost of scaling up TB services in Indonesia. TB CARE I–management sciences for health. Cambridge: Management Science for Health.* . 2013.
7. Peter, J.G., et al., *Comparison of two methods for acquisition of sputum samples for diagnosis of suspected tuberculosis in smear-negative or sputum-scarce people: a randomised controlled trial.* Lancet Respir Med, 2013. **1**(6): p. 471-8.
8. Paediatric Operational Sustainability Expertise Exchange group. *Paediatric Operational Sustainability Expertise Exchange group (POSEE group) budgeting tools* 2020 [cited 16/02/2021; Available from: https://www.dropbox.com/sh/rmzlqoot9m1muxe/AACOPh8SuS4WwMQD_vfN-oHCa?dl=0.
9. World Health Organization. *OneHealth Tool.* 2020; Available from: http://www.who.int/choice/onehealthtool/en/.
10. Stop TB Partnership | Global Drug Facility, *Global Drug Facility (GDF) Diagnostics catalogue August 2020.* 2021.
11. Sarin, S., et al., *Cost and operational impact of promoting upfront GeneXpert MTB/RIF test referrals for presumptive pediatric tuberculosis patients in India.* PLOS ONE, 2019. **14**(4): p. e0214675.
12. Sohn, H., et al., *Cost and affordability analysis of TB-LAMP and Xpert MTB/RIF assays as routine diagnostic tests in peripheral laboratories in Malawi and Vietnam.* J Glob Health Sci, 2019. **1**(1).
13. Pooran, A., et al., *Point of care Xpert MTB/RIF versus smear microscopy for tuberculosis diagnosis in southern African primary care clinics: a multicentre economic evaluation.* The Lancet. Global health, 2019. **7**(6): p. e798-e807.
14. Stop TB Partnership | Global Drug Facility, *Global Drug Facility (GDF) Medicines catalogue January 2021*. 2021.
15. Sweeney, S., et al., *Value TB Dataset: costs per intervention*. 2021, Harvard Dataverse.

For peer review only - http://bmjopen.bmj.com/site/about/guidelines.xhtml

Xpert Ultra stool testing to diagnose tuberculosis in children in Ethiopia and Indonesia: a model-based cost-effectiveness analysis.

Appendix 3: Supplementary results

Additional results for base case analysis 2
- Age-specific results 2
  - Age 0-4 years 2
  - Age 5-14 years 4
- Cost-effectiveness acceptability curves 6

Results for low prevalence sensitivity analysis 8
- Age-specific results 8
  - All ages: 0-14 years 8
  - Age 0-4 years 10
  - Age 5-14 years 12

Results for Xpert baseline sensitivity analysis 14
- Age-specific results 14
  - All ages: 0-14 years 14
  - Age 0-4 years 16
  - Age 5-14 years 18

Results for 0% discount rate sensitivity analysis 20
- Age-specific results 20
  - All ages: 0-14 years 20
  - Age 0-4 years 22
  - Age 5-14 years 24

Results for 5% discount rate sensitivity analysis 26
- Age-specific results 26
  - All ages: 0-14 years 26
  - Age 0-4 years 28
  - Age 5-14 years 30

Comparison of ICERs for sensitivity analyses 32

For peer review only

For peer review only - http://bmjopen.bmj.com/site/about/guidelines.xhtml

Additional results for base case analysis

Age-specific results

Age 0-4 years

Table A1 Ethiopia

Quantity per 100 children with presumptive TB (unless stated):	Standard of care	Intervention	Difference
children with true TB	45.5 (8.7 - 85.0)	45.5 (8.7 - 85.0)	0.0 (0.0 - 0.0)
assessments	208.1 (172.8 - 241.7)	252.9 (217.5 - 286.1)	44.7 (32.9 - 55.1)
bacteriological investigations	3.4 (2.1 - 4.9)	103.1 (87.6 - 112.5)	99.8 (84.3 - 109.1)
anti-TB treatments (ATT)	36.6 (15.3 - 59.7)	36.4 (15.2 - 59.3)	-0.2 (-2.8 - 2.7)
ATT initiated at PHC	68.3 (59.6 - 75.7)	81.0 (70.9 - 88.4)	12.7 (8.0 - 17.4)
percent of true-positive receiving ATT	66.2 (55.9 - 75.4)	66.8 (58.4 - 74.6)	0.7 (-3.4 - 5.1)
percent of ATT bacteriologically confirmed	0.6 (0.3 - 1.0)	31.4 (20.4 - 42.2)	30.8 (19.9 - 41.4)
percent of ATT false-positive	21.7 (2.4 - 65.8)	20.7 (2.4 - 63.4)	-1.0 (-4.2 - 1.9)
referrals, inc. self-referrals	40.1 (28.3 - 51.7)	14.7 (9.1 - 21.8)	-25.4 (-33.2 - -17.4)
deaths	7.5 (1.4 - 15.2)	7.3 (1.4 - 14.7)	-0.1 (-1.2 - 0.7)
life-years lost	205.9 (37.9 - 418.6)	202.4 (37.5 - 405.7)	-3.4 (-32.0 - 19.9)
cost	17934.1 (7124.0 - 35159.0)	17667.9 (7614.0 - 32685.5)	-266.2 (-13326.6 - 12081.4)

For peer review only

Table A2 Indonesia

Quantity per 100 children with presumptive TB (unless stated):	Standard of care	Intervention	Difference
children with true TB	45.5 (8.7 - 85.0)	45.5 (8.7 - 85.0)	0.0 (0.0 - 0.0)
assessments	209.4 (173.8 - 242.9)	255.2 (219.1 - 289.2)	45.9 (33.8 - 56.0)
bacteriological investigations	3.4 (2.1 - 5.0)	103.6 (88.2 - 113.0)	100.2 (84.9 - 109.5)
anti-TB treatments (ATT)	36.8 (15.4 - 60.4)	36.5 (15.2 - 59.6)	-0.3 (-3.0 - 2.4)
ATT initiated at PHC	70.1 (61.1 - 77.4)	83.6 (72.6 - 90.3)	13.4 (8.6 - 18.1)
percent of true-positive receiving ATT	66.6 (56.3 - 76.0)	67.0 (58.5 - 74.8)	0.4 (-3.7 - 4.8)
percent of ATT bacteriologically confirmed	0.6 (0.3 - 1.0)	31.4 (20.4 - 42.1)	30.8 (20.0 - 41.3)
percent of ATT false-positive	21.8 (2.4 - 65.8)	20.7 (2.4 - 63.7)	-1.1 (-4.3 - 1.9)
referrals, inc. self-referrals	41.5 (29.2 - 53.1)	15.3 (9.5 - 22.4)	-26.3 (-34.4 - -17.9)
deaths	7.4 (1.4 - 15.1)	7.3 (1.4 - 14.6)	-0.1 (-1.1 - 0.8)
life-years lost	209.8 (38.7 - 429.1)	207.8 (39.0 - 416.6)	-2.0 (-31.0 - 23.2)
cost	13672.3 (7286.9 - 22370.9)	14090.7 (8344.4 - 21727.0)	418.4 (-8064.8 - 9011.5)

For peer review only - http://bmjopen.bmj.com/site/about/guidelines.xhtml

Age 5-14 years

Table A3 Ethiopia

Quantity per 100 children with presumptive TB (unless stated):	Standard of care	Intervention	Difference
children with true TB	45.5 (8.7 - 85.0)	45.5 (8.7 - 85.0)	0.0 (0.0 - 0.0)
assessments	197.9 (169.6 - 225.2)	242.2 (200.8 - 281.7)	44.3 (24.8 - 62.7)
bacteriological investigations	47.1 (12.5 - 82.7)	101.9 (86.2 - 111.6)	54.8 (16.5 - 91.1)
anti-TB treatments (ATT)	29.6 (10.6 - 53.8)	42.6 (18.5 - 67.8)	13.0 (1.5 - 30.3)
ATT initiated at PHC	74.6 (63.8 - 84.3)	82.4 (71.8 - 90.2)	7.8 (2.1 - 12.9)
percent of true-positive receiving ATT	53.6 (31.9 - 72.9)	76.8 (69.4 - 83.1)	23.2 (5.1 - 45.4)
percent of ATT bacteriologically confirmed	14.5 (2.4 - 39.5)	33.7 (20.3 - 46.1)	19.2 (-5.9 - 37.4)
percent of ATT false-positive	21.6 (2.6 - 65.3)	22.4 (2.8 - 66.2)	0.8 (-2.5 - 6.2)
referrals, inc. self-referrals	23.1 (8.8 - 39.1)	13.3 (7.3 - 20.5)	-9.7 (-22.7 - 4.4)
deaths	3.4 (0.6 - 7.4)	1.9 (0.3 - 3.9)	-1.5 (-4.0 - -0.2)
life-years lost	93.7 (16.9 - 203.0)	52.5 (9.6 - 107.9)	-41.1 (-111.6 - -4.3)
cost	14407.8 (5303.7 - 29936.5)	20277.4 (8872.7 - 37127.0)	5869.6 (-6634.5 - 19361.5)

For peer review only

Table A4 Indonesia

Quantity per 100 children with presumptive TB (unless stated):	Standard of care	Intervention	Difference
children with true TB	45.5 (8.7 - 85.0)	45.5 (8.7 - 85.0)	0.0 (0.0 - 0.0)
assessments	198.8 (170.2 - 226.2)	244.3 (202.0 - 284.1)	45.5 (25.6 - 64.3)
bacteriological investigations	47.3 (12.7 - 83.3)	102.3 (86.7 - 112.2)	54.9 (16.6 - 91.6)
anti-TB treatments (ATT)	29.6 (10.5 - 54.2)	42.7 (18.5 - 68.1)	13.1 (1.4 - 30.8)
ATT initiated at PHC	77.2 (65.5 - 87.0)	85.1 (73.6 - 92.2)	7.9 (1.5 - 13.5)
percent of true-positive receiving ATT	53.6 (31.5 - 73.0)	76.9 (69.6 - 83.3)	23.3 (4.9 - 45.9)
percent of ATT bacteriologically confirmed	14.1 (2.3 - 38.3)	33.7 (20.2 - 46.1)	19.5 (-4.9 - 37.4)
percent of ATT false-positive	21.7 (2.6 - 65.3)	22.4 (2.8 - 66.3)	0.8 (-2.6 - 6.1)
referrals, inc. self-referrals	23.9 (9.1 - 40.3)	13.8 (7.5 - 21.2)	-10.1 (-23.7 - 4.3)
deaths	3.4 (0.6 - 7.4)	1.9 (0.4 - 3.9)	-1.5 (-4.1 - -0.1)
life-years lost	96.6 (17.4 - 210.3)	53.9 (10.0 - 111.1)	-42.7 (-115.7 - -4.3)
cost	11270.2 (6013.6 - 18958.7)	14987.1 (8815.0 - 23229.9)	3716.9 (-3812.4 - 10646.6)

For peer review only

Cost-effectiveness acceptability curves

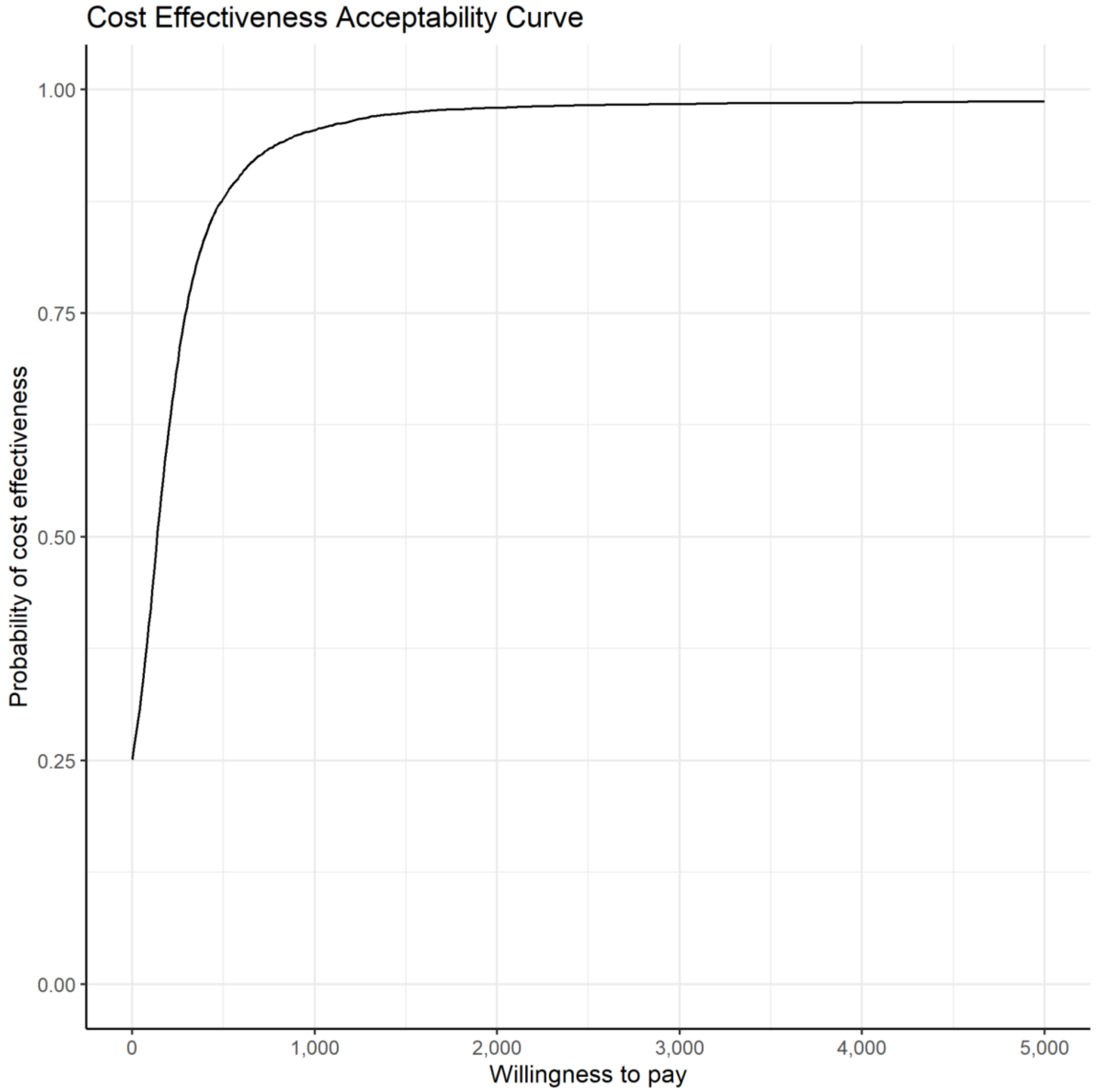

Figure A1 Ethiopia

For peer review only - http://bmjopen.bmj.com/site/about/guidelines.xhtml

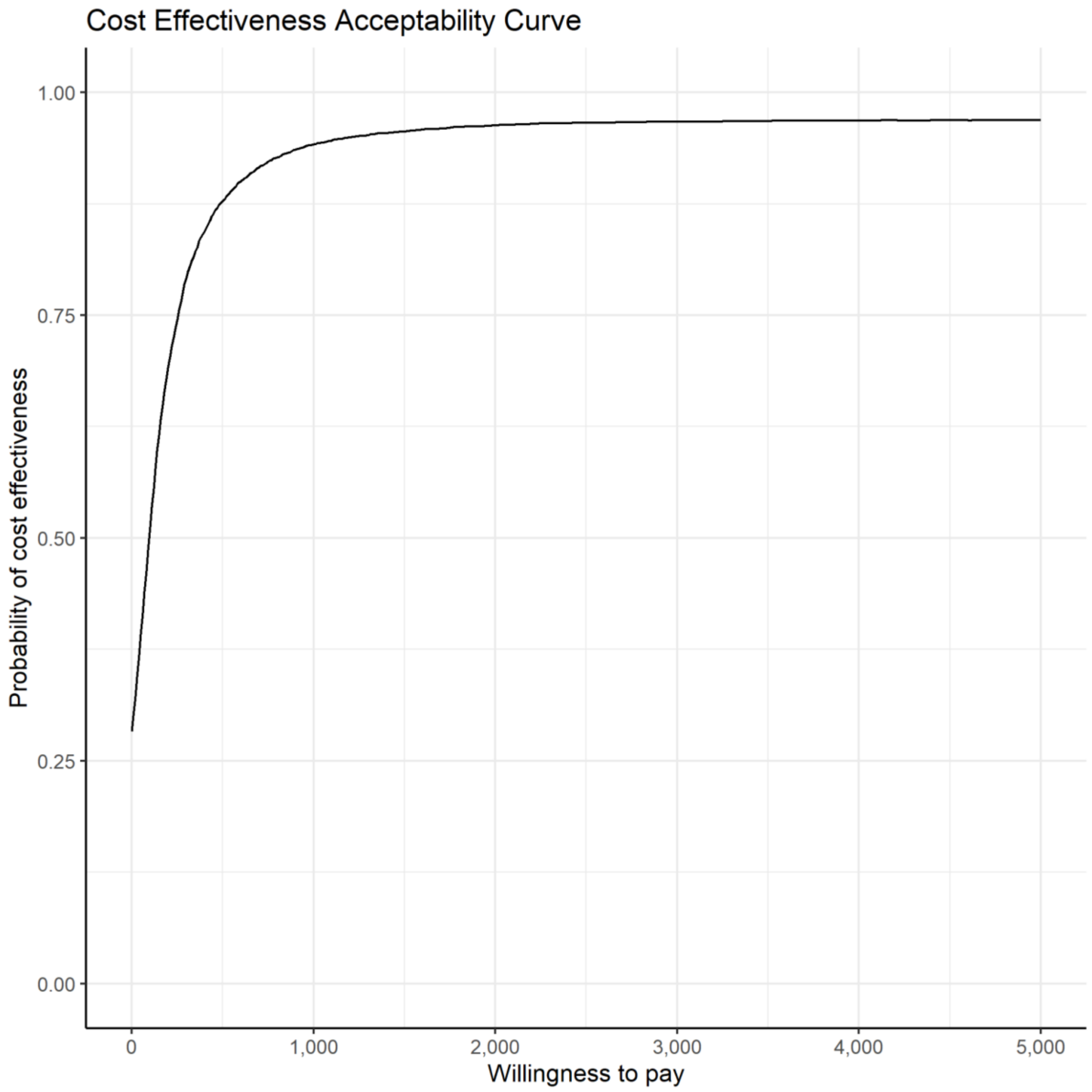

Figure A2 Indonesia

For peer review only - http://bmjopen.bmj.com/site/about/guidelines.xhtml

Results for low prevalence sensitivity analysis

Age-specific results

All ages: 0-14 years

Table A5 Ethiopia

Quantity per 100 children with presumptive TB (unless stated):	Standard of care	Intervention	Difference
children with true TB	22.8 (4.4 - 42.5)	22.8 (4.4 - 42.5)	0.0 (0.0 - 0.0)
assessments	224.4 (203.7 - 248.1)	260.5 (239.5 - 281.9)	36.0 (22.2 - 53.2)
bacteriological investigations	32.9 (9.7 - 60.1)	98.3 (85.7 - 110.0)	65.3 (36.2 - 91.3)
anti-TB treatments (ATT)	22.3 (11.0 - 35.6)	25.5 (13.5 - 38.0)	3.2 (-3.1 - 11.4)
ATT initiated at PHC	66.7 (56.8 - 76.9)	84.0 (72.4 - 93.3)	17.3 (8.7 - 26.2)
percent of true-positive receiving ATT	60.4 (44.3 - 74.6)	71.2 (64.4 - 77.6)	10.8 (-2.7 - 27.7)
percent of ATT bacteriologically confirmed	6.9 (1.5 - 17.2)	29.0 (17.8 - 39.9)	22.1 (9.7 - 34.0)
percent of ATT false-positive	41.2 (17.1 - 79.8)	39.7 (16.7 - 78.8)	-1.5 (-5.9 - 4.2)
referrals, inc. self-referrals	41.7 (22.6 - 60.9)	9.4 (2.0 - 21.6)	-32.2 (-52.2 - -12.2)
deaths	2.3 (0.4 - 4.8)	2.1 (0.4 - 4.4)	-0.2 (-1.1 - 0.3)
life-years lost	64.0 (11.7 - 131.4)	57.3 (10.4 - 120.7)	-6.7 (-30.5 - 9.3)
cost	11688.2 (5594.7 - 21367.9)	12666.3 (6357.3 - 21679.1)	978.1 (-7407.9 - 9219.3)

peer review only

Table A6 Indonesia

Quantity per 100 children with presumptive TB (unless stated):	Standard of care	Intervention	Difference
children with true TB	22.8 (4.4 - 42.5)	22.8 (4.4 - 42.5)	0.0 (0.0 - 0.0)
assessments	221.3 (204.3 - 239.6)	271.1 (248.7 - 292.5)	49.8 (37.8 - 60.8)
bacteriological investigations	25.5 (8.1 - 43.9)	105.5 (90.4 - 113.9)	80.0 (56.7 - 100.3)
anti-TB treatments (ATT)	22.1 (11.2 - 34.2)	26.0 (14.1 - 38.5)	4.0 (-0.4 - 9.9)
ATT initiated at PHC	70.8 (61.5 - 78.1)	82.7 (71.8 - 89.5)	11.9 (7.2 - 16.4)
percent of true-positive receiving ATT	60.3 (48.2 - 71.4)	71.8 (65.9 - 77.3)	11.5 (1.8 - 23.1)
percent of ATT bacteriologically confirmed	5.0 (1.2 - 11.2)	28.7 (17.8 - 39.1)	23.6 (13.1 - 34.3)
percent of ATT false-positive	40.8 (16.7 - 79.7)	40.5 (17.4 - 79.3)	-0.3 (-4.1 - 5.0)
referrals, inc. self-referrals	38.2 (27.9 - 48.3)	17.4 (12.9 - 23.8)	-20.8 (-29.8 - -10.8)
deaths	2.7 (0.5 - 5.4)	2.3 (0.4 - 4.6)	-0.4 (-1.1 - 0.0)
life-years lost	77.4 (14.7 - 155.1)	66.5 (12.4 - 132.3)	-10.9 (-30.9 - 0.1)
cost	10894.9 (6314.8 - 17164.5)	12513.6 (7709.2 - 18868.1)	1618.7 (-4945.3 - 8121.1)

For peer review only - http://bmjopen.bmj.com/site/about/guidelines.xhtml

Age 0-4 years

Table A7 Ethiopia

Quantity per 100 children with presumptive TB (unless stated):	Standard of care	Intervention	Difference
children with true TB	22.8 (4.4 - 42.5)	22.8 (4.4 - 42.5)	0.0 (0.0 - 0.0)
assessments	235.5 (209.9 - 262.1)	264.5 (244.8 - 284.9)	29.0 (13.7 - 47.9)
bacteriological investigations	3.7 (2.3 - 5.5)	98.6 (86.2 - 110.5)	94.8 (82.5 - 106.6)
anti-TB treatments (ATT)	25.5 (12.3 - 39.3)	22.6 (11.0 - 34.7)	-3.0 (-6.9 - 0.4)
ATT initiated at PHC	62.6 (53.4 - 72.7)	83.6 (71.9 - 93.1)	21.0 (11.6 - 30.7)
percent of true-positive receiving ATT	69.3 (57.6 - 79.5)	64.8 (55.8 - 73.1)	-4.5 (-10.6 - 2.6)
percent of ATT bacteriologically confirmed	0.6 (0.3 - 1.0)	28.8 (17.8 - 39.7)	28.2 (17.4 - 38.9)
percent of ATT false-positive	40.7 (14.3 - 80.7)	37.8 (14.0 - 77.9)	-2.9 (-6.6 - 1.8)
referrals, inc. self-referrals	56.0 (35.1 - 73.4)	9.8 (2.1 - 22.3)	-46.2 (-64.8 - -25.7)
deaths	3.4 (0.6 - 7.2)	3.9 (0.7 - 7.8)	0.4 (-0.2 - 1.4)
life-years lost	94.9 (17.3 - 197.9)	106.6 (19.9 - 213.9)	11.7 (-6.3 - 37.5)
cost	13498.5 (6292.1 - 24528.2)	11443.3 (5638.1 - 19702.2)	-2055.2 (-11147.7 - 5839.4)

For peer review only

Table A8 Indonesia

Quantity per 100 children with presumptive TB (unless stated):	Standard of care	Intervention	Difference
children with true TB	22.8 (4.4 - 42.5)	22.8 (4.4 - 42.5)	0.0 (0.0 - 0.0)
assessments	228.3 (208.1 - 249.0)	274.5 (252.9 - 294.7)	46.2 (34.0 - 55.8)
bacteriological investigations	3.5 (2.2 - 5.2)	106.0 (90.9 - 114.3)	102.5 (87.3 - 110.8)
anti-TB treatments (ATT)	24.4 (11.7 - 37.5)	23.8 (11.8 - 36.2)	-0.6 (-3.0 - 1.4)
ATT initiated at PHC	67.8 (59.3 - 75.2)	82.1 (71.5 - 88.8)	14.2 (9.1 - 18.9)
percent of true-positive receiving ATT	66.6 (56.3 - 76.0)	67.0 (58.5 - 74.8)	0.4 (-3.7 - 4.8)
percent of ATT bacteriologically confirmed	0.5 (0.3 - 1.0)	28.7 (17.6 - 39.6)	28.1 (17.2 - 38.9)
percent of ATT false-positive	40.3 (14.1 - 80.3)	38.8 (14.6 - 78.5)	-1.5 (-4.8 - 3.1)
referrals, inc. self-referrals	47.5 (38.8 - 55.3)	17.9 (13.4 - 24.4)	-29.6 (-35.9 - -22.1)
deaths	3.7 (0.7 - 7.5)	3.7 (0.7 - 7.3)	-0.0 (-0.5 - 0.4)
life-years lost	104.9 (19.4 - 214.5)	103.9 (19.5 - 208.3)	-1.0 (-15.5 - 11.6)
cost	11896.1 (6406.6 - 19196.8)	12176.0 (7451.0 - 18467.1)	279.8 (-7399.6 - 7997.8)

For peer review only

Age 5-14 years

Table A9 Ethiopia

Quantity per 100 children with presumptive TB (unless stated):	Standard of care	Intervention	Difference
children with true TB	22.8 (4.4 - 42.5)	22.8 (4.4 - 42.5)	0.0 (0.0 - 0.0)
assessments	217.8 (198.9 - 241.4)	258.0 (236.2 - 280.4)	40.3 (25.0 - 58.7)
bacteriological investigations	50.4 (13.8 - 85.9)	98.1 (85.4 - 109.7)	47.6 (10.1 - 85.7)
anti-TB treatments (ATT)	20.3 (8.1 - 35.7)	27.2 (14.0 - 40.7)	6.9 (-2.2 - 18.6)
ATT initiated at PHC	70.2 (58.8 - 81.9)	84.2 (72.7 - 93.4)	14.1 (4.7 - 23.9)
percent of true-positive receiving ATT	55.1 (32.5 - 75.7)	75.1 (67.2 - 82.0)	20.0 (0.5 - 43.3)
percent of ATT bacteriologically confirmed	12.7 (2.1 - 35.4)	29.4 (16.9 - 41.7)	16.6 (-5.9 - 33.0)
percent of ATT false-positive	40.6 (15.3 - 80.4)	40.2 (16.0 - 79.7)	-0.4 (-5.3 - 6.7)
referrals, inc. self-referrals	33.1 (12.2 - 55.5)	9.2 (1.9 - 21.2)	-23.8 (-47.1 - -1.3)
deaths	1.6 (0.3 - 3.6)	1.0 (0.2 - 2.1)	-0.6 (-1.9 - -0.0)
life-years lost	45.5 (8.0 - 100.0)	27.7 (5.1 - 57.5)	-17.8 (-51.9 - -0.3)
cost	10603.2 (4477.3 - 20733.1)	13401.2 (6616.6 - 22999.5)	2798.0 (-6051.3 - 11513.5)

For peer review only - http://bmjopen.bmj.com/site/about/guidelines.xhtml

Table A10 Indonesia

Quantity per 100 children with presumptive TB (unless stated):	Standard of care	Intervention	Difference
children with true TB	22.8 (4.4 - 42.5)	22.8 (4.4 - 42.5)	0.0 (0.0 - 0.0)
assessments	213.8 (198.0 - 233.1)	267.4 (243.3 - 290.7)	53.6 (39.5 - 69.5)
bacteriological investigations	48.9 (13.3 - 84.6)	105.1 (89.9 - 113.6)	56.2 (18.0 - 93.5)
anti-TB treatments (ATT)	19.6 (7.9 - 34.4)	28.5 (15.0 - 42.2)	8.8 (0.5 - 20.4)
ATT initiated at PHC	75.1 (63.7 - 85.5)	83.2 (72.2 - 90.3)	8.1 (0.7 - 14.4)
percent of true-positive receiving ATT	53.6 (31.5 - 73.0)	76.9 (69.6 - 83.3)	23.3 (4.9 - 45.9)
percent of ATT bacteriologically confirmed	12.2 (1.9 - 34.3)	29.1 (16.6 - 41.3)	16.9 (-4.8 - 33.0)
percent of ATT false-positive	40.2 (15.1 - 79.9)	41.4 (16.8 - 80.4)	1.2 (-3.2 - 8.0)
referrals, inc. self-referrals	28.3 (11.5 - 44.1)	16.9 (12.3 - 23.3)	-11.4 (-26.4 - 5.6)
deaths	1.7 (0.3 - 3.7)	0.9 (0.2 - 2.0)	-0.8 (-2.0 - -0.1)
life-years lost	48.3 (8.7 - 105.1)	26.9 (5.0 - 55.6)	-21.4 (-57.9 - -2.1)
cost	9830.4 (5352.0 - 15979.3)	12871.7 (7911.6 - 19463.3)	3041.3 (-3390.0 - 8592.6)

For peer review only - http://bmjopen.bmj.com/site/about/guidelines.xhtml

Results for Xpert baseline sensitivity analysis

Age-specific results

All ages: 0-14 years

Table A11 Ethiopia

Quantity per 100 children with presumptive TB (unless stated):	Standard of care	Intervention	Difference
children with true TB	45.5 (8.7 - 85.0)	45.5 (8.7 - 85.0)	0.0 (0.0 - 0.0)
assessments	199.4 (168.3 - 230.2)	246.2 (207.3 - 283.3)	46.8 (33.2 - 59.9)
bacteriological investigations	30.7 (8.7 - 57.5)	102.3 (86.8 - 112.0)	71.7 (41.5 - 96.3)
anti-TB treatments (ATT)	34.3 (14.2 - 56.8)	40.3 (17.6 - 64.4)	6.0 (0.1 - 15.0)
ATT initiated at PHC	73.6 (63.9 - 81.8)	81.9 (71.6 - 89.5)	8.4 (3.2 - 13.2)
percent of true-positive receiving ATT	62.6 (51.4 - 72.7)	73.0 (66.7 - 78.8)	10.5 (1.6 - 22.0)
percent of ATT bacteriologically confirmed	14.0 (3.0 - 32.6)	32.8 (20.7 - 44.1)	18.8 (-0.2 - 34.6)
percent of ATT false-positive	21.3 (2.7 - 63.8)	21.9 (2.9 - 64.9)	0.6 (-2.4 - 5.1)
referrals, inc. self-referrals	29.5 (17.0 - 42.9)	13.8 (8.0 - 21.0)	-15.6 (-25.8 - -4.9)
deaths	4.6 (0.9 - 9.4)	3.9 (0.7 - 8.3)	-0.7 (-2.0 - 0.0)
life-years lost	127.7 (23.8 - 260.7)	108.7 (19.7 - 228.5)	-19.0 (-55.1 - 0.1)
cost	16678.2 (6843.1 - 32205.5)	19297.7 (8413.8 - 35444.7)	2619.5 (-9141.3 - 14513.1)

For peer review only - http://bmjopen.bmj.com/site/about/guidelines.xhtml

Table A12 Indonesia

Quantity per 100 children with presumptive TB (unless stated):	Standard of care	Intervention	Difference
children with true TB	45.5 (8.7 - 85.0)	45.5 (8.7 - 85.0)	0.0 (0.0 - 0.0)
assessments	202.3 (170.6 - 232.9)	249.9 (211.2 - 286.5)	47.7 (34.7 - 59.6)
bacteriological investigations	24.7 (7.8 - 43.2)	103.0 (87.5 - 112.6)	78.2 (54.5 - 98.4)
anti-TB treatments (ATT)	35.1 (14.8 - 57.5)	39.5 (17.1 - 63.3)	4.4 (-0.4 - 11.1)
ATT initiated at PHC	74.4 (64.7 - 81.7)	84.4 (73.2 - 91.2)	10.0 (5.4 - 14.6)
percent of true-positive receiving ATT	63.8 (54.3 - 72.8)	71.8 (65.9 - 77.3)	8.0 (0.6 - 16.6)
percent of ATT bacteriologically confirmed	10.7 (2.6 - 22.7)	32.5 (20.9 - 43.4)	21.7 (7.7 - 35.3)
percent of ATT false-positive	21.6 (2.7 - 64.4)	21.8 (2.9 - 64.6)	0.2 (-2.8 - 4.2)
referrals, inc. self-referrals	33.0 (21.5 - 45.5)	14.5 (8.6 - 21.7)	-18.4 (-27.6 - -9.5)
deaths	5.2 (1.0 - 10.3)	4.7 (0.9 - 9.3)	-0.5 (-1.6 - 0.1)
life-years lost	147.8 (28.1 - 294.2)	133.1 (24.7 - 264.6)	-14.7 (-45.7 - 3.5)
cost	12852.5 (7260.6 - 20698.7)	14525.7 (8603.6 - 22403.0)	1673.3 (-5630.9 - 8936.4)

For peer review only

Age 0-4 years

Table A13 Ethiopia

Quantity per 100 children with presumptive TB (unless stated):	Standard of care	Intervention	Difference
children with true TB	45.5 (8.7 - 85.0)	45.5 (8.7 - 85.0)	0.0 (0.0 - 0.0)
assessments	208.0 (172.6 - 241.6)	252.9 (217.5 - 286.1)	44.9 (33.1 - 55.2)
bacteriological investigations	3.4 (2.1 - 4.9)	103.1 (87.6 - 112.5)	99.8 (84.3 - 109.1)
anti-TB treatments (ATT)	36.7 (15.4 - 59.9)	36.4 (15.2 - 59.3)	-0.3 (-2.9 - 2.5)
ATT initiated at PHC	68.4 (59.7 - 75.8)	81.0 (70.9 - 88.4)	12.6 (7.9 - 17.2)
percent of true-positive receiving ATT	66.5 (56.2 - 75.7)	66.8 (58.4 - 74.6)	0.3 (-3.7 - 4.8)
percent of ATT bacteriologically confirmed	1.1 (0.5 - 1.7)	31.4 (20.4 - 42.2)	30.3 (19.7 - 41.0)
percent of ATT false-positive	21.7 (2.4 - 65.7)	20.7 (2.4 - 63.4)	-1.0 (-4.1 - 1.9)
referrals, inc. self-referrals	40.1 (28.3 - 51.7)	14.7 (9.1 - 21.8)	-25.4 (-33.2 - -17.4)
deaths	7.4 (1.4 - 15.1)	7.3 (1.4 - 14.7)	-0.1 (-1.1 - 0.8)
life-years lost	204.3 (37.6 - 416.1)	202.4 (37.5 - 405.7)	-1.8 (-29.6 - 21.8)
cost	18004.2 (7149.6 - 35253.8)	17667.9 (7614.0 - 32685.5)	-336.2 (-13382.6 - 11963.8)

For peer review only

For peer review only - http://bmjopen.bmj.com/site/about/guidelines.xhtml

Table A14 Indonesia

**Quantity per 100 children with presumptive TB (unless stated):**	**Standard of care**	**Intervention**	**Difference**
children with true TB	45.5 (8.7 - 85.0)	45.5 (8.7 - 85.0)	0.0 (0.0 - 0.0)
assessments	209.2 (173.5 - 242.9)	255.2 (219.1 - 289.2)	46.1 (34.0 - 56.2)
bacteriological investigations	3.4 (2.1 - 5.0)	103.6 (88.2 - 113.0)	100.2 (84.9 - 109.5)
anti-TB treatments (ATT)	37.0 (15.5 - 60.7)	36.5 (15.2 - 59.6)	-0.5 (-3.1 - 2.2)
ATT initiated at PHC	70.3 (61.3 - 77.5)	83.6 (72.6 - 90.3)	13.3 (8.4 - 17.9)
percent of true-positive receiving ATT	67.0 (56.6 - 76.3)	67.0 (58.5 - 74.8)	0.1 (-3.9 - 4.5)
percent of ATT bacteriologically confirmed	1.1 (0.5 - 1.7)	31.4 (20.4 - 42.1)	30.3 (19.6 - 40.9)
percent of ATT false-positive	21.8 (2.4 - 65.7)	20.7 (2.4 - 63.7)	-1.0 (-4.2 - 1.9)
referrals, inc. self-referrals	41.5 (29.2 - 53.1)	15.3 (9.5 - 22.4)	-26.3 (-34.4 - -17.9)
deaths	7.3 (1.4 - 15.0)	7.3 (1.4 - 14.6)	-0.0 (-1.0 - 0.9)
life-years lost	208.1 (38.4 - 426.3)	207.8 (39.0 - 416.6)	-0.3 (-28.4 - 25.5)
cost	13704.3 (7307.0 - 22423.2)	14090.7 (8344.4 - 21727.0)	386.4 (-8103.9 - 8967.9)

For peer review only - http://bmjopen.bmj.com/site/about/guidelines.xhtml

Age 5-14 years

Table A15 Ethiopia

Quantity per 100 children with presumptive TB (unless stated):	Standard of care	Intervention	Difference
children with true TB	45.5 (8.7 - 85.0)	45.5 (8.7 - 85.0)	0.0 (0.0 - 0.0)
assessments	194.2 (164.3 - 224.2)	242.2 (200.8 - 281.7)	48.0 (31.6 - 65.4)
bacteriological investigations	47.1 (12.5 - 82.7)	101.9 (86.2 - 111.6)	54.8 (16.5 - 91.1)
anti-TB treatments (ATT)	32.9 (12.5 - 56.8)	42.6 (18.5 - 67.8)	9.7 (0.9 - 22.5)
ATT initiated at PHC	77.2 (66.1 - 87.6)	82.4 (71.8 - 90.2)	5.2 (-2.2 - 11.8)
percent of true-positive receiving ATT	60.2 (44.6 - 74.9)	76.8 (69.4 - 83.1)	16.5 (3.3 - 32.6)
percent of ATT bacteriologically confirmed	23.5 (4.2 - 56.3)	33.7 (20.3 - 46.1)	10.2 (-22.8 - 33.6)
percent of ATT false-positive	20.7 (2.5 - 64.1)	22.4 (2.8 - 66.2)	1.7 (-1.9 - 8.3)
referrals, inc. self-referrals	23.0 (8.8 - 39.1)	13.3 (7.3 - 20.5)	-9.7 (-22.7 - 4.4)
deaths	3.0 (0.6 - 6.3)	1.9 (0.3 - 3.9)	-1.1 (-2.9 - -0.1)
life-years lost	81.8 (15.2 - 172.9)	52.5 (9.6 - 107.9)	-29.3 (-80.4 - -2.9)
cost	15884.6 (6196.1 - 31393.0)	20277.4 (8872.7 - 37127.0)	4392.8 (-7520.5 - 16511.0)

For peer review only - http://bmjopen.bmj.com/site/about/guidelines.xhtml

Table A16 Indonesia

Quantity per 100 children with presumptive TB (unless stated):	Standard of care	Intervention	Difference
children with true TB	45.5 (8.7 - 85.0)	45.5 (8.7 - 85.0)	0.0 (0.0 - 0.0)
assessments	194.9 (164.8 - 225.1)	244.3 (202.0 - 284.1)	49.4 (32.8 - 67.1)
bacteriological investigations	47.3 (12.7 - 83.3)	102.3 (86.7 - 112.2)	54.9 (16.6 - 91.6)
anti-TB treatments (ATT)	33.1 (12.6 - 57.0)	42.7 (18.5 - 68.1)	9.7 (0.8 - 22.5)
ATT initiated at PHC	79.6 (67.8 - 90.1)	85.1 (73.6 - 92.2)	5.5 (-2.4 - 12.6)
percent of true-positive receiving ATT	60.5 (44.7 - 75.1)	76.9 (69.6 - 83.3)	16.5 (3.1 - 32.6)
percent of ATT bacteriologically confirmed	23.5 (4.3 - 56.3)	33.7 (20.2 - 46.1)	10.2 (-22.6 - 33.6)
percent of ATT false-positive	20.8 (2.5 - 64.3)	22.4 (2.8 - 66.3)	1.7 (-1.9 - 8.3)
referrals, inc. self-referrals	23.8 (9.0 - 40.3)	13.8 (7.5 - 21.2)	-10.1 (-23.7 - 4.3)
deaths	2.9 (0.6 - 6.3)	1.9 (0.4 - 3.9)	-1.1 (-2.9 - -0.1)
life-years lost	83.9 (15.8 - 178.7)	53.9 (10.0 - 111.1)	-30.1 (-83.3 - -2.7)
cost	11945.9 (6523.9 - 19684.3)	14987.1 (8815.0 - 23229.9)	3041.3 (-4161.7 - 9508.2)

For peer review only - http://bmjopen.bmj.com/site/about/guidelines.xhtml

Results for 0% discount rate sensitivity analysis

Age-specific results

All ages: 0-14 years

Table A17 Ethiopia

Quantity per 100 children with presumptive TB (unless stated):	Standard of care	Intervention	Difference
children with true TB	45.5 (8.7 - 85.0)	45.5 (8.7 - 85.0)	0.0 (0.0 - 0.0)
assessments	201.8 (171.8 - 230.9)	246.2 (207.3 - 283.3)	44.4 (29.5 - 58.1)
bacteriological investigations	30.7 (8.7 - 57.5)	102.3 (86.8 - 112.0)	71.7 (41.5 - 96.3)
anti-TB treatments (ATT)	32.2 (13.2 - 54.5)	40.3 (17.6 - 64.4)	8.1 (0.6 - 20.3)
ATT initiated at PHC	71.8 (62.3 - 79.6)	81.9 (71.6 - 89.5)	10.1 (5.8 - 14.2)
percent of true-positive receiving ATT	58.3 (43.0 - 71.1)	73.0 (66.7 - 78.8)	14.7 (2.8 - 30.5)
percent of ATT bacteriologically confirmed	8.0 (1.7 - 19.8)	32.8 (20.7 - 44.1)	24.8 (10.6 - 37.8)
percent of ATT false-positive	21.9 (2.8 - 64.6)	21.9 (2.9 - 64.9)	0.0 (-3.0 - 4.0)
referrals, inc. self-referrals	29.5 (17.0 - 42.9)	13.8 (8.0 - 21.0)	-15.6 (-25.8 - -4.9)
deaths	4.9 (0.9 - 10.0)	3.9 (0.7 - 8.3)	-1.0 (-2.8 - -0.1)
life-years lost	339.1 (62.7 - 692.0)	271.6 (49.2 - 570.9)	-67.5 (-189.8 - -4.1)
cost	15729.4 (6368.3 - 31027.5)	19297.7 (8413.8 - 35444.7)	3568.3 (-8472.2 - 16311.6)

For peer review only - http://bmjopen.bmj.com/site/about/guidelines.xhtml

Table A18 Indonesia

Quantity per 100 children with presumptive TB (unless stated):	Standard of care	Intervention	Difference
children with true TB	45.5 (8.7 - 85.0)	45.5 (8.7 - 85.0)	0.0 (0.0 - 0.0)
assessments	204.2 (173.4 - 233.5)	249.9 (211.2 - 286.5)	45.7 (31.9 - 58.0)
bacteriological investigations	24.7 (7.8 - 43.2)	103.0 (87.5 - 112.6)	78.2 (54.5 - 98.4)
anti-TB treatments (ATT)	33.3 (14.1 - 55.3)	39.5 (17.1 - 63.3)	6.2 (0.1 - 15.2)
ATT initiated at PHC	73.0 (63.2 - 80.3)	84.4 (73.2 - 91.2)	11.3 (7.1 - 15.4)
percent of true-positive receiving ATT	60.3 (48.2 - 71.4)	71.8 (65.9 - 77.3)	11.5 (1.8 - 23.1)
percent of ATT bacteriologically confirmed	5.9 (1.4 - 12.9)	32.5 (20.9 - 43.4)	26.6 (14.9 - 38.2)
percent of ATT false-positive	22.0 (2.8 - 65.1)	21.8 (2.9 - 64.6)	-0.3 (-3.5 - 3.5)
referrals, inc. self-referrals	33.0 (21.5 - 45.5)	14.5 (8.6 - 21.7)	-18.4 (-27.6 - -9.6)
deaths	5.4 (1.0 - 10.9)	4.7 (0.9 - 9.3)	-0.8 (-2.2 - 0.0)
life-years lost	391.6 (74.2 - 784.5)	336.7 (62.6 - 669.4)	-55.0 (-156.1 - 0.6)
cost	12508.1 (7056.4 - 20279.0)	14525.7 (8603.6 - 22403.0)	2017.6 (-5421.3 - 9470.6)

For peer review only - http://bmjopen.bmj.com/site/about/guidelines.xhtml

Age 0-4 years

Table A19 Ethiopia

Quantity per 100 children with presumptive TB (unless stated):	Standard of care	Intervention	Difference
children with true TB	45.5 (8.7 - 85.0)	45.5 (8.7 - 85.0)	0.0 (0.0 - 0.0)
assessments	208.1 (172.8 - 241.7)	252.9 (217.5 - 286.1)	44.7 (32.9 - 55.1)
bacteriological investigations	3.4 (2.1 - 4.9)	103.1 (87.6 - 112.5)	99.8 (84.3 - 109.1)
anti-TB treatments (ATT)	36.6 (15.3 - 59.7)	36.4 (15.2 - 59.3)	-0.2 (-2.8 - 2.7)
ATT initiated at PHC	68.3 (59.6 - 75.7)	81.0 (70.9 - 88.4)	12.7 (8.0 - 17.4)
percent of true-positive receiving ATT	66.2 (55.9 - 75.4)	66.8 (58.4 - 74.6)	0.7 (-3.4 - 5.1)
percent of ATT bacteriologically confirmed	0.6 (0.3 - 1.0)	31.4 (20.4 - 42.2)	30.8 (19.9 - 41.4)
percent of ATT false-positive	21.7 (2.4 - 65.8)	20.7 (2.4 - 63.4)	-1.0 (-4.2 - 1.9)
referrals, inc. self-referrals	40.1 (28.3 - 51.7)	14.7 (9.1 - 21.8)	-25.4 (-33.2 - -17.4)
deaths	7.5 (1.4 - 15.2)	7.3 (1.4 - 14.7)	-0.1 (-1.2 - 0.7)
life-years lost	514.5 (94.8 - 1046.0)	505.9 (93.8 - 1013.6)	-8.6 (-79.9 - 49.8)
cost	17934.1 (7124.0 - 35159.0)	17667.9 (7614.0 - 32685.5)	-266.2 (-13326.6 - 12081.4)

For peer review only

Table A20 Indonesia

Quantity per 100 children with presumptive TB (unless stated):	Standard of care	Intervention	Difference
children with true TB	45.5 (8.7 - 85.0)	45.5 (8.7 - 85.0)	0.0 (0.0 - 0.0)
assessments	209.4 (173.8 - 242.9)	255.2 (219.1 - 289.2)	45.9 (33.8 - 56.0)
bacteriological investigations	3.4 (2.1 - 5.0)	103.6 (88.2 - 113.0)	100.2 (84.9 - 109.5)
anti-TB treatments (ATT)	36.8 (15.4 - 60.4)	36.5 (15.2 - 59.6)	-0.3 (-3.0 - 2.4)
ATT initiated at PHC	70.1 (61.1 - 77.4)	83.6 (72.6 - 90.3)	13.4 (8.6 - 18.1)
percent of true-positive receiving ATT	66.6 (56.3 - 76.0)	67.0 (58.5 - 74.8)	0.4 (-3.7 - 4.8)
percent of ATT bacteriologically confirmed	0.6 (0.3 - 1.0)	31.4 (20.4 - 42.1)	30.8 (20.0 - 41.3)
percent of ATT false-positive	21.8 (2.4 - 65.8)	20.7 (2.4 - 63.7)	-1.1 (-4.3 - 1.9)
referrals, inc. self-referrals	41.5 (29.2 - 53.1)	15.3 (9.5 - 22.4)	-26.3 (-34.4 - -17.9)
deaths	7.4 (1.4 - 15.1)	7.3 (1.4 - 14.6)	-0.1 (-1.1 - 0.8)
life-years lost	530.6 (97.9 - 1085.3)	525.6 (98.7 - 1053.7)	-5.0 (-78.3 - 58.6)
cost	13672.3 (7286.9 - 22370.9)	14090.7 (8344.4 - 21727.0)	418.4 (-8064.8 - 9011.5)

For peer review only - http://bmjopen.bmj.com/site/about/guidelines.xhtml

Age 5-14 years

Table A21 Ethiopia

Quantity per 100 children with presumptive TB (unless stated):	Standard of care	Intervention	Difference
children with true TB	45.5 (8.7 - 85.0)	45.5 (8.7 - 85.0)	0.0 (0.0 - 0.0)
assessments	197.9 (169.6 - 225.2)	242.2 (200.8 - 281.7)	44.3 (24.8 - 62.7)
bacteriological investigations	47.1 (12.5 - 82.7)	101.9 (86.2 - 111.6)	54.8 (16.5 - 91.1)
anti-TB treatments (ATT)	29.6 (10.6 - 53.8)	42.6 (18.5 - 67.8)	13.0 (1.5 - 30.3)
ATT initiated at PHC	74.6 (63.8 - 84.3)	82.4 (71.8 - 90.2)	7.8 (2.1 - 12.9)
percent of true-positive receiving ATT	53.6 (31.9 - 72.9)	76.8 (69.4 - 83.1)	23.2 (5.1 - 45.4)
percent of ATT bacteriologically confirmed	14.5 (2.4 - 39.5)	33.7 (20.3 - 46.1)	19.2 (-5.9 - 37.4)
percent of ATT false-positive	21.6 (2.6 - 65.3)	22.4 (2.8 - 66.2)	0.8 (-2.5 - 6.2)
referrals, inc. self-referrals	23.1 (8.8 - 39.1)	13.3 (7.3 - 20.5)	-9.7 (-22.7 - 4.4)
deaths	3.4 (0.6 - 7.4)	1.9 (0.3 - 3.9)	-1.5 (-4.0 - -0.2)
life-years lost	234.0 (42.2 - 507.3)	131.2 (24.1 - 269.5)	-102.8 (-278.9 - -10.6)
cost	14407.8 (5303.7 - 29936.5)	20277.4 (8872.7 - 37127.0)	5869.6 (-6634.5 - 19361.5)

For peer review only - http://bmjopen.bmj.com/site/about/guidelines.xhtml

Table A22 Indonesia

Quantity per 100 children with presumptive TB (unless stated):	Standard of care	Intervention	Difference
children with true TB	45.5 (8.7 - 85.0)	45.5 (8.7 - 85.0)	0.0 (0.0 - 0.0)
assessments	198.8 (170.2 - 226.2)	244.3 (202.0 - 284.1)	45.5 (25.6 - 64.3)
bacteriological investigations	47.3 (12.7 - 83.3)	102.3 (86.7 - 112.2)	54.9 (16.6 - 91.6)
anti-TB treatments (ATT)	29.6 (10.5 - 54.2)	42.7 (18.5 - 68.1)	13.1 (1.4 - 30.8)
ATT initiated at PHC	77.2 (65.5 - 87.0)	85.1 (73.6 - 92.2)	7.9 (1.5 - 13.5)
percent of true-positive receiving ATT	53.6 (31.5 - 73.0)	76.9 (69.6 - 83.3)	23.3 (4.9 - 45.9)
percent of ATT bacteriologically confirmed	14.1 (2.3 - 38.3)	33.7 (20.2 - 46.1)	19.5 (-4.9 - 37.4)
percent of ATT false-positive	21.7 (2.6 - 65.3)	22.4 (2.8 - 66.3)	0.8 (-2.6 - 6.1)
referrals, inc. self-referrals	23.9 (9.1 - 40.3)	13.8 (7.5 - 21.2)	-10.1 (-23.7 - 4.3)
deaths	3.4 (0.6 - 7.4)	1.9 (0.4 - 3.9)	-1.5 (-4.1 - -0.1)
life-years lost	244.3 (44.1 - 531.9)	136.3 (25.4 - 281.0)	-108.0 (-292.8 - -10.8)
cost	11270.2 (6013.6 - 18958.7)	14987.1 (8815.0 - 23229.9)	3716.9 (-3812.4 - 10646.6)

For peer review only - http://bmjopen.bmj.com/site/about/guidelines.xhtml

Results for 5% discount rate sensitivity analysis

Age-specific results

All ages: 0-14 years

Table A23 Ethiopia

**Quantity per 100 children with presumptive TB (unless stated):**	**Standard of care**	**Intervention**	**Difference**
children with true TB	45.5 (8.7 - 85.0)	45.5 (8.7 - 85.0)	0.0 (0.0 - 0.0)
assessments	201.8 (171.8 - 230.9)	246.2 (207.3 - 283.3)	44.4 (29.5 - 58.1)
bacteriological investigations	30.7 (8.7 - 57.5)	102.3 (86.8 - 112.0)	71.7 (41.5 - 96.3)
anti-TB treatments (ATT)	32.2 (13.2 - 54.5)	40.3 (17.6 - 64.4)	8.1 (0.6 - 20.3)
ATT initiated at PHC	71.8 (62.3 - 79.6)	81.9 (71.6 - 89.5)	10.1 (5.8 - 14.2)
percent of true-positive receiving ATT	58.3 (43.0 - 71.1)	73.0 (66.7 - 78.8)	14.7 (2.8 - 30.5)
percent of ATT bacteriologically confirmed	8.0 (1.7 - 19.8)	32.8 (20.7 - 44.1)	24.8 (10.6 - 37.8)
percent of ATT false-positive	21.9 (2.8 - 64.6)	21.9 (2.9 - 64.9)	0.0 (-3.0 - 4.0)
referrals, inc. self-referrals	29.5 (17.0 - 42.9)	13.8 (8.0 - 21.0)	-15.6 (-25.8 - -4.9)
deaths	4.9 (0.9 - 10.0)	3.9 (0.7 - 8.3)	-1.0 (-2.8 - -0.1)
life-years lost	89.0 (16.5 - 181.7)	71.3 (12.9 - 149.9)	-17.7 (-49.8 - -1.1)
cost	15729.4 (6368.3 - 31027.5)	19297.7 (8413.8 - 35444.7)	3568.3 (-8472.2 - 16311.6)

For peer review only - http://bmjopen.bmj.com/site/about/guidelines.xhtml

Table A24 Indonesia

Quantity per 100 children with presumptive TB (unless stated):	Standard of care	Intervention	Difference
children with true TB	45.5 (8.7 - 85.0)	45.5 (8.7 - 85.0)	0.0 (0.0 - 0.0)
assessments	204.2 (173.4 - 233.5)	249.9 (211.2 - 286.5)	45.7 (31.9 - 58.0)
bacteriological investigations	24.7 (7.8 - 43.2)	103.0 (87.5 - 112.6)	78.2 (54.5 - 98.4)
anti-TB treatments (ATT)	33.3 (14.1 - 55.3)	39.5 (17.1 - 63.3)	6.2 (0.1 - 15.2)
ATT initiated at PHC	73.0 (63.2 - 80.3)	84.4 (73.2 - 91.2)	11.3 (7.1 - 15.4)
percent of true-positive receiving ATT	60.3 (48.2 - 71.4)	71.8 (65.9 - 77.3)	11.5 (1.8 - 23.1)
percent of ATT bacteriologically confirmed	5.9 (1.4 - 12.9)	32.5 (20.9 - 43.4)	26.6 (14.9 - 38.2)
percent of ATT false-positive	22.0 (2.8 - 65.1)	21.8 (2.9 - 64.6)	-0.3 (-3.5 - 3.5)
referrals, inc. self-referrals	33.0 (21.5 - 45.5)	14.5 (8.6 - 21.7)	-18.4 (-27.6 - -9.6)
deaths	5.4 (1.0 - 10.9)	4.7 (0.9 - 9.3)	-0.8 (-2.2 - 0.0)
life-years lost	101.1 (19.2 - 202.4)	86.9 (16.2 - 172.7)	-14.2 (-40.3 - 0.2)
cost	12508.1 (7056.4 - 20279.0)	14525.7 (8603.6 - 22403.0)	2017.6 (-5421.3 - 9470.6)

For peer review only - http://bmjopen.bmj.com/site/about/guidelines.xhtml

Age 0-4 years

Table A25 Ethiopia

Quantity per 100 children with presumptive TB (unless stated):	Standard of care	Intervention	Difference
children with true TB	45.5 (8.7 - 85.0)	45.5 (8.7 - 85.0)	0.0 (0.0 - 0.0)
assessments	208.1 (172.8 - 241.7)	252.9 (217.5 - 286.1)	44.7 (32.9 - 55.1)
bacteriological investigations	3.4 (2.1 - 4.9)	103.1 (87.6 - 112.5)	99.8 (84.3 - 109.1)
anti-TB treatments (ATT)	36.6 (15.3 - 59.7)	36.4 (15.2 - 59.3)	-0.2 (-2.8 - 2.7)
ATT initiated at PHC	68.3 (59.6 - 75.7)	81.0 (70.9 - 88.4)	12.7 (8.0 - 17.4)
percent of true-positive receiving ATT	66.2 (55.9 - 75.4)	66.8 (58.4 - 74.6)	0.7 (-3.4 - 5.1)
percent of ATT bacteriologically confirmed	0.6 (0.3 - 1.0)	31.4 (20.4 - 42.2)	30.8 (19.9 - 41.4)
percent of ATT false-positive	21.7 (2.4 - 65.8)	20.7 (2.4 - 63.4)	-1.0 (-4.2 - 1.9)
referrals, inc. self-referrals	40.1 (28.3 - 51.7)	14.7 (9.1 - 21.8)	-25.4 (-33.2 - -17.4)
deaths	7.5 (1.4 - 15.2)	7.3 (1.4 - 14.7)	-0.1 (-1.2 - 0.7)
life-years lost	135.1 (24.9 - 274.6)	132.8 (24.6 - 266.1)	-2.3 (-21.0 - 13.1)
cost	17934.1 (7124.0 - 35159.0)	17667.9 (7614.0 - 32685.5)	-266.2 (-13326.6 - 12081.4)

For peer review only - http://bmjopen.bmj.com/site/about/guidelines.xhtml

Table A26 Indonesia

Quantity per 100 children with presumptive TB (unless stated):	Standard of care	Intervention	Difference
children with true TB	45.5 (8.7 - 85.0)	45.5 (8.7 - 85.0)	0.0 (0.0 - 0.0)
assessments	209.4 (173.8 - 242.9)	255.2 (219.1 - 289.2)	45.9 (33.8 - 56.0)
bacteriological investigations	3.4 (2.1 - 5.0)	103.6 (88.2 - 113.0)	100.2 (84.9 - 109.5)
anti-TB treatments (ATT)	36.8 (15.4 - 60.4)	36.5 (15.2 - 59.6)	-0.3 (-3.0 - 2.4)
ATT initiated at PHC	70.1 (61.1 - 77.4)	83.6 (72.6 - 90.3)	13.4 (8.6 - 18.1)
percent of true-positive receiving ATT	66.6 (56.3 - 76.0)	67.0 (58.5 - 74.8)	0.4 (-3.7 - 4.8)
percent of ATT bacteriologically confirmed	0.6 (0.3 - 1.0)	31.4 (20.4 - 42.1)	30.8 (20.0 - 41.3)
percent of ATT false-positive	21.8 (2.4 - 65.8)	20.7 (2.4 - 63.7)	-1.1 (-4.3 - 1.9)
referrals, inc. self-referrals	41.5 (29.2 - 53.1)	15.3 (9.5 - 22.4)	-26.3 (-34.4 - -17.9)
deaths	7.4 (1.4 - 15.1)	7.3 (1.4 - 14.6)	-0.1 (-1.1 - 0.8)
life-years lost	136.9 (25.3 - 280.0)	135.6 (25.5 - 271.9)	-1.3 (-20.2 - 15.1)
cost	13672.3 (7286.9 - 22370.9)	14090.7 (8344.4 - 21727.0)	418.4 (-8064.8 - 9011.5)

For peer review only - http://bmjopen.bmj.com/site/about/guidelines.xhtml

Age 5-14 years

Table A27 Ethiopia

Quantity per 100 children with presumptive TB (unless stated):	Standard of care	Intervention	Difference
children with true TB	45.5 (8.7 - 85.0)	45.5 (8.7 - 85.0)	0.0 (0.0 - 0.0)
assessments	197.9 (169.6 - 225.2)	242.2 (200.8 - 281.7)	44.3 (24.8 - 62.7)
bacteriological investigations	47.1 (12.5 - 82.7)	101.9 (86.2 - 111.6)	54.8 (16.5 - 91.1)
anti-TB treatments (ATT)	29.6 (10.6 - 53.8)	42.6 (18.5 - 67.8)	13.0 (1.5 - 30.3)
ATT initiated at PHC	74.6 (63.8 - 84.3)	82.4 (71.8 - 90.2)	7.8 (2.1 - 12.9)
percent of true-positive receiving ATT	53.6 (31.9 - 72.9)	76.8 (69.4 - 83.1)	23.2 (5.1 - 45.4)
percent of ATT bacteriologically confirmed	14.5 (2.4 - 39.5)	33.7 (20.3 - 46.1)	19.2 (-5.9 - 37.4)
percent of ATT false-positive	21.6 (2.6 - 65.3)	22.4 (2.8 - 66.2)	0.8 (-2.5 - 6.2)
referrals, inc. self-referrals	23.1 (8.8 - 39.1)	13.3 (7.3 - 20.5)	-9.7 (-22.7 - 4.4)
deaths	3.4 (0.6 - 7.4)	1.9 (0.3 - 3.9)	-1.5 (-4.0 - -0.2)
life-years lost	61.4 (11.1 - 133.2)	34.5 (6.3 - 70.8)	-27.0 (-73.2 - -2.8)
cost	14407.8 (5303.7 - 29936.5)	20277.4 (8872.7 - 37127.0)	5869.6 (-6634.5 - 19361.5)

For peer review only - http://bmjopen.bmj.com/site/about/guidelines.xhtml

Table A28 Indonesia

Quantity per 100 children with presumptive TB (unless stated):	Standard of care	Intervention	Difference
children with true TB	45.5 (8.7 - 85.0)	45.5 (8.7 - 85.0)	0.0 (0.0 - 0.0)
assessments	198.8 (170.2 - 226.2)	244.3 (202.0 - 284.1)	45.5 (25.6 - 64.3)
bacteriological investigations	47.3 (12.7 - 83.3)	102.3 (86.7 - 112.2)	54.9 (16.6 - 91.6)
anti-TB treatments (ATT)	29.6 (10.5 - 54.2)	42.7 (18.5 - 68.1)	13.1 (1.4 - 30.8)
ATT initiated at PHC	77.2 (65.5 - 87.0)	85.1 (73.6 - 92.2)	7.9 (1.5 - 13.5)
percent of true-positive receiving ATT	53.6 (31.5 - 73.0)	76.9 (69.6 - 83.3)	23.3 (4.9 - 45.9)
percent of ATT bacteriologically confirmed	14.1 (2.3 - 38.3)	33.7 (20.2 - 46.1)	19.5 (-4.9 - 37.4)
percent of ATT false-positive	21.7 (2.6 - 65.3)	22.4 (2.8 - 66.3)	0.8 (-2.6 - 6.1)
referrals, inc. self-referrals	23.9 (9.1 - 40.3)	13.8 (7.5 - 21.2)	-10.1 (-23.7 - 4.3)
deaths	3.4 (0.6 - 7.4)	1.9 (0.4 - 3.9)	-1.5 (-4.1 - -0.1)
life-years lost	63.0 (11.4 - 137.2)	35.2 (6.5 - 72.5)	-27.9 (-75.5 - -2.8)
cost	11270.2 (6013.6 - 18958.7)	14987.1 (8815.0 - 23229.9)	3716.9 (-3812.4 - 10646.6)

For peer review only - http://bmjopen.bmj.com/site/about/guidelines.xhtml

Comparison of ICERs for sensitivity analyses

Table A29 ICERs by sensitivity analysis for each country

scenario	Ethiopia	Indonesia
basecase	132.2	93.8
Xpert SOC	137.8	114.8
Low prevalence	178.1	150.4
0% discount rate	54.8	38.3
5% discount rate	199.3	142.2

For peer review only

For peer review only - http://bmjopen.bmj.com/site/about/guidelines.xhtml

Xpert Ultra stool testing to diagnose tuberculosis in children in Ethiopia and Indonesia: a model-based cost-effectiveness analysis.

Appendix 4: Consolidated Health Economic Evaluation Reporting Standards (CHEERS) checklist – Items to include when reporting economic evaluations of health interventions

Section/item	Item No	Recommendation	Reported on page No/ line No
**Title and abstract**			
Title	1	Identify the study as an economic evaluation or use more specific terms such as "cost-effectiveness analysis", and describe the interventions compared.	page 1, line 1 to 2
Abstract	2	Provide a structured summary of objectives, perspective, setting, methods (including study design and inputs), results (including base case and uncertainty analyses), and conclusions.	page 2, line 1 to 32
**Introduction**			
Background and objectives	3	Provide an explicit statement of the broader context for the study.	page 4, line 33 to 38
		Present the study question and its relevance for health policy or practice decisions.	page 4, line 33 to 34
**Methods**			
Target population and subgroups	4	Describe characteristics of the base case population and subgroups analysed, including why they were chosen.	page 5, line 1 to 12; page 6, line 4 to 9
Setting and location	5	State relevant aspects of the system(s) in which the decision(s) need(s) to be made.	page 4, line 39 to 44; page 5, line 3 to 26
Study perspective	6	Describe the perspective of the study and relate this to the costs being evaluated.	page 7, line 2
Comparators	7	Describe the interventions or strategies being compared and state why they were chosen.	page 3, line 4 to 32; page 5, line 14 to 32
Time horizon	8	State the time horizon(s) over which costs and consequences are being evaluated and say why appropriate.	page 7, line 5; page 7, line 24 to 25
Discount rate	9	Report the choice of discount rate(s) used for costs and outcomes and say why appropriate.	page 7, line 5; page 7, line 25
Choice of health outcomes	10	Describe what outcomes were used as the measure(s) of benefit in the evaluation and their relevance for the type of analysis performed.	page 7, line 24 to 33;
Measurement of effectiveness	11a	*Single study-based estimates:* Describe fully the design features of the single effectiveness study and why the single study was a sufficient source of clinical effectiveness data.	not applicable
	11b	*Synthesis-based estimates*: Describe fully the methods used for identification of included studies and synthesis of clinical effectiveness data.	page 6, line 17 to 35; page8, line 7 to 16; Table 1; Appendix 2a
Measurement and valuation of preference based outcomes	12	If applicable, describe the population and methods used to elicit preferences for outcomes.	not applicable
Estimating resources and costs	13a	*Single study-based economic evaluation:* Describe approaches used to estimate resource use associated with the alternative interventions. Describe primary or secondary research methods for valuing each resource	not applicable

For peer review only

Section/item	Item No	Recommendation	Reported on page No/ line No
		item in terms of its unit cost. Describe any adjustments made to approximate to opportunity costs.	
	13b	*Model-based economic evaluation:* Describe approaches and data sources used to estimate resource use associated with model health states. Describe primary or secondary research methods for valuing each resource item in terms of its unit cost. Describe any adjustments made to approximate to opportunity costs.	page 7, line 1 to 40; Table 2; Appendix 2b
Currency, price date, and conversion	14	Report the dates of the estimated resource quantities and unit costs. Describe methods for adjusting estimated unit costs to the year of reported costs if necessary. Describe methods for converting costs into a common currency base and the exchange rate.	page 7 line 2 to 5; Appendix 2b
Choice of model	15	Describe and give reasons for the specific type of decision-analytical model used. Providing a figure to show model structure is strongly recommended.	page 6, line 4 to 15; Figure 1; Appendix 2a, Figure 1
Assumptions	16	Describe all structural or other assumptions underpinning the decision-analytical model.	page 5, line 3 to 40; page 6, line 1 to 15; Figure 1; Appendix 2a, Figure 1
Analytical methods	17	Describe all analytical methods supporting the evaluation. This could include methods for dealing with skewed, missing, or censored data; extrapolation methods; methods for pooling data; approaches to validate or make adjustments (such as half cycle corrections) to a model; and methods for handling population heterogeneity and uncertainty.	page 7, line 29 to 40
**Results**			
Study parameters	18	Report the values, ranges, references, and, if used, probability distributions for all parameters. Report reasons or sources for distributions used to represent uncertainty where appropriate. Providing a table to show the input values is strongly recommended.	page 17, table 1; page 18, table 2: Appendix 2a; Appendix 2b
Incremental costs and outcomes	19	For each intervention, report mean values for the main categories of estimated costs and outcomes of interest, as well as mean differences between the comparator groups. If applicable, report incremental cost-effectiveness ratios.	page 8, line 17 to 38; page 21, Table 3; Appendix 3
Characterising uncertainty	20a	*Single study-based economic evaluation:* Describe the effects of sampling uncertainty for the estimated incremental cost and incremental effectiveness parameters, together with the impact of methodological assumptions (such as discount rate, study perspective).	not applicable
	20b	*Model-based economic evaluation:* Describe the effects on the results of uncertainty for all input parameters, and uncertainty related to the structure of the model and assumptions.	page 9, line 2-16; Figure 2: Figure 3; Appendix 3
Characterising heterogeneity	21	If applicable, report differences in costs, outcomes, or cost-effectiveness that can be explained by variations between subgroups of patients with different baseline characteristics or other observed variability in effects that are not reducible by more information.	page 8, line 25 to 38; Table 3; Appendix 3
**Discussion**			
Study findings, limitations,	22	Summarise key study findings and describe how they support the conclusions reached. Discuss limitations	page 9, line 18-29; page 9, line 40-41; page 10, line 1-32;

For peer review only

Section/item	Item No	Recommendation	Reported on page No/ line No
generalisability, and current knowledge		and the generalisability of the findings and how the findings fit with current knowledge.	
**Other**			
Source of funding	23	Describe how the study was funded and the role of the funder in the identification, design, conduct, and reporting of the analysis. Describe other non-monetary sources of support.	page 12, line 16 to 20; Information provided via the submission system
Conflicts of interest	24	Describe any potential for conflict of interest of study contributors in accordance with journal policy. In the absence of a journal policy, we recommend authors comply with International Committee of Medical Journal Editors recommendations.	page 12, line 15; Information provided via the submission system

For consistency, the CHEERS statement checklist format is based on the format of the CONSORT statement checklist

For peer review only
